# Supplementary figures and images for: Fine-mapping a genome-wide meta-analysis of 98,374 migraine cases identifies 181 sets of candidate causal variants
Source: medRxiv. 2024 May 20:2024.05.20.24307608. Preprint. [Version 1] doi: 10.1101/2024.05.20.24307608 (PMC11451805; doi:10.1101/2024.05.20.24307608)

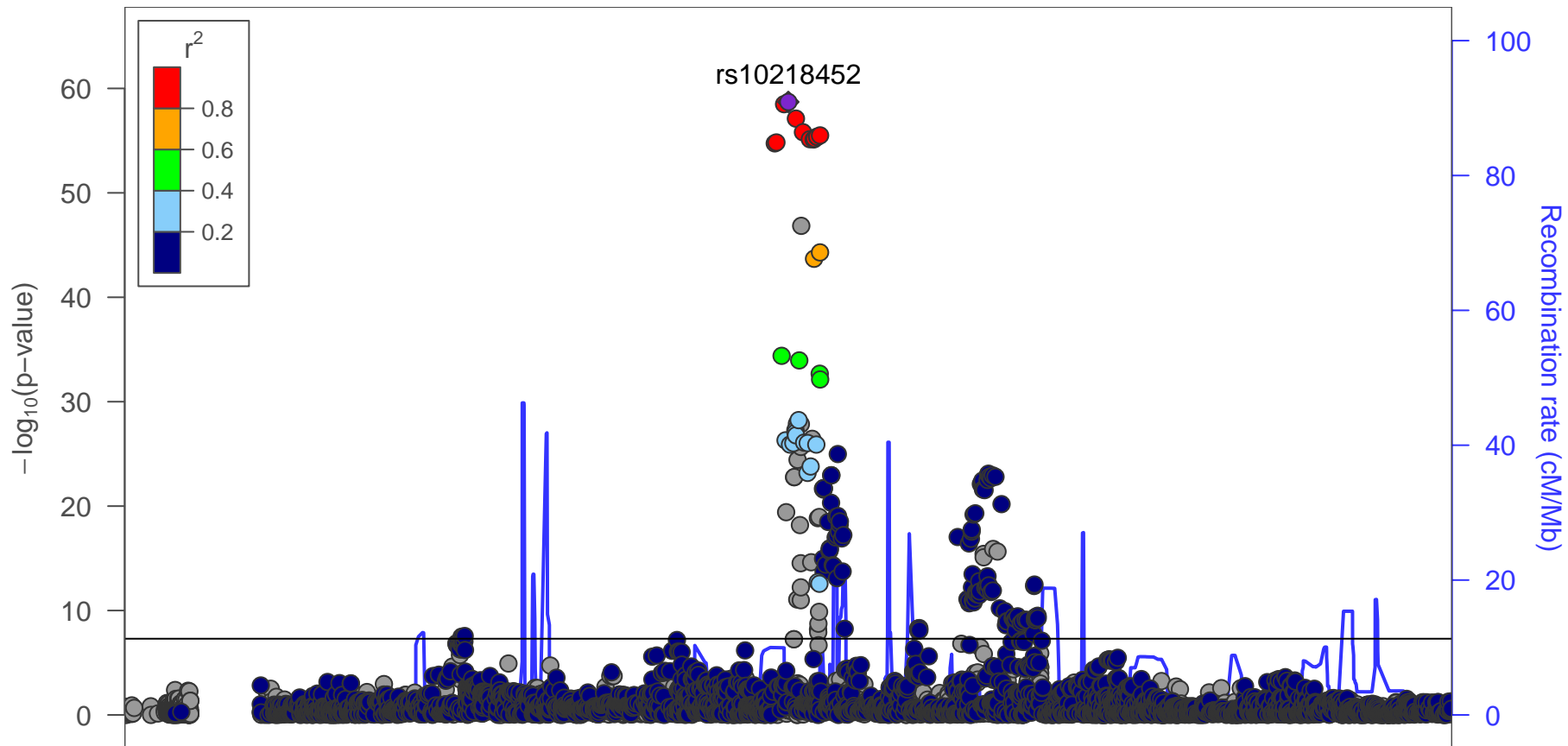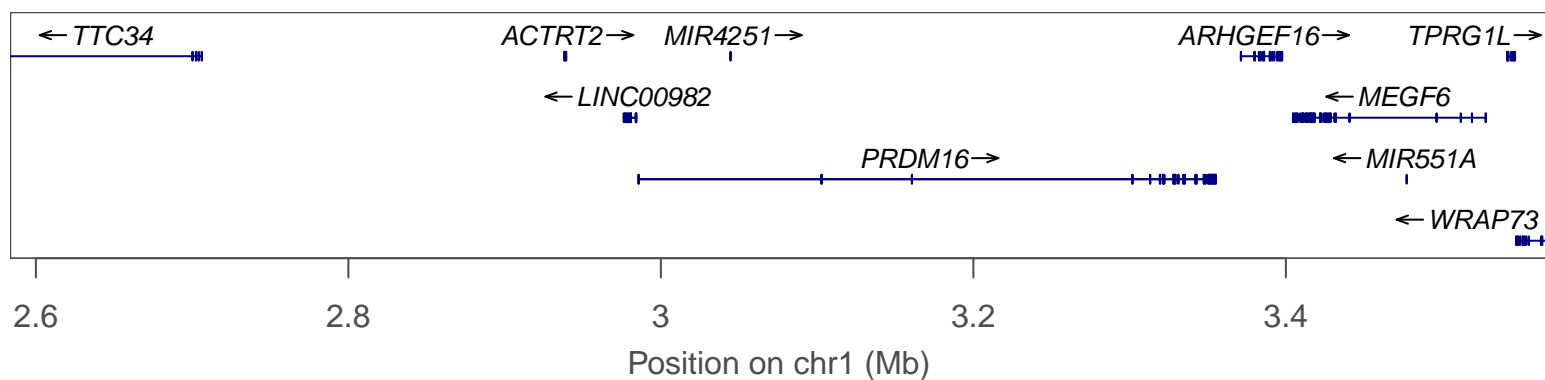

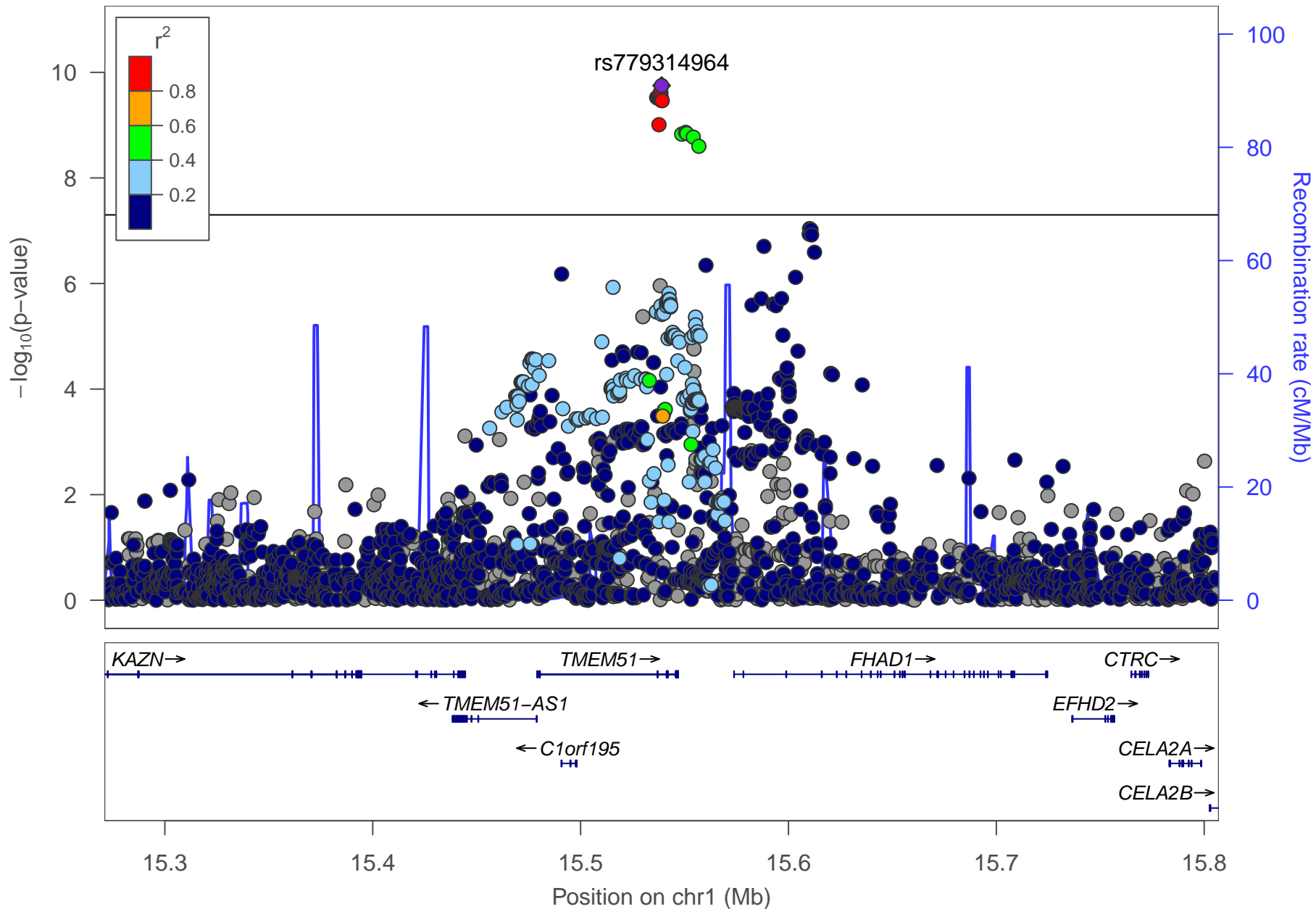

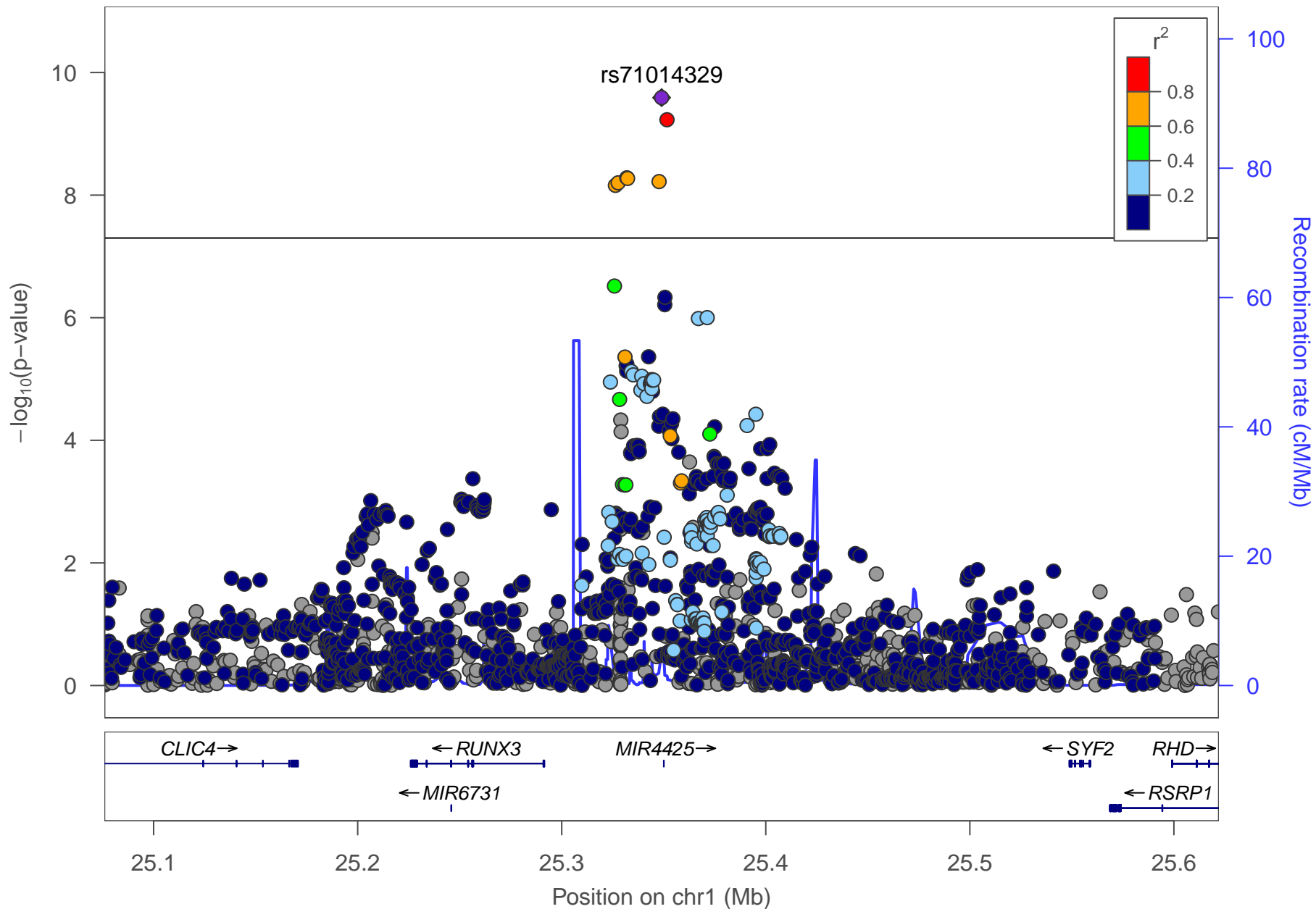

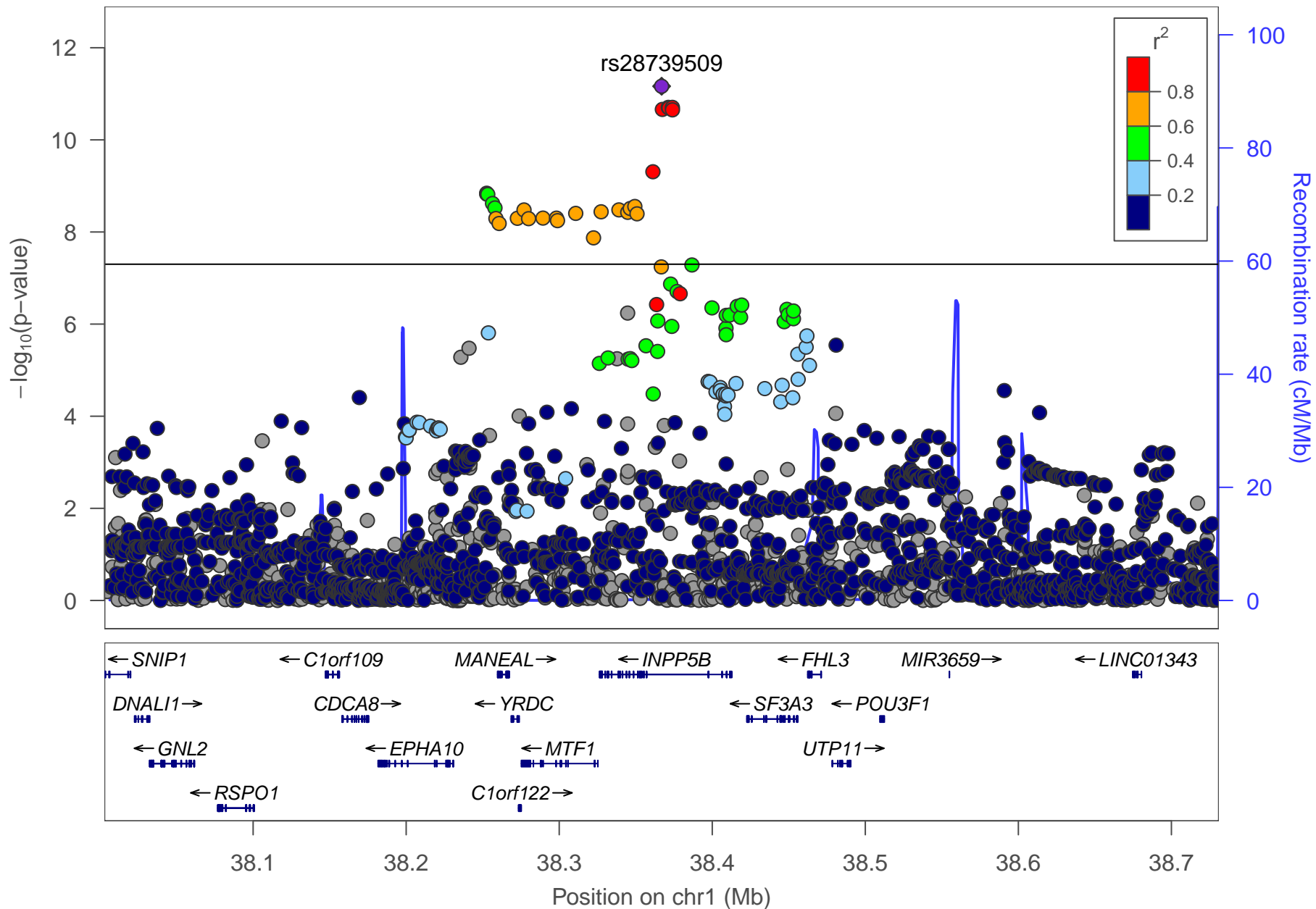

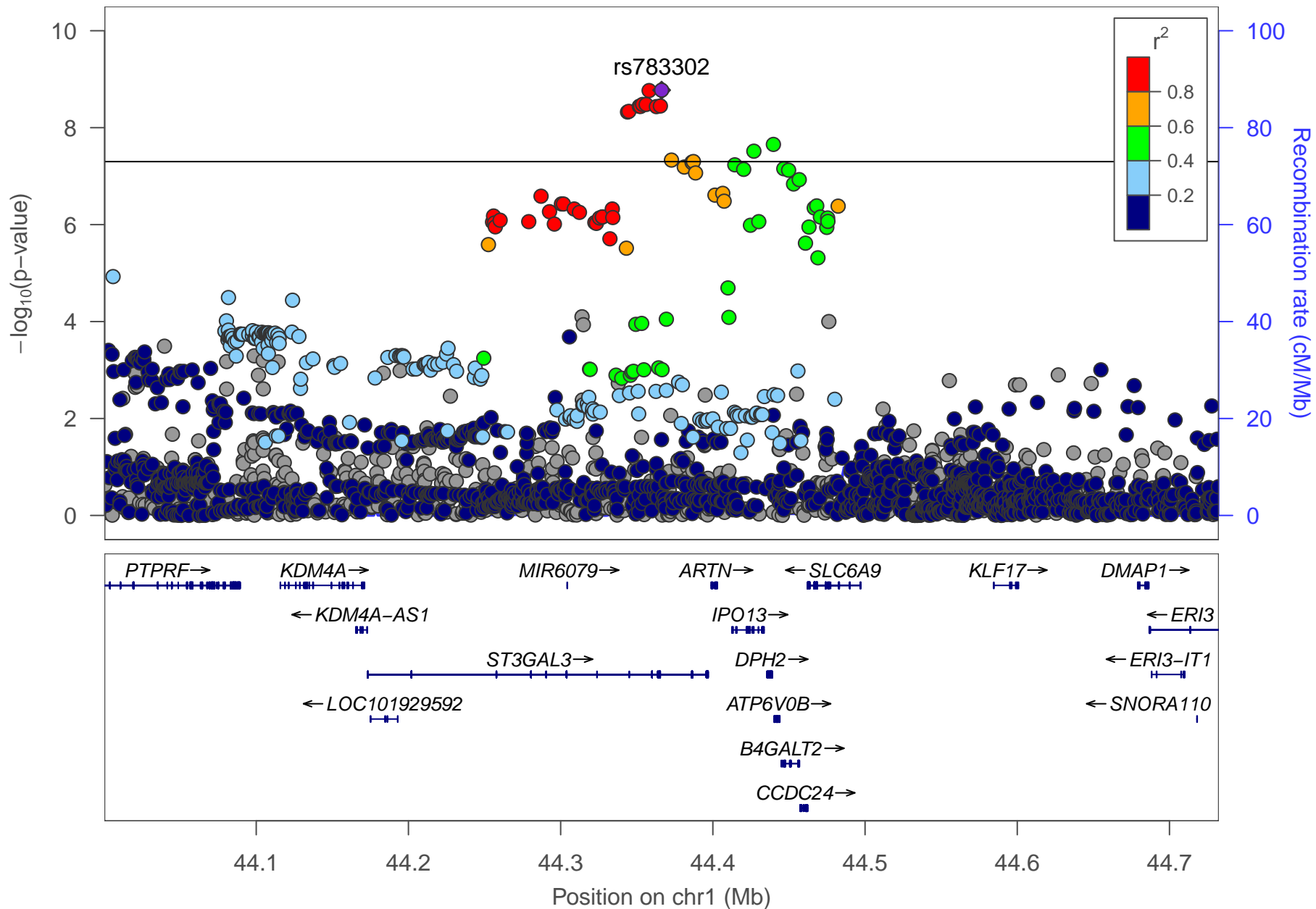

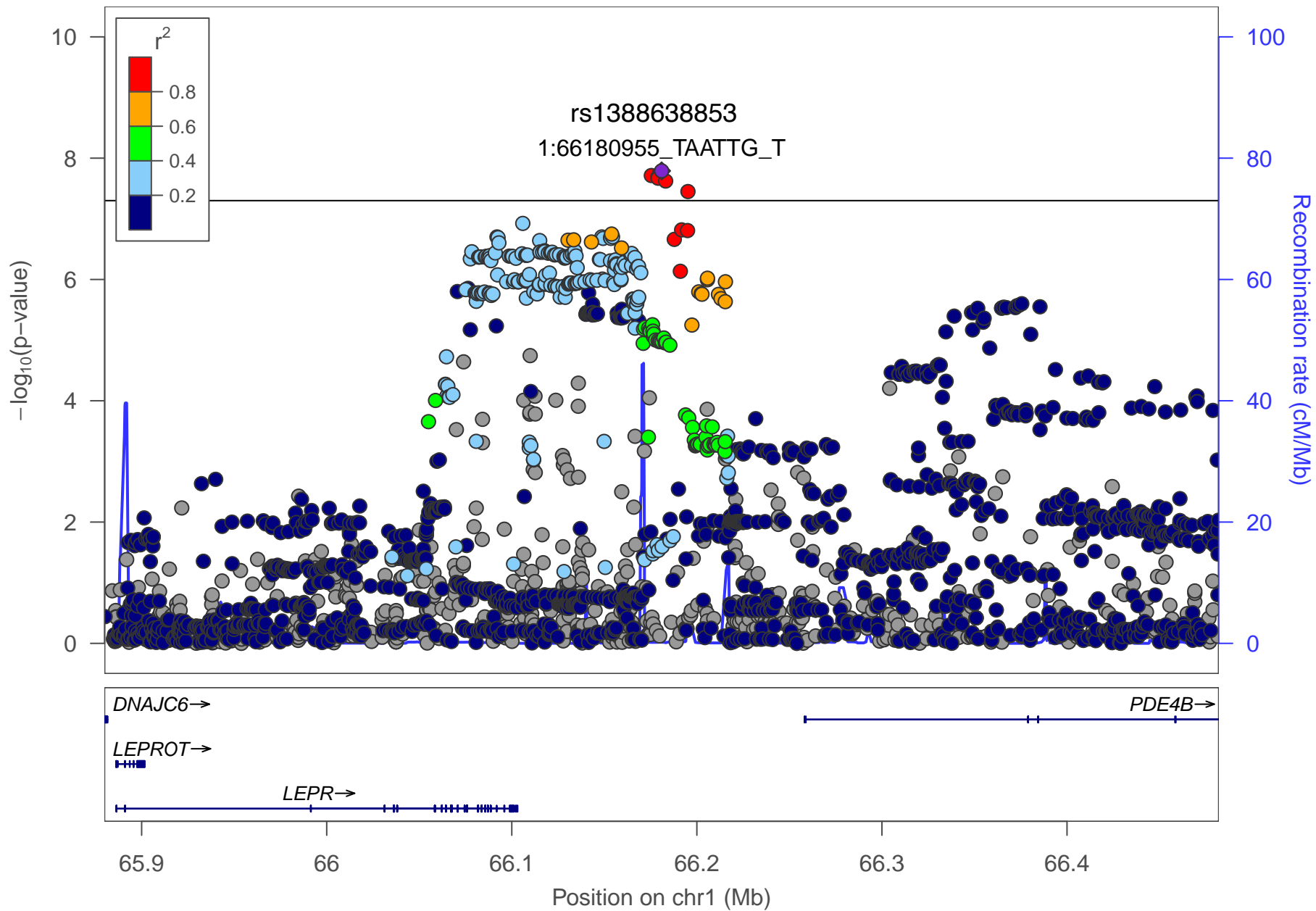

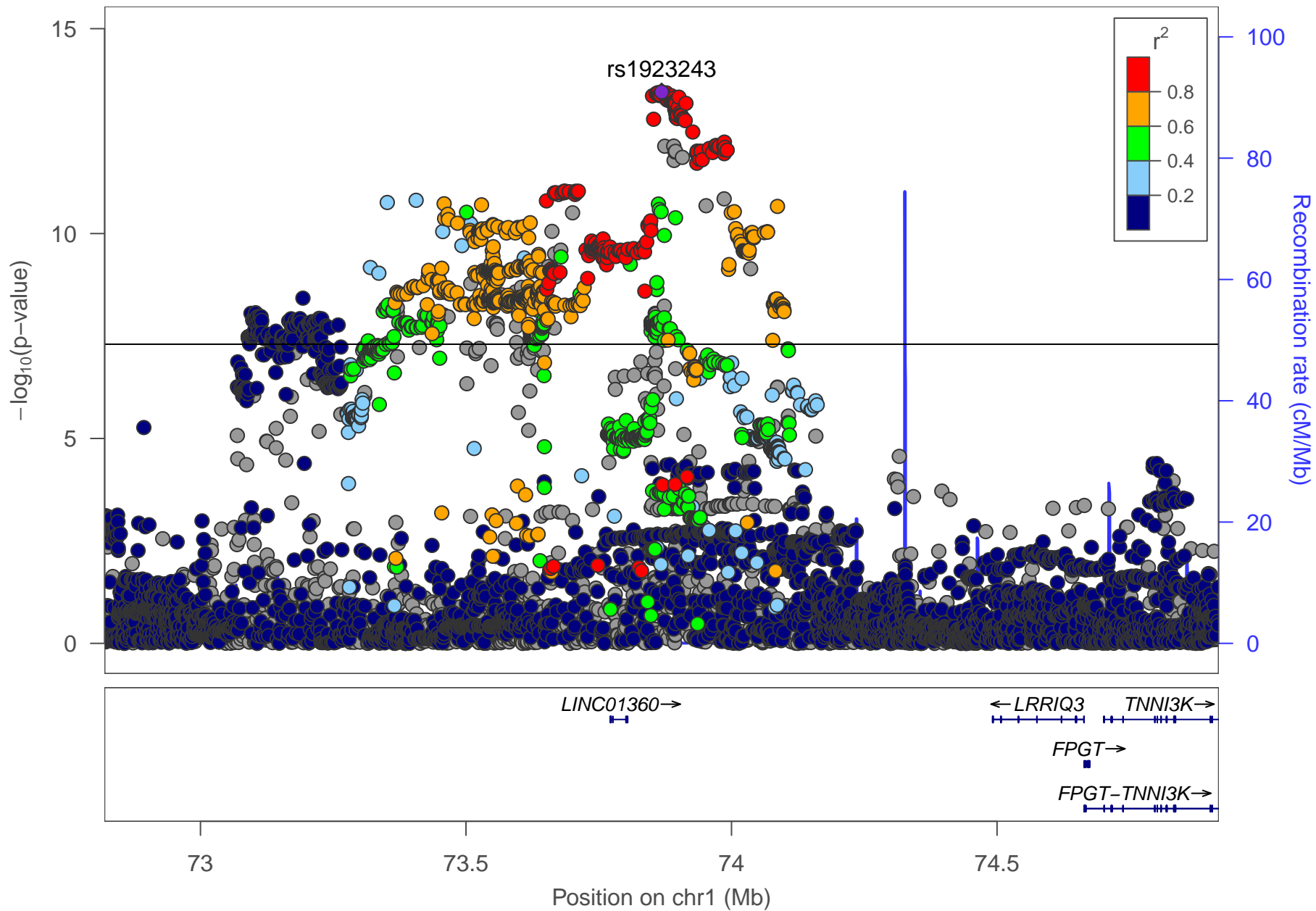

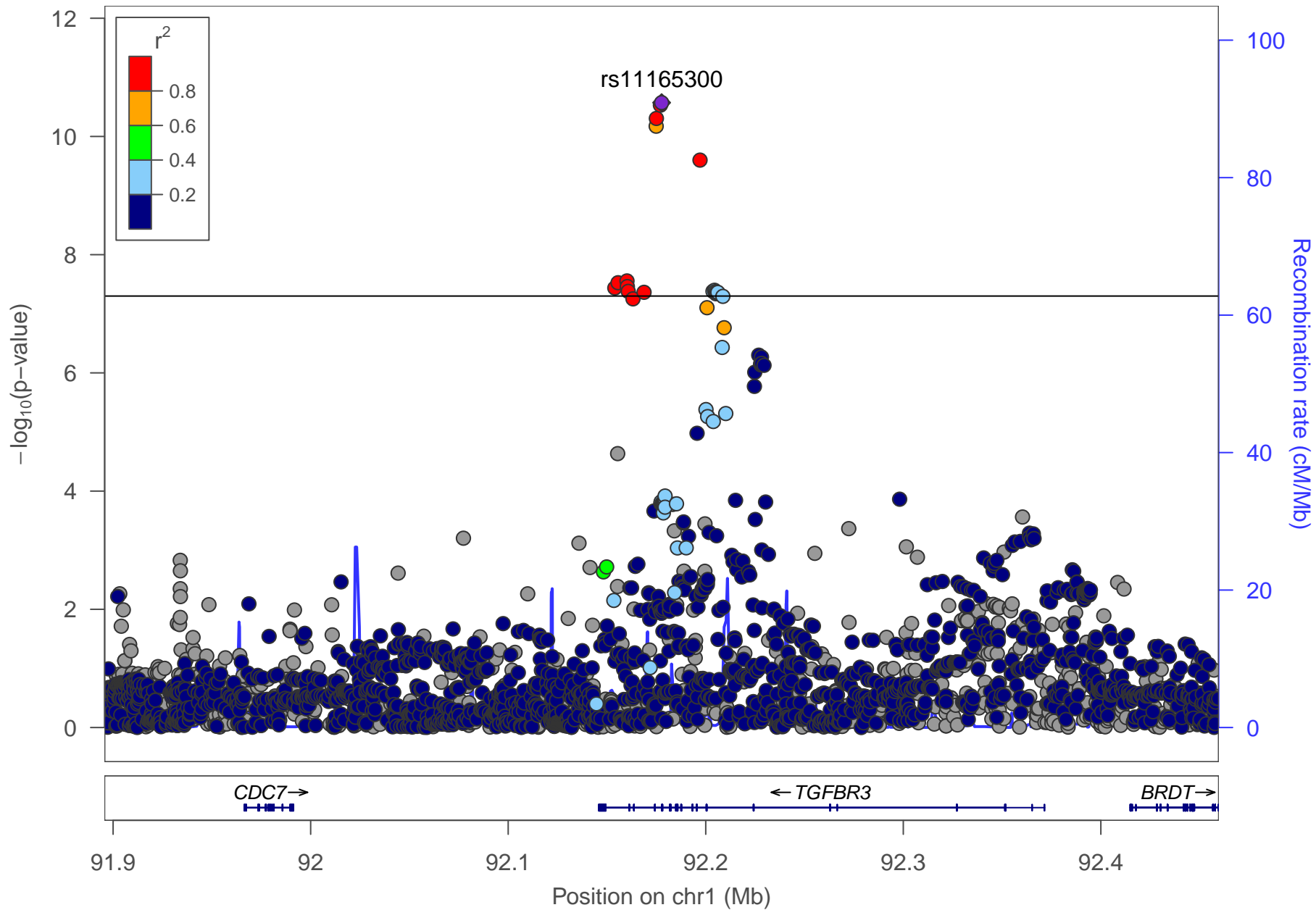

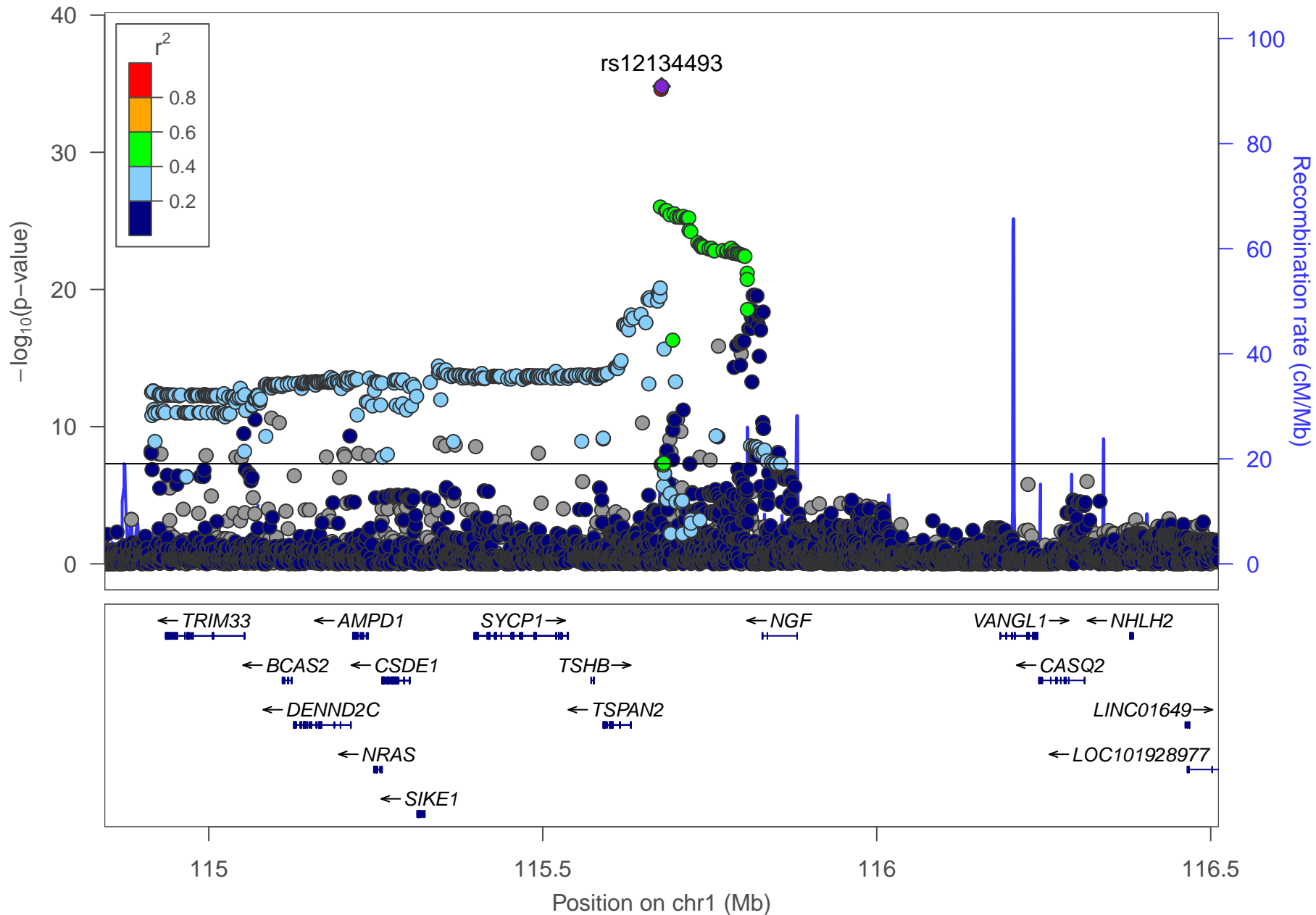

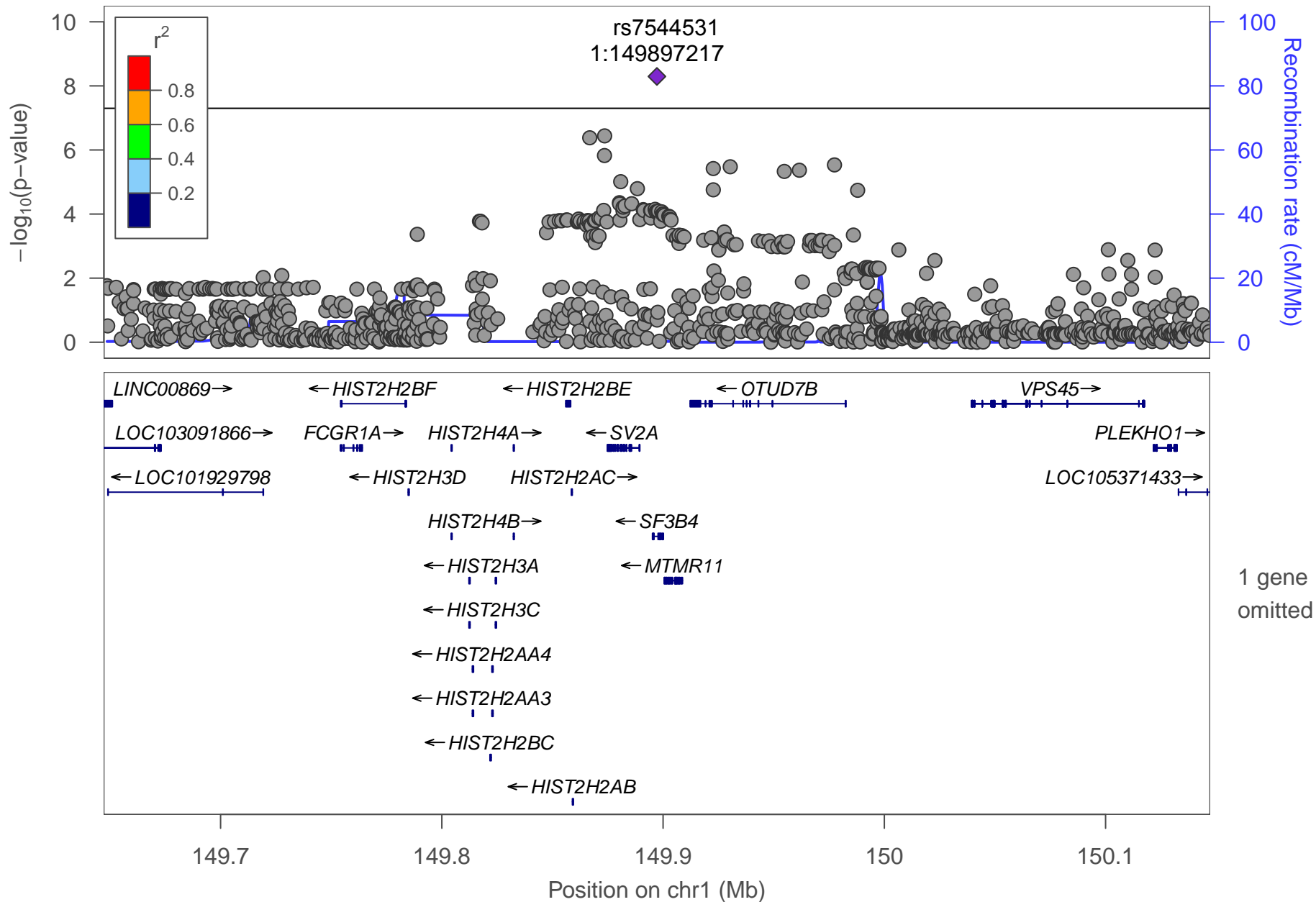

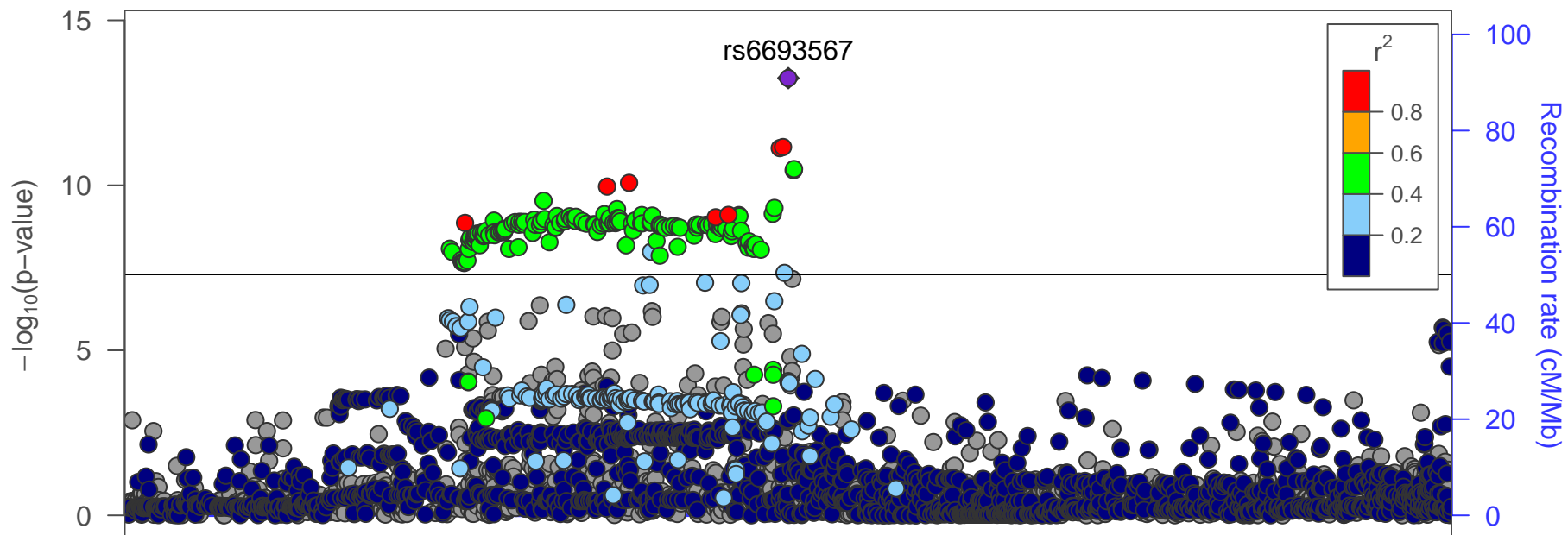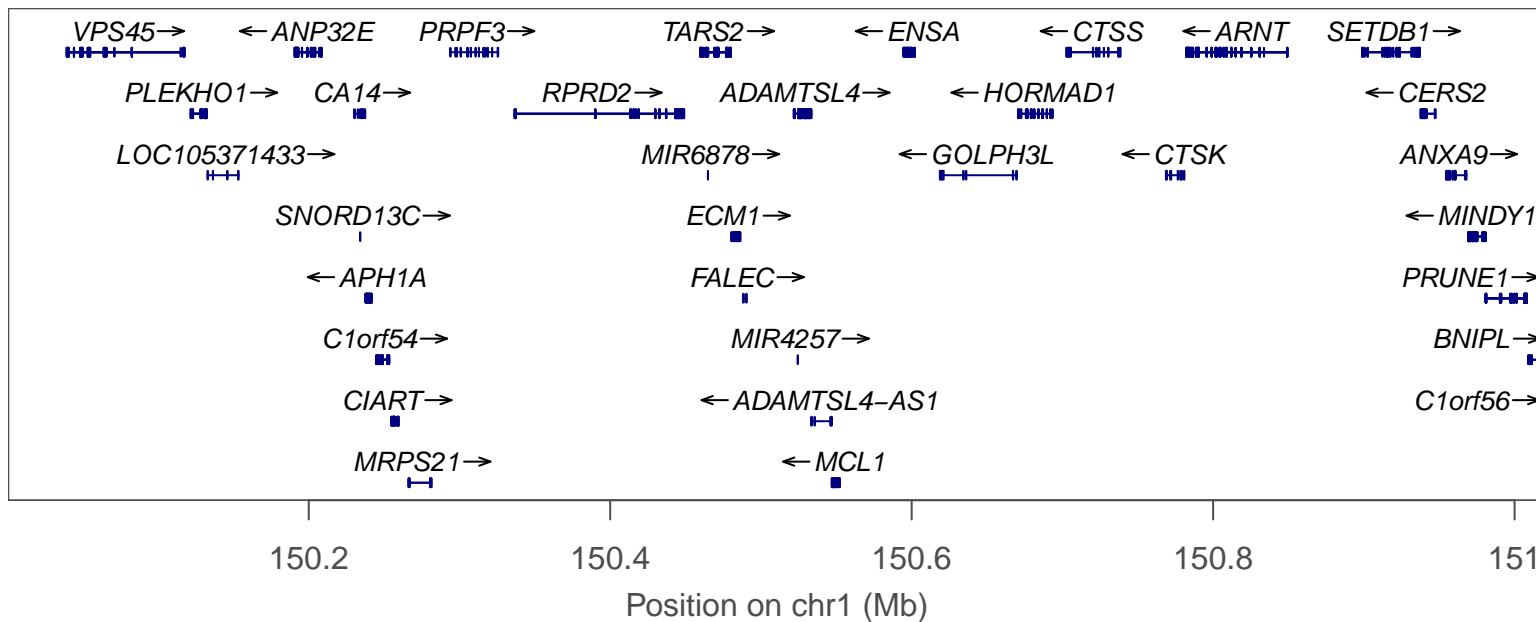

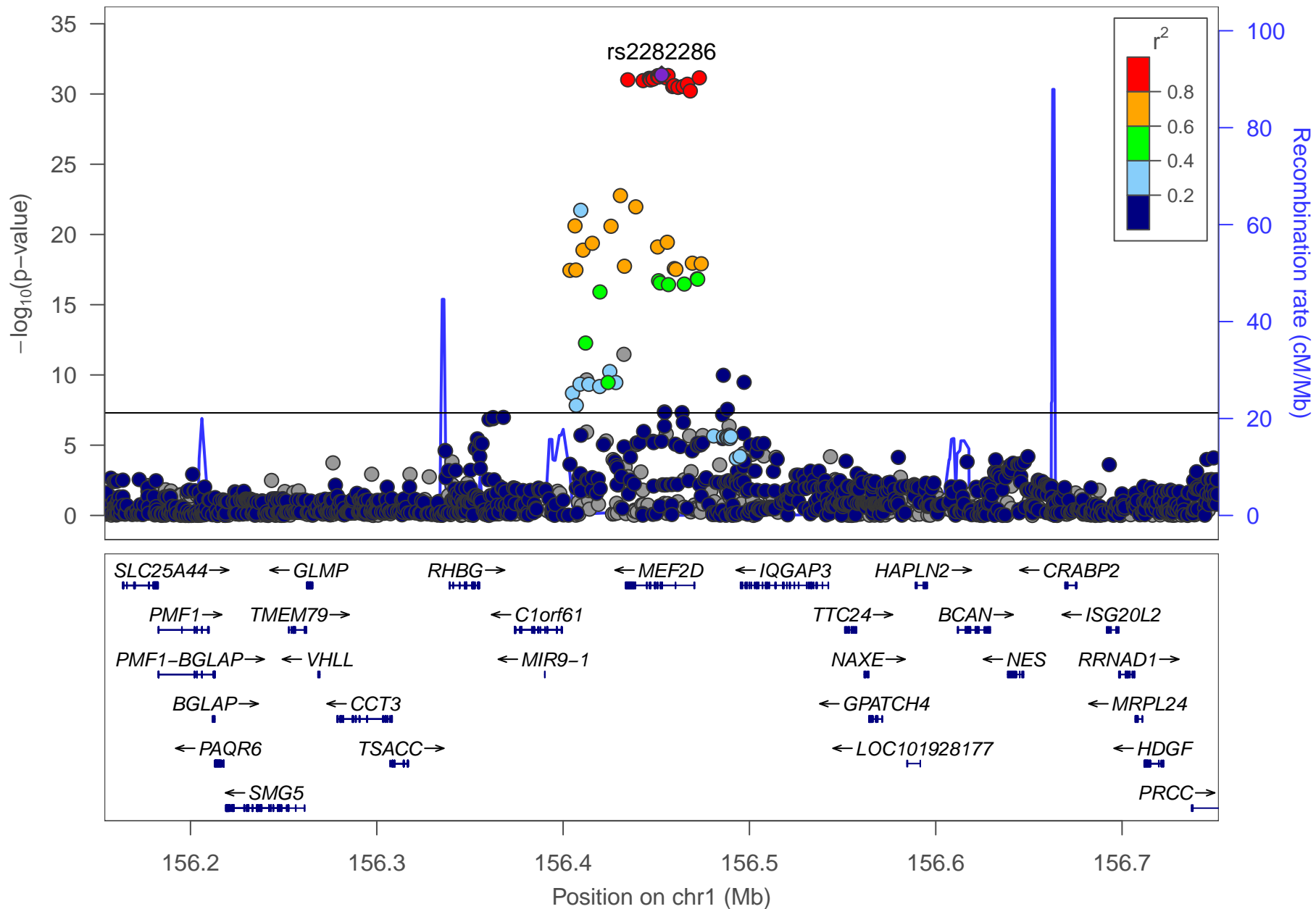

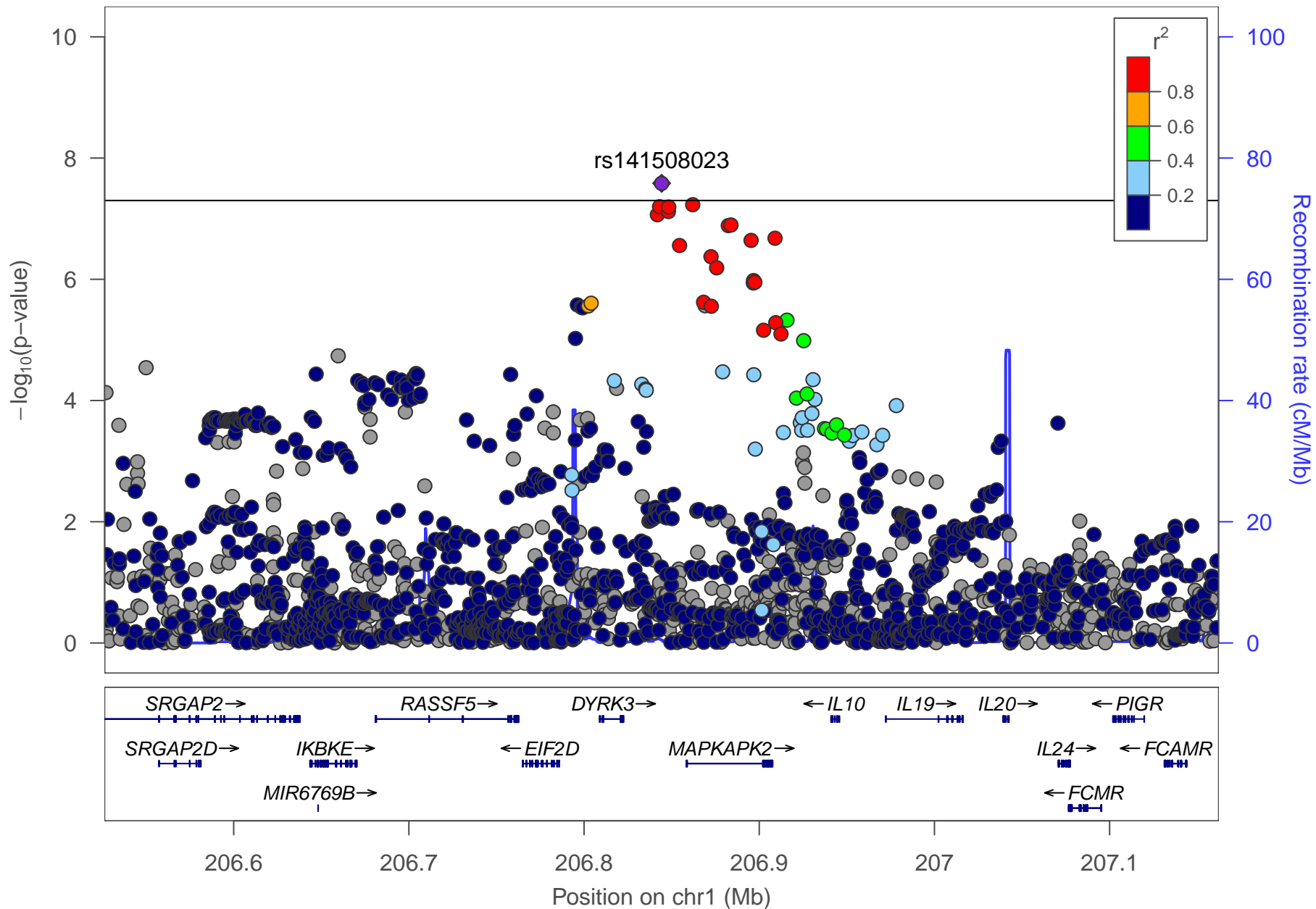

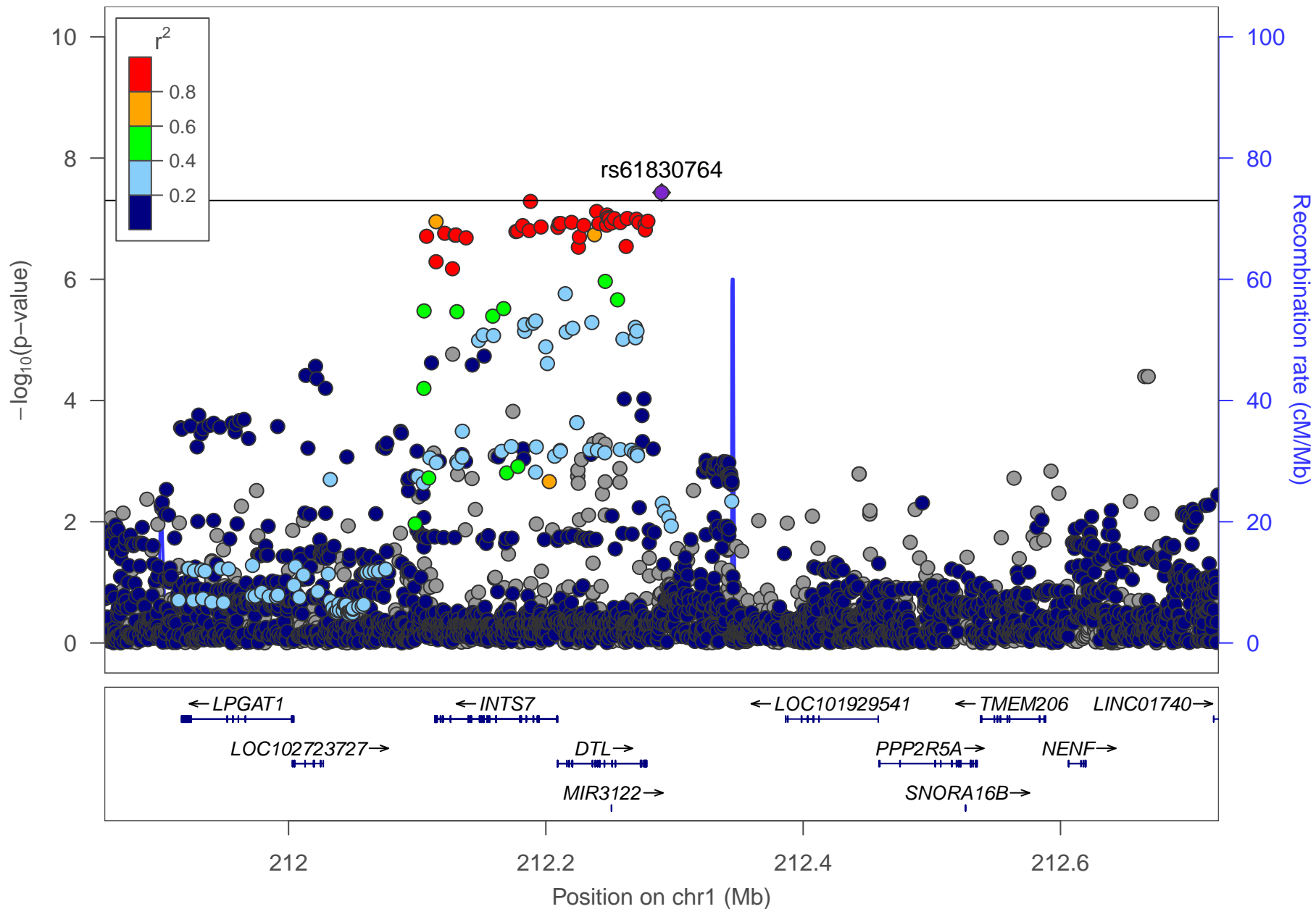

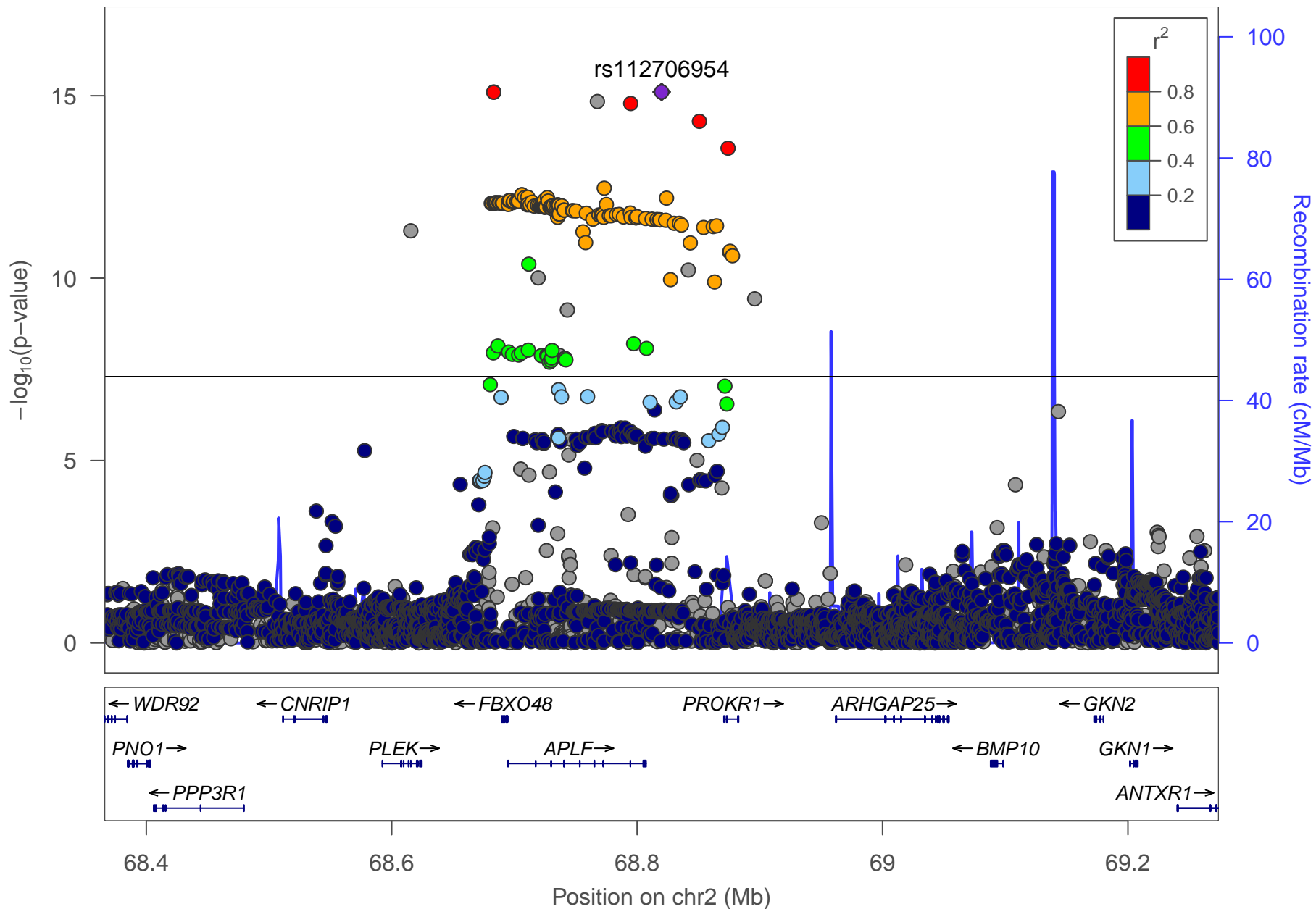

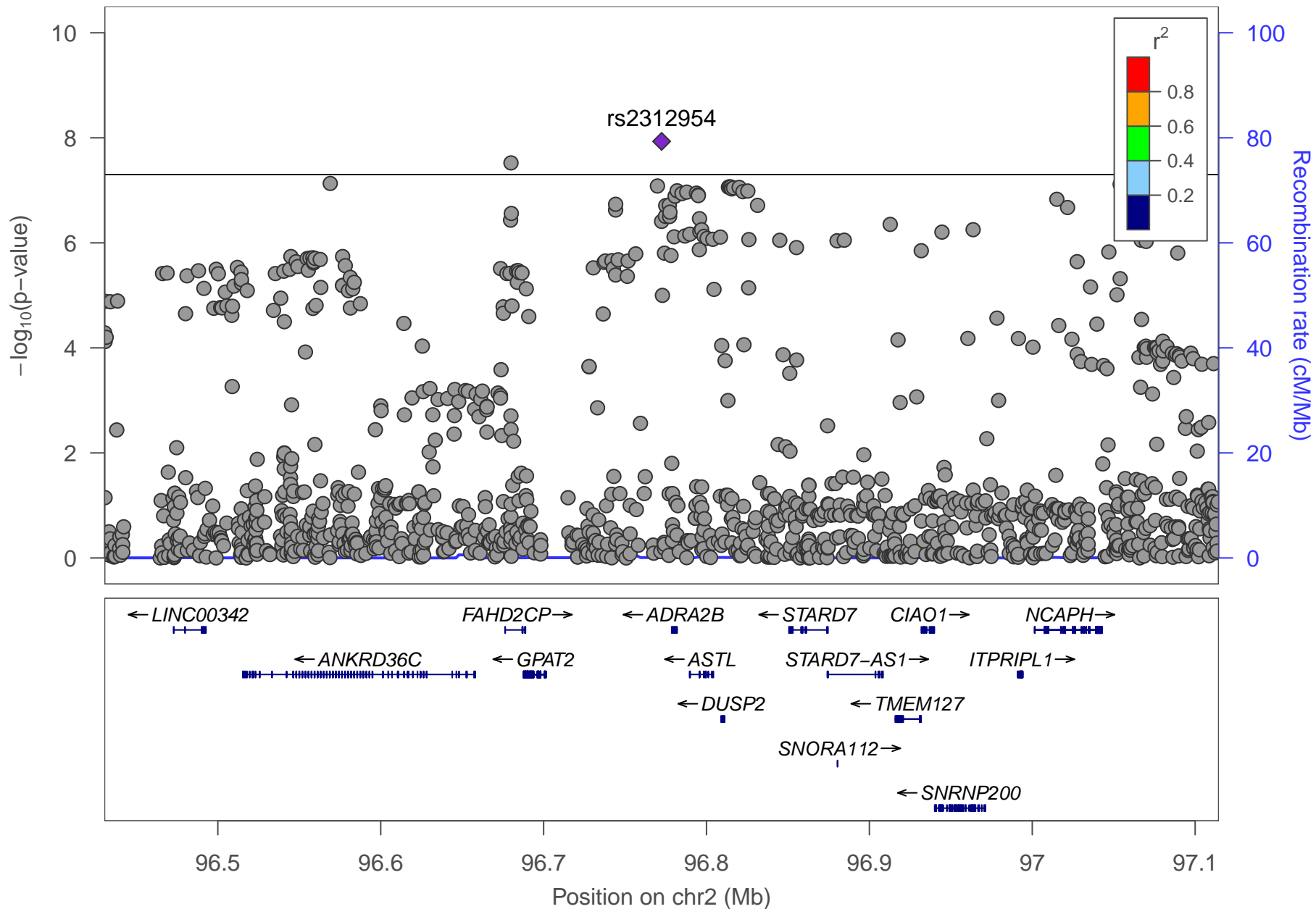

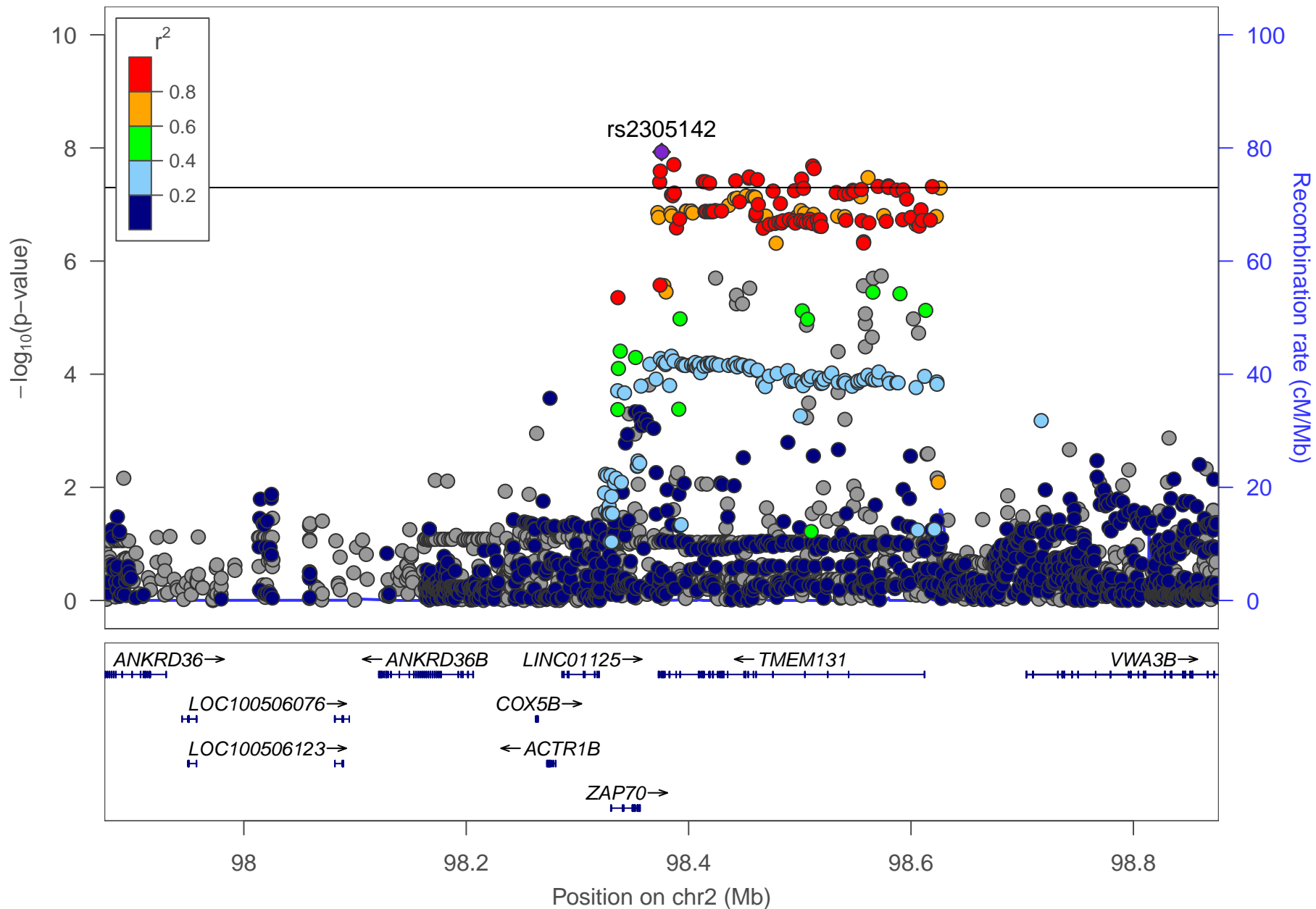

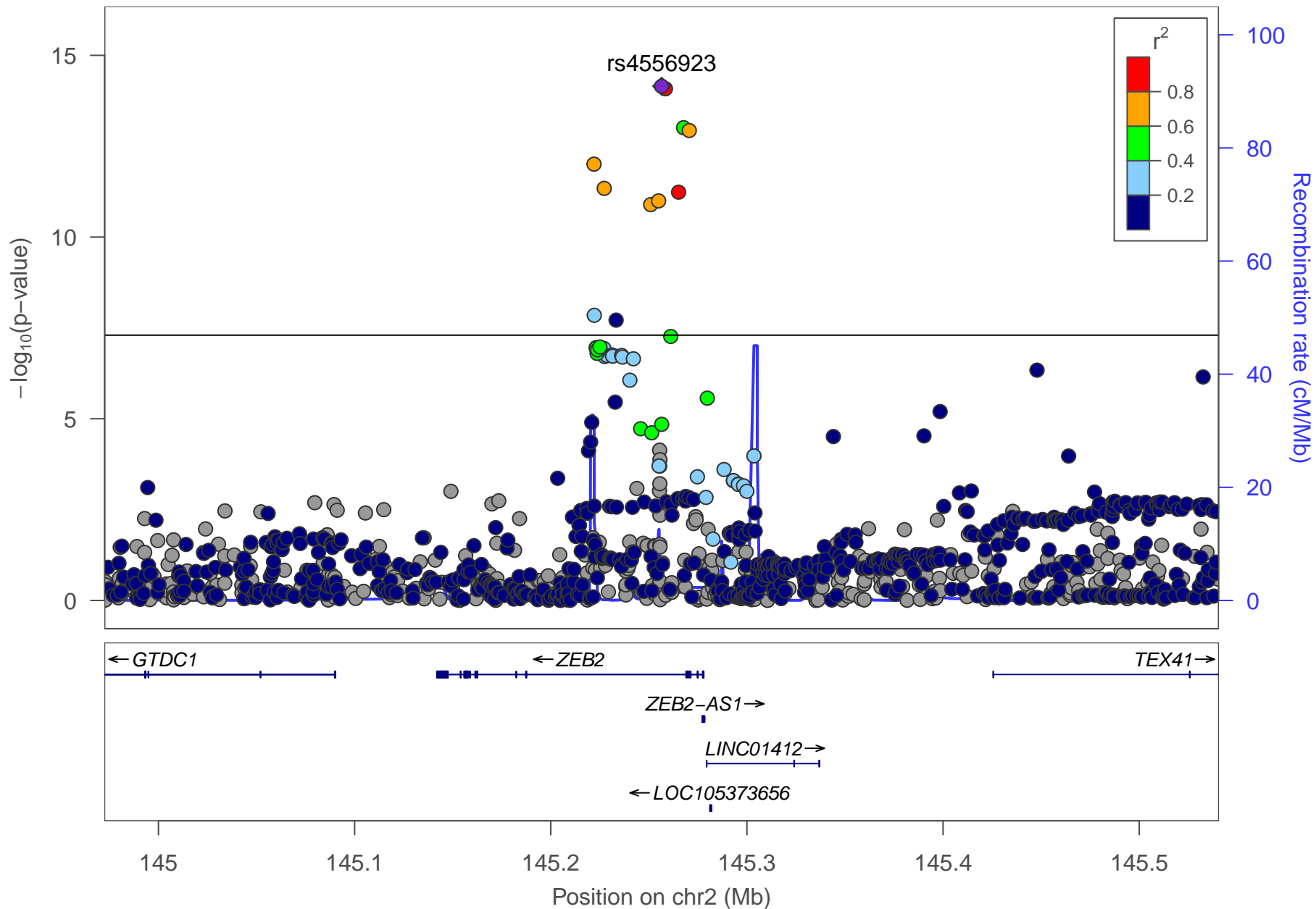

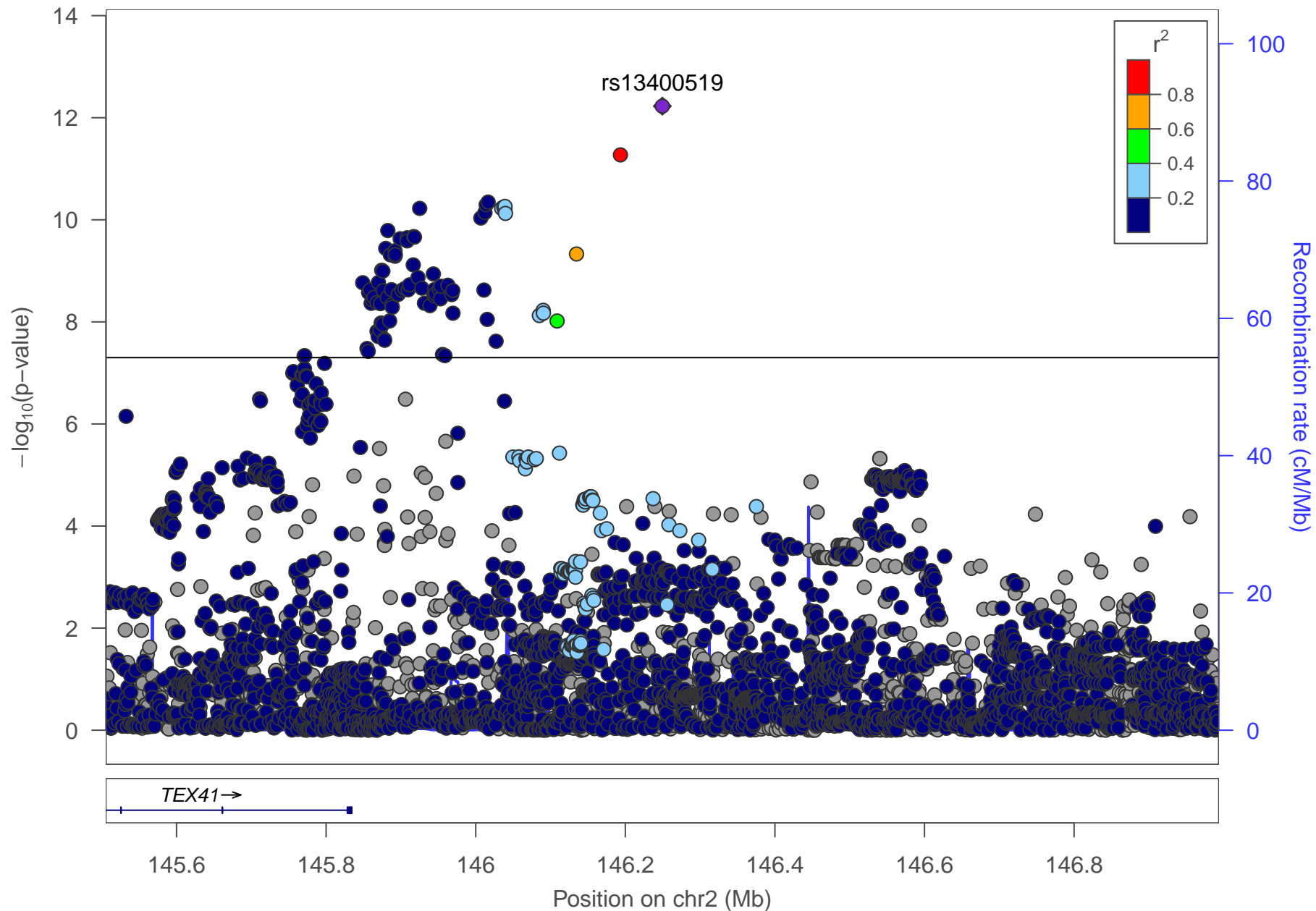

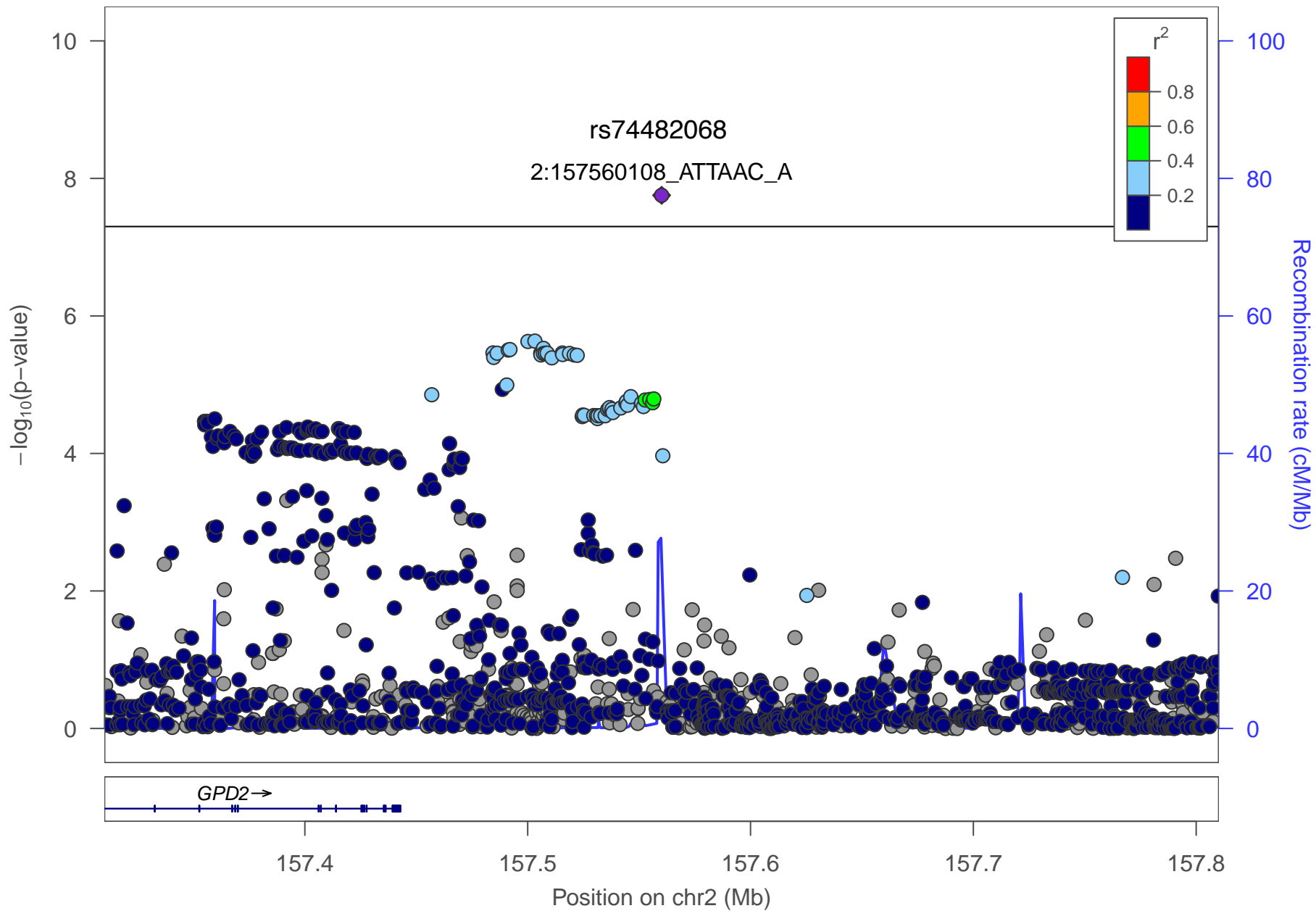

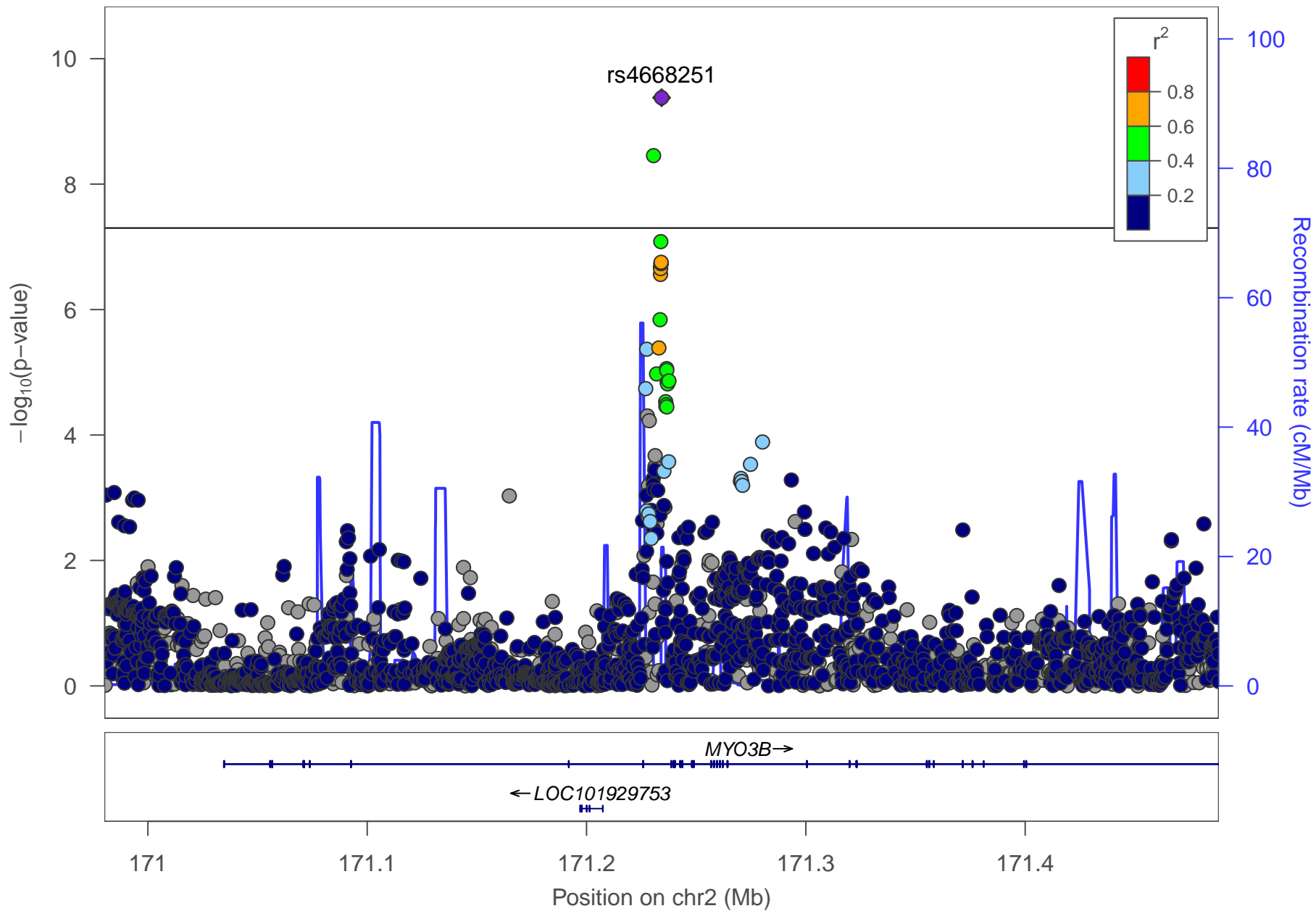

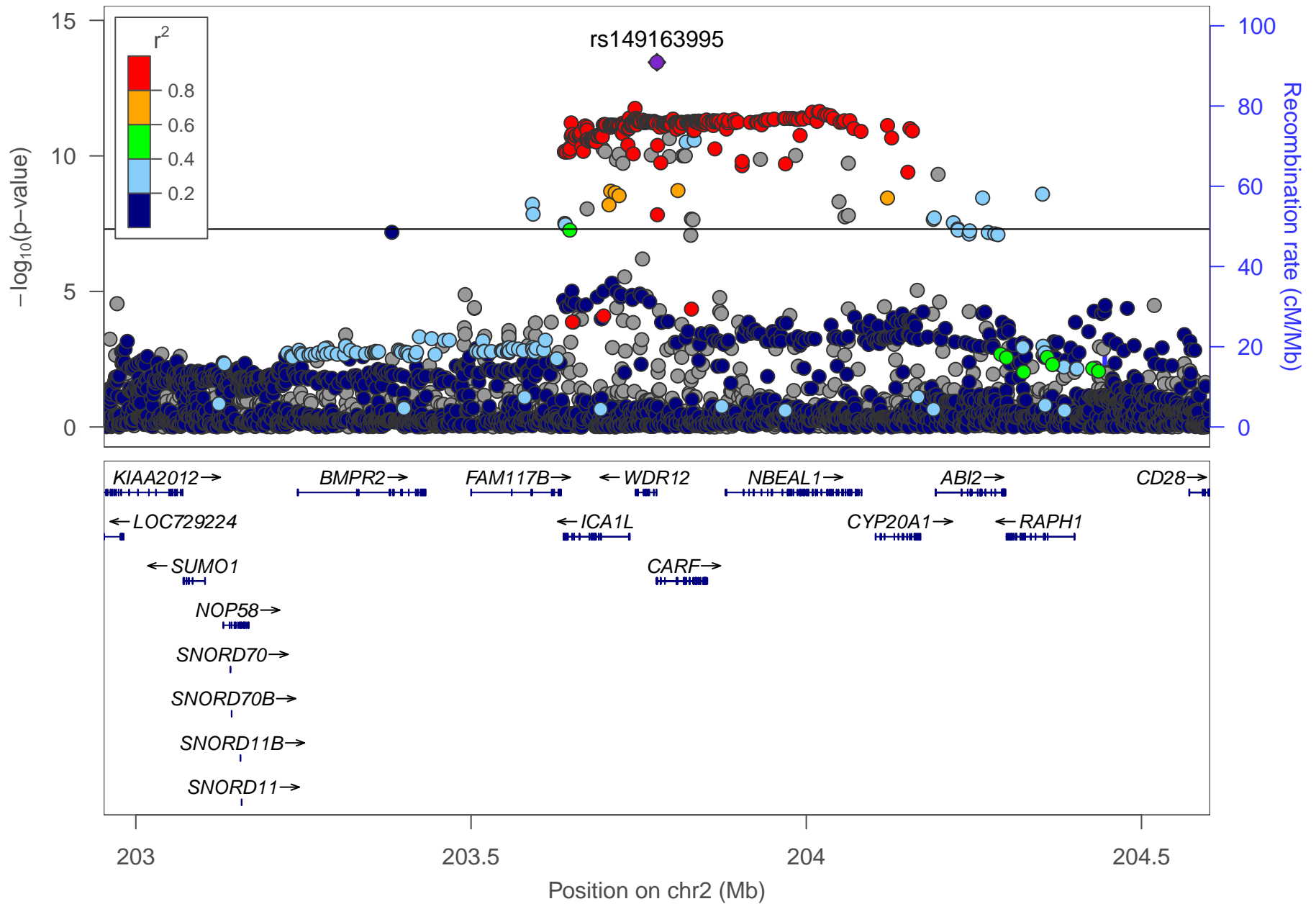

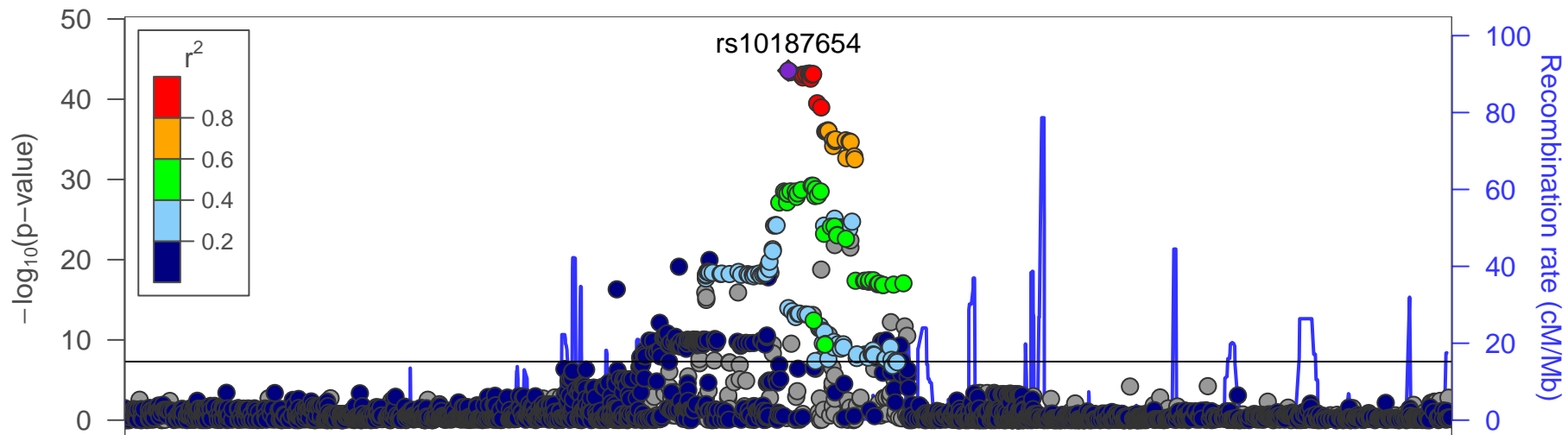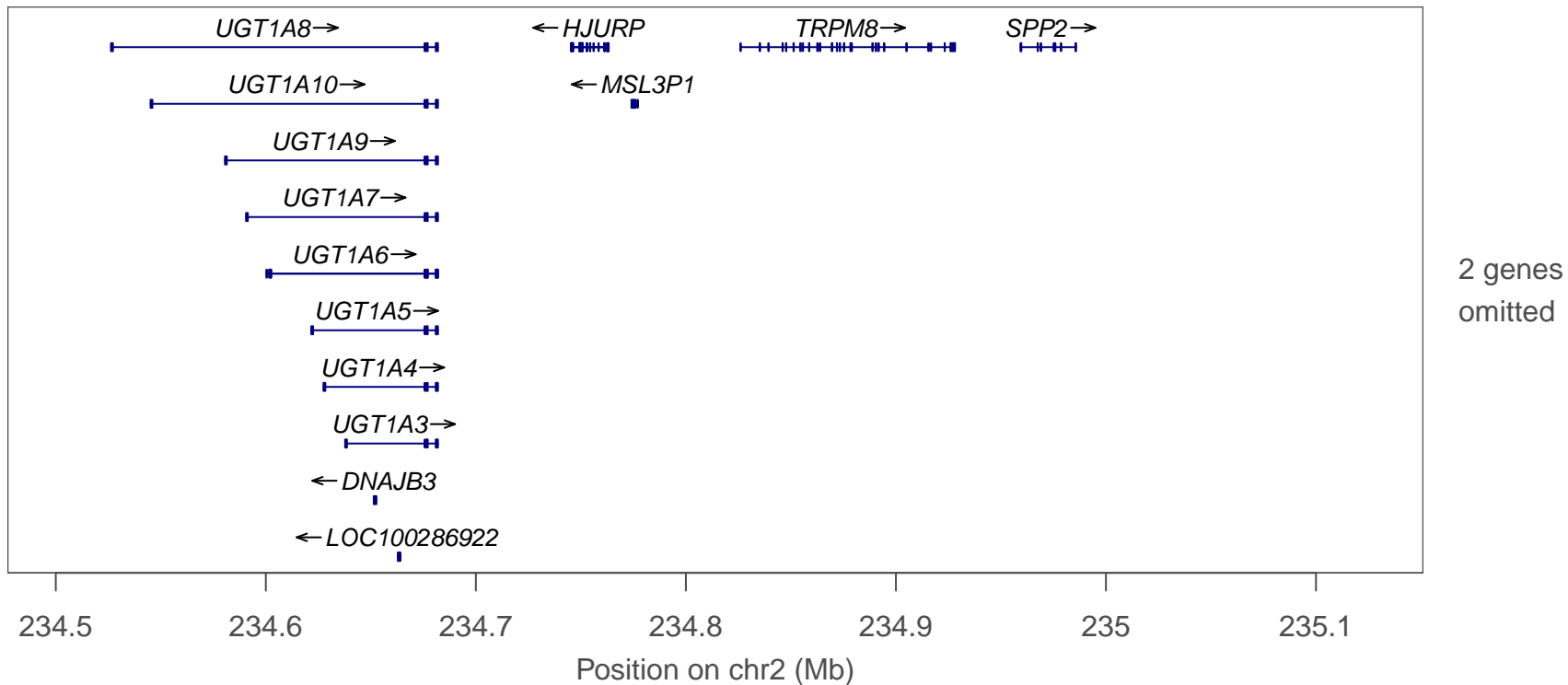

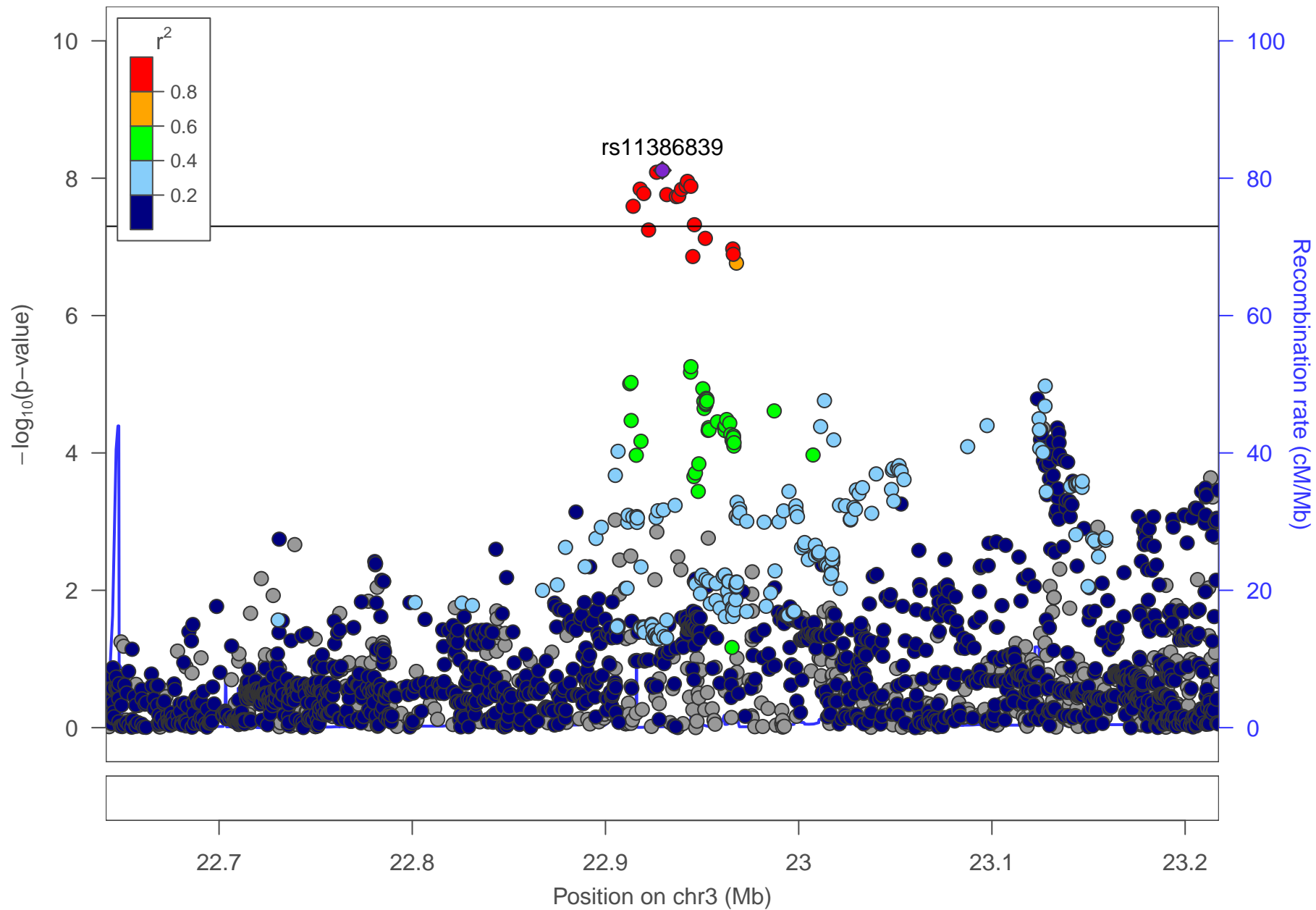

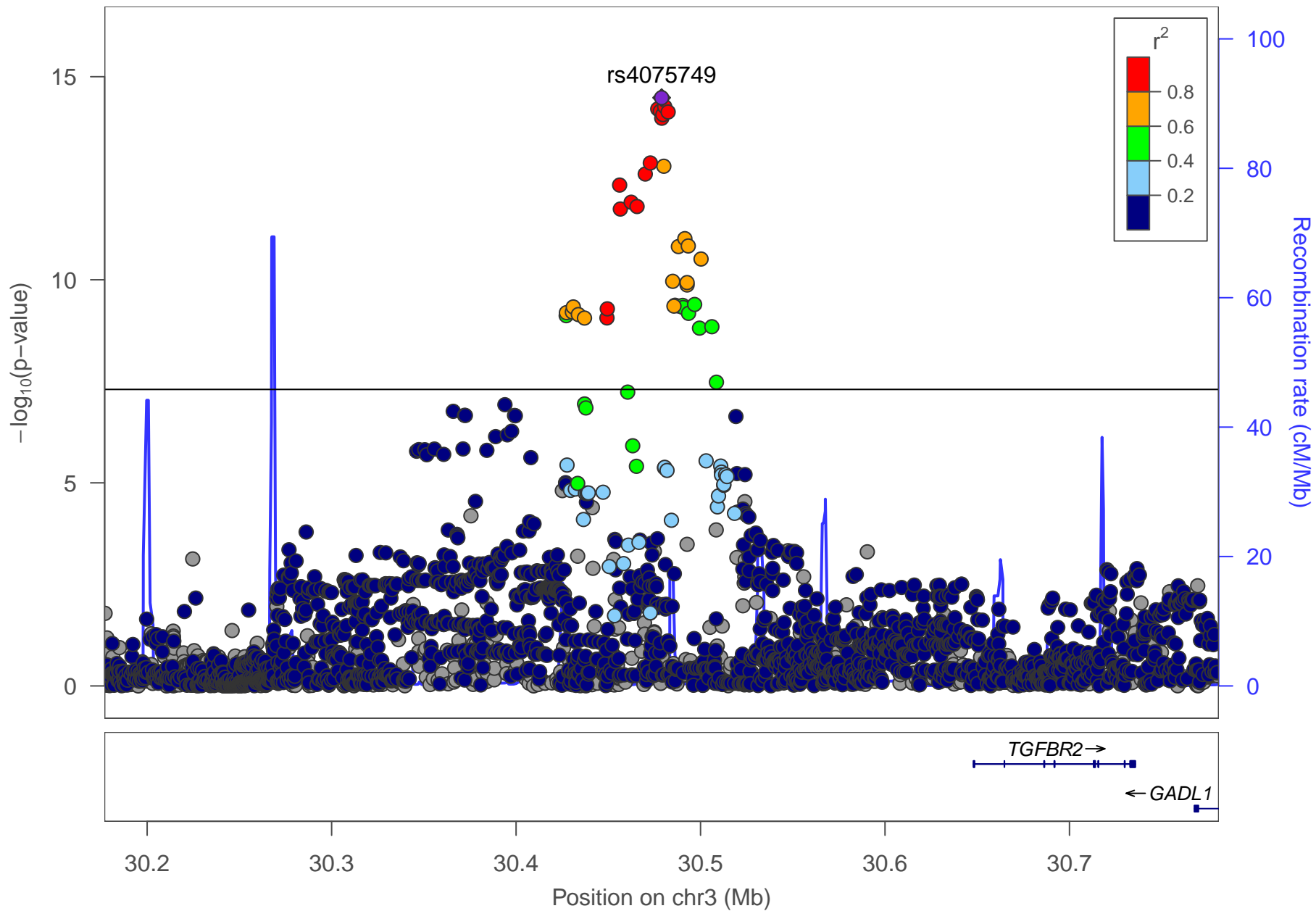

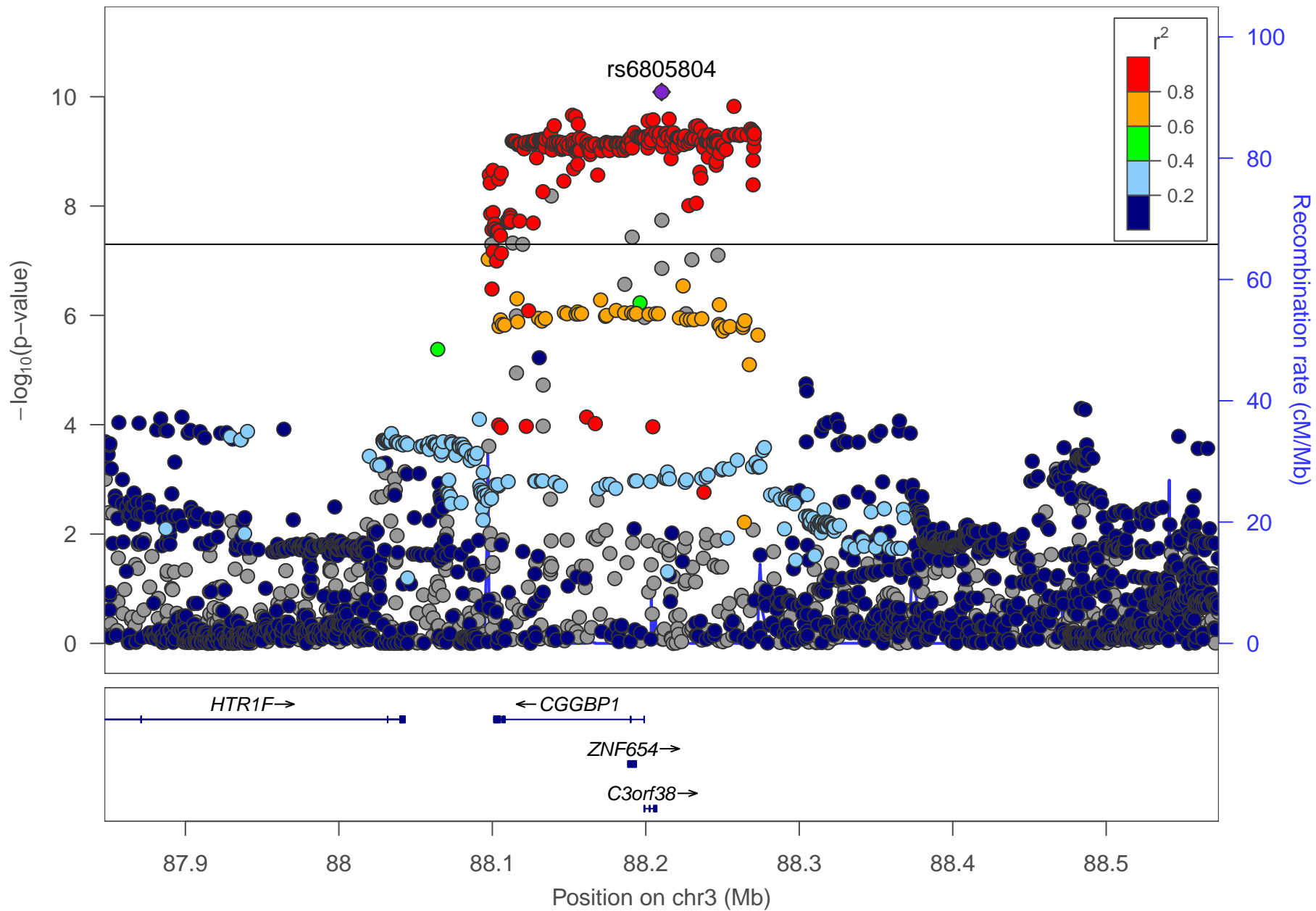

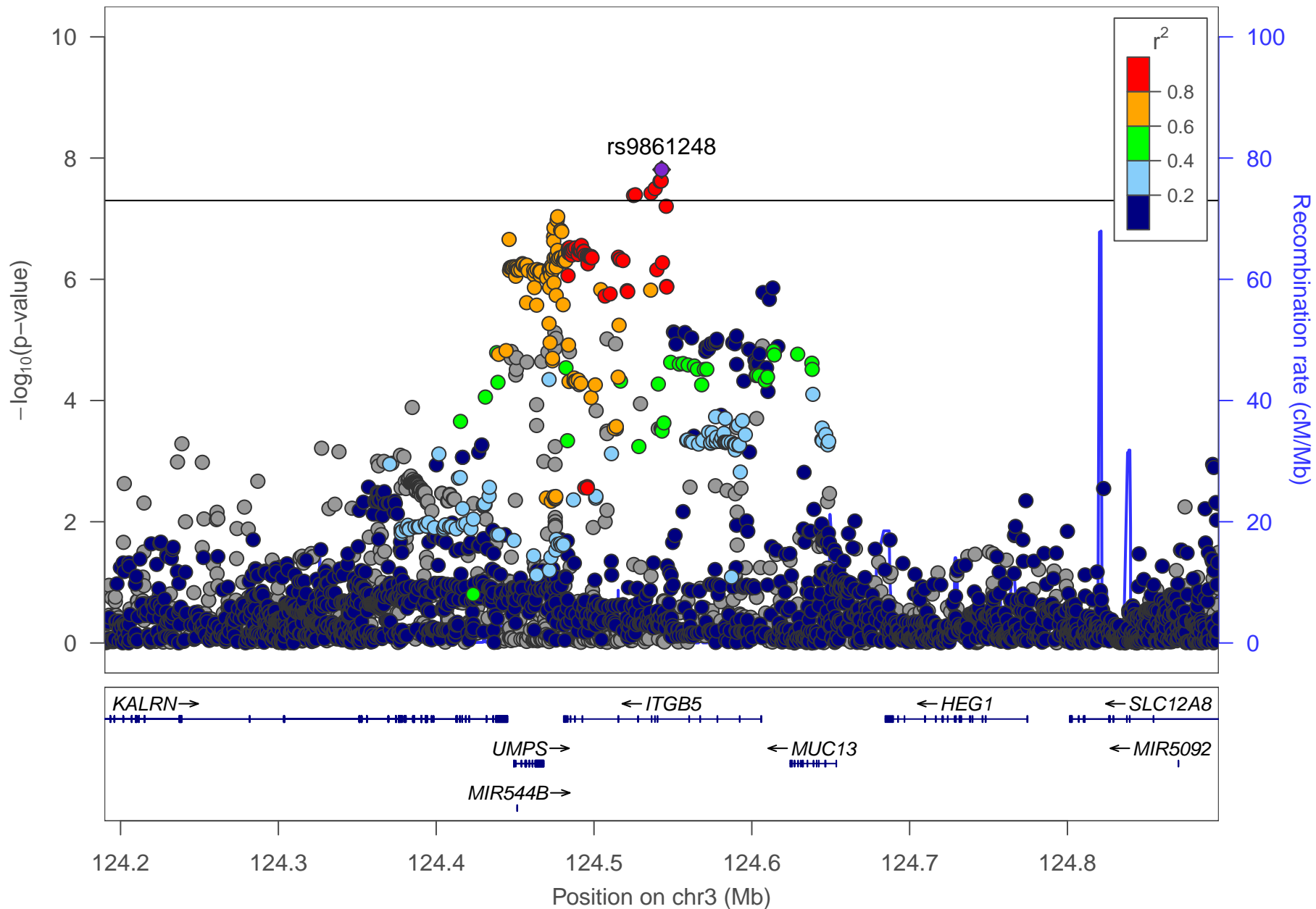

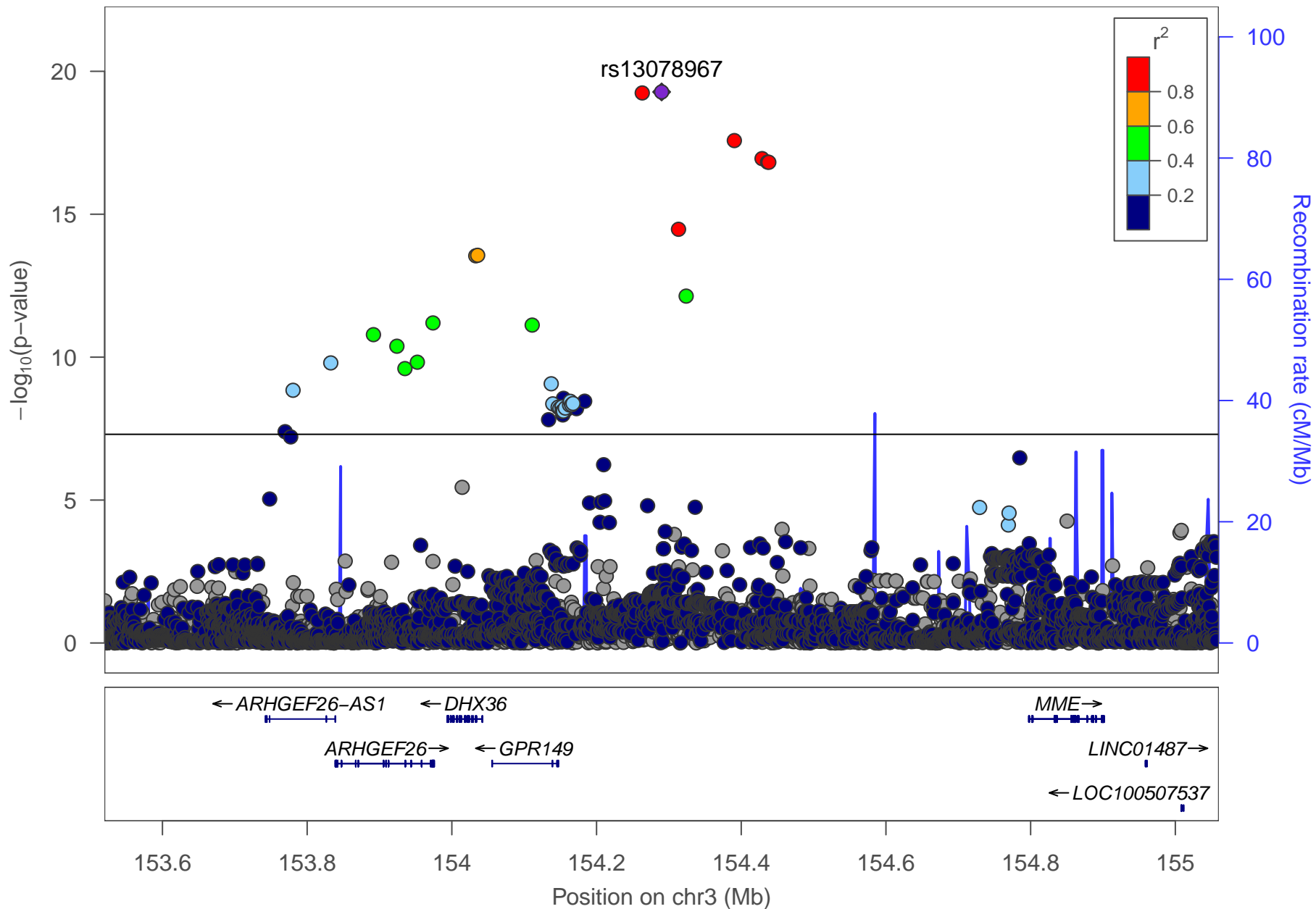

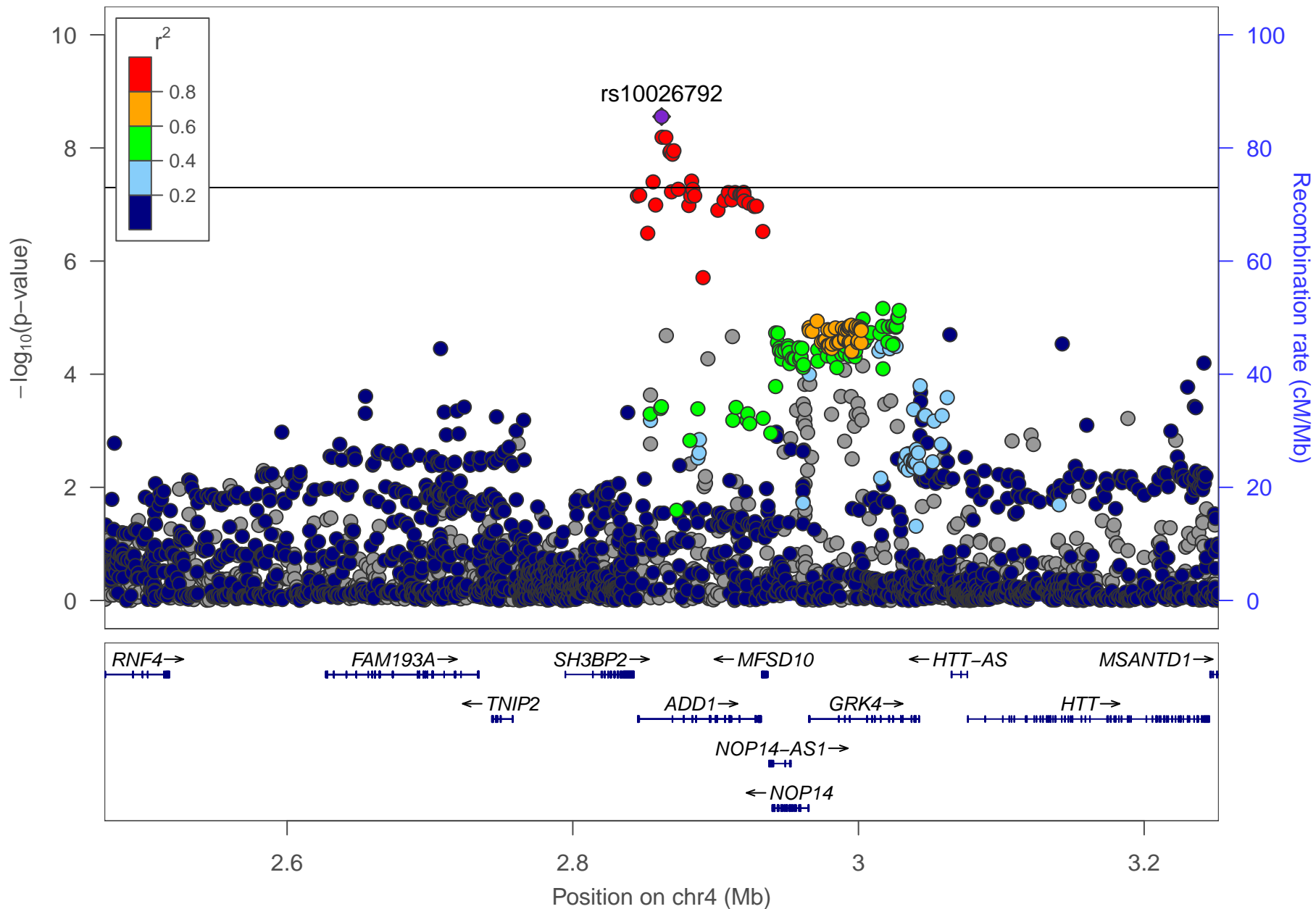

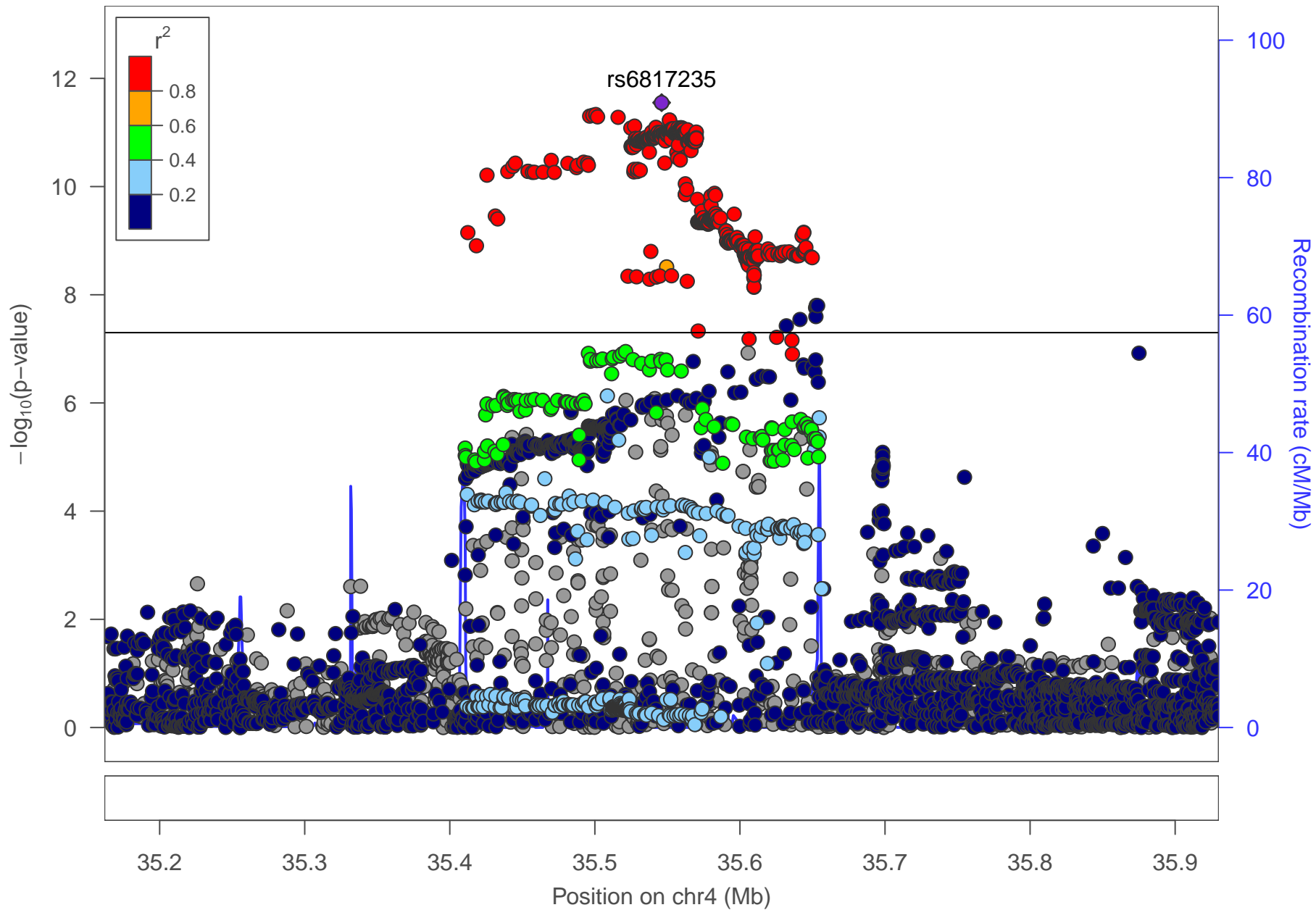

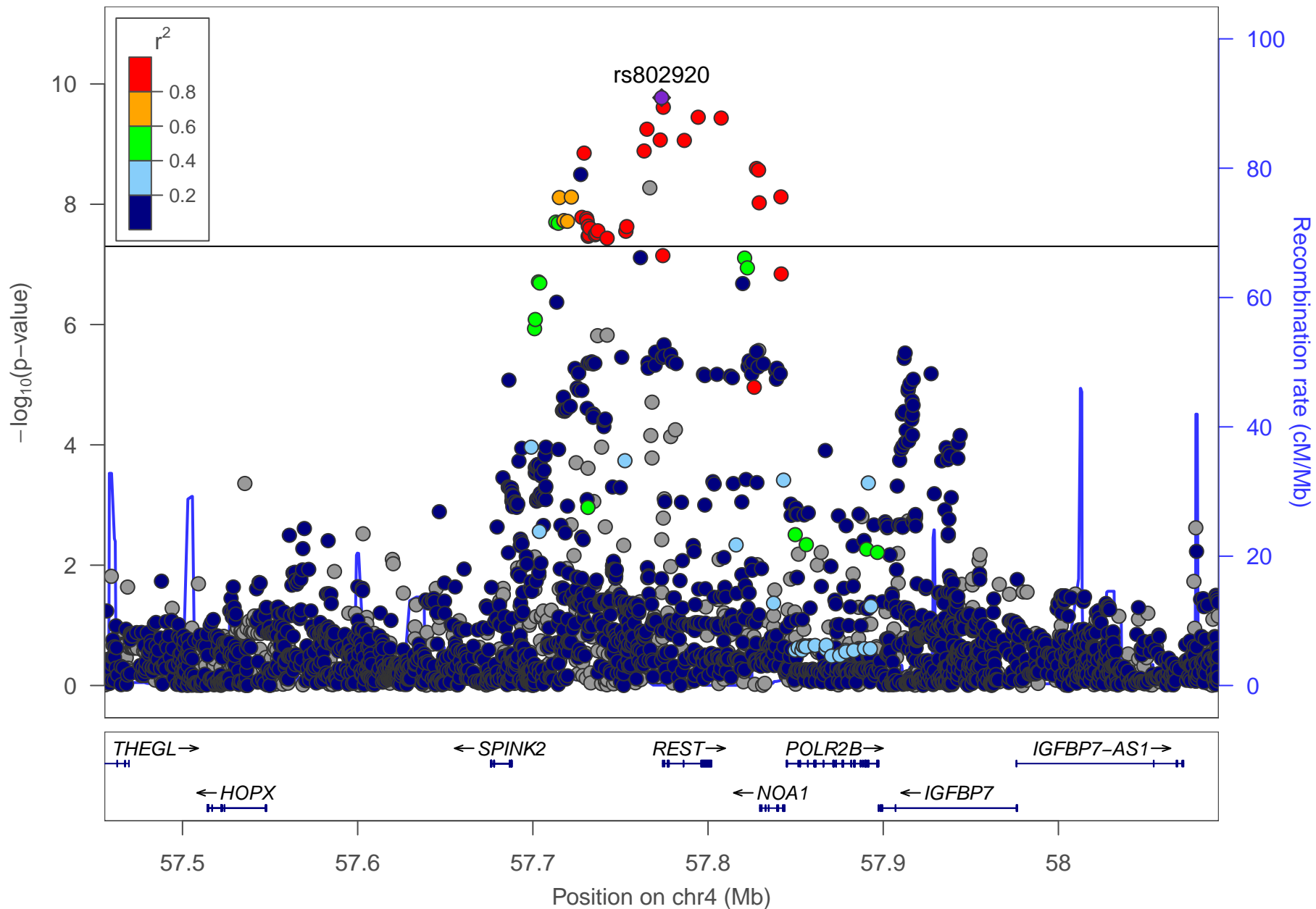

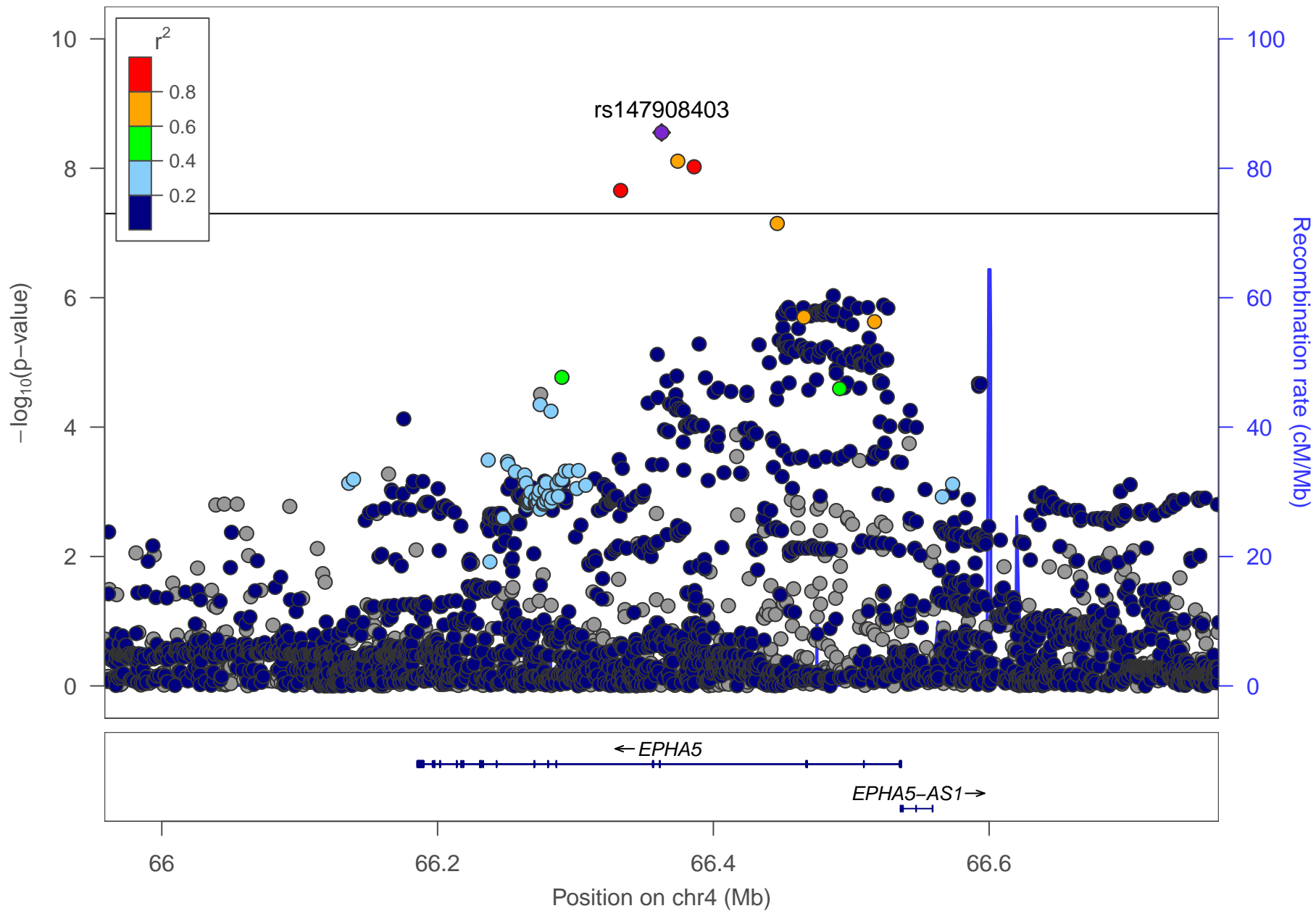

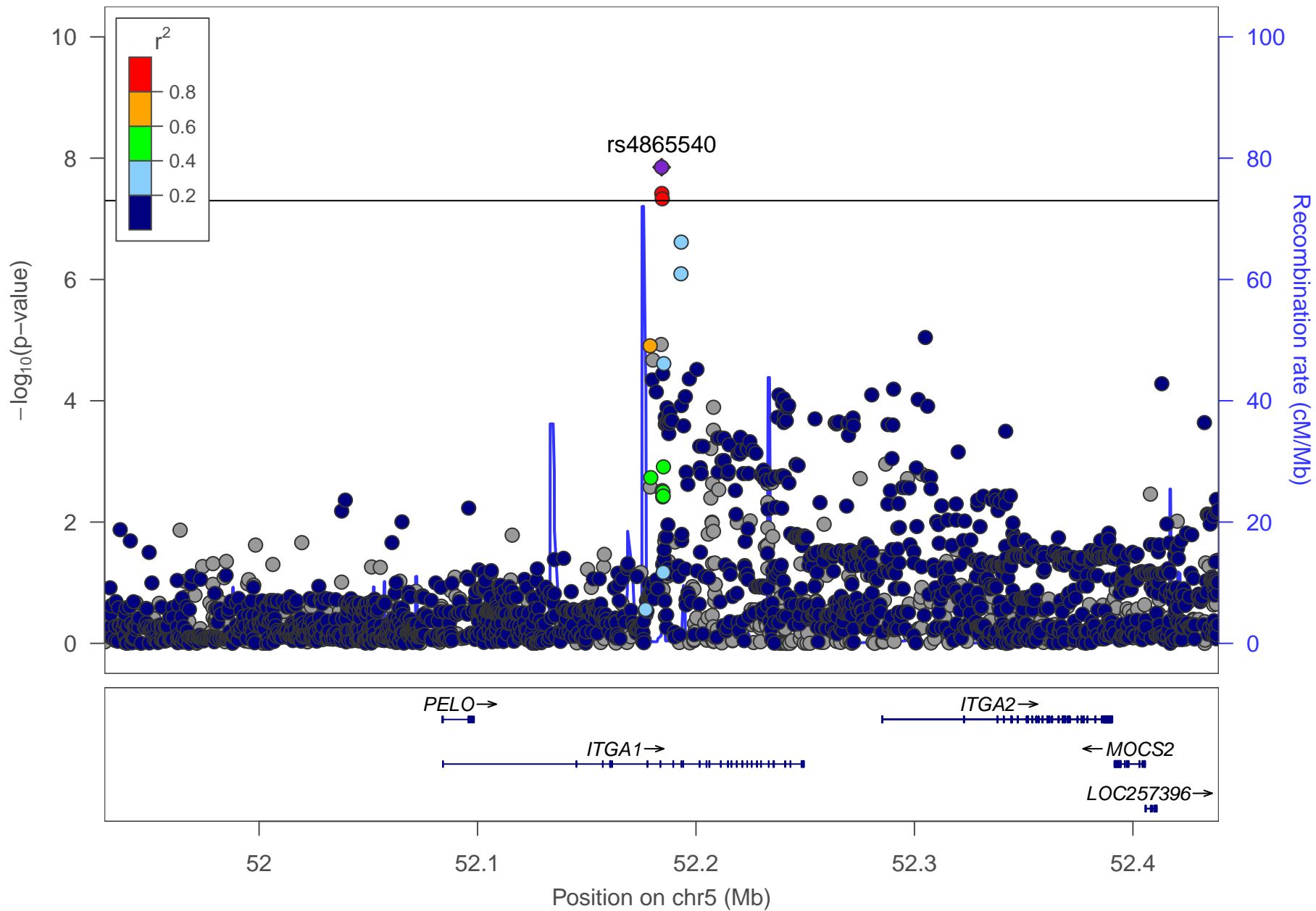

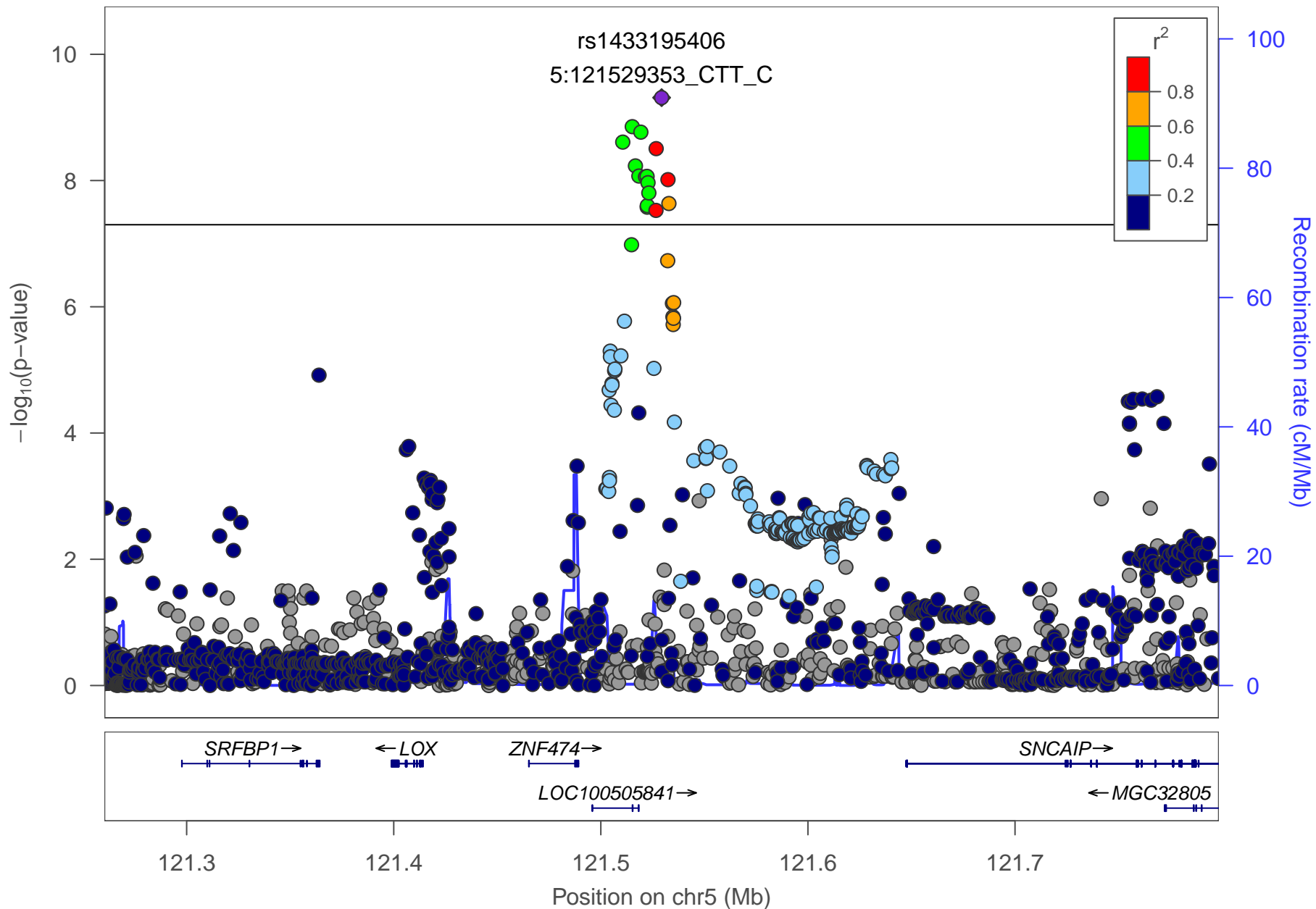

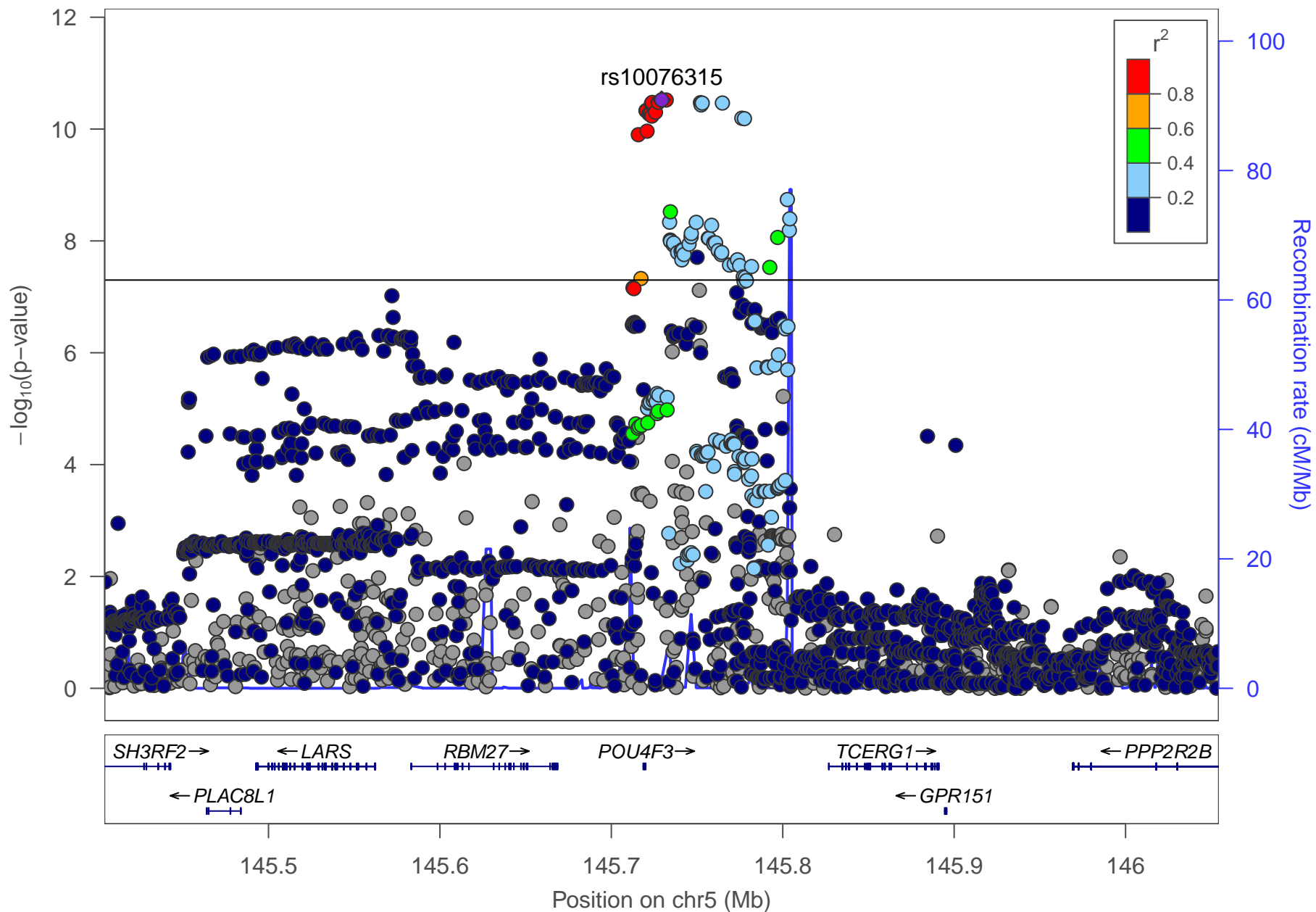

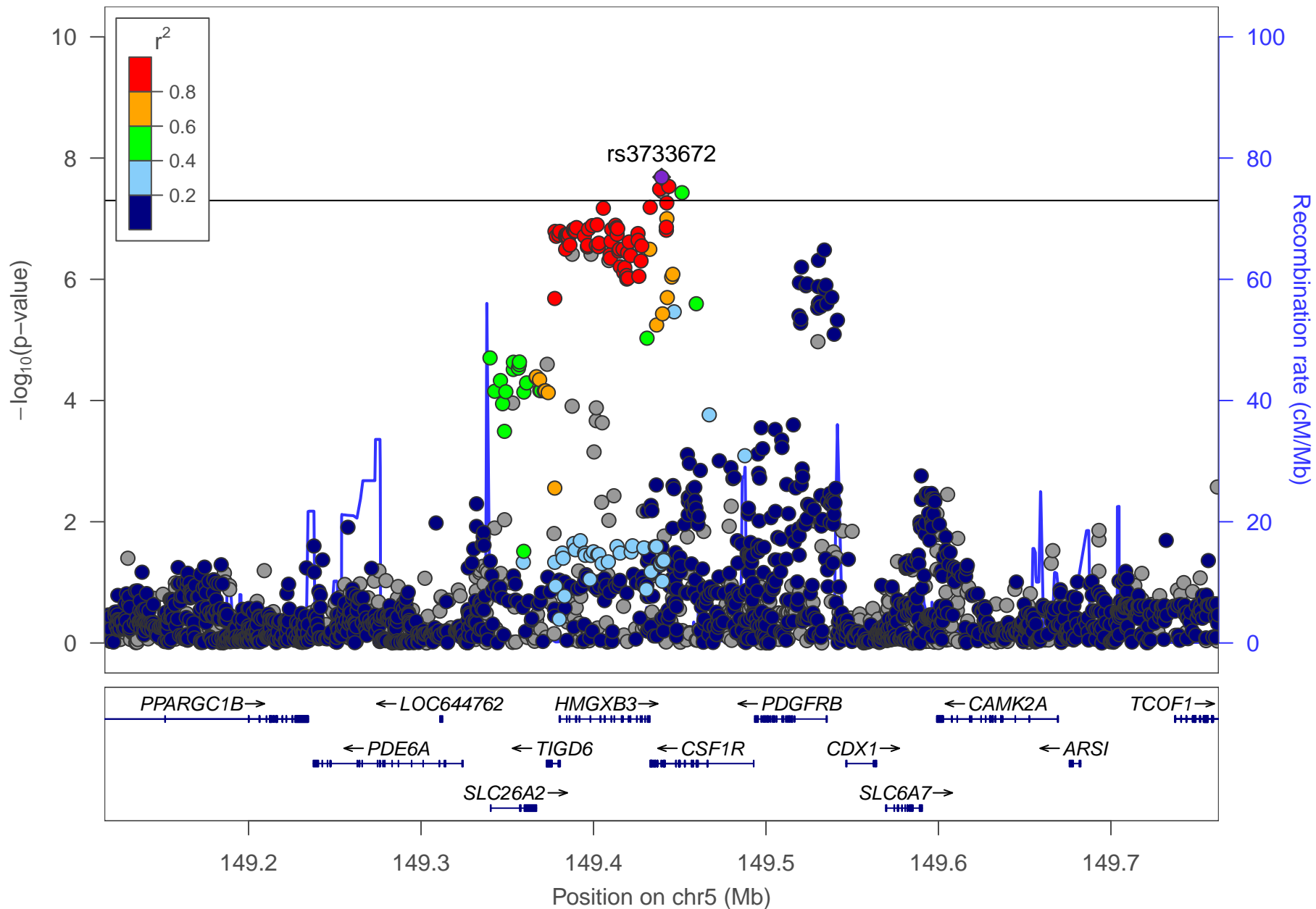

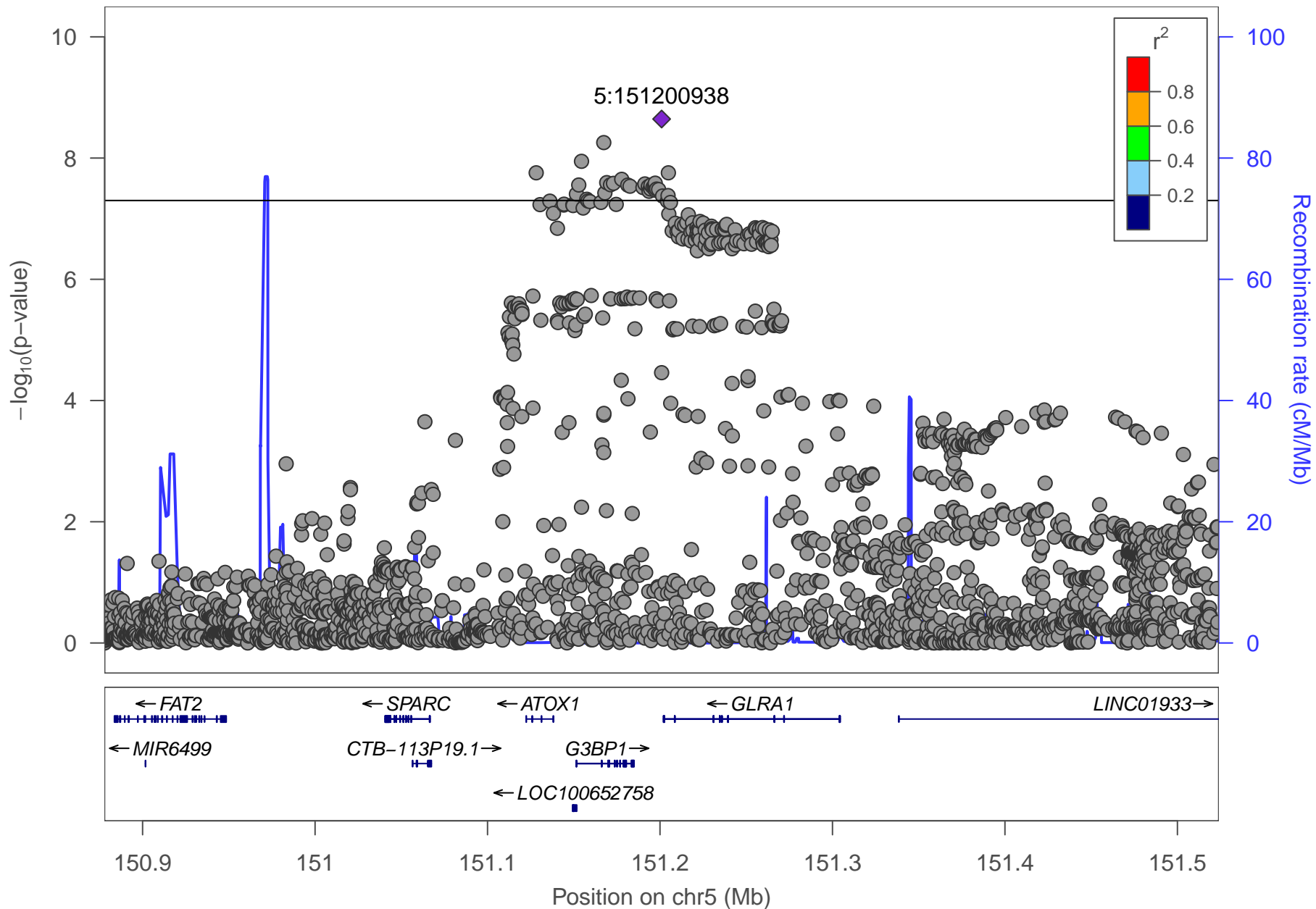

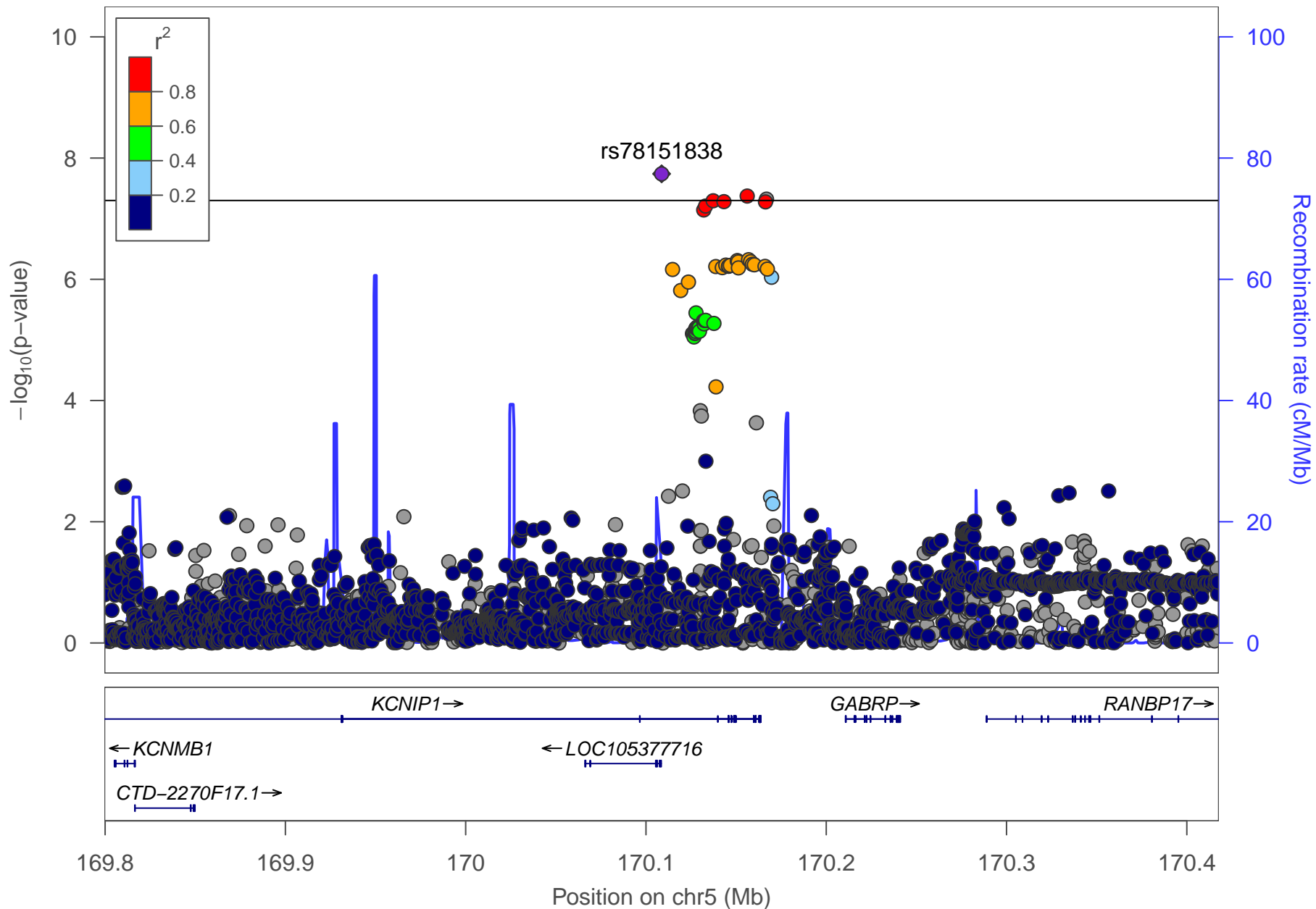

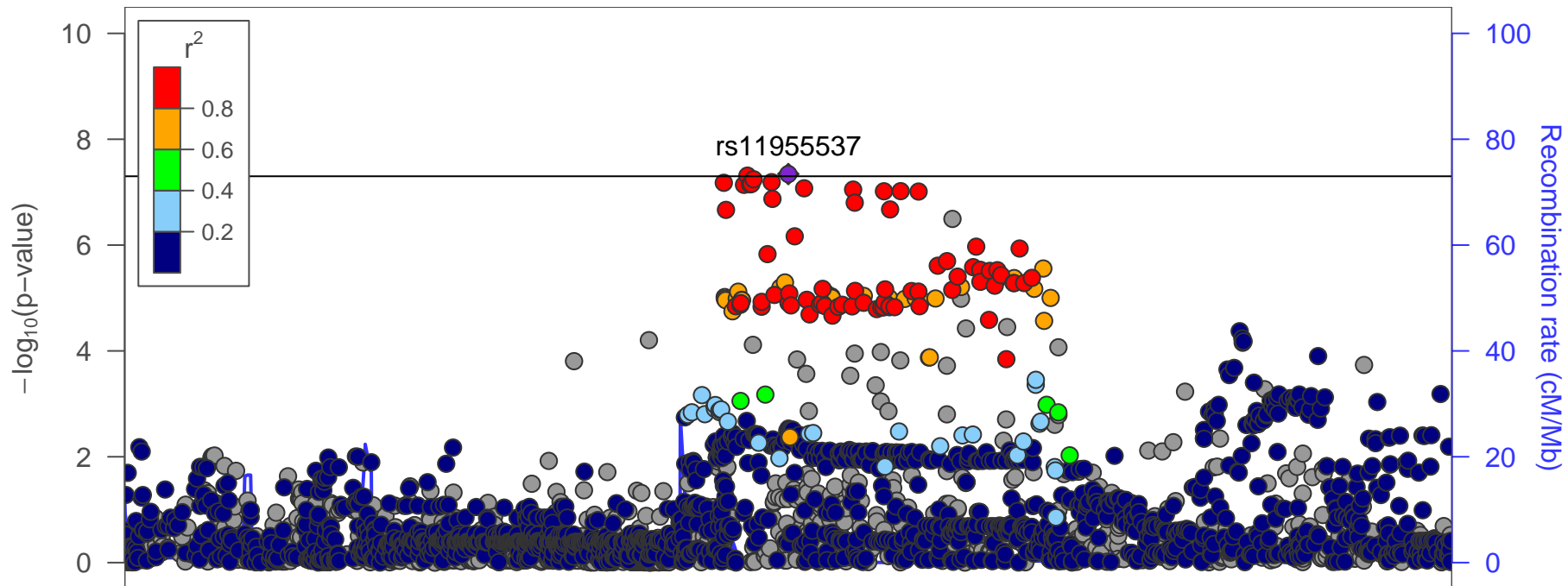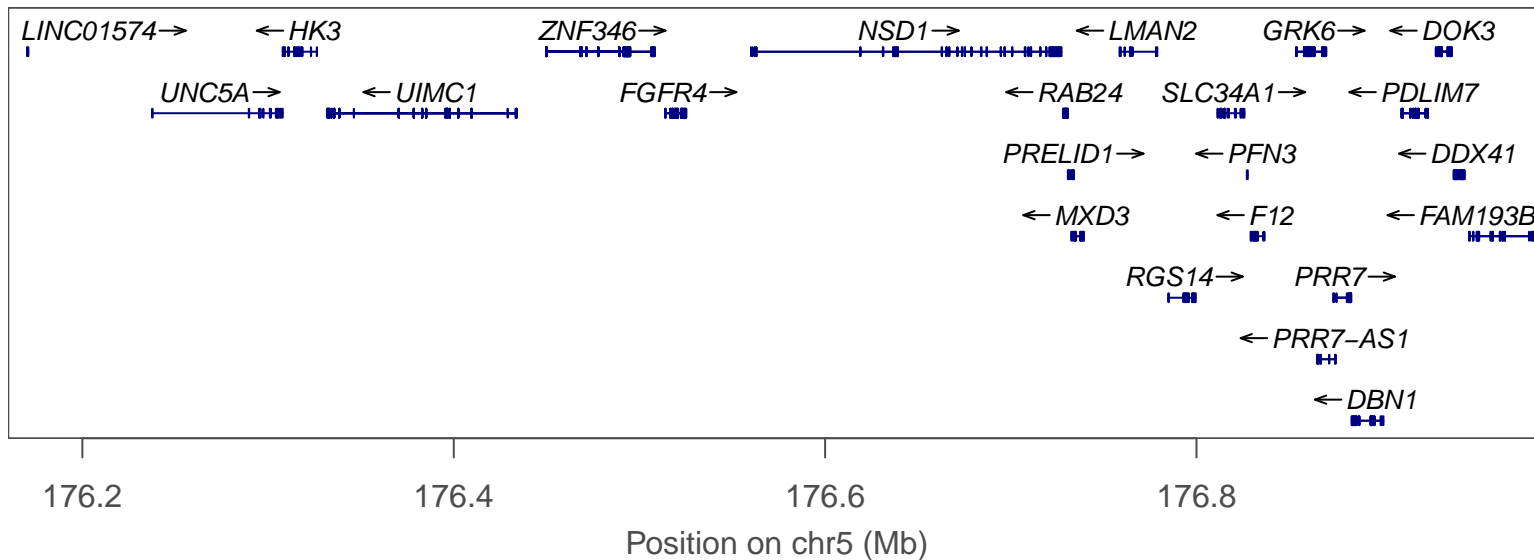

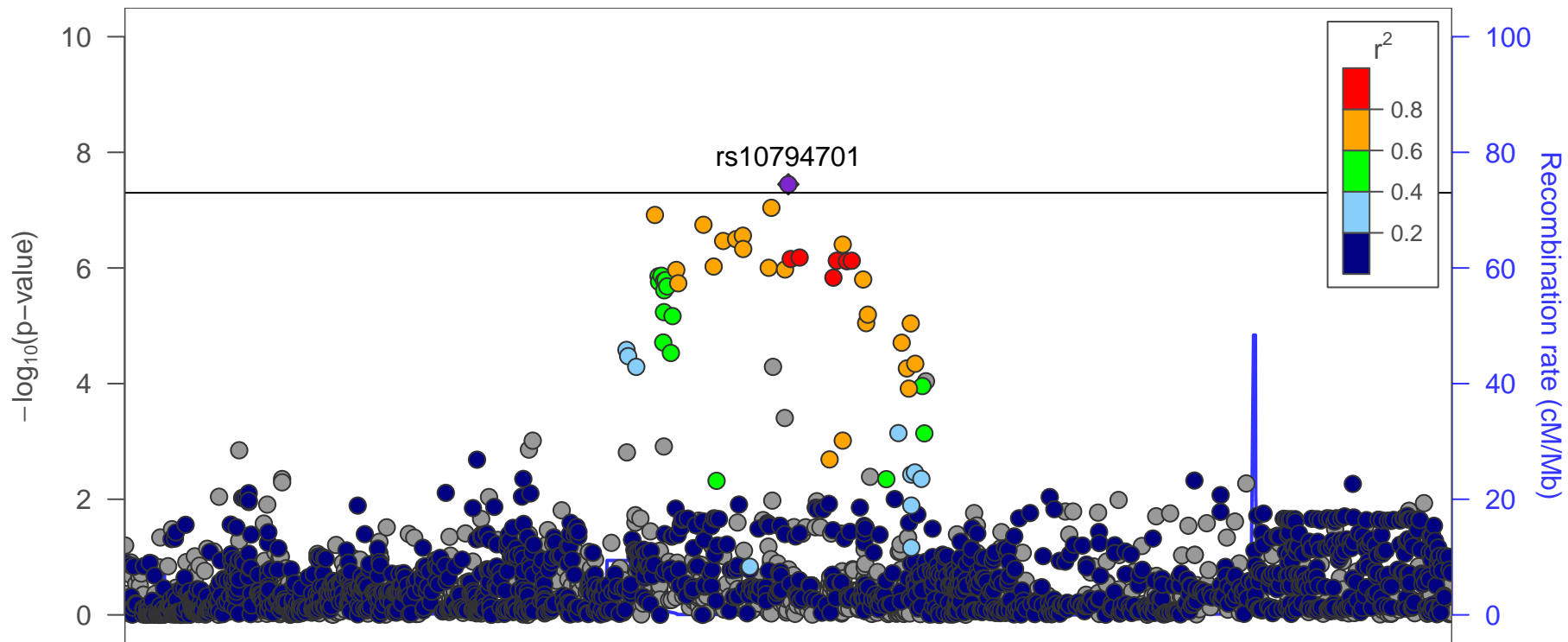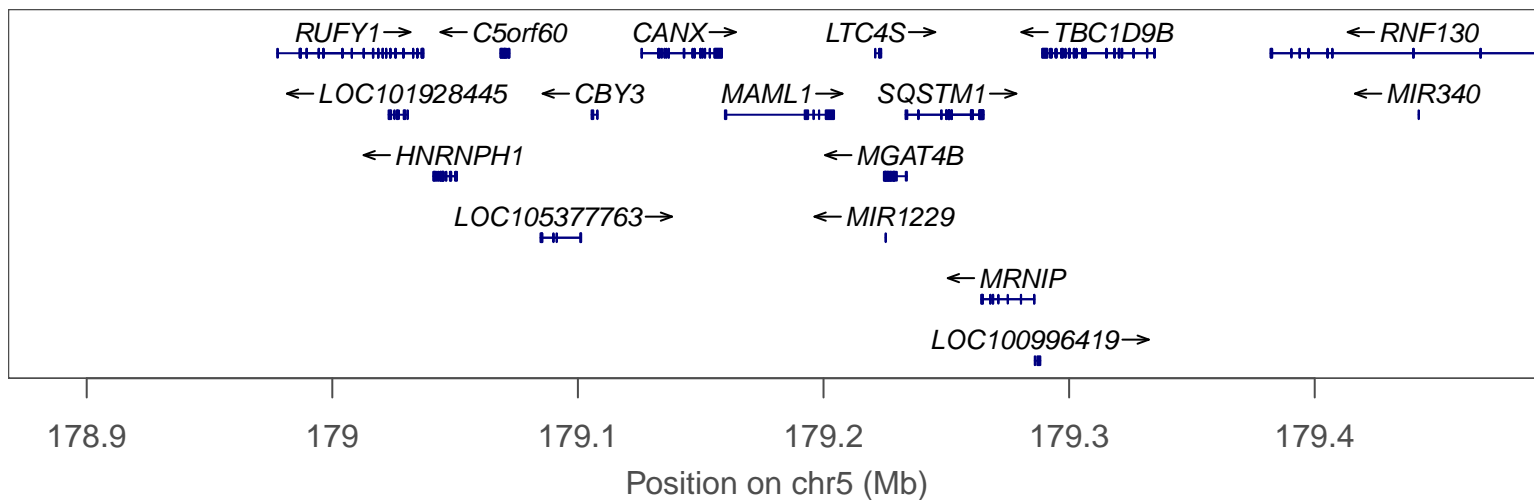

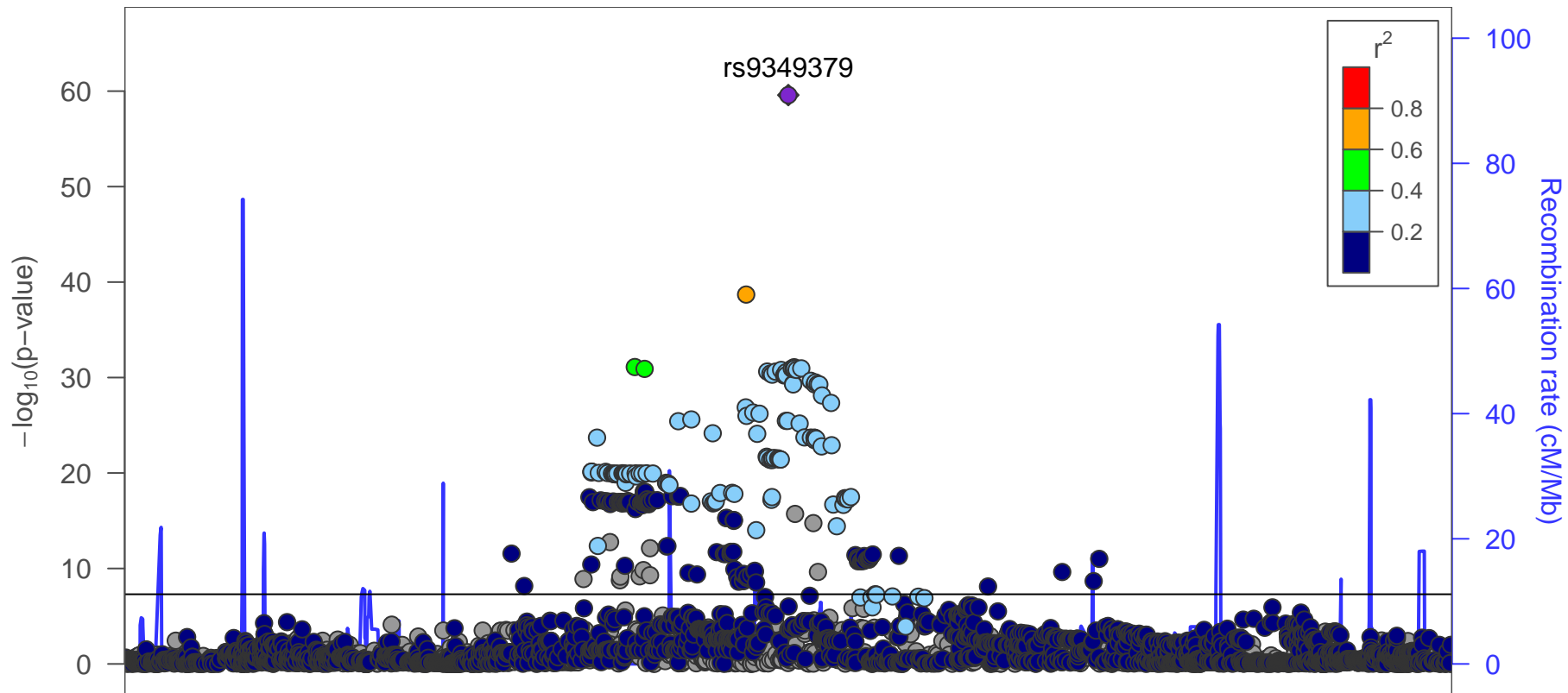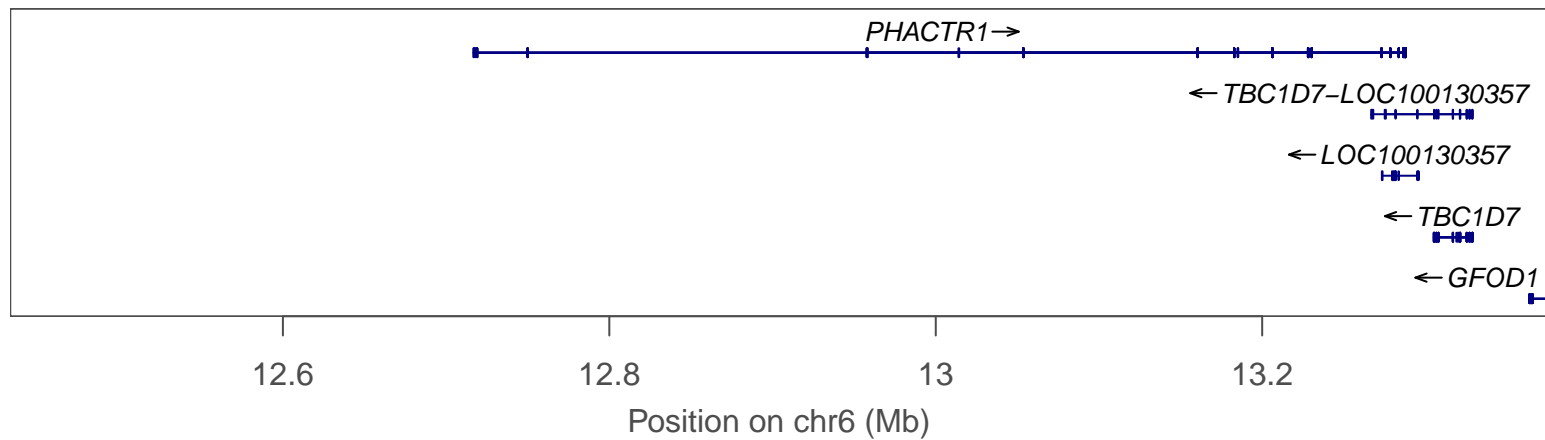

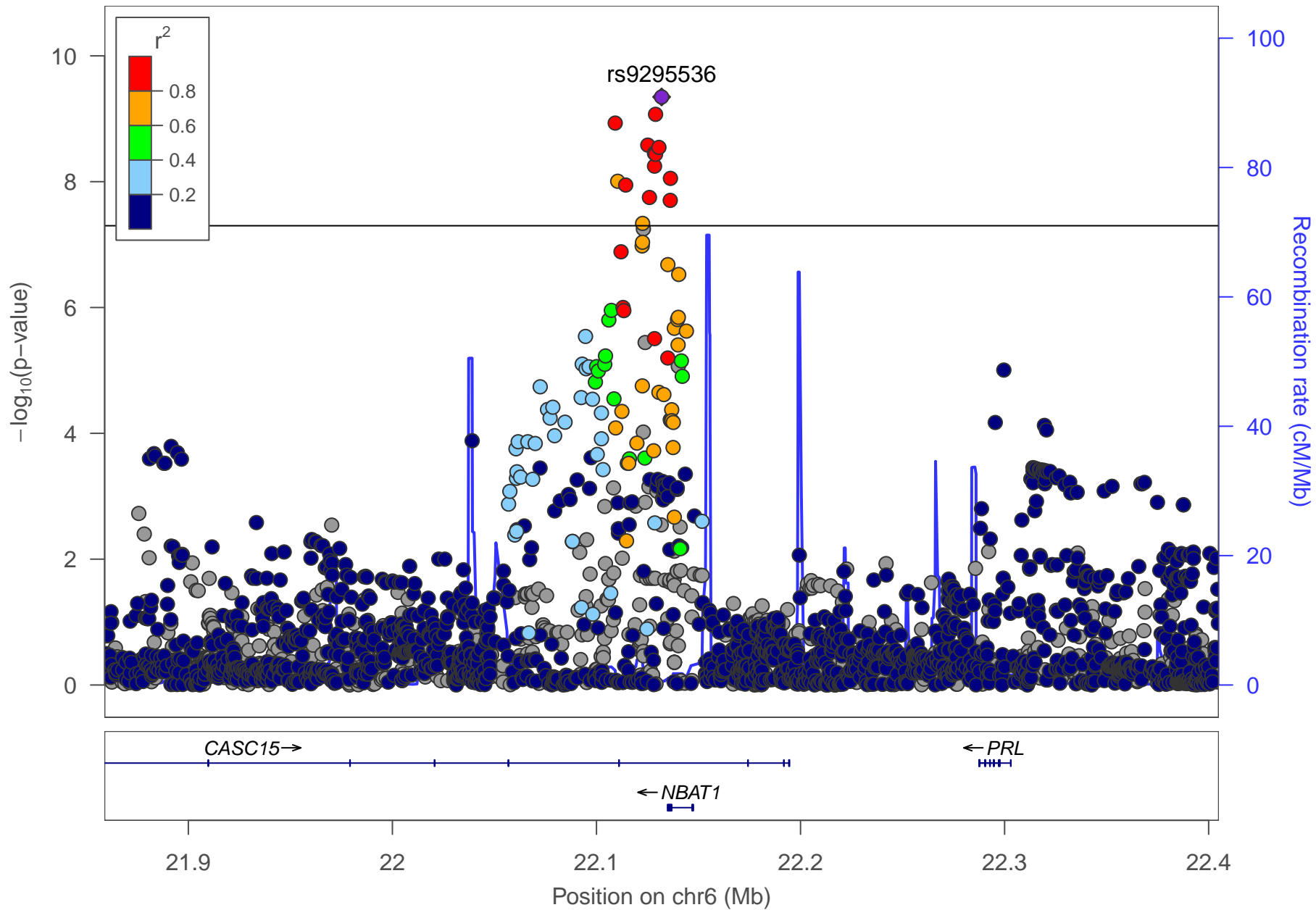

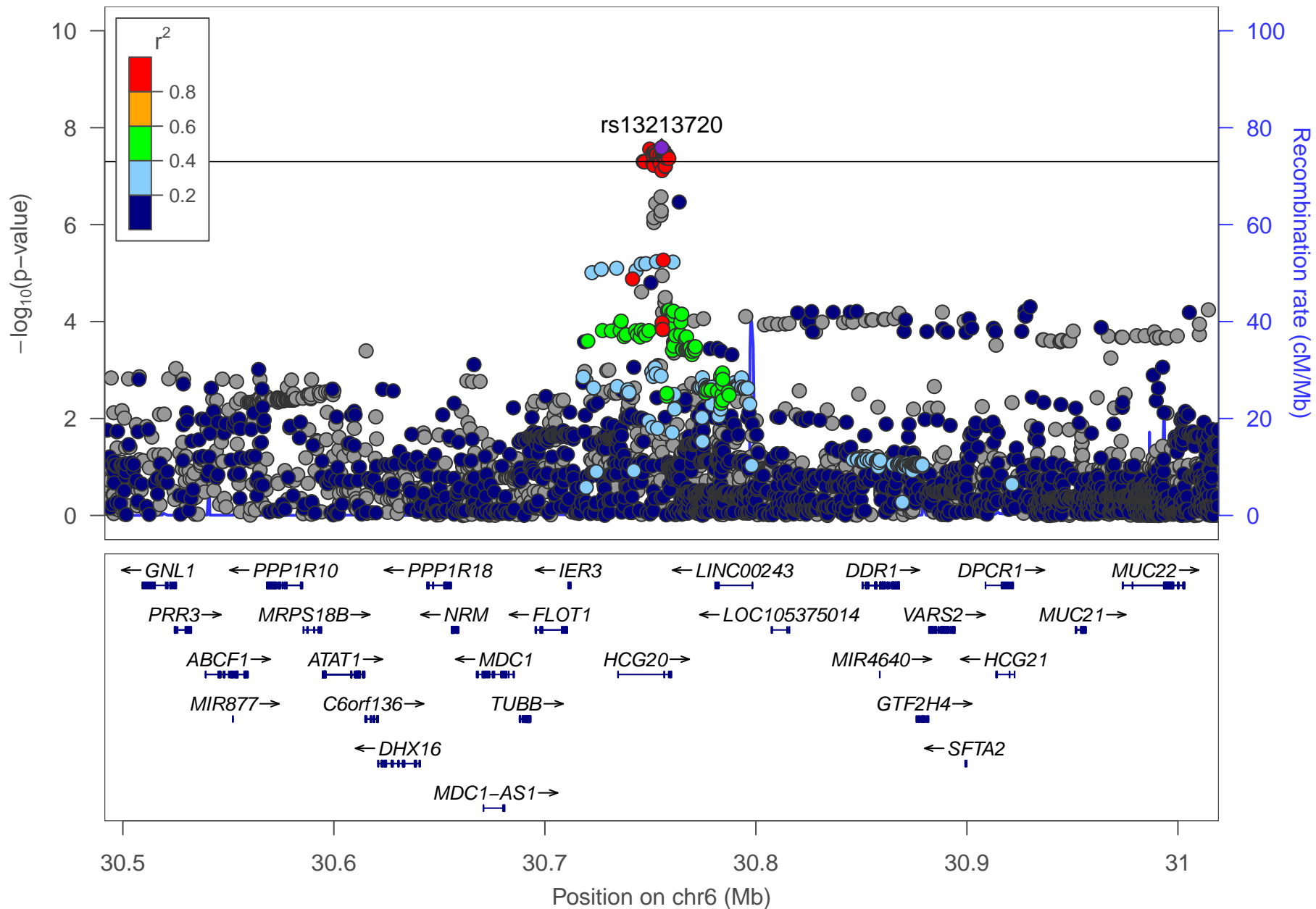

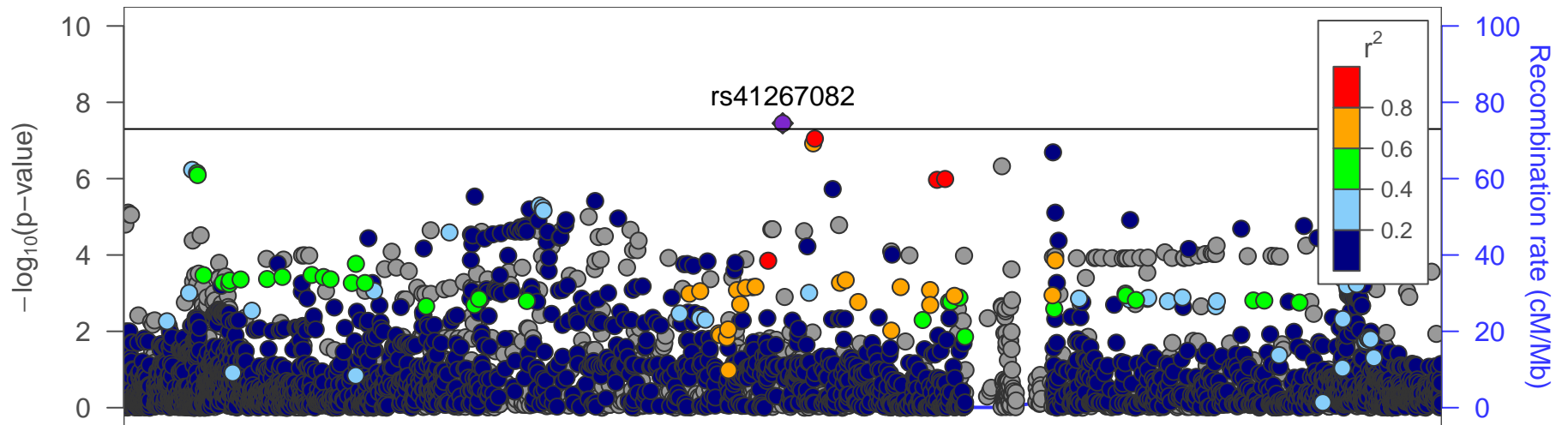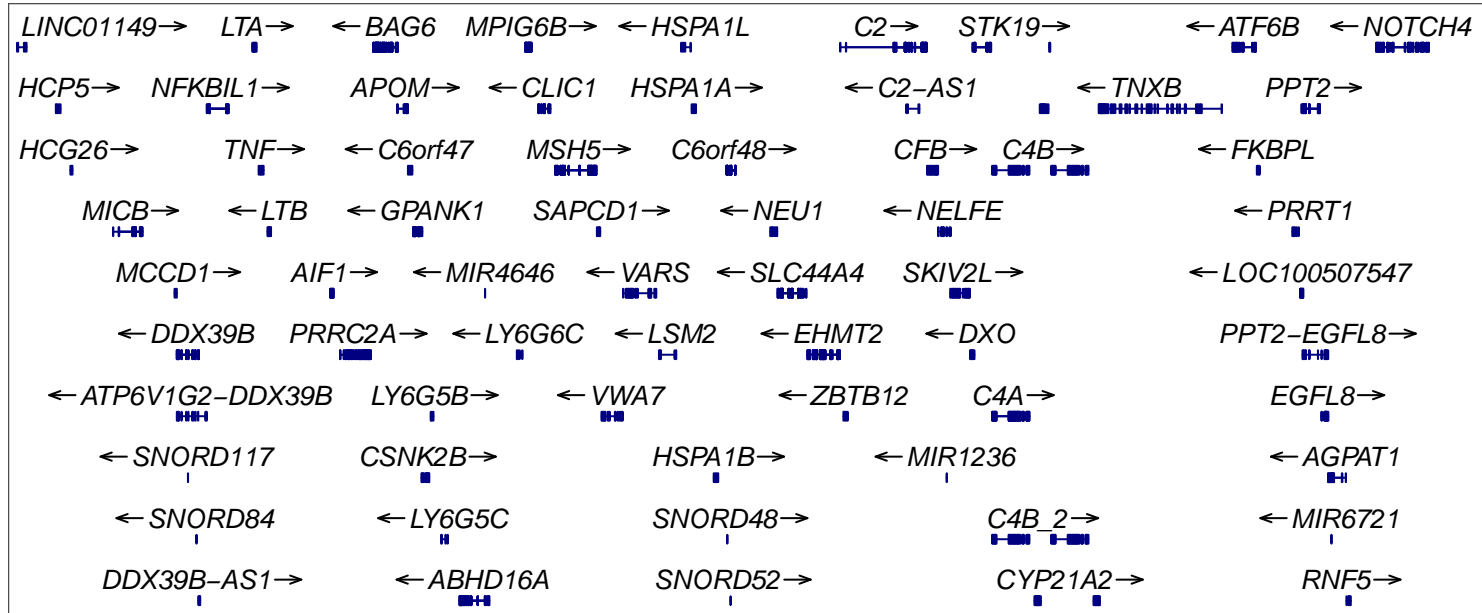

19 genes  
omitted

31.6

31.8

32

32.2

Position on chr6 (Mb)

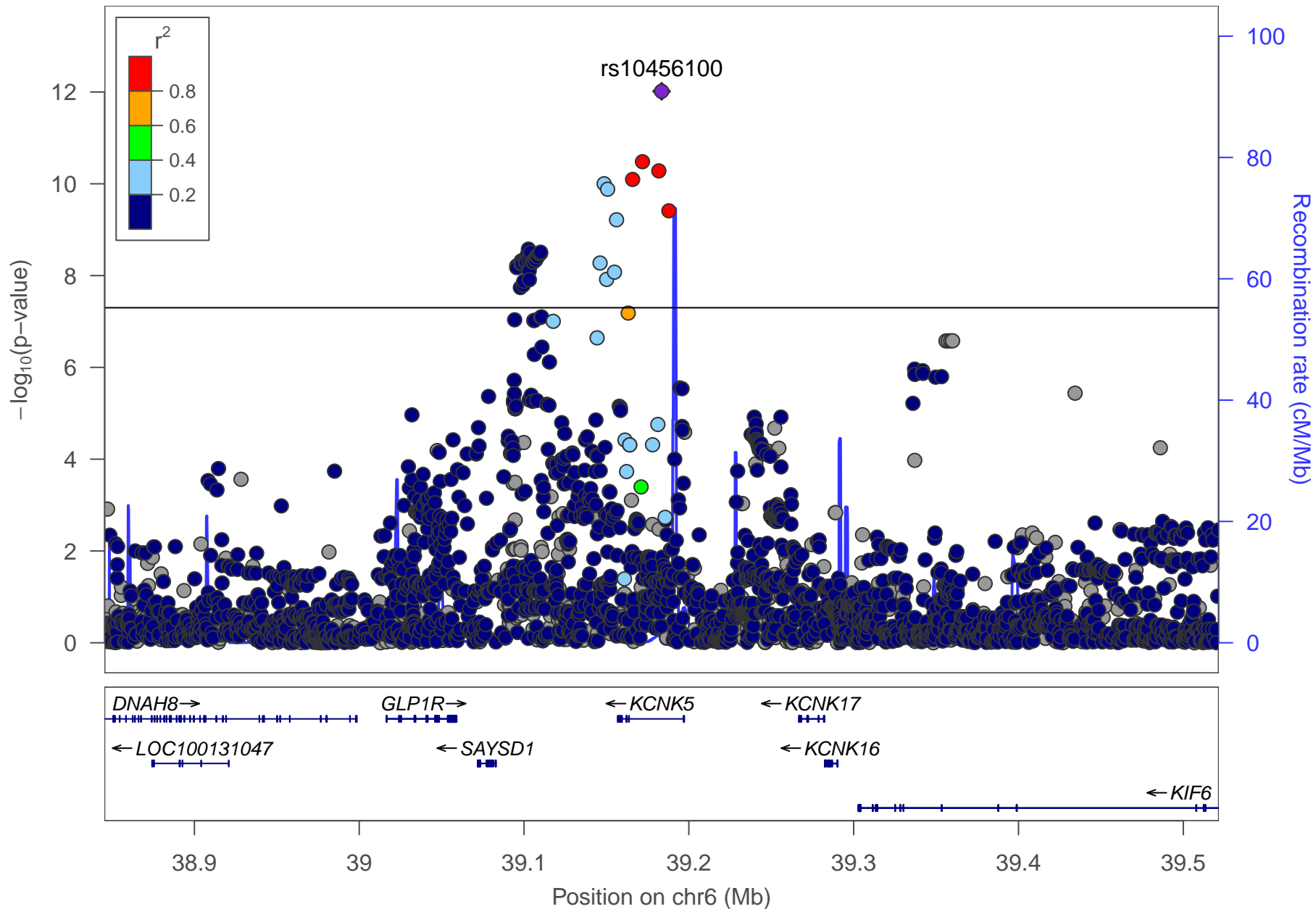

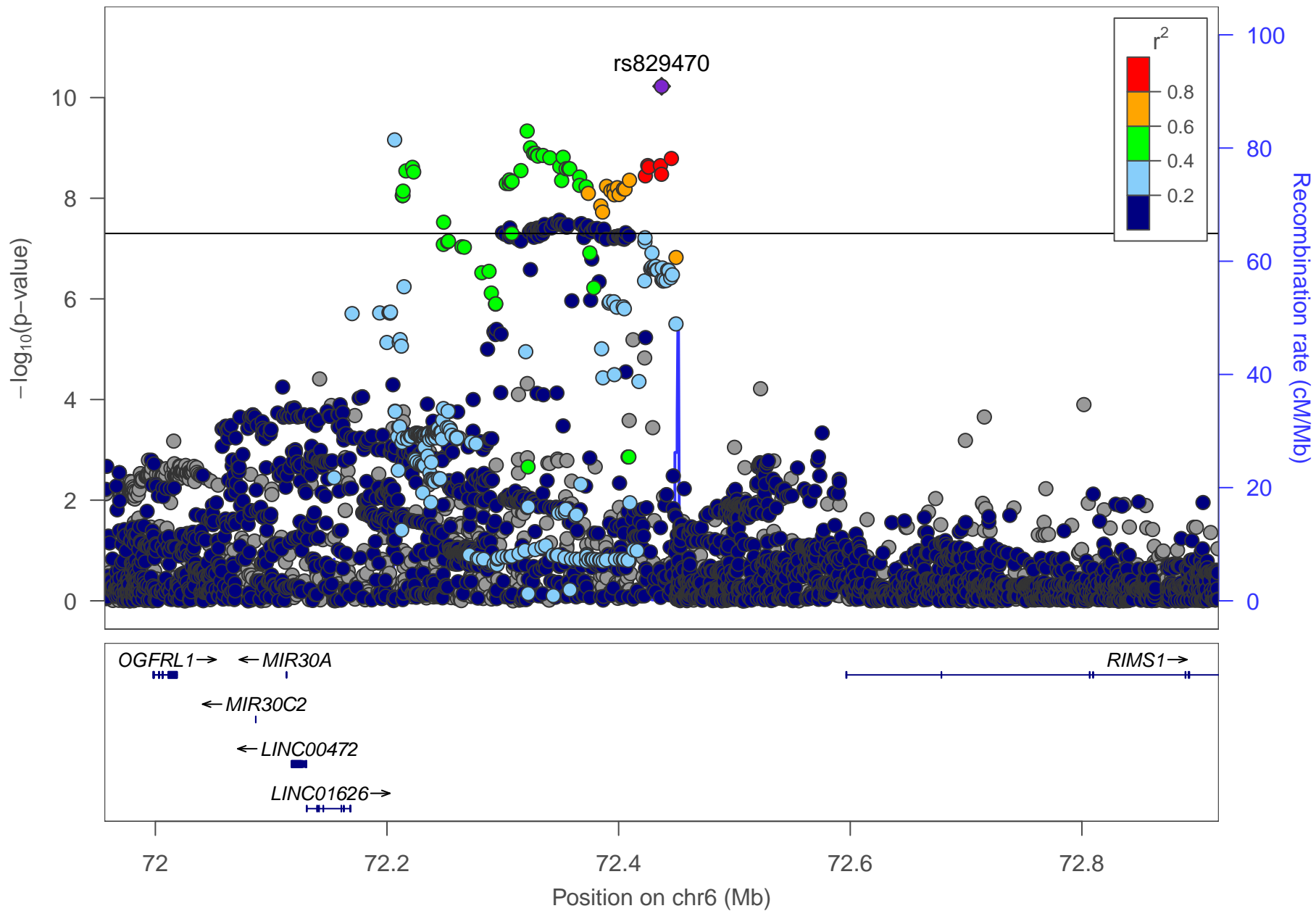

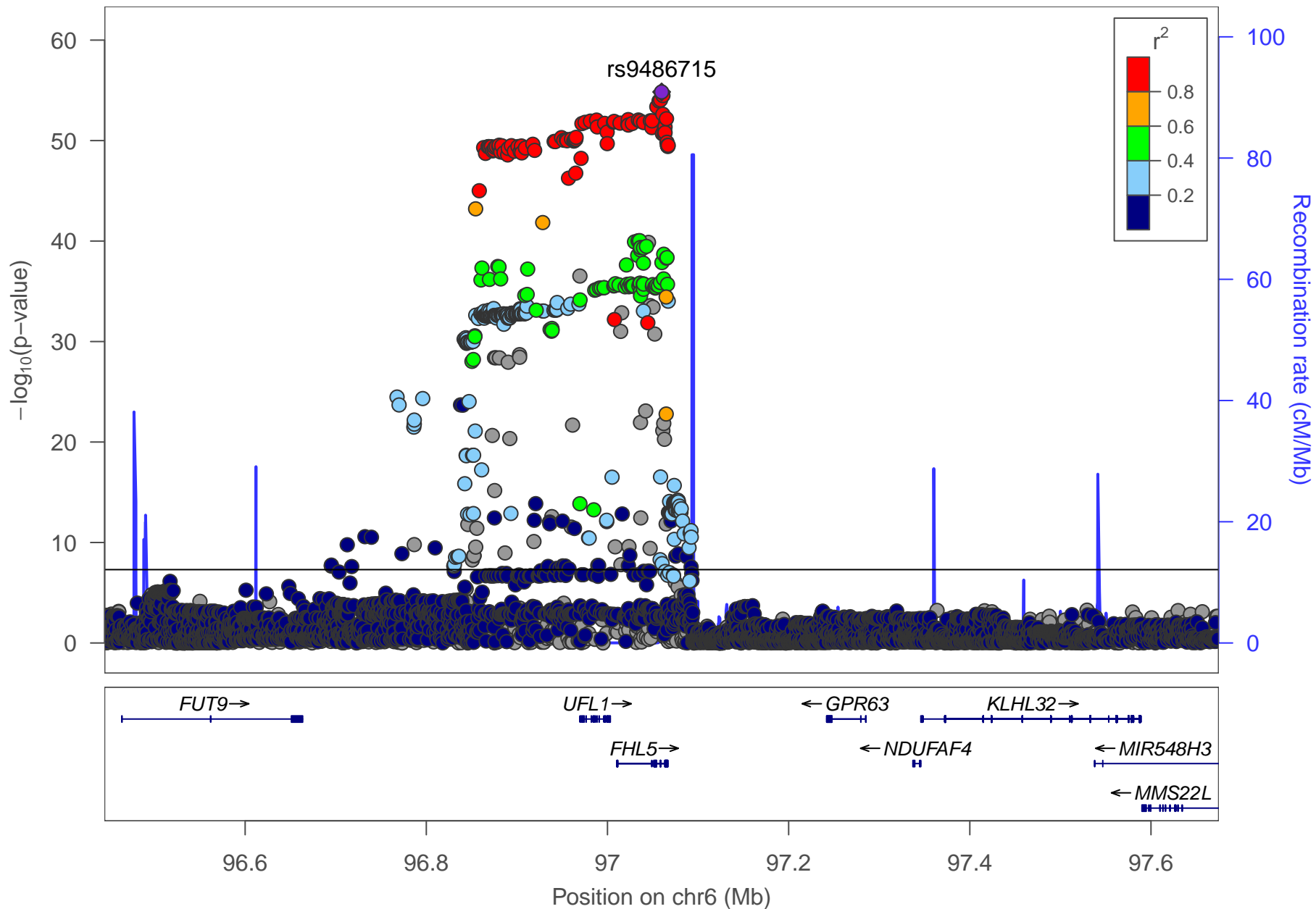

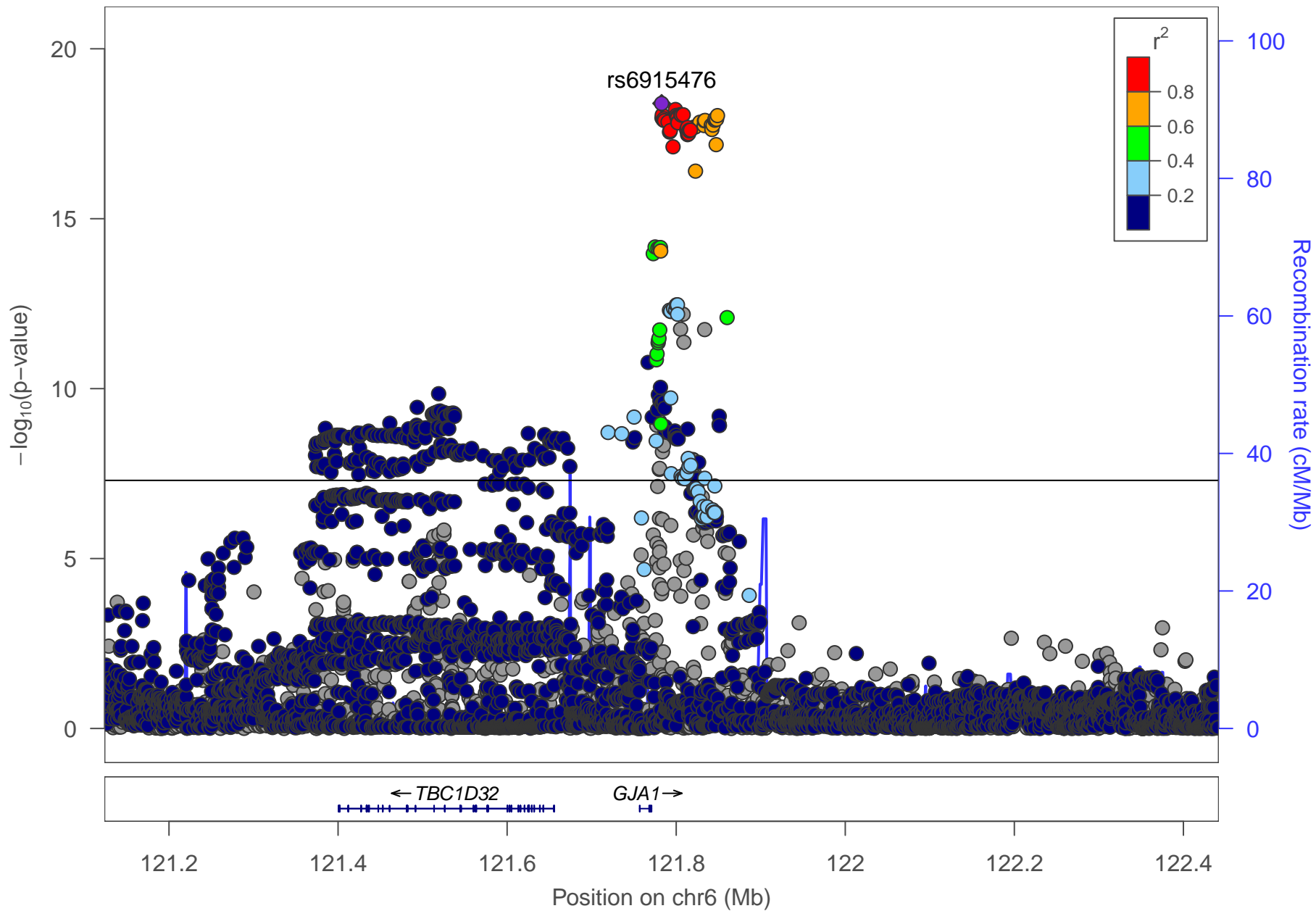

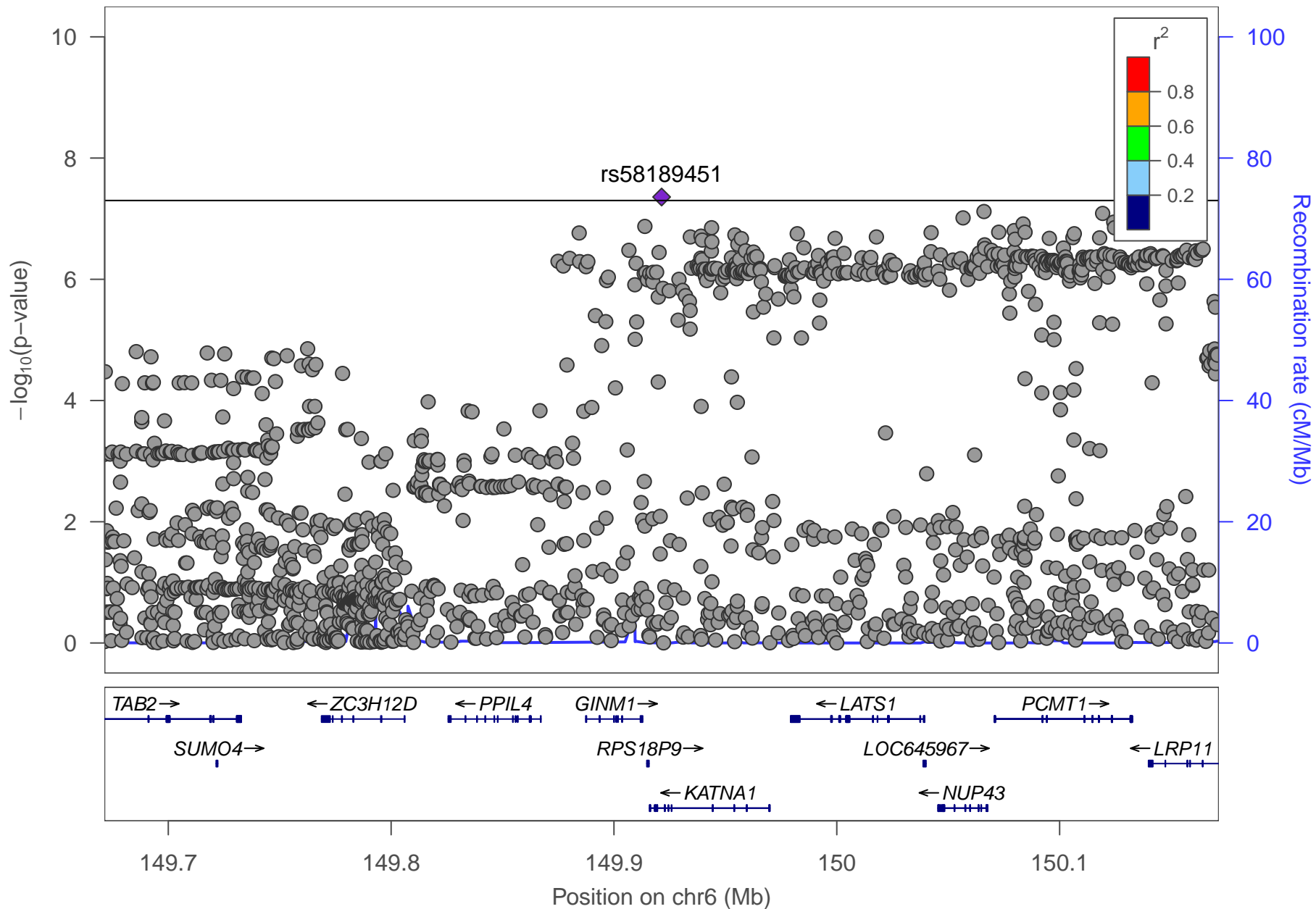

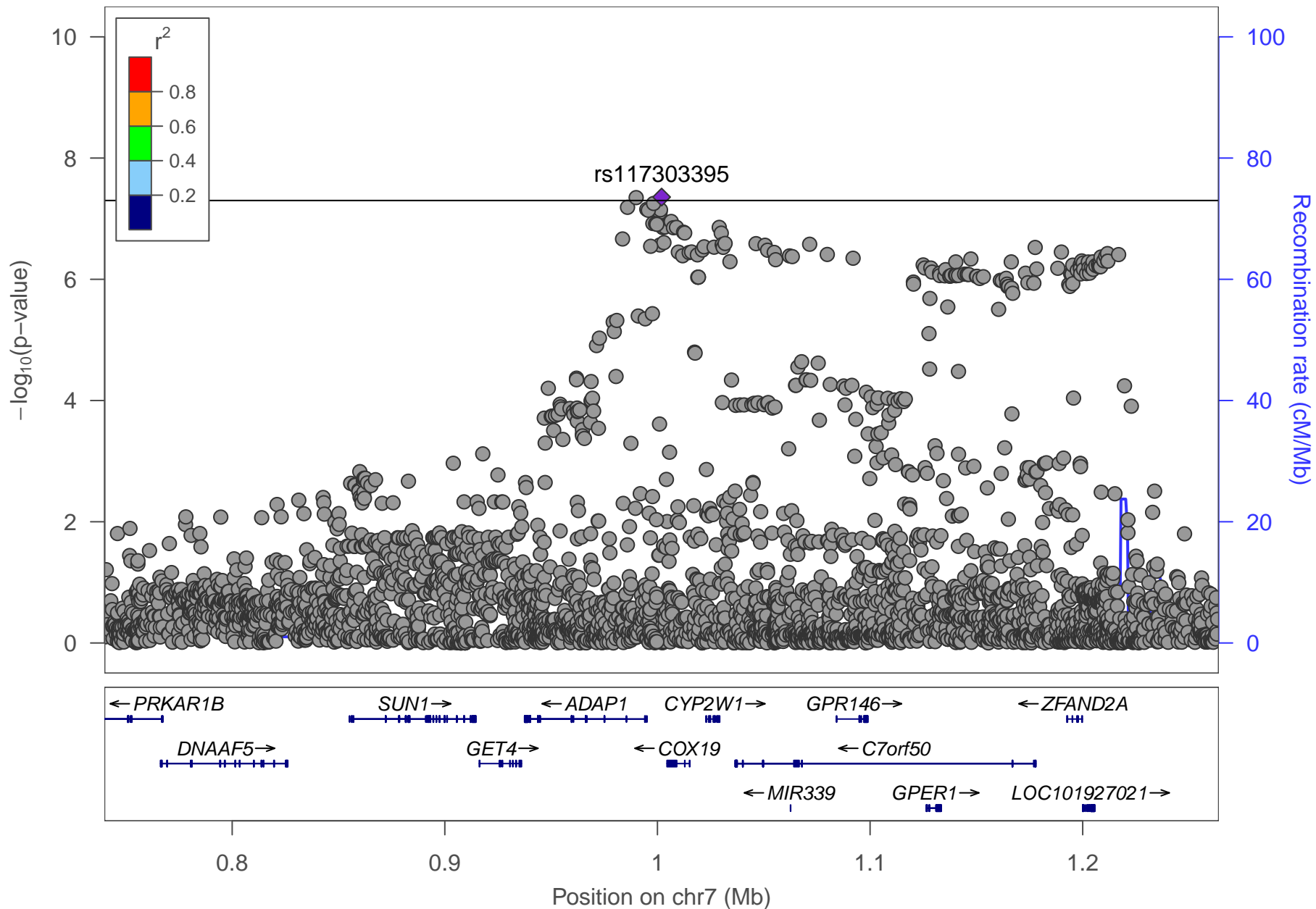

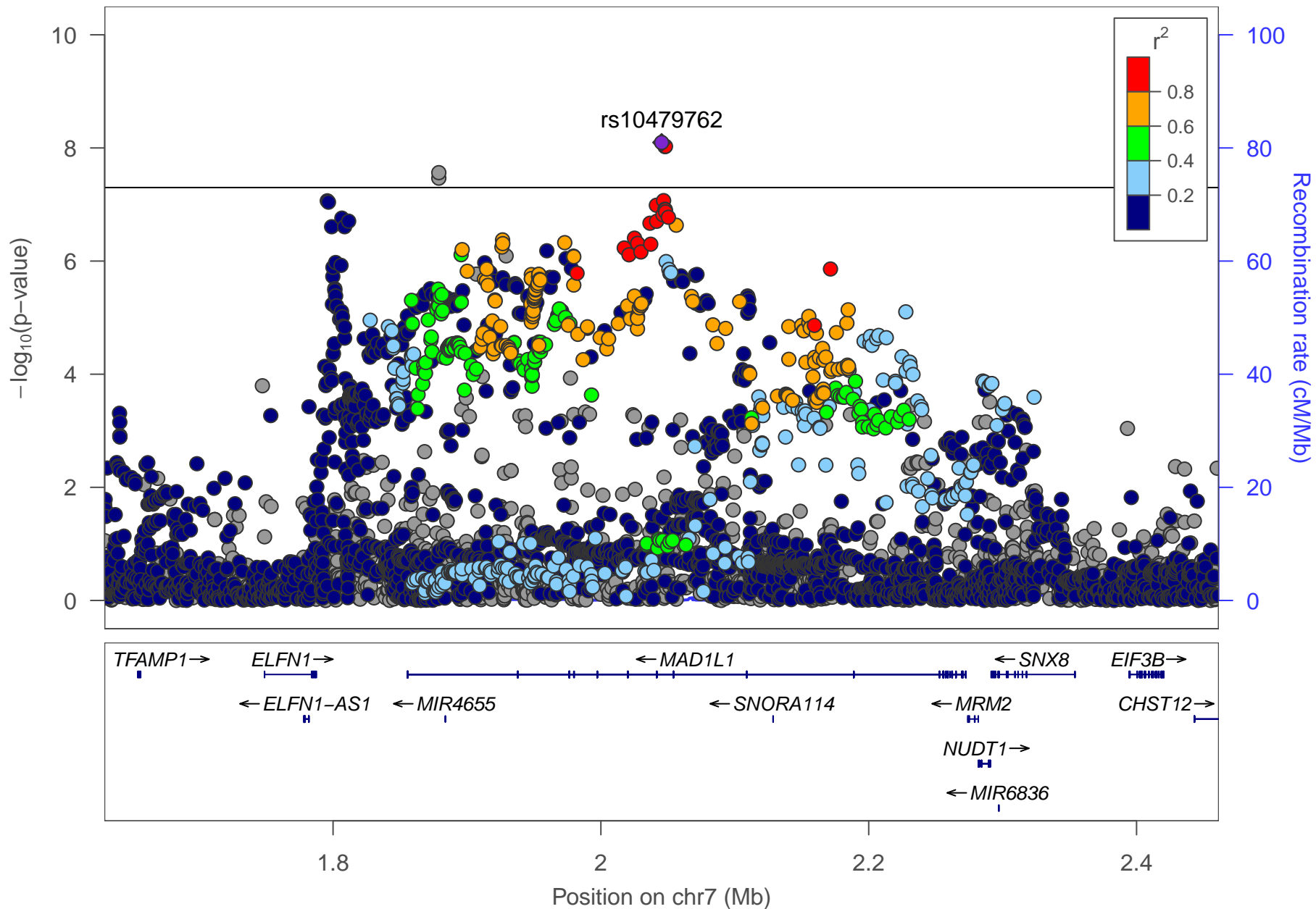

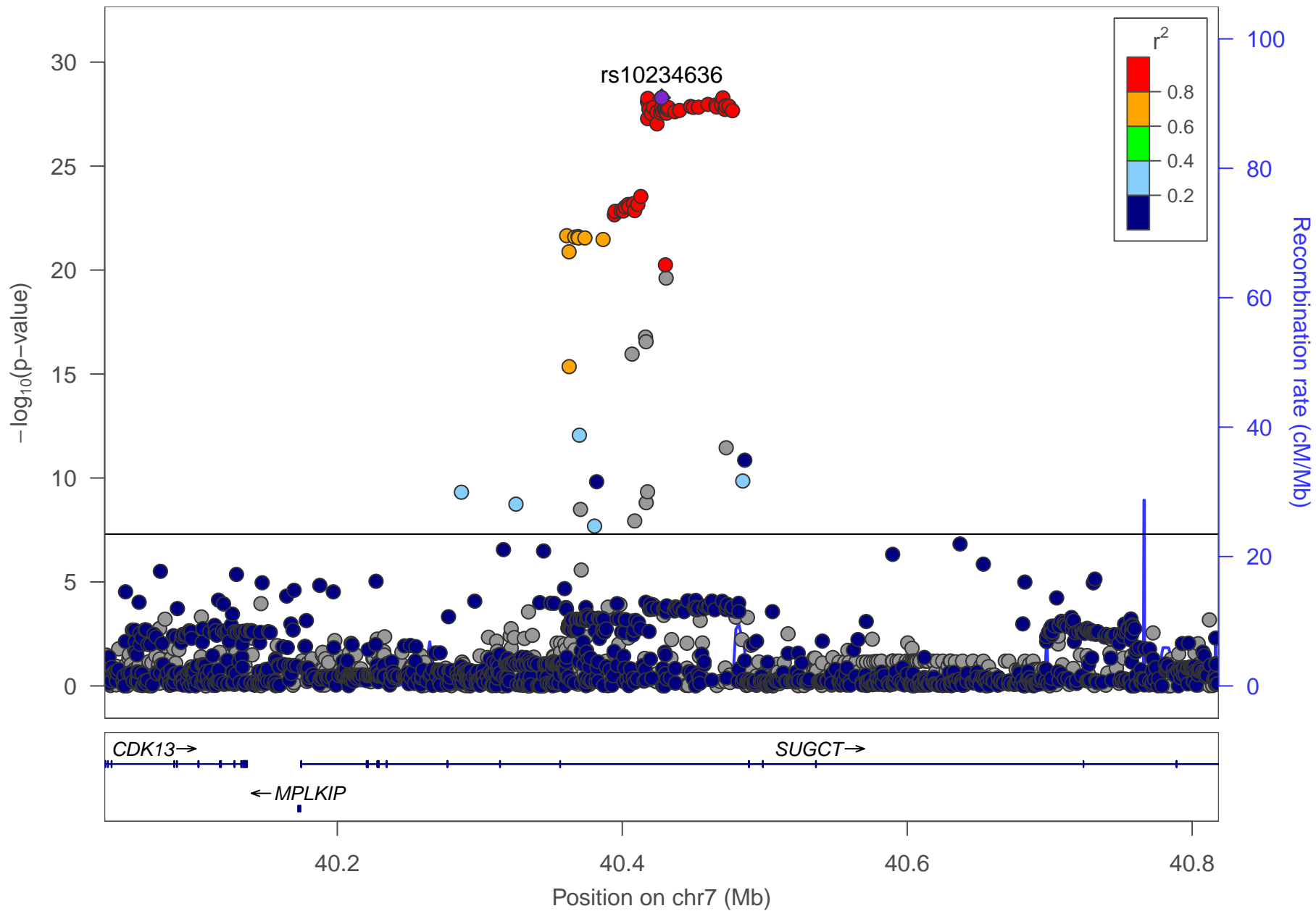

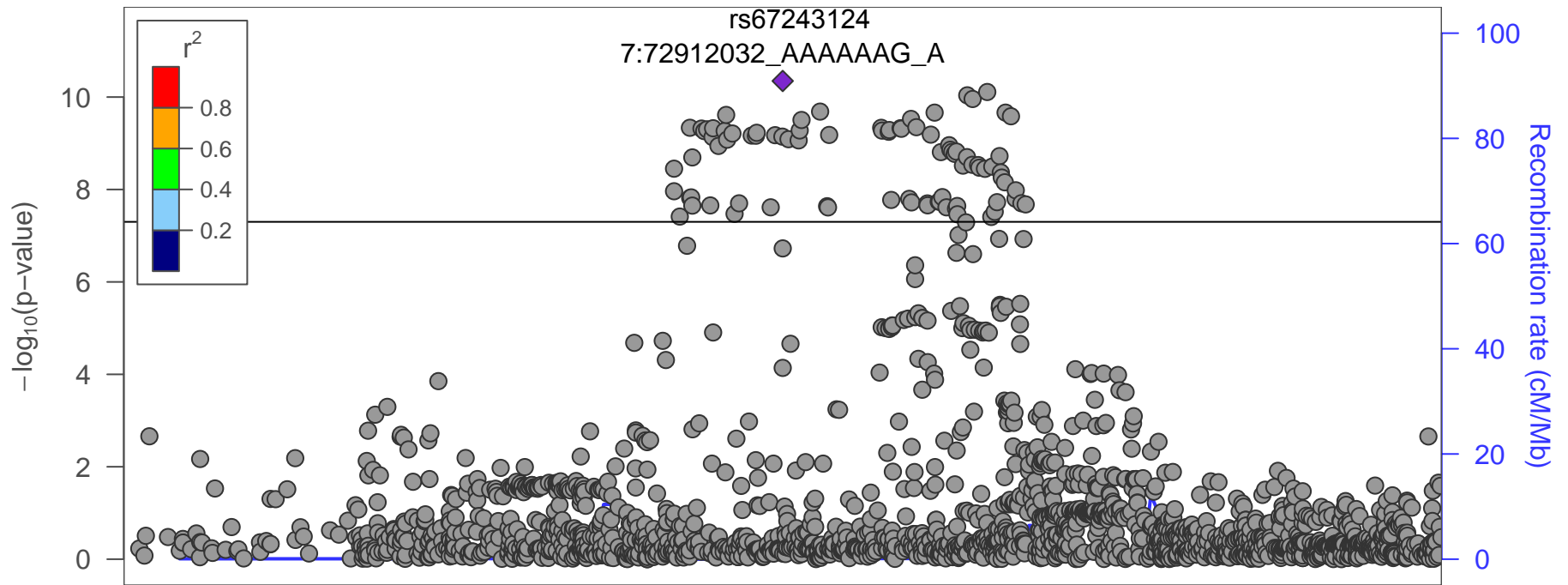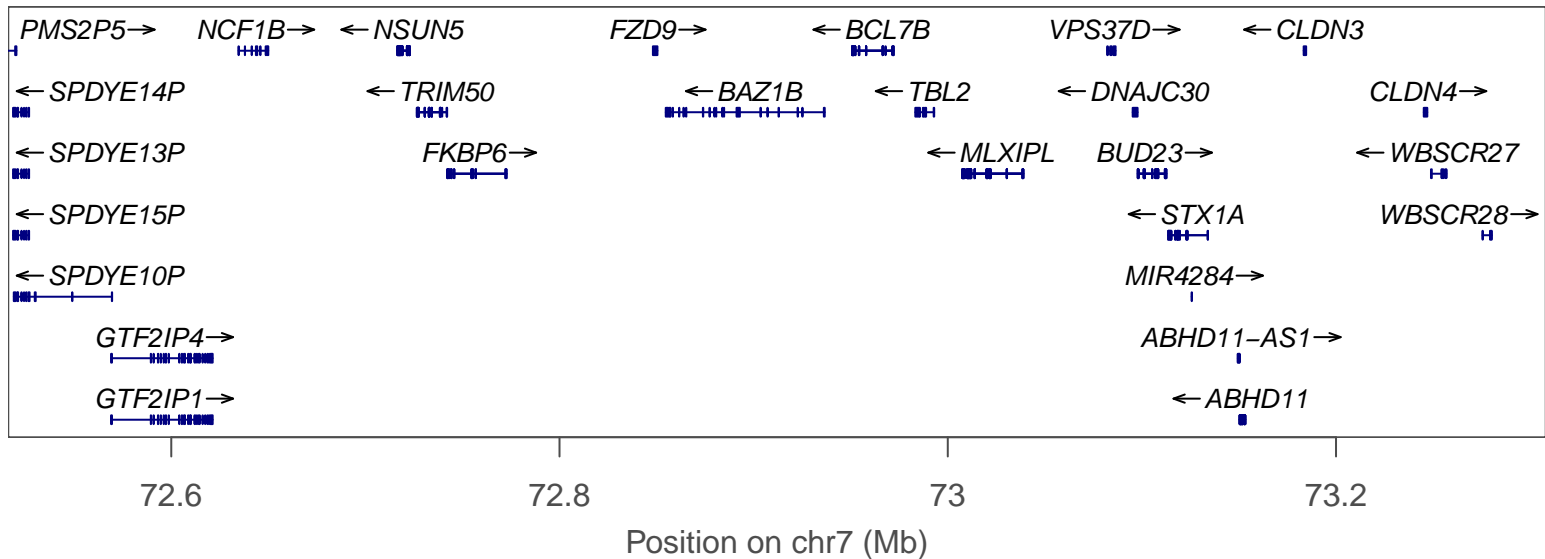

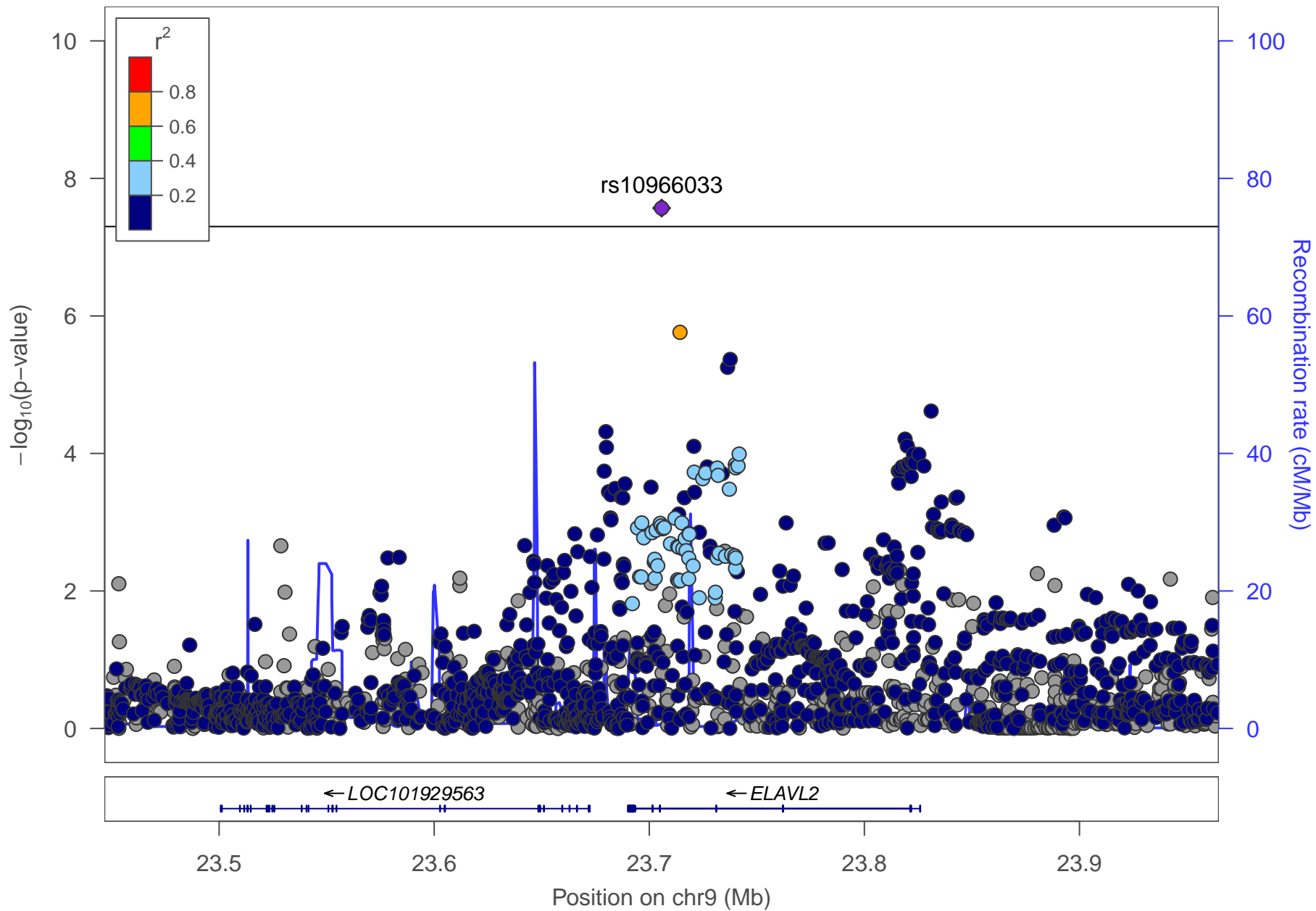

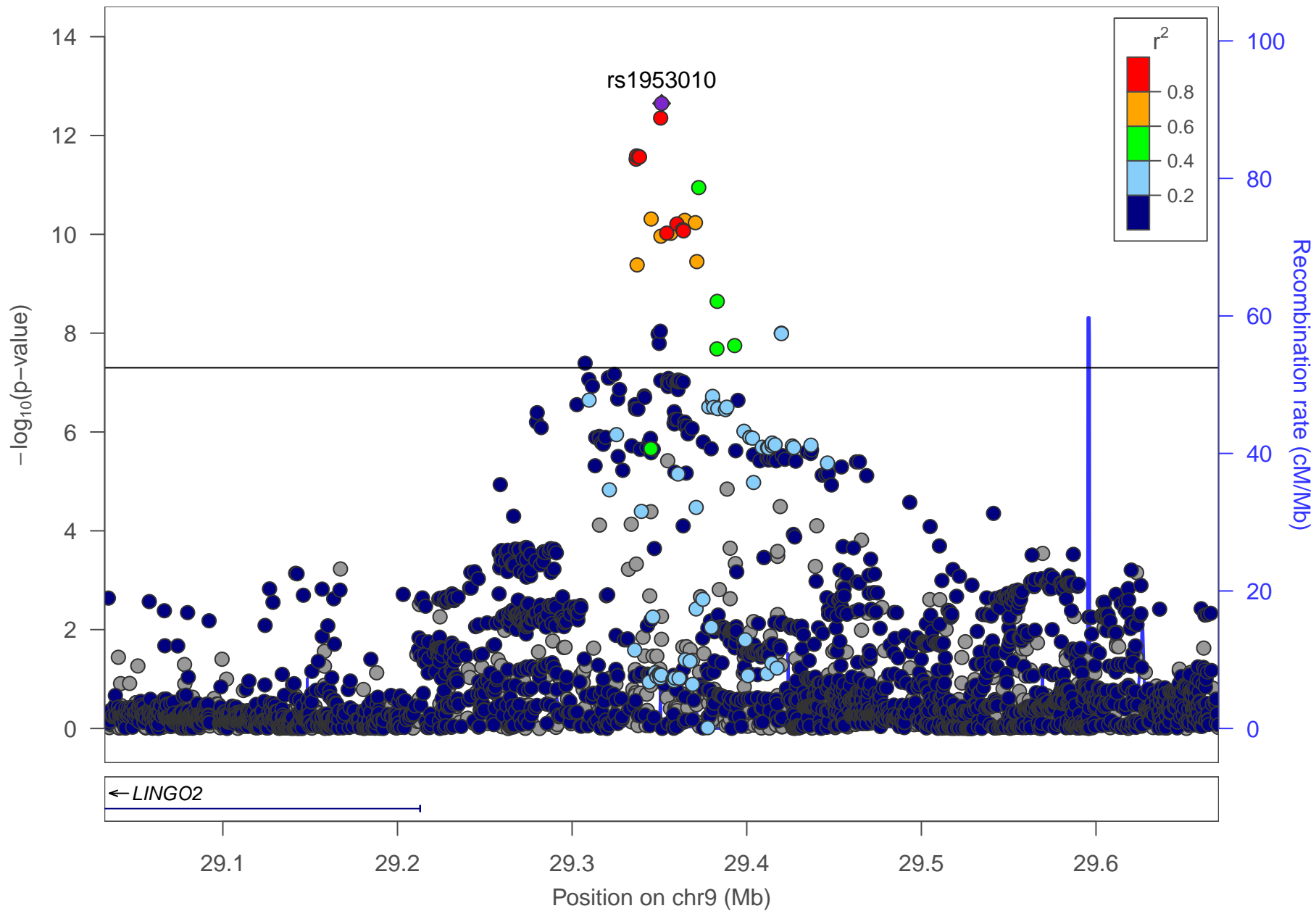

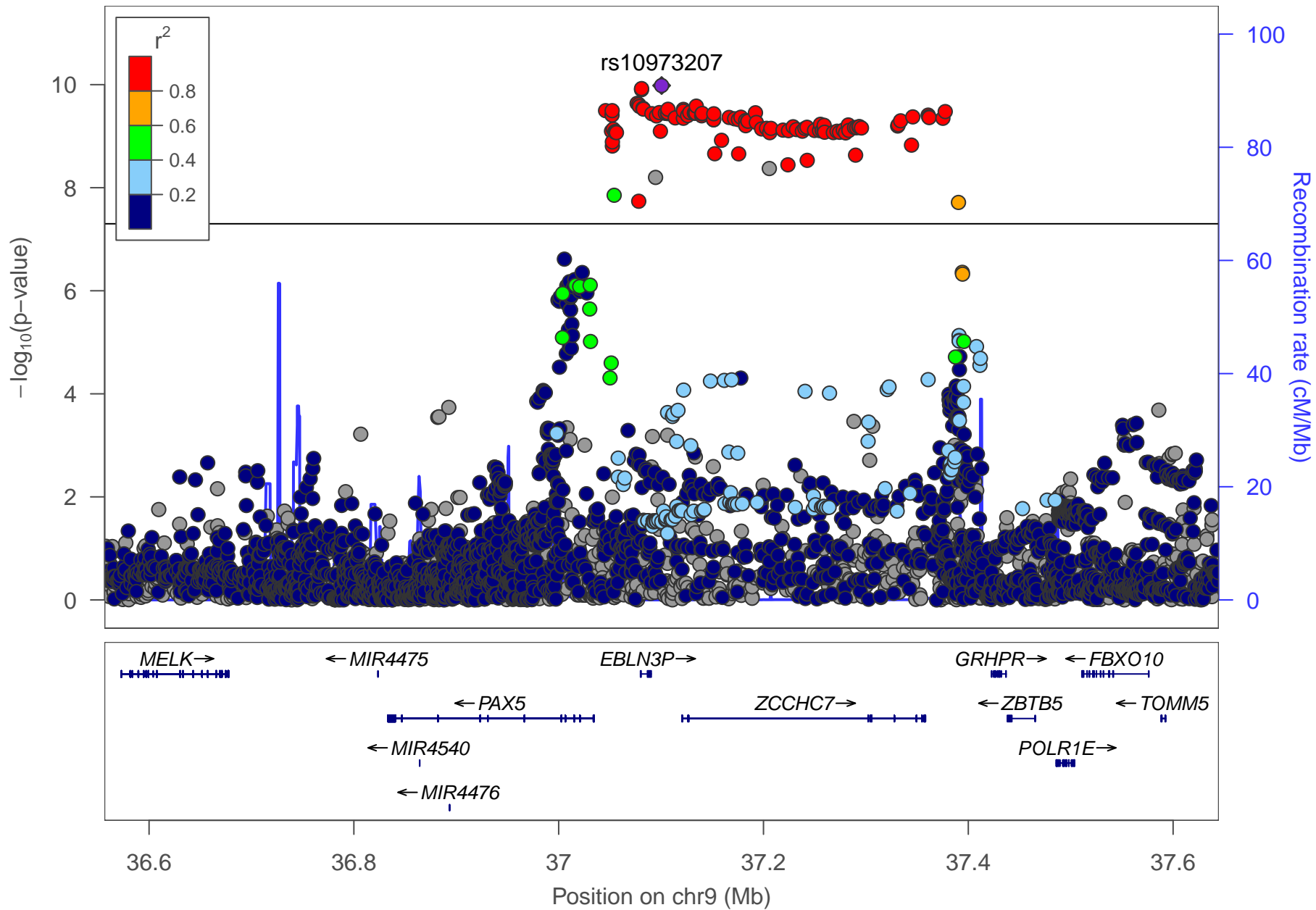

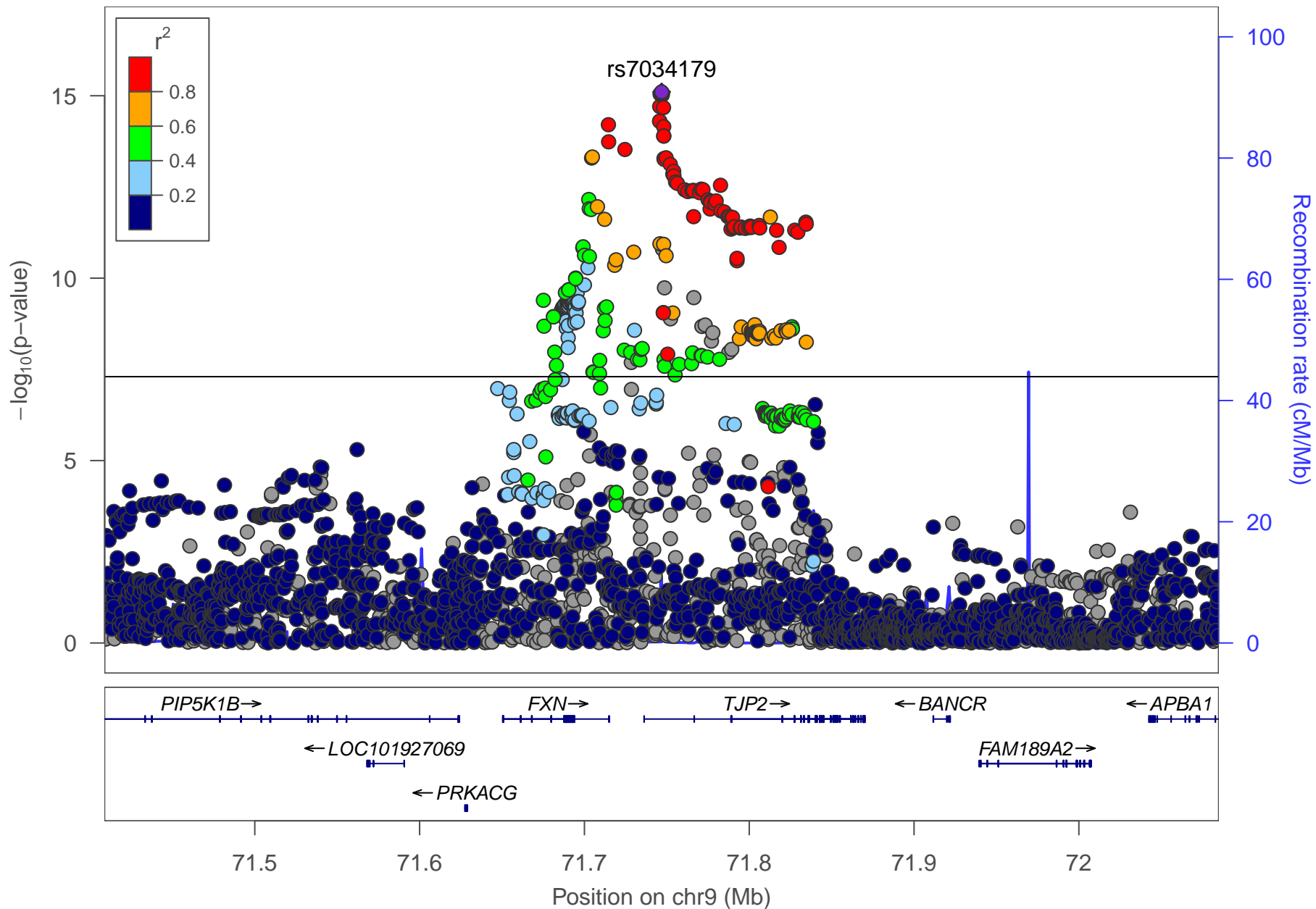

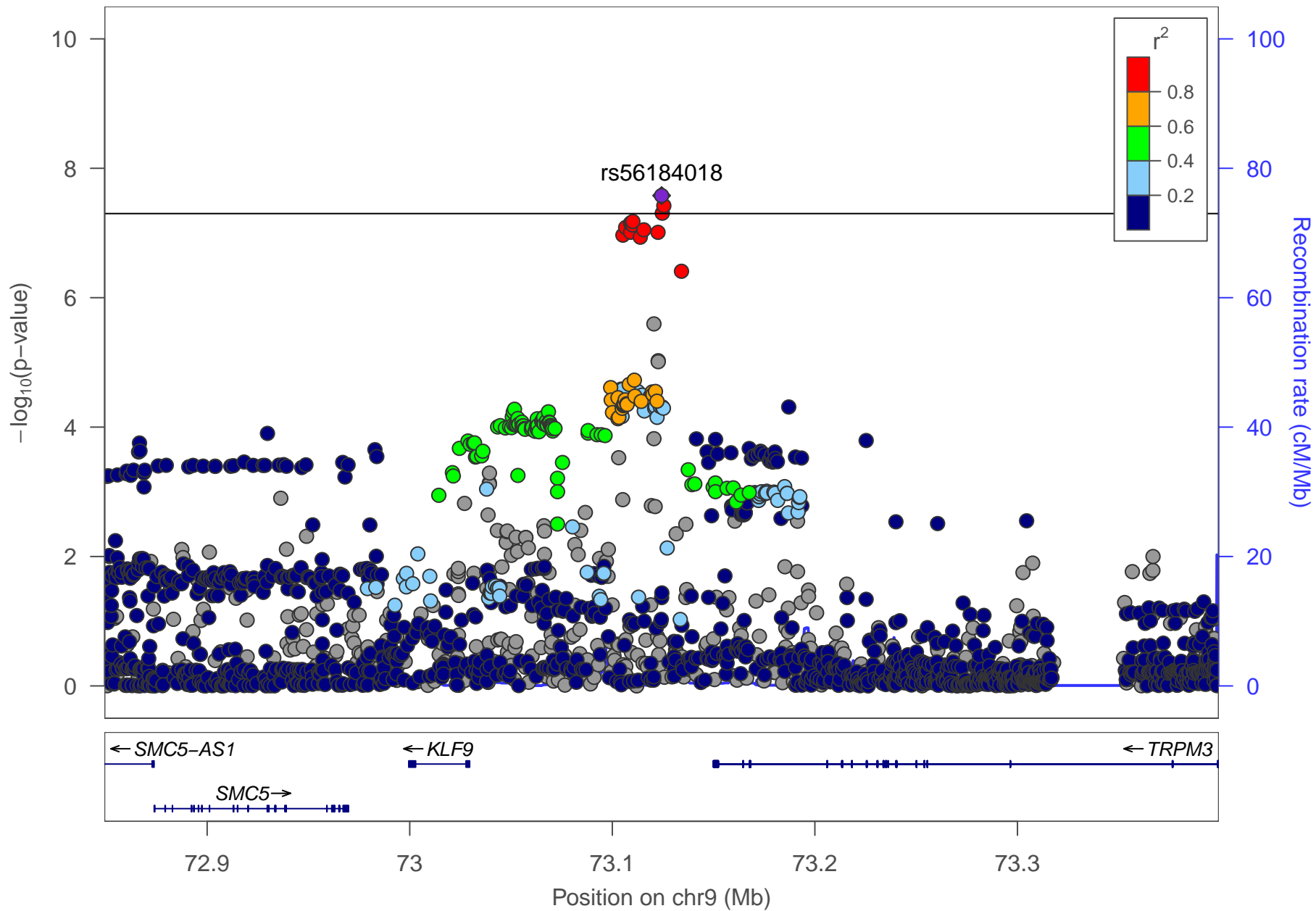

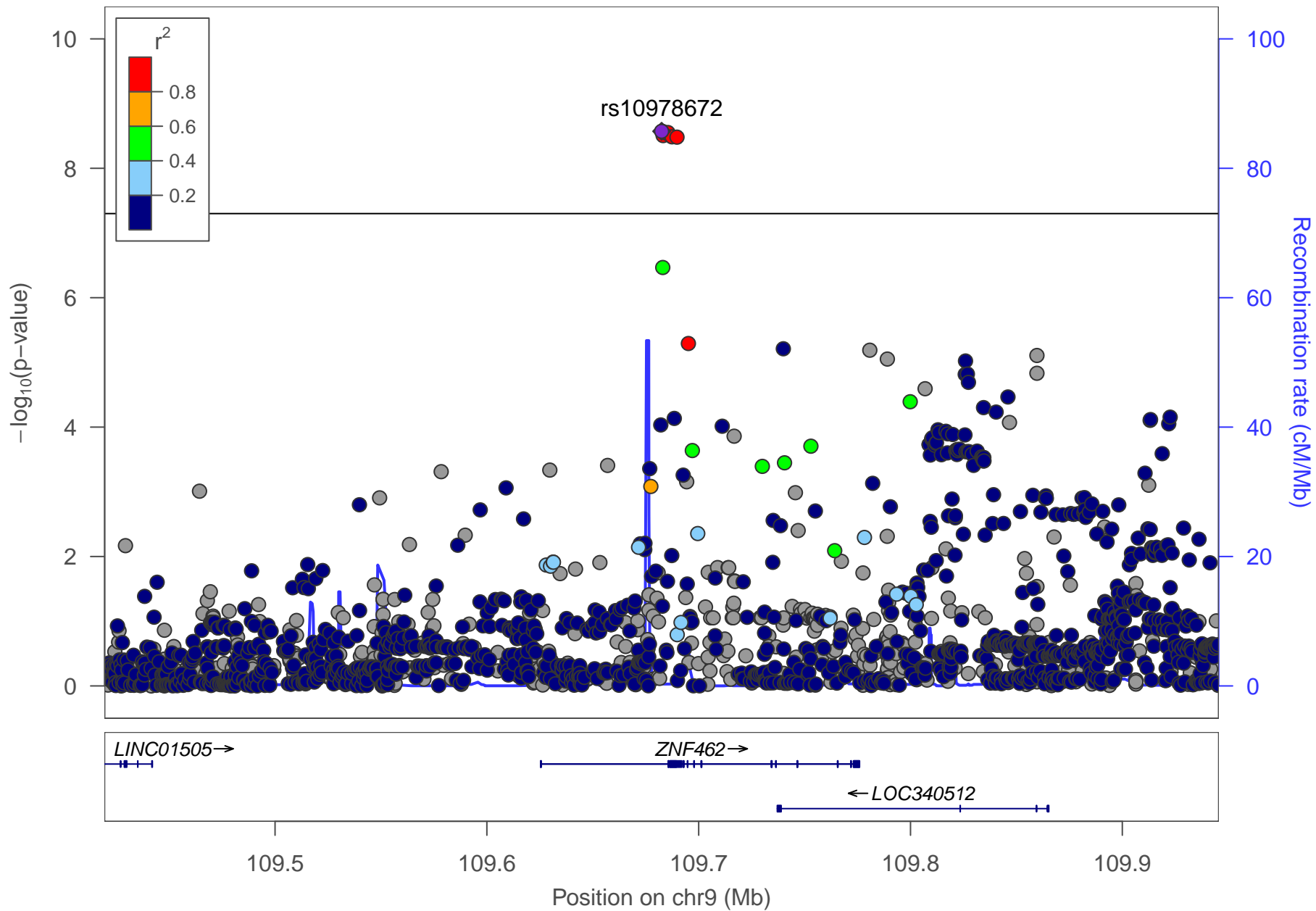

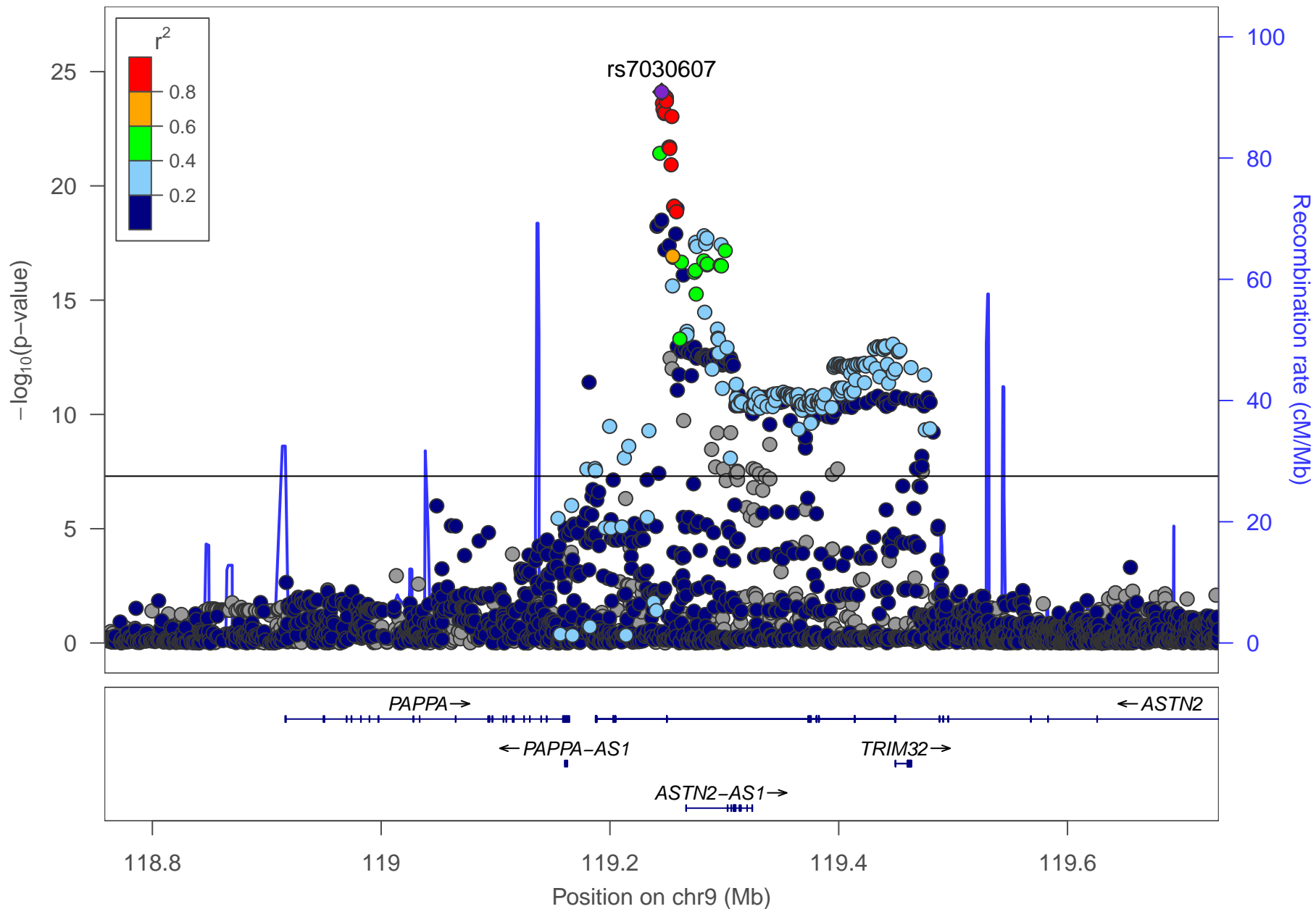

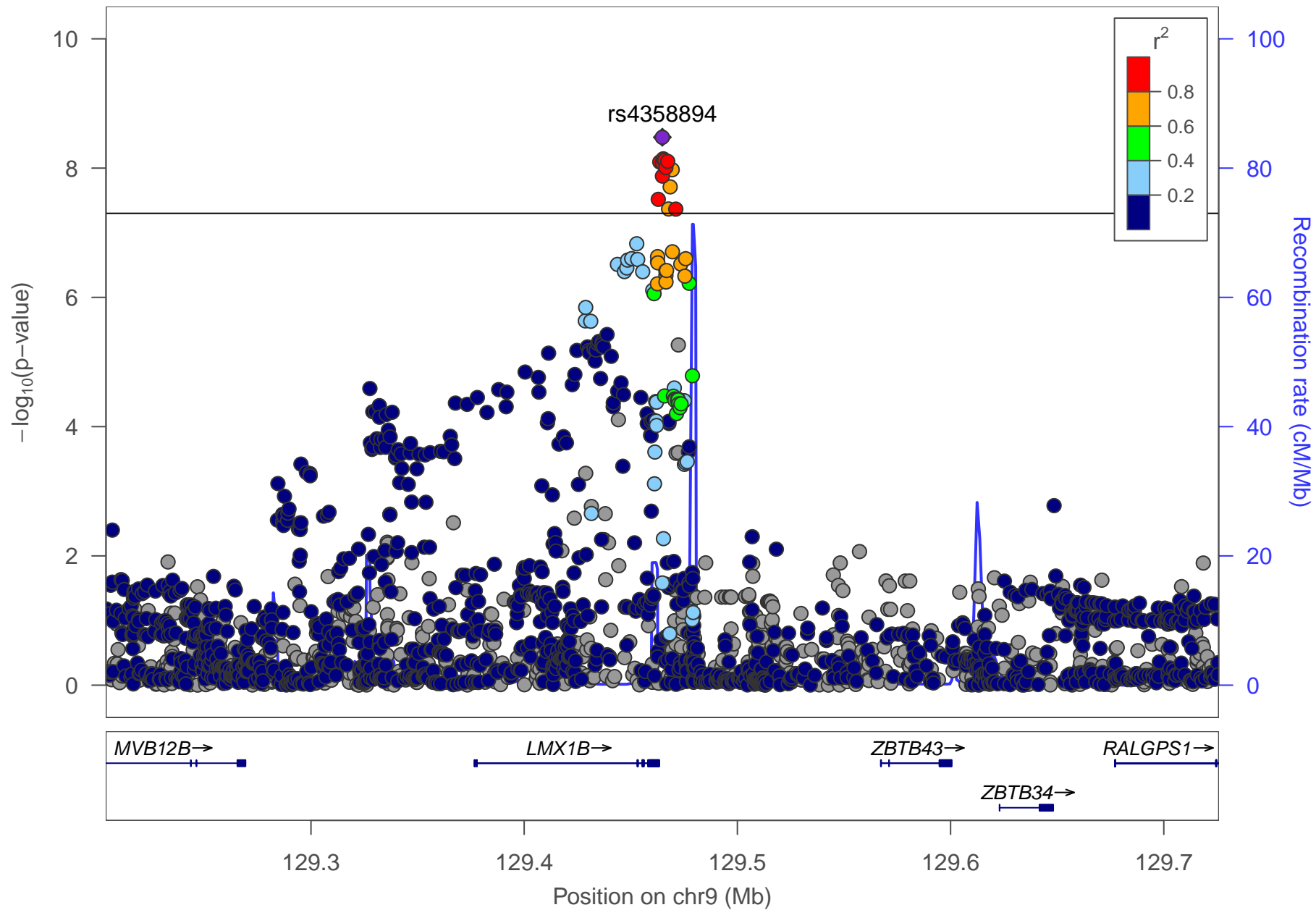

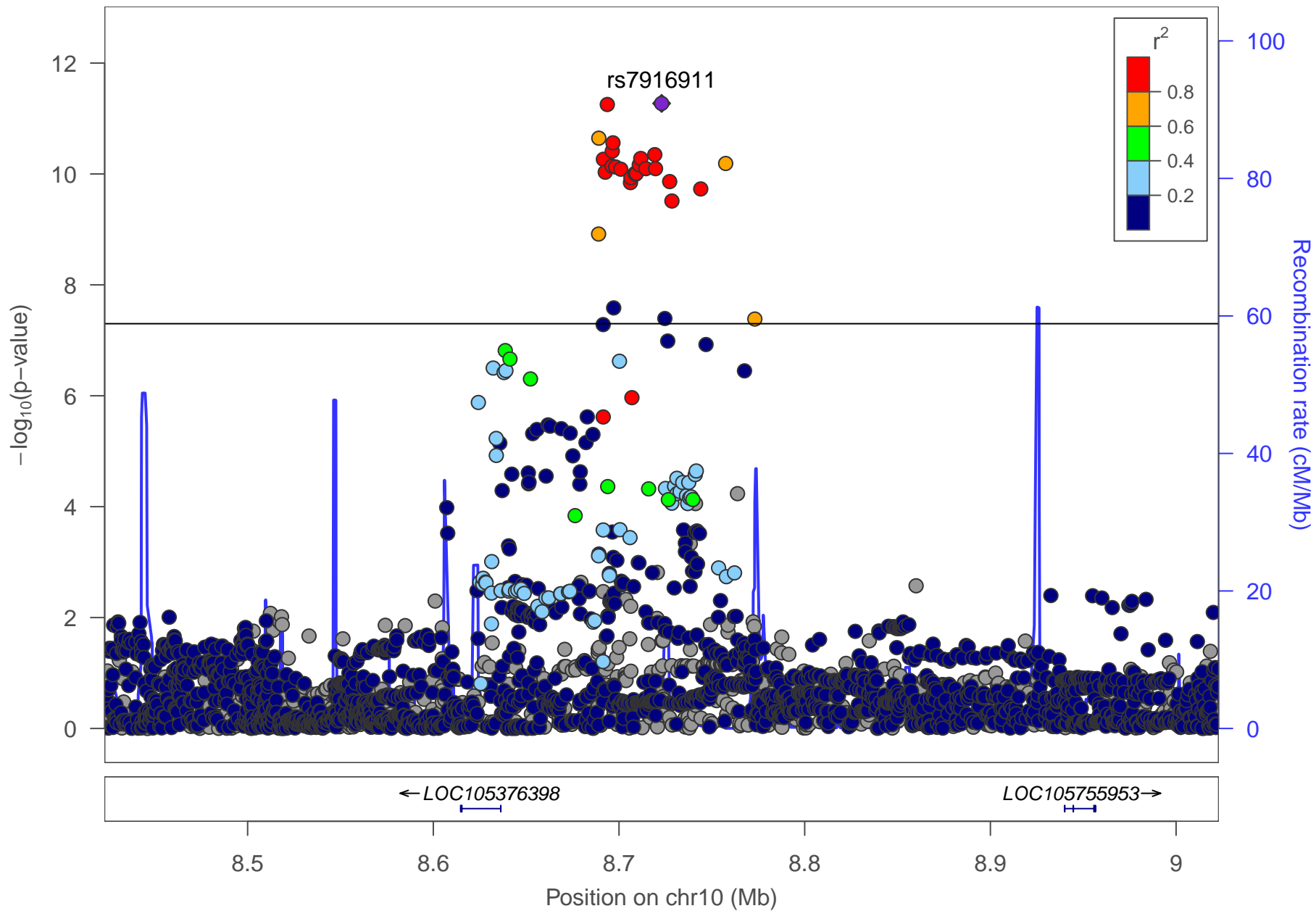

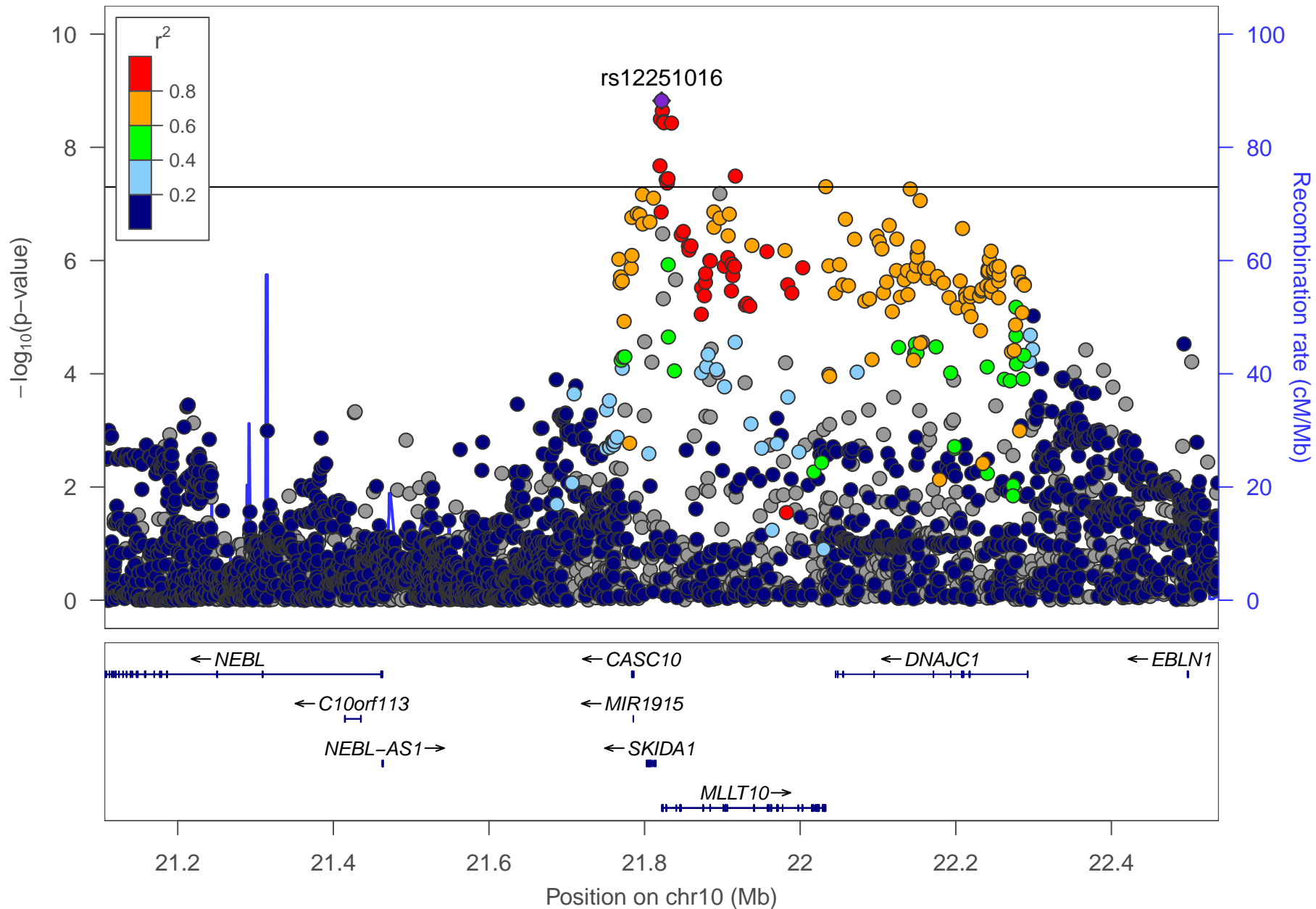

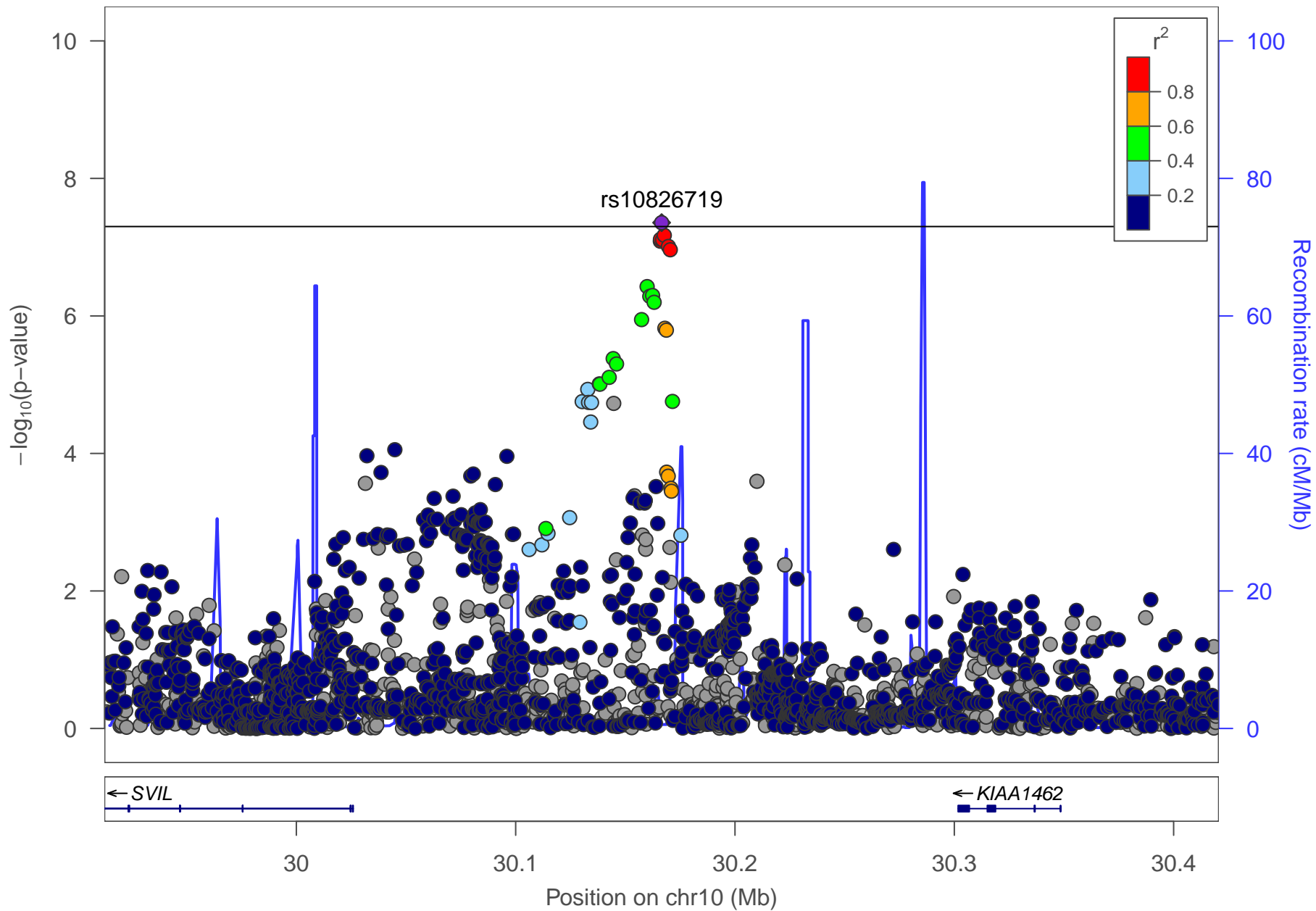

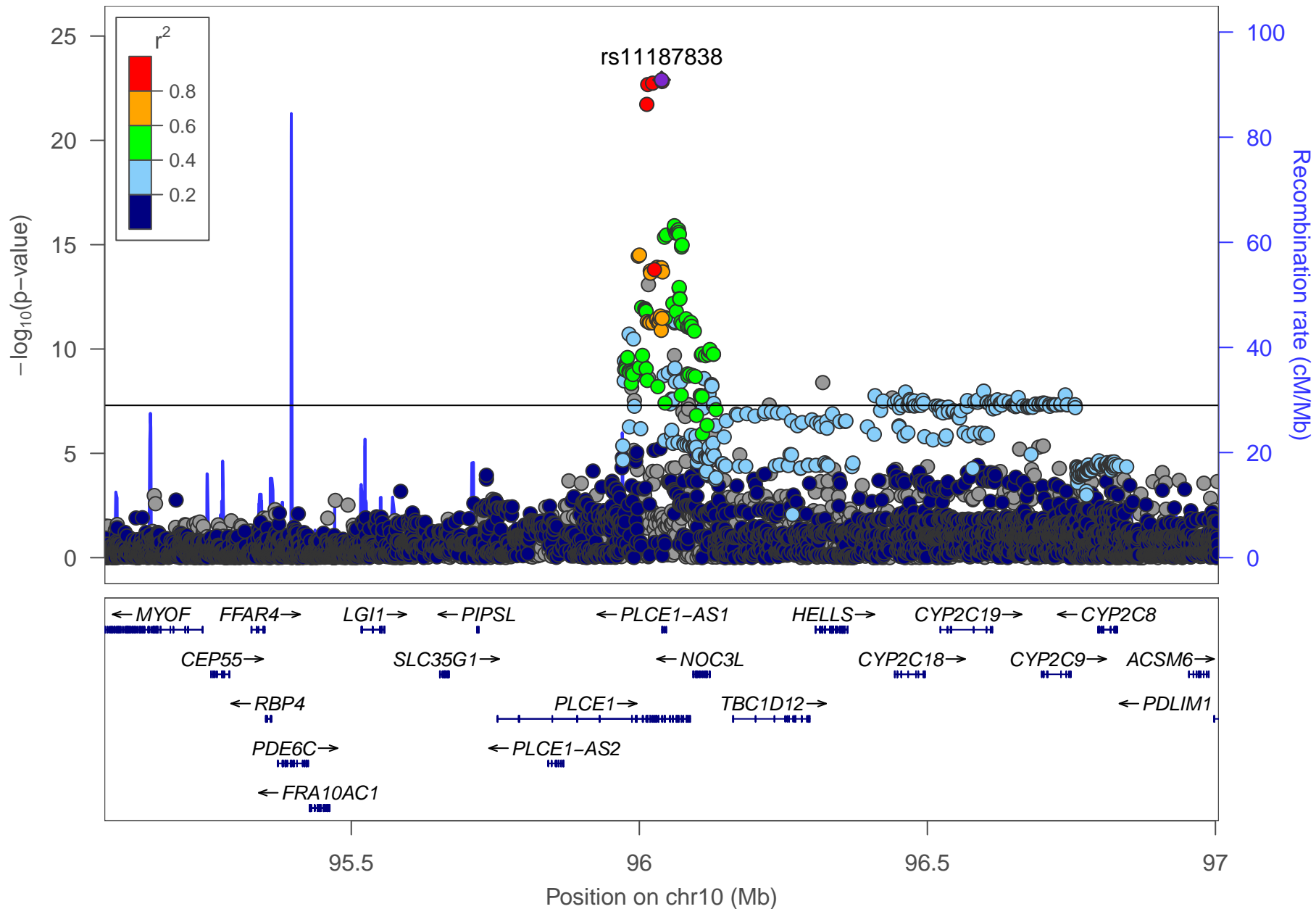

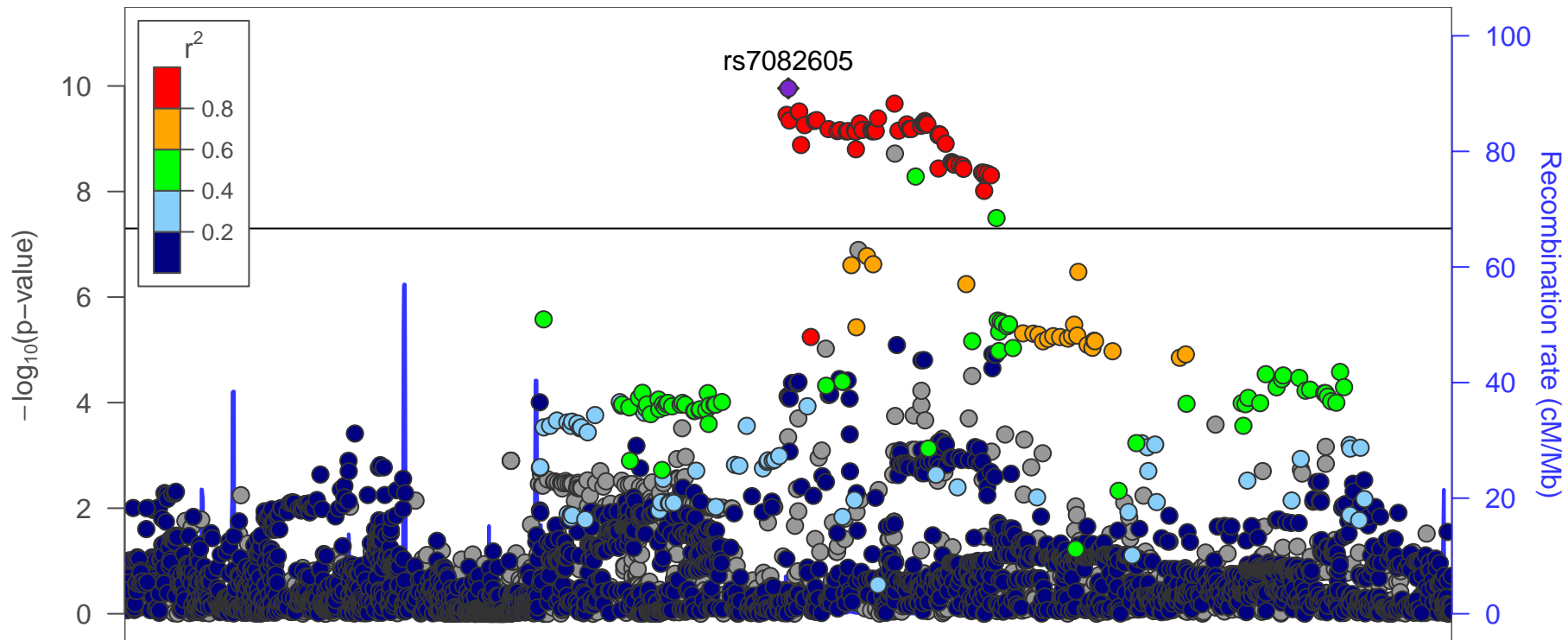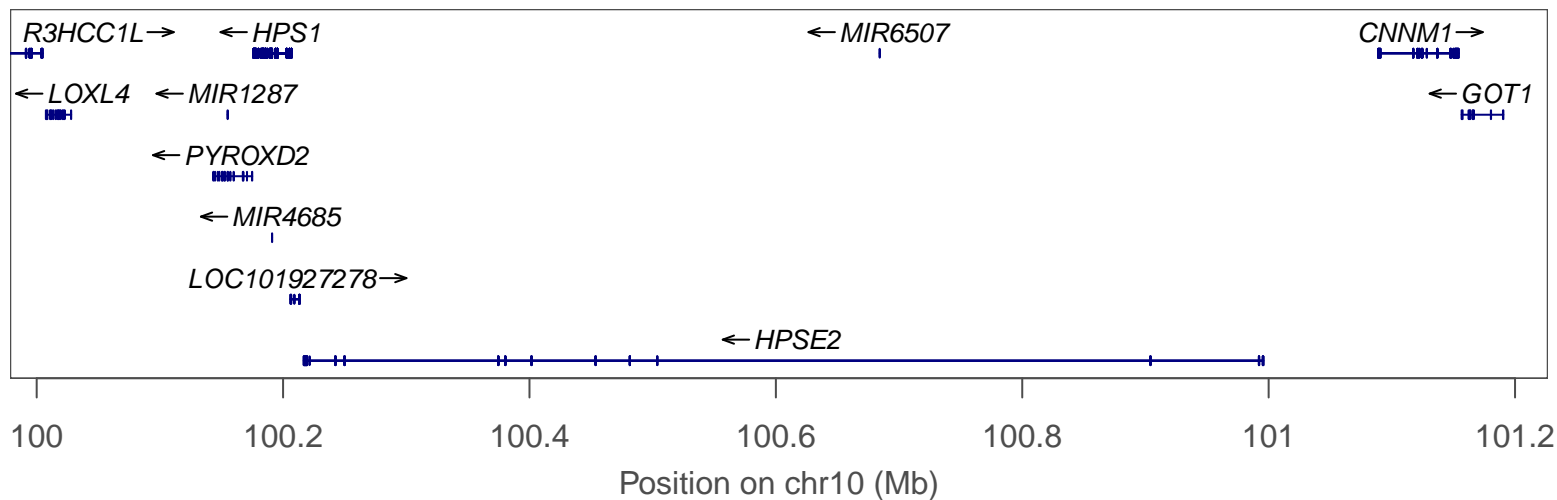

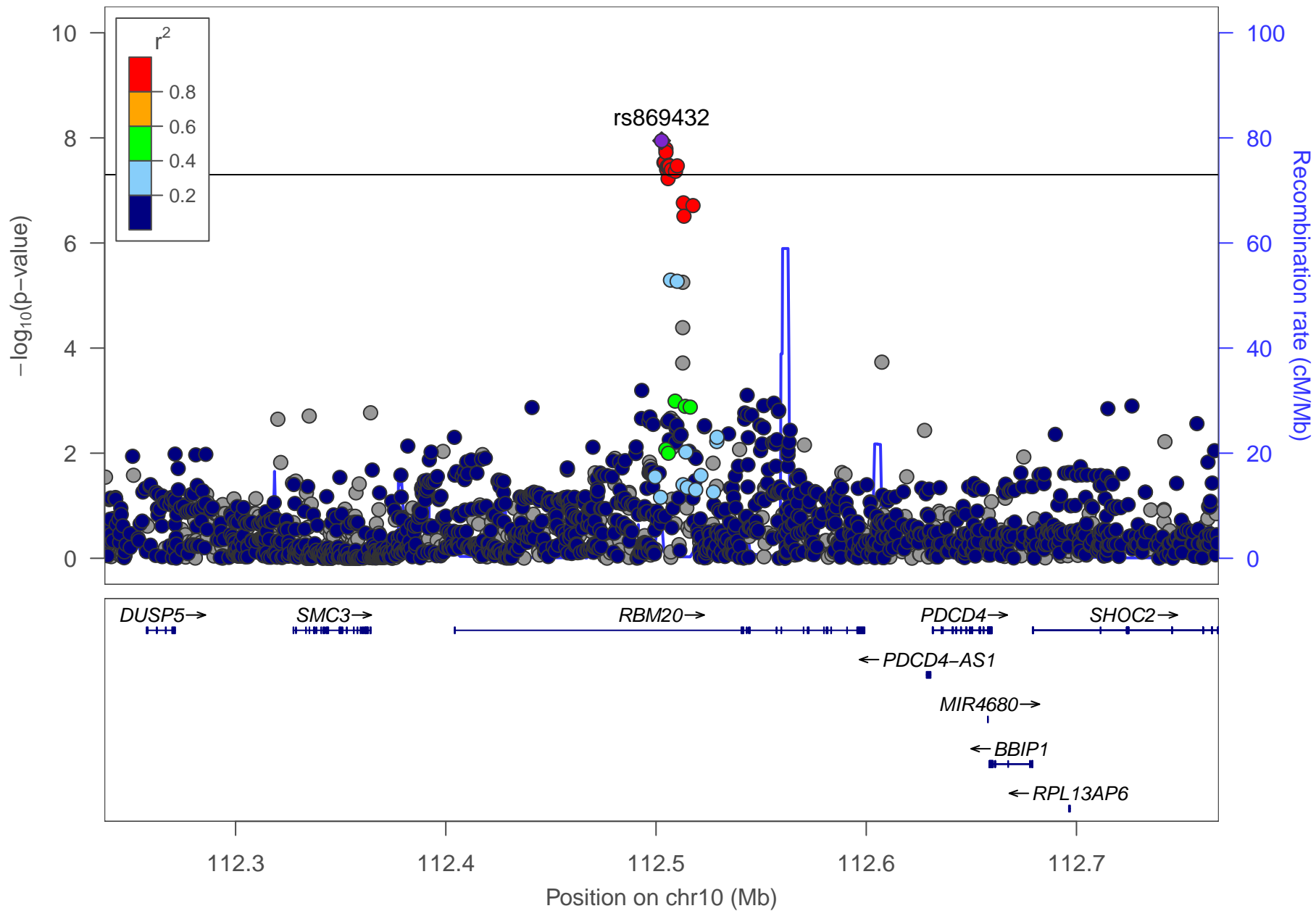

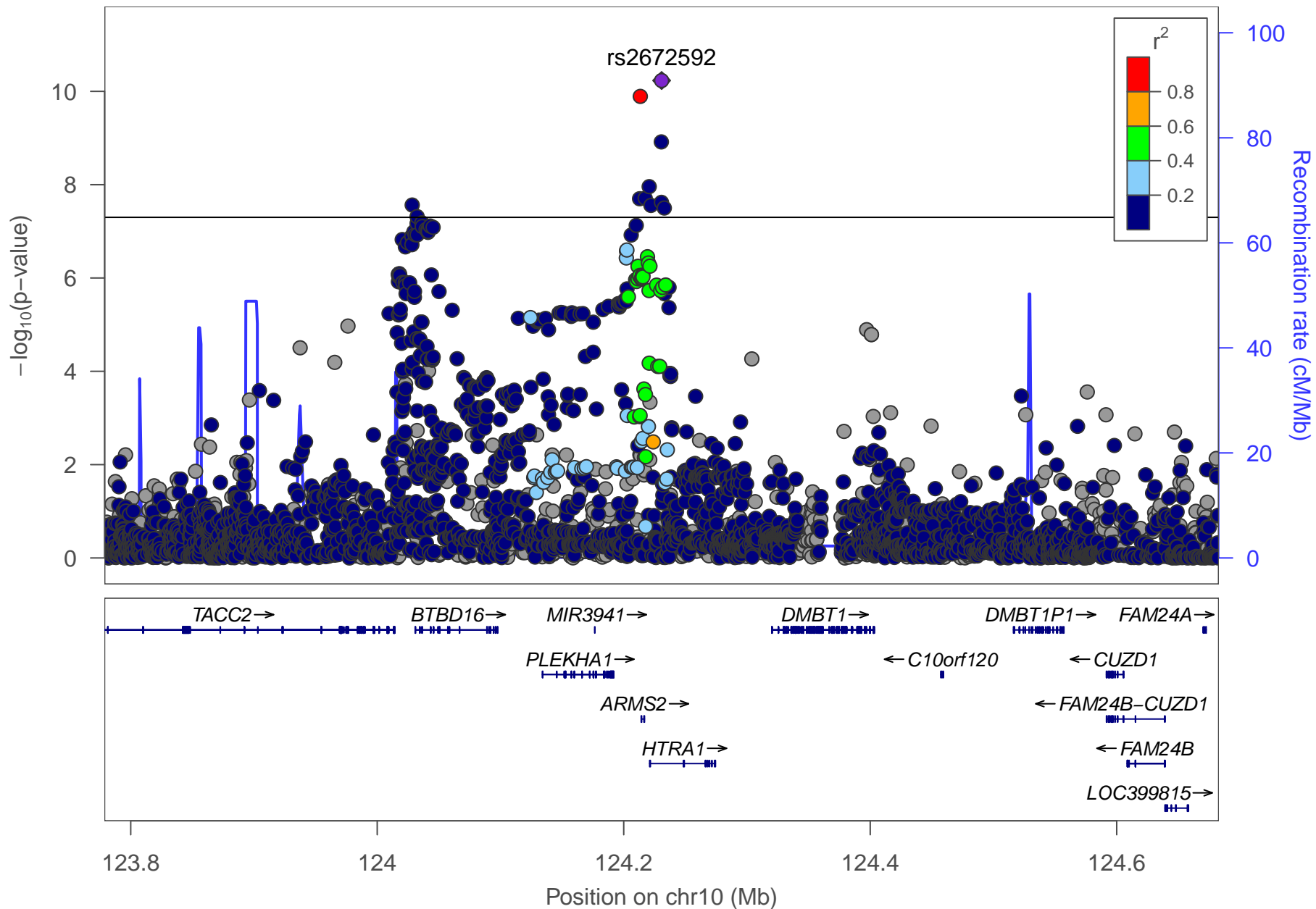

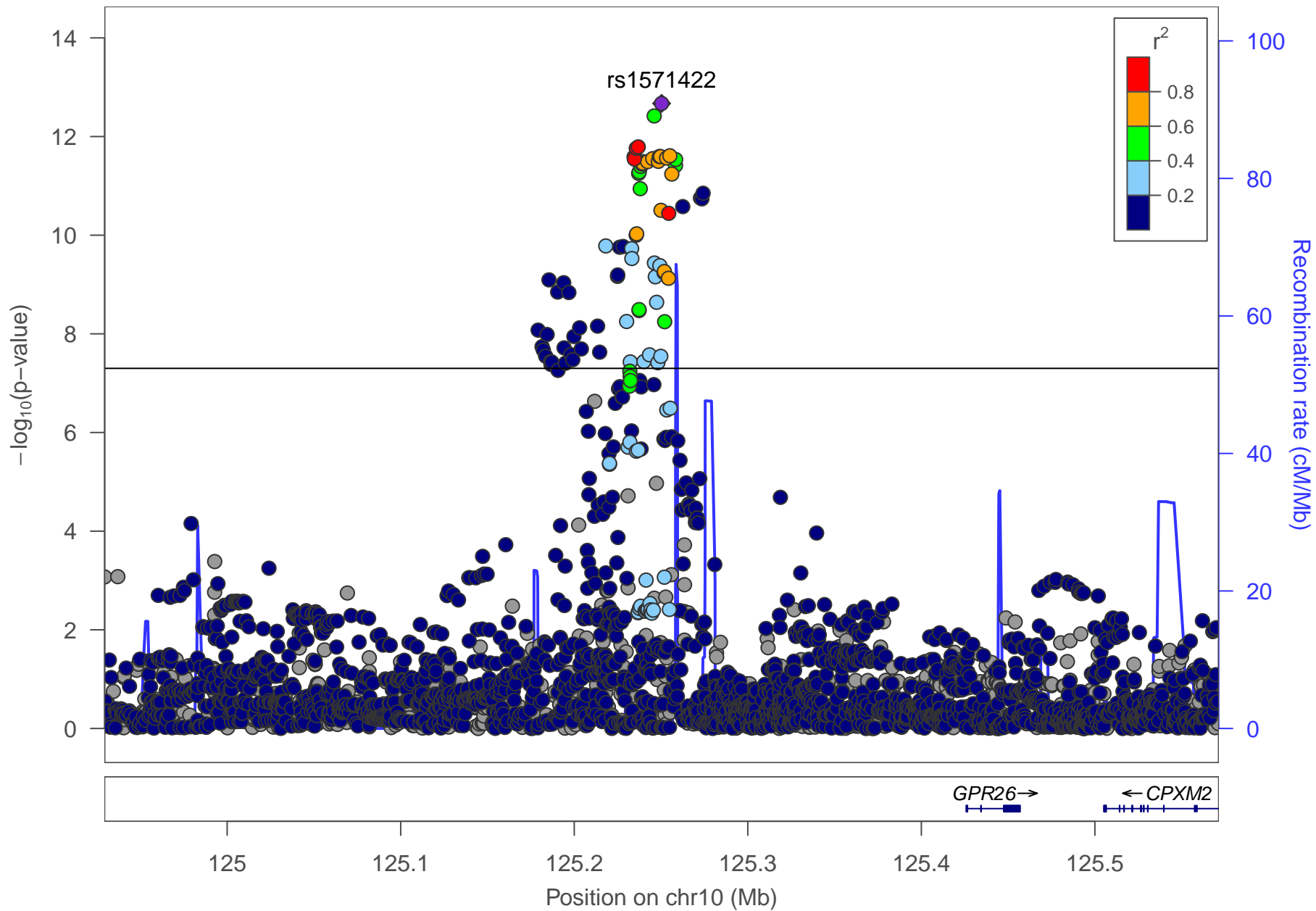

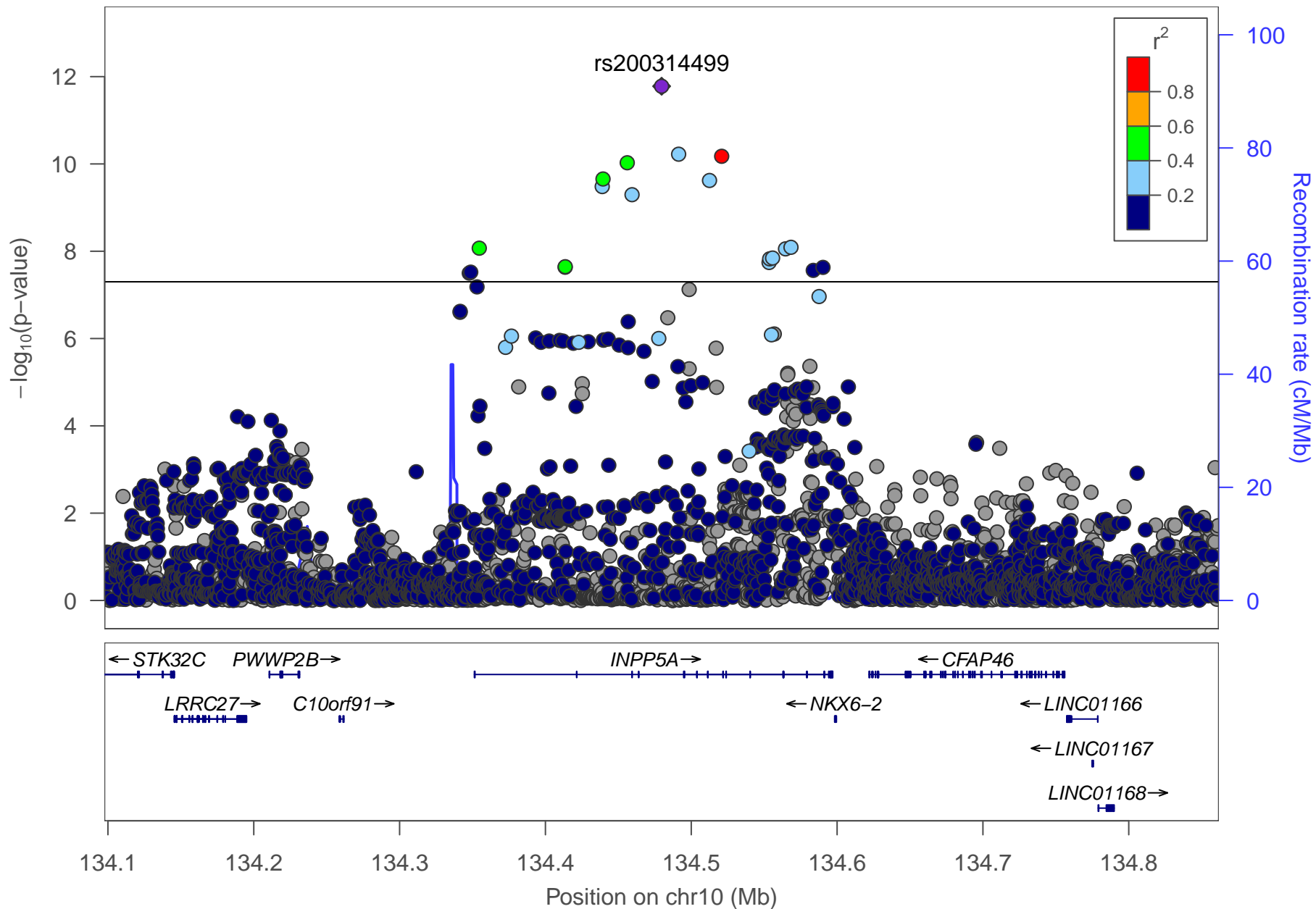

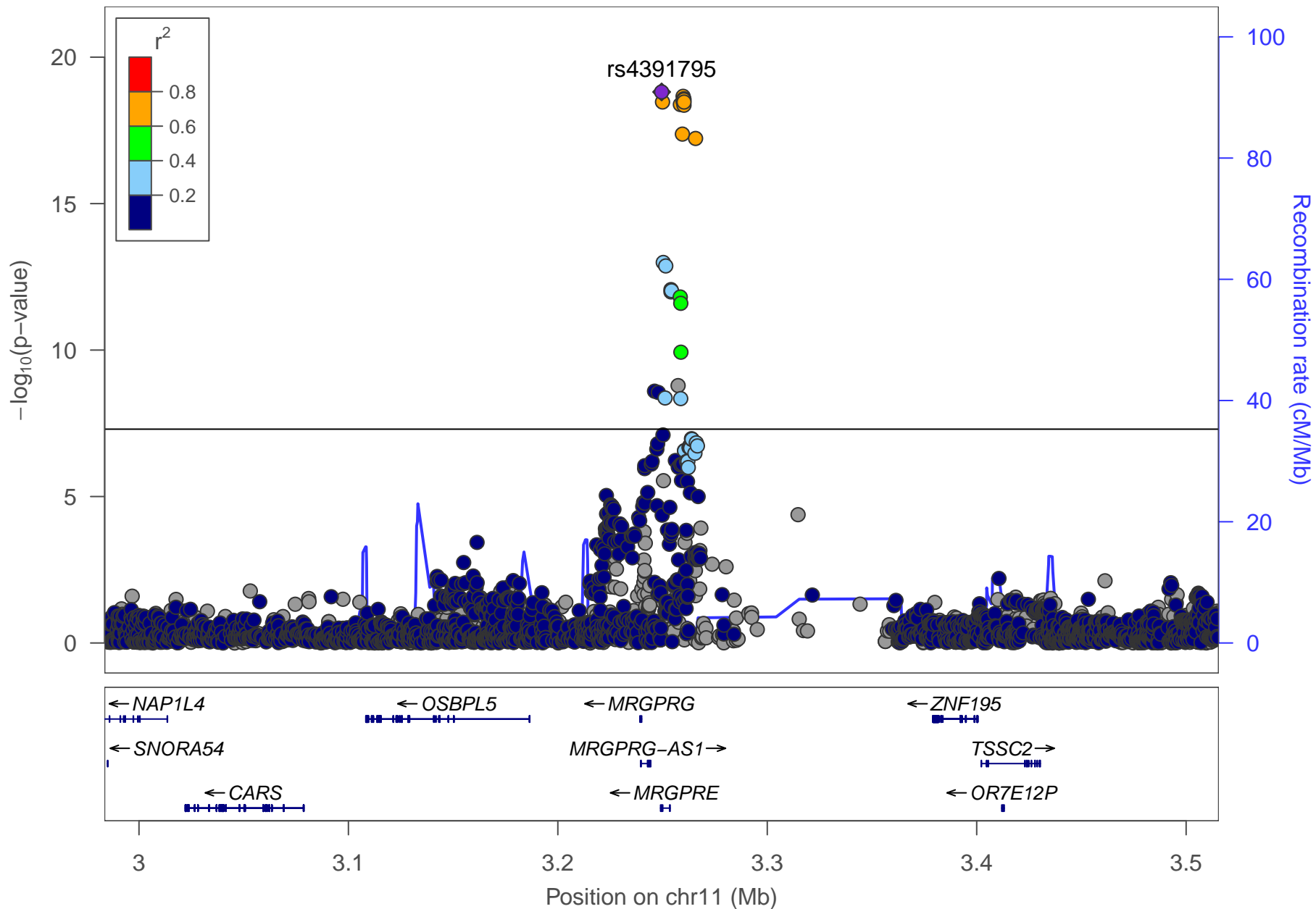

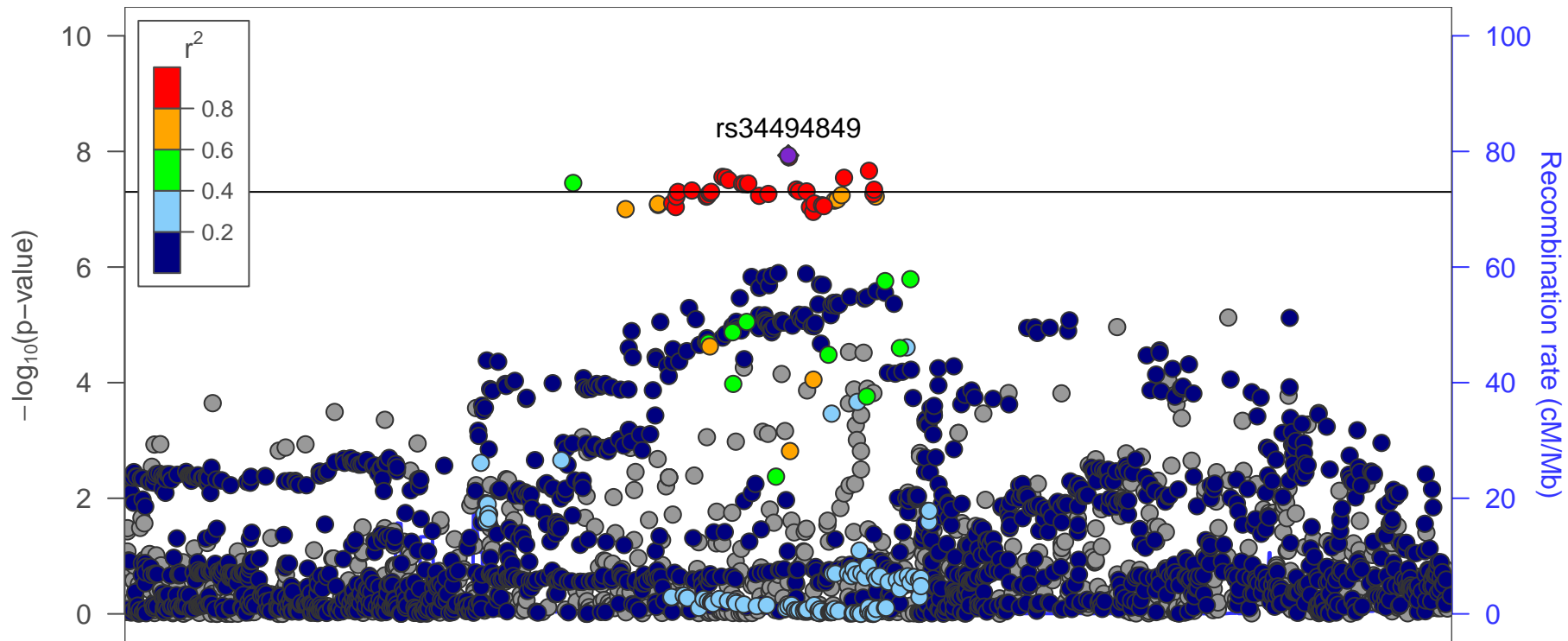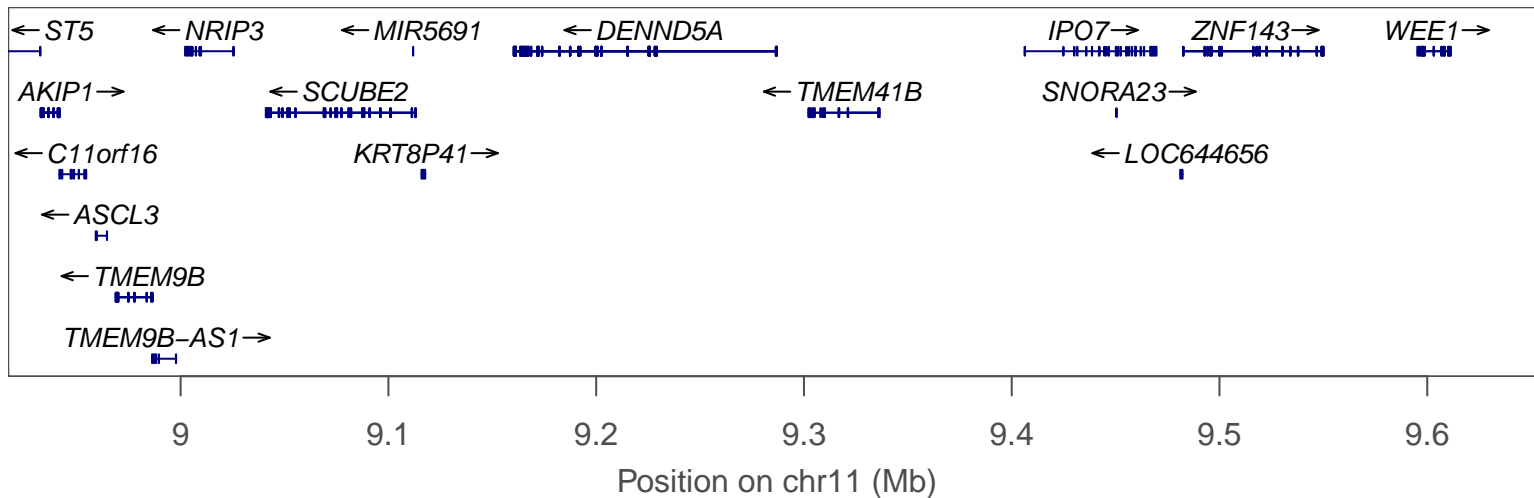

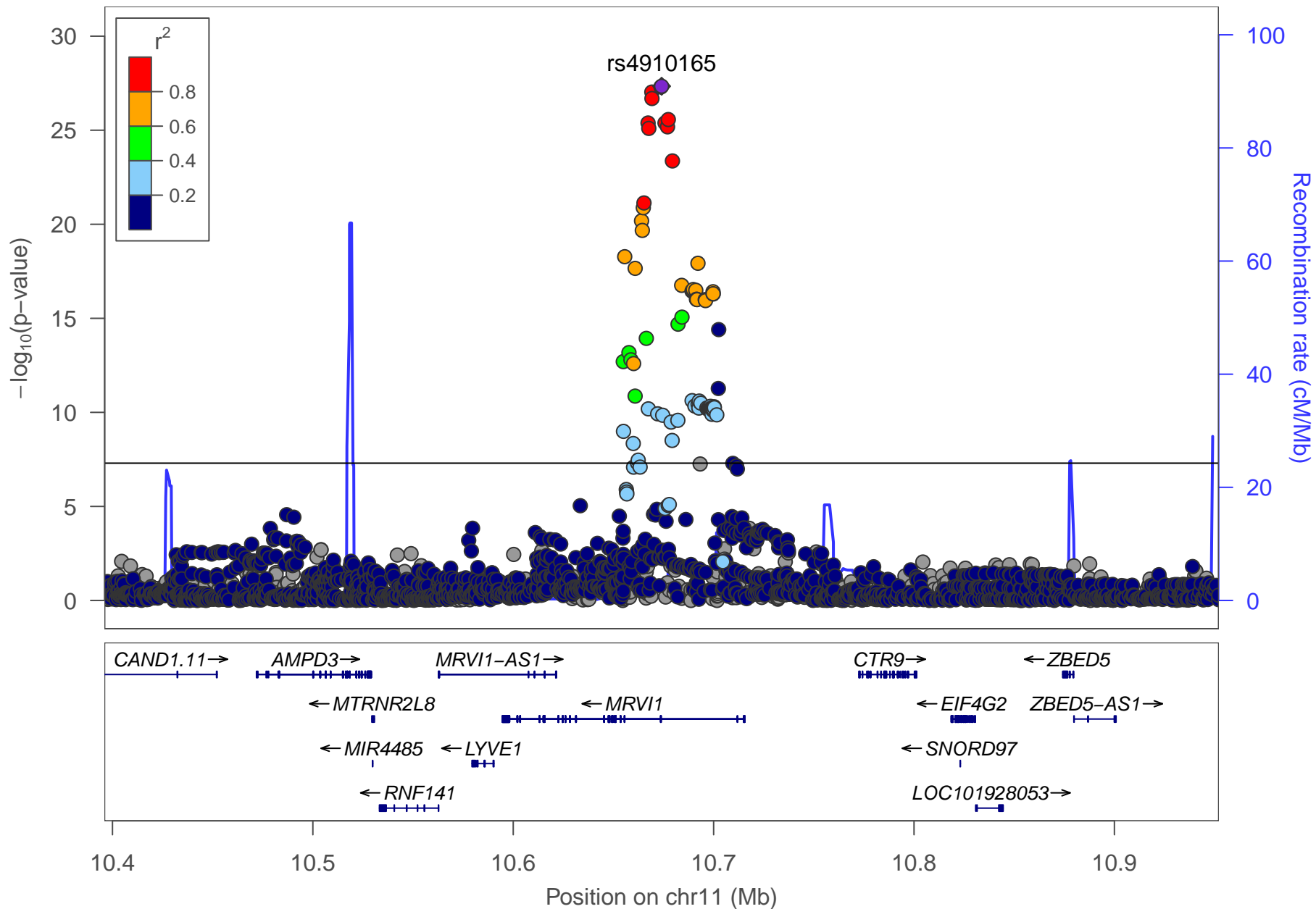

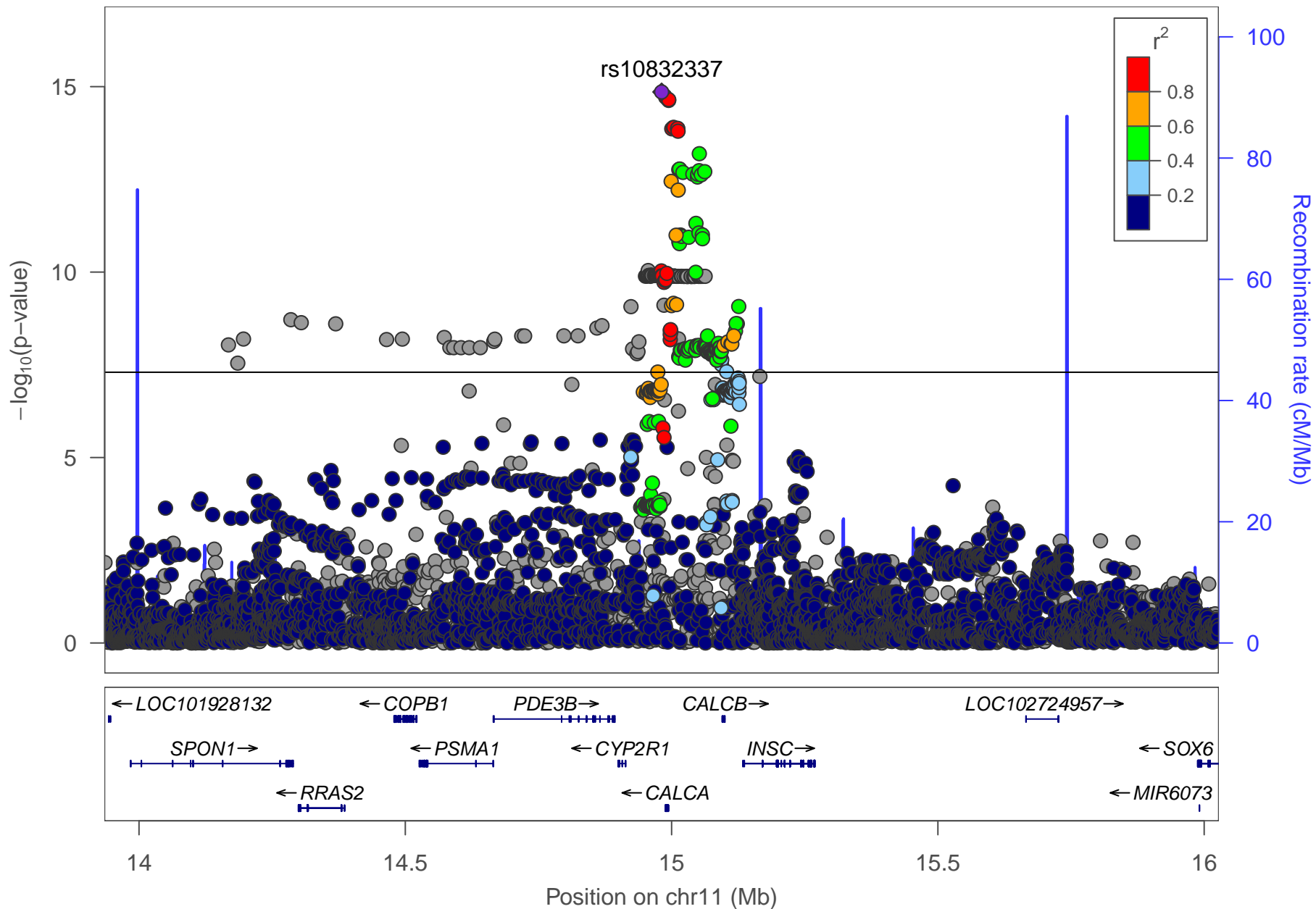

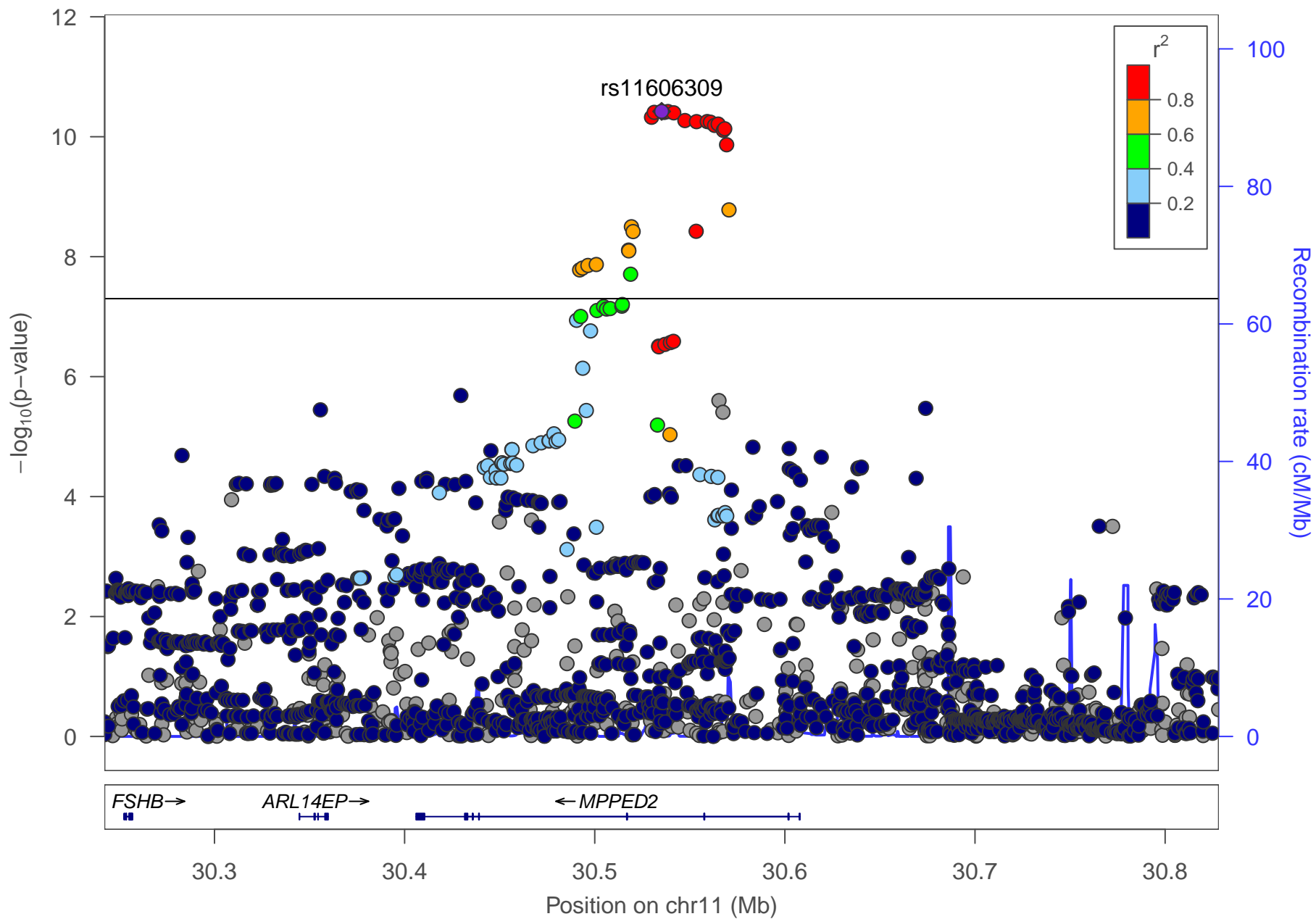

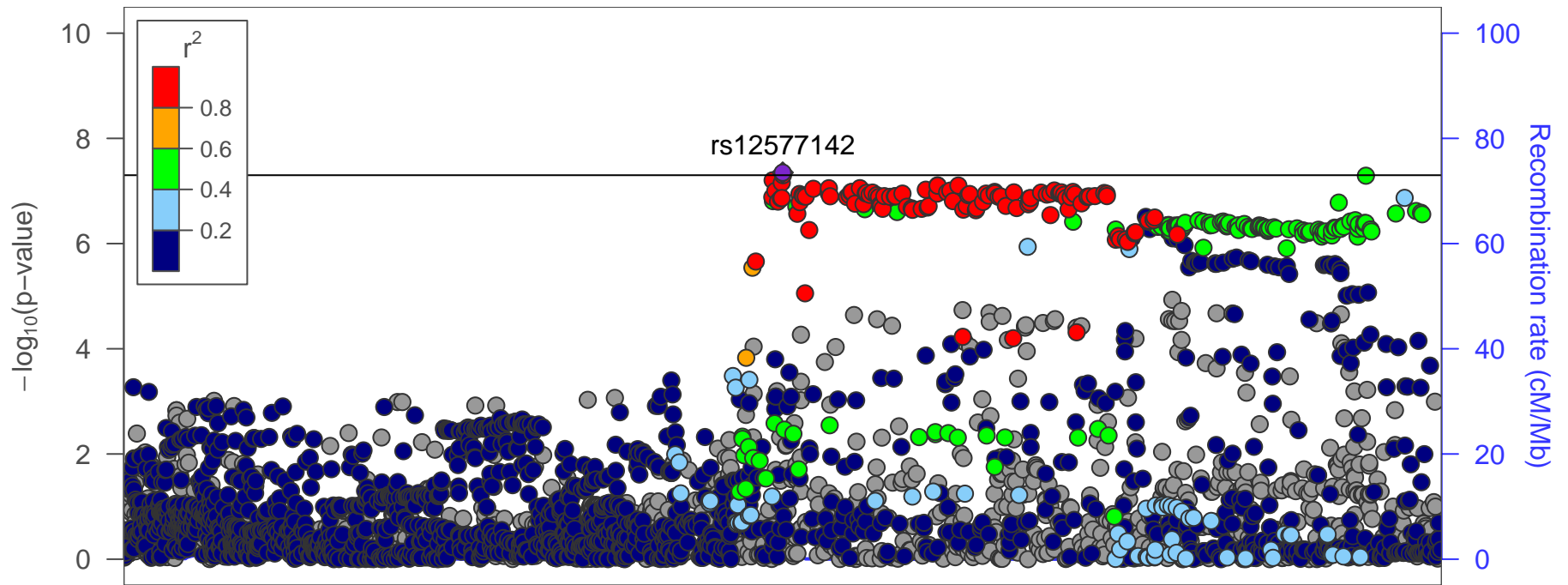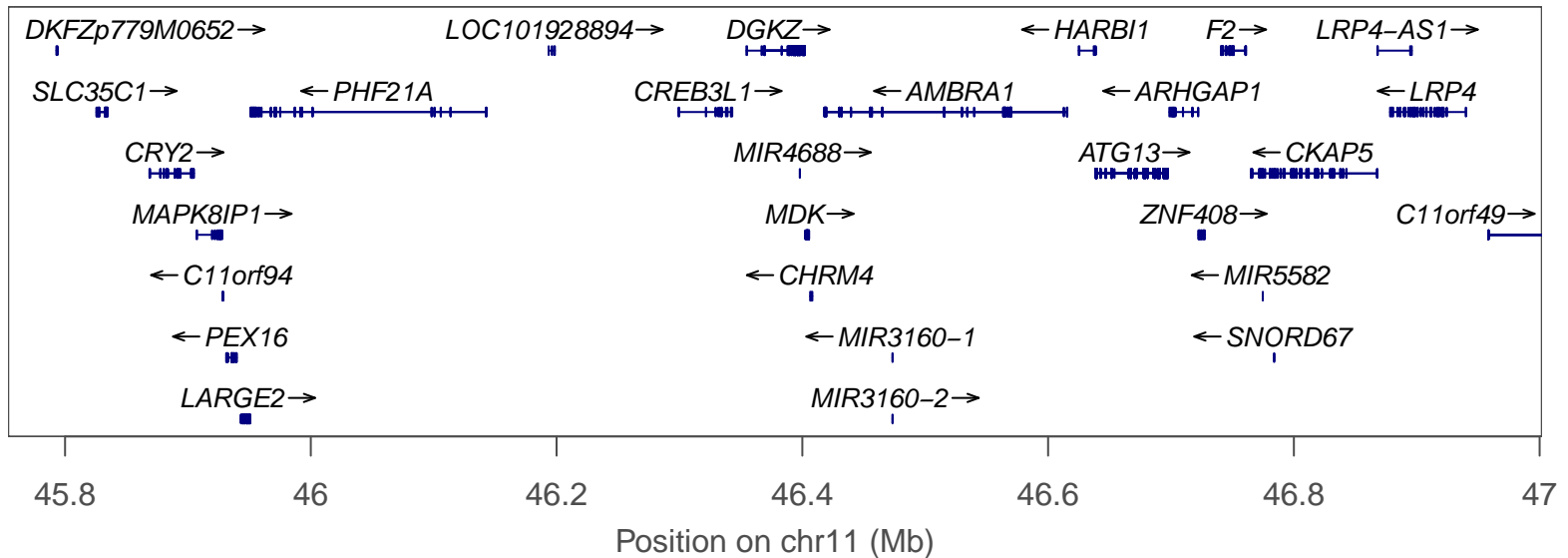

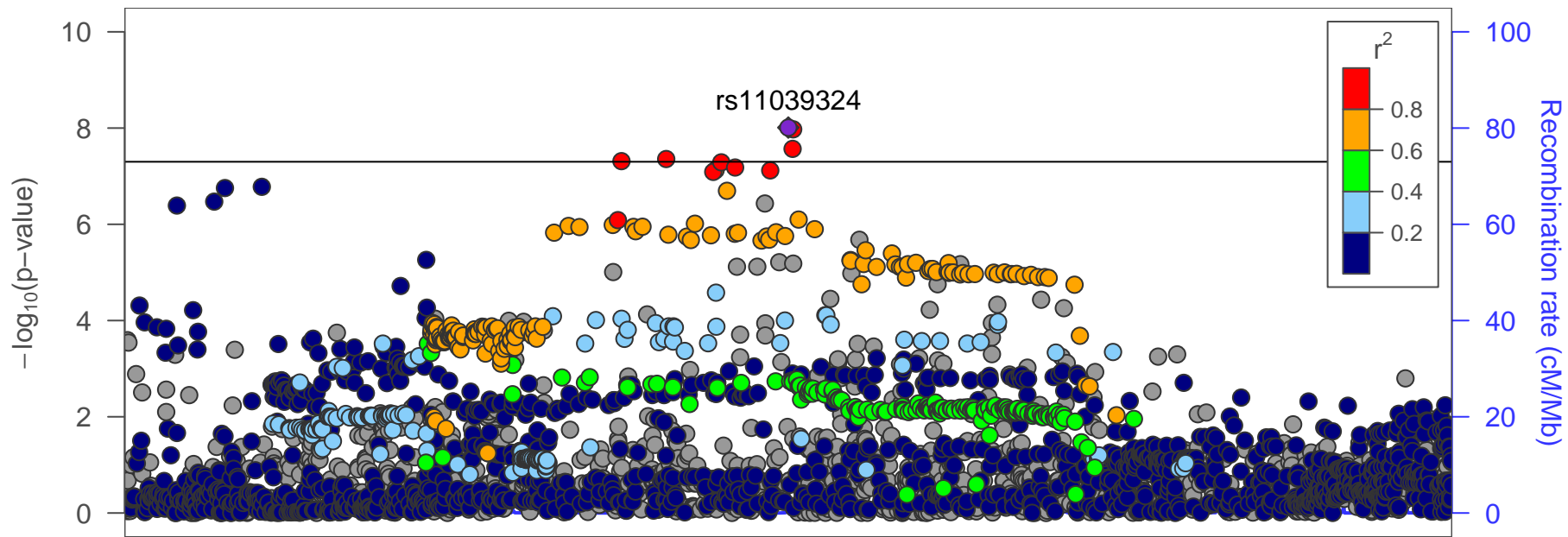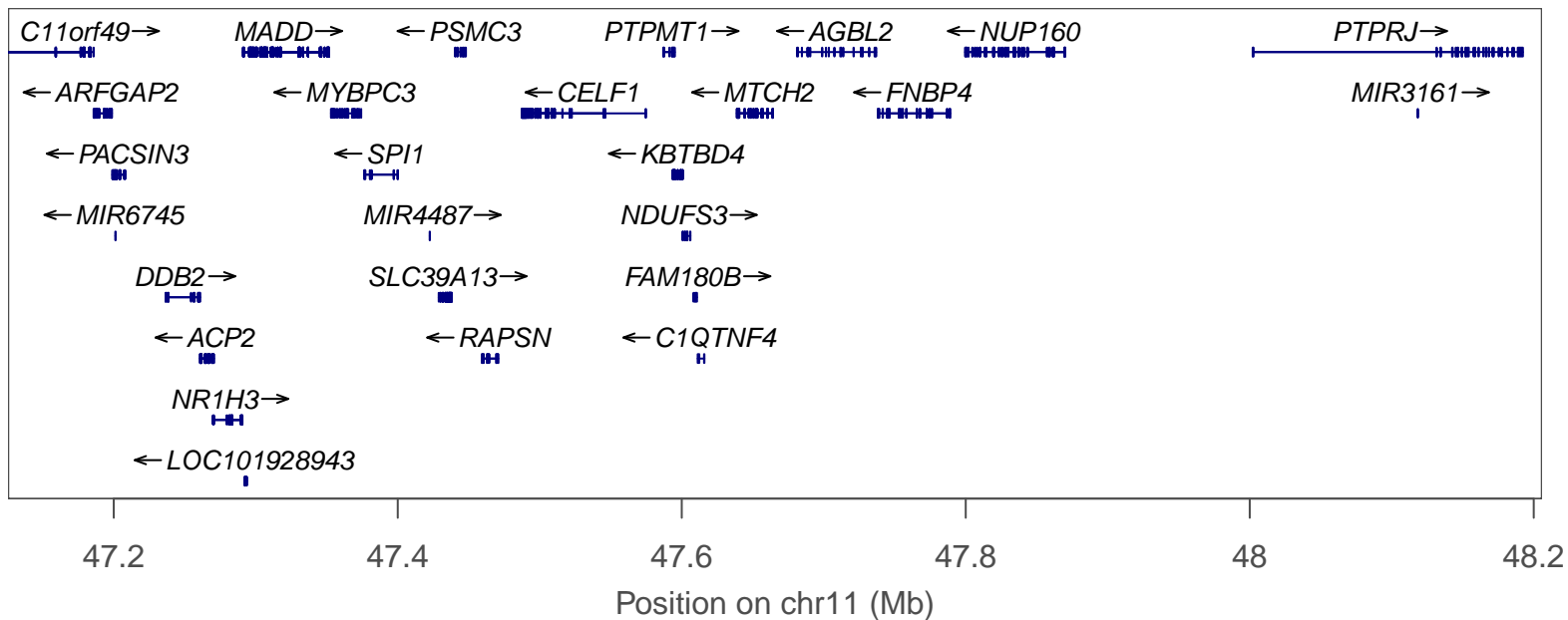

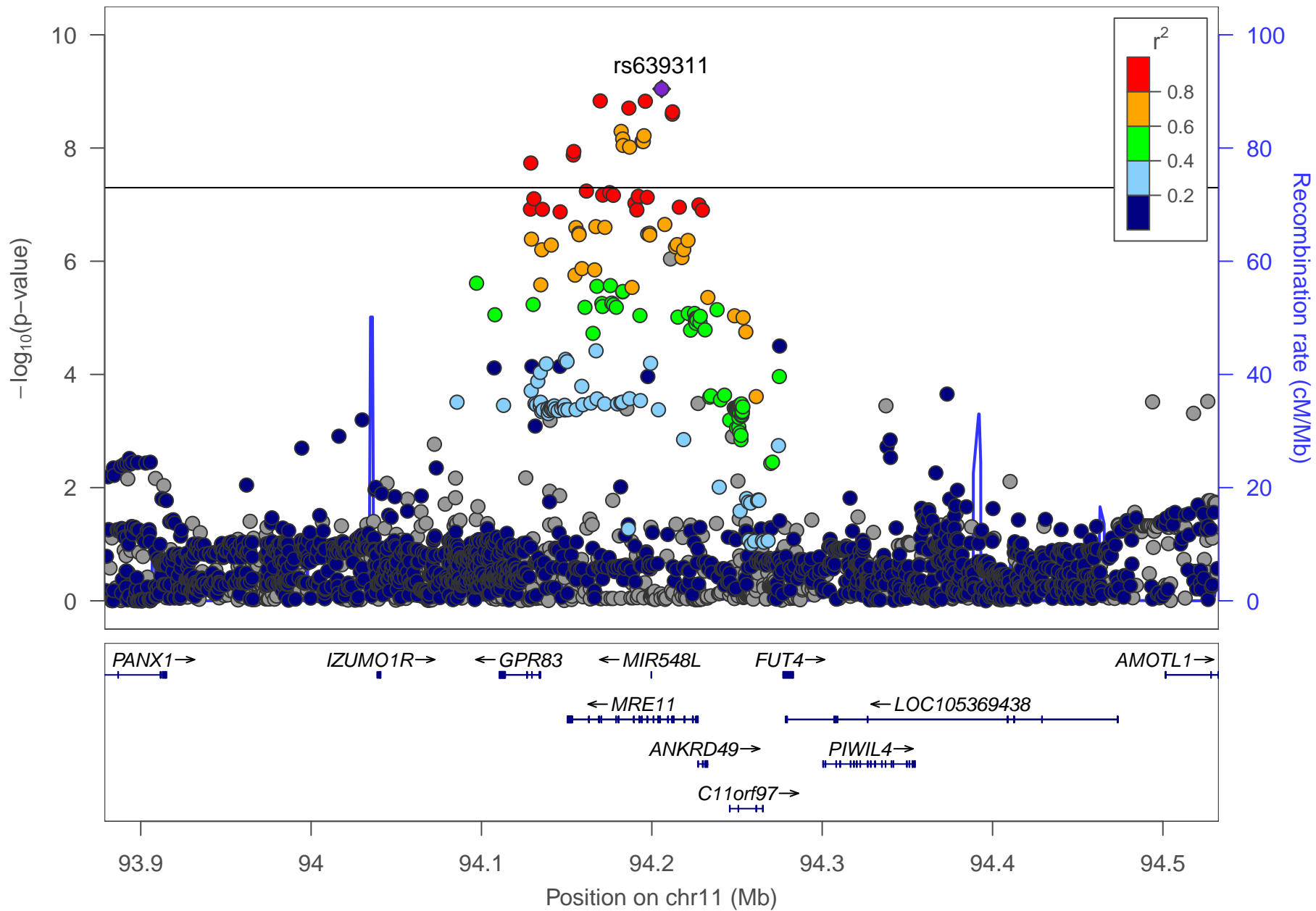

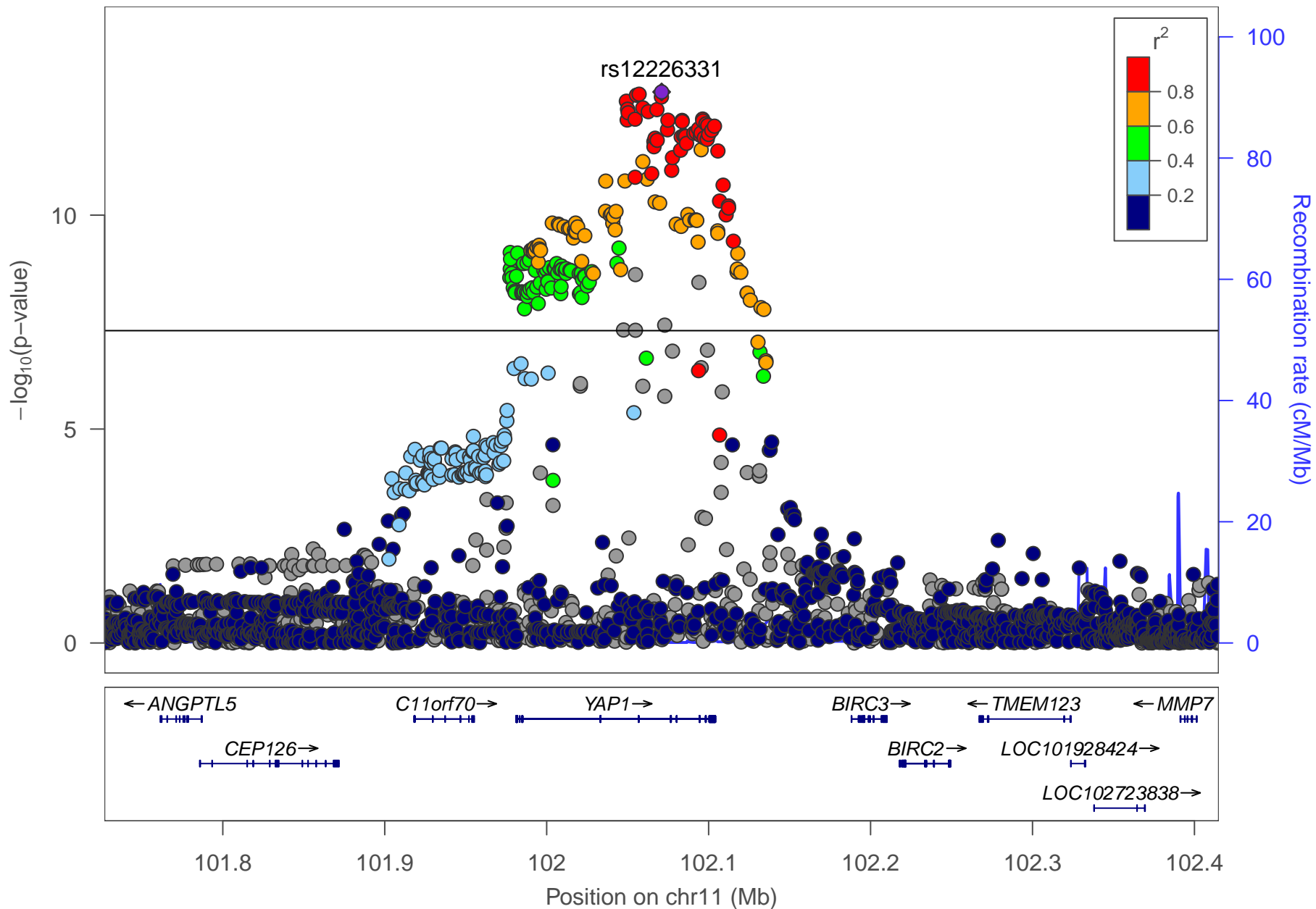

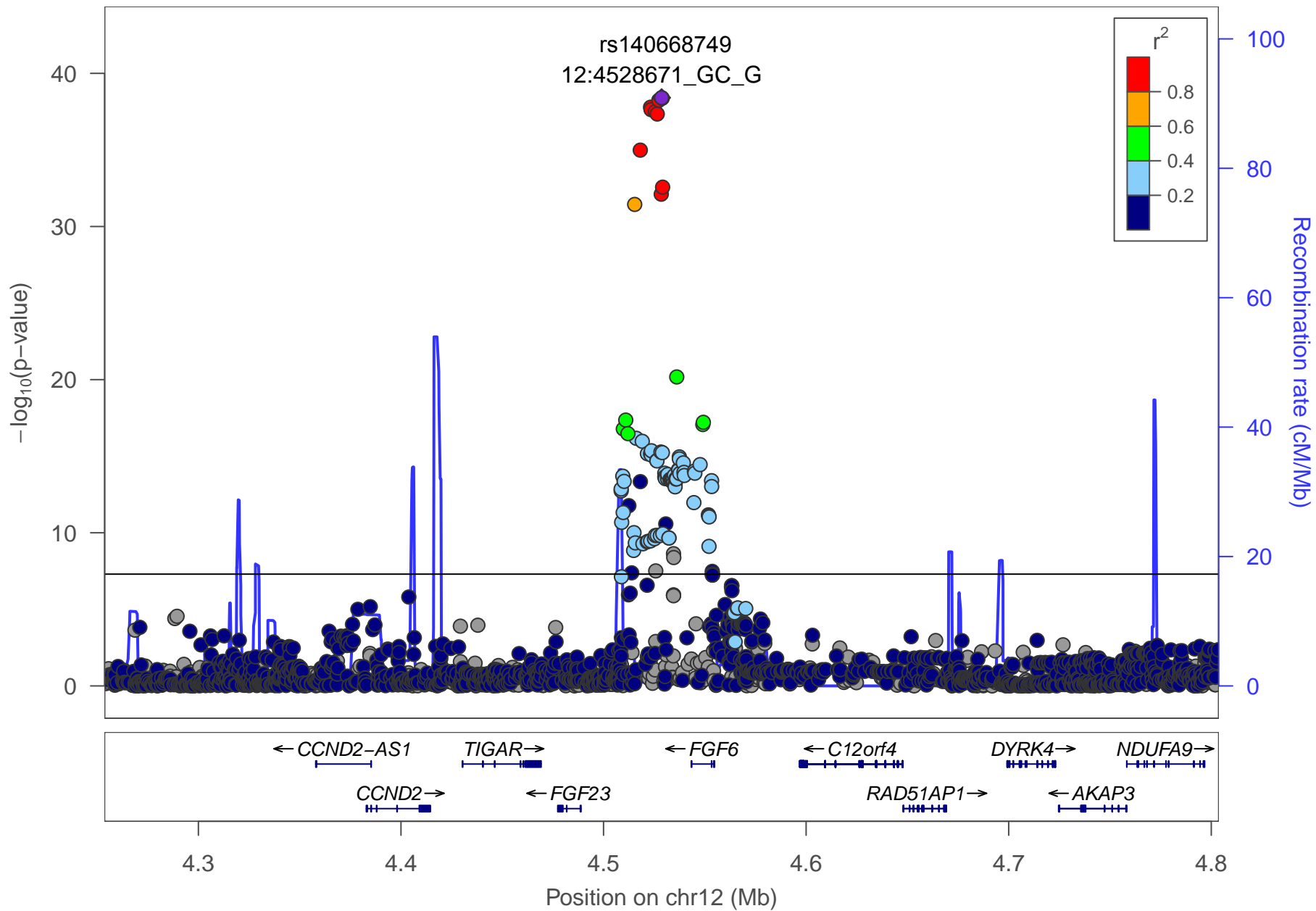

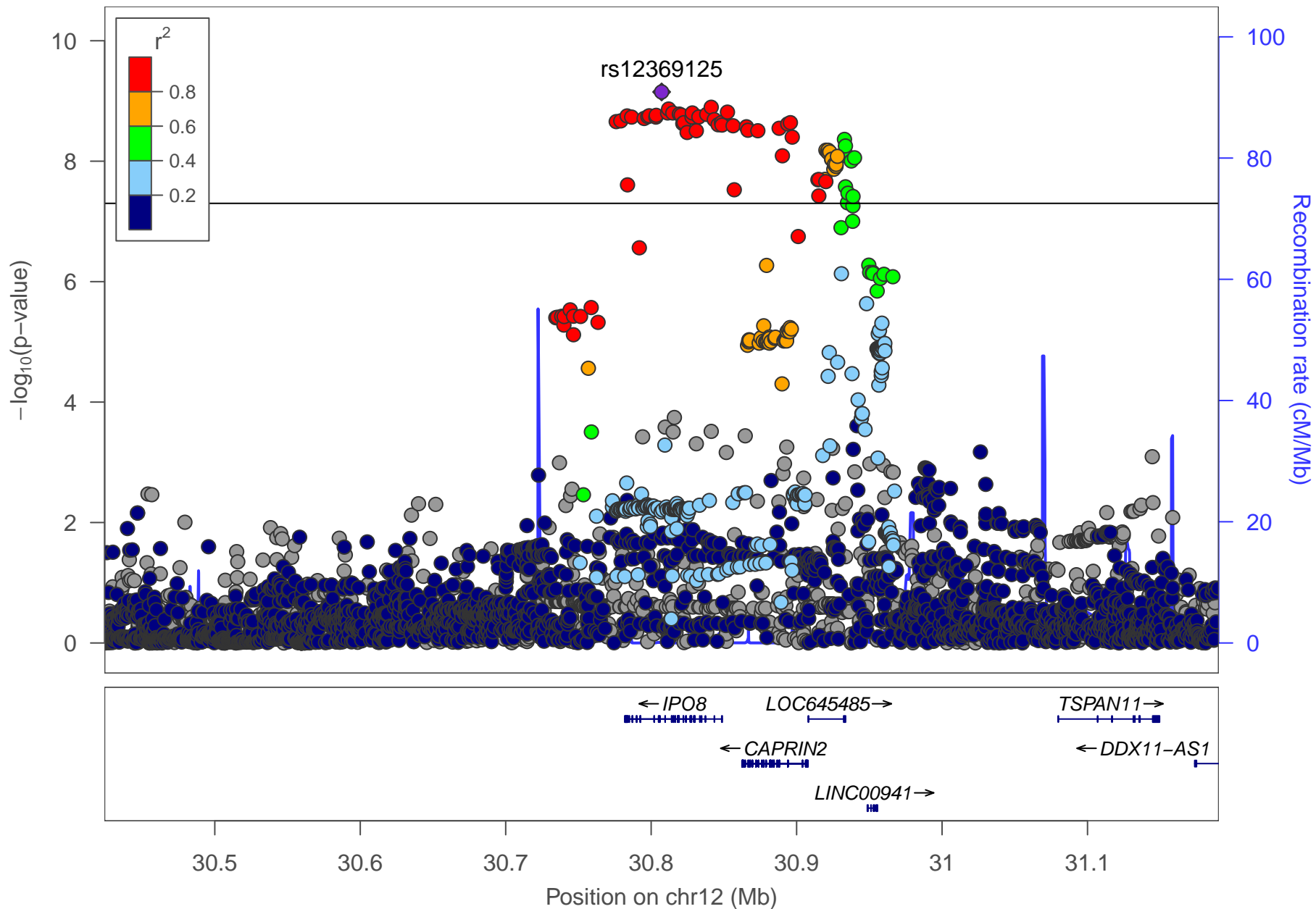

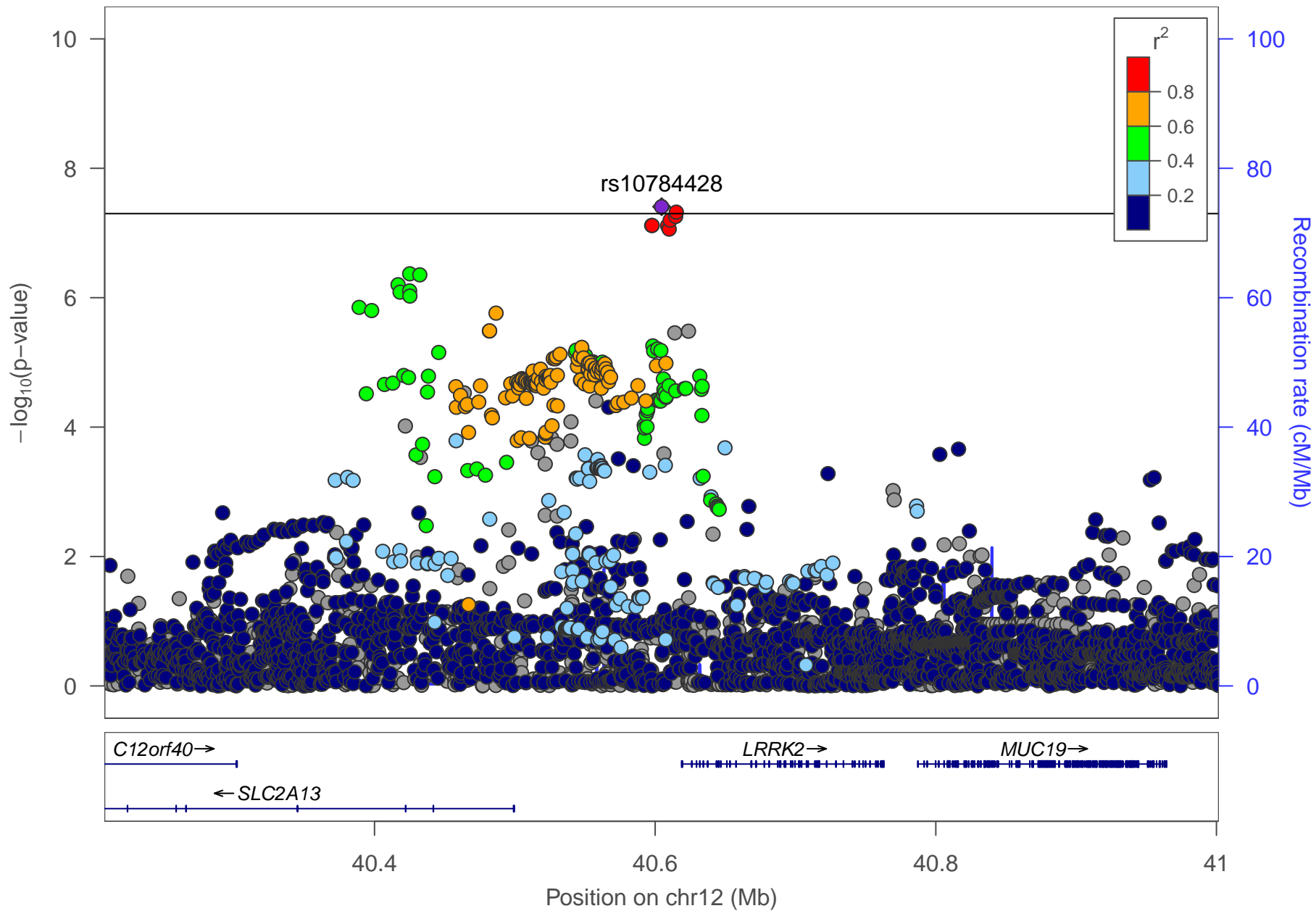

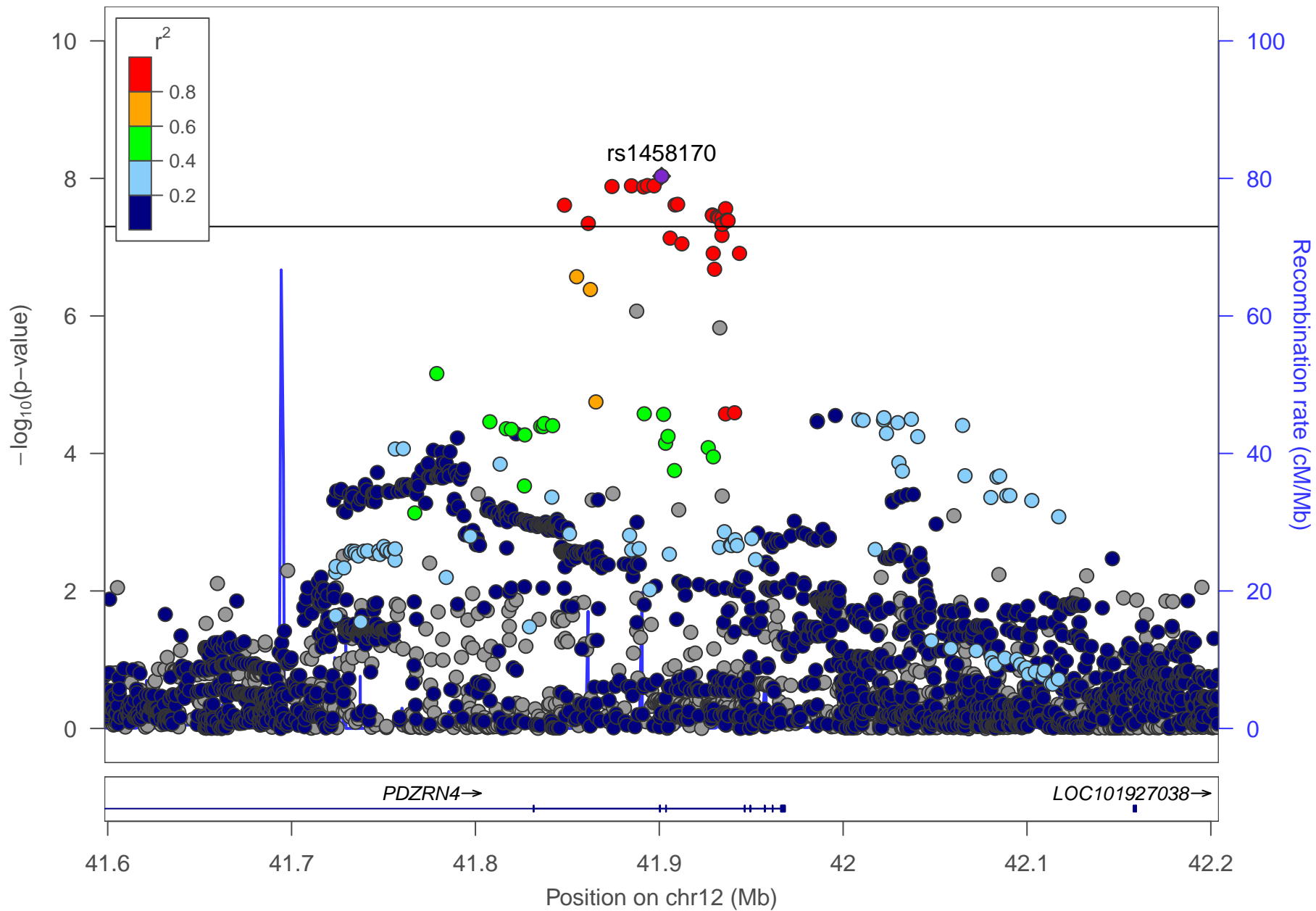

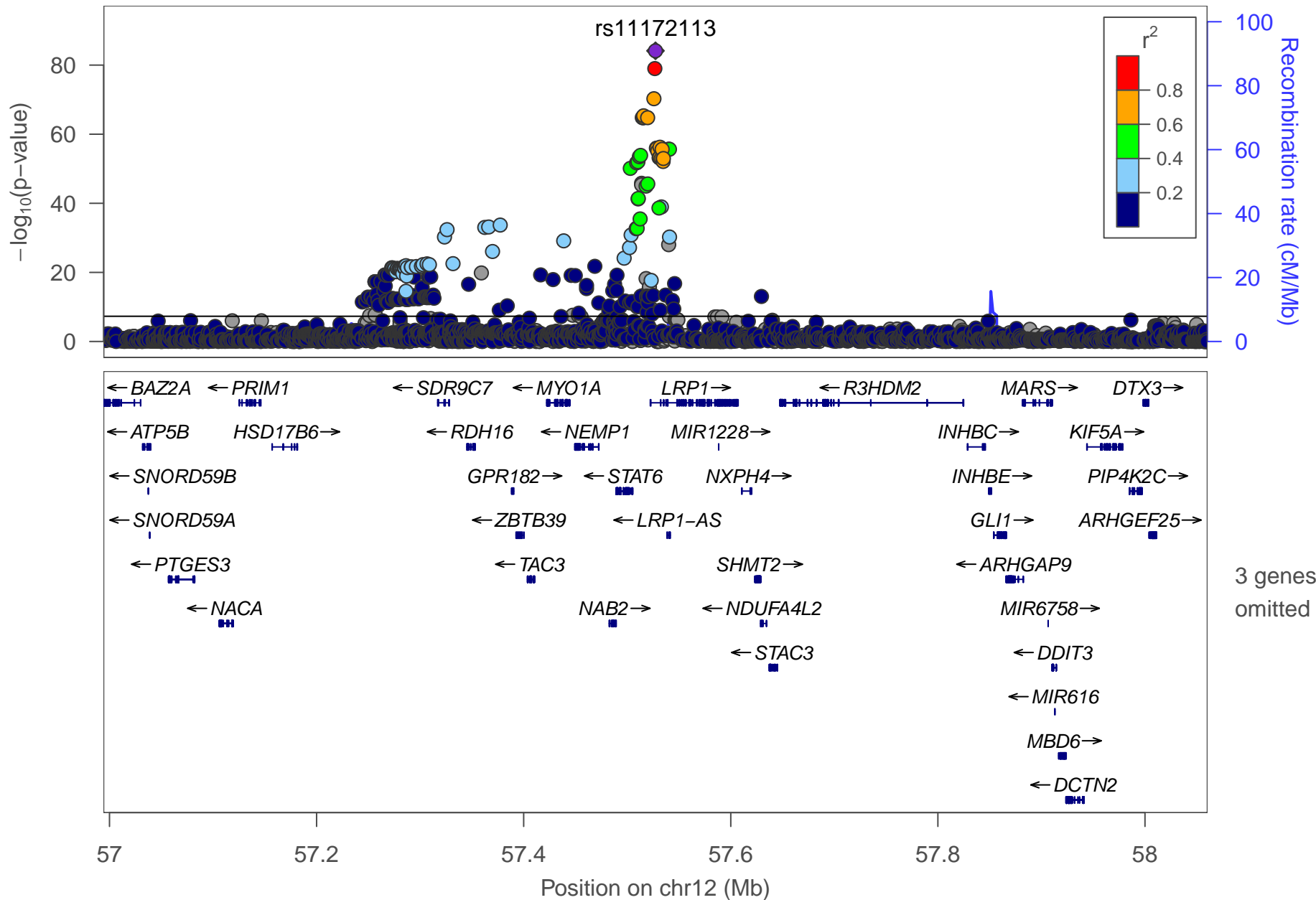

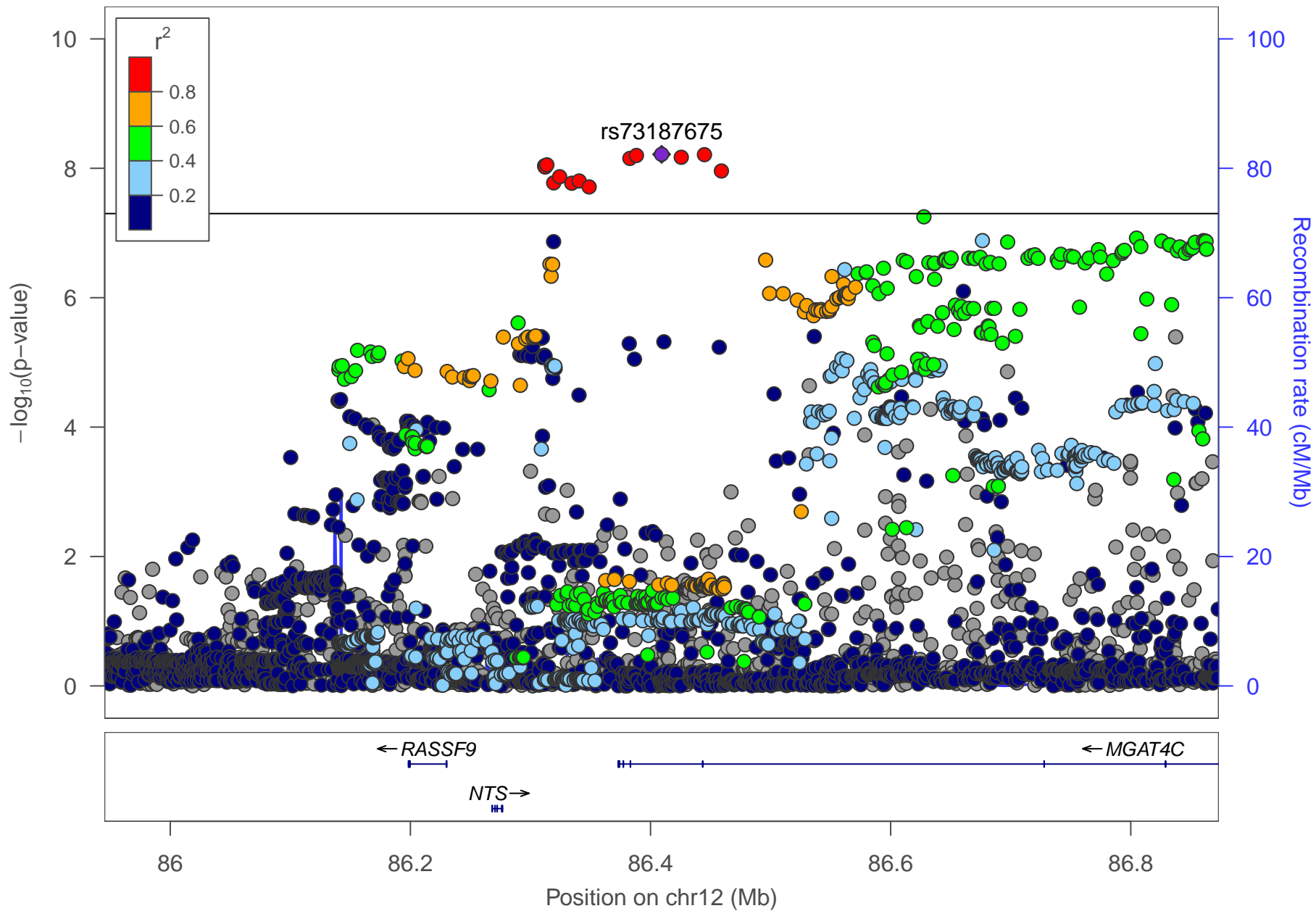

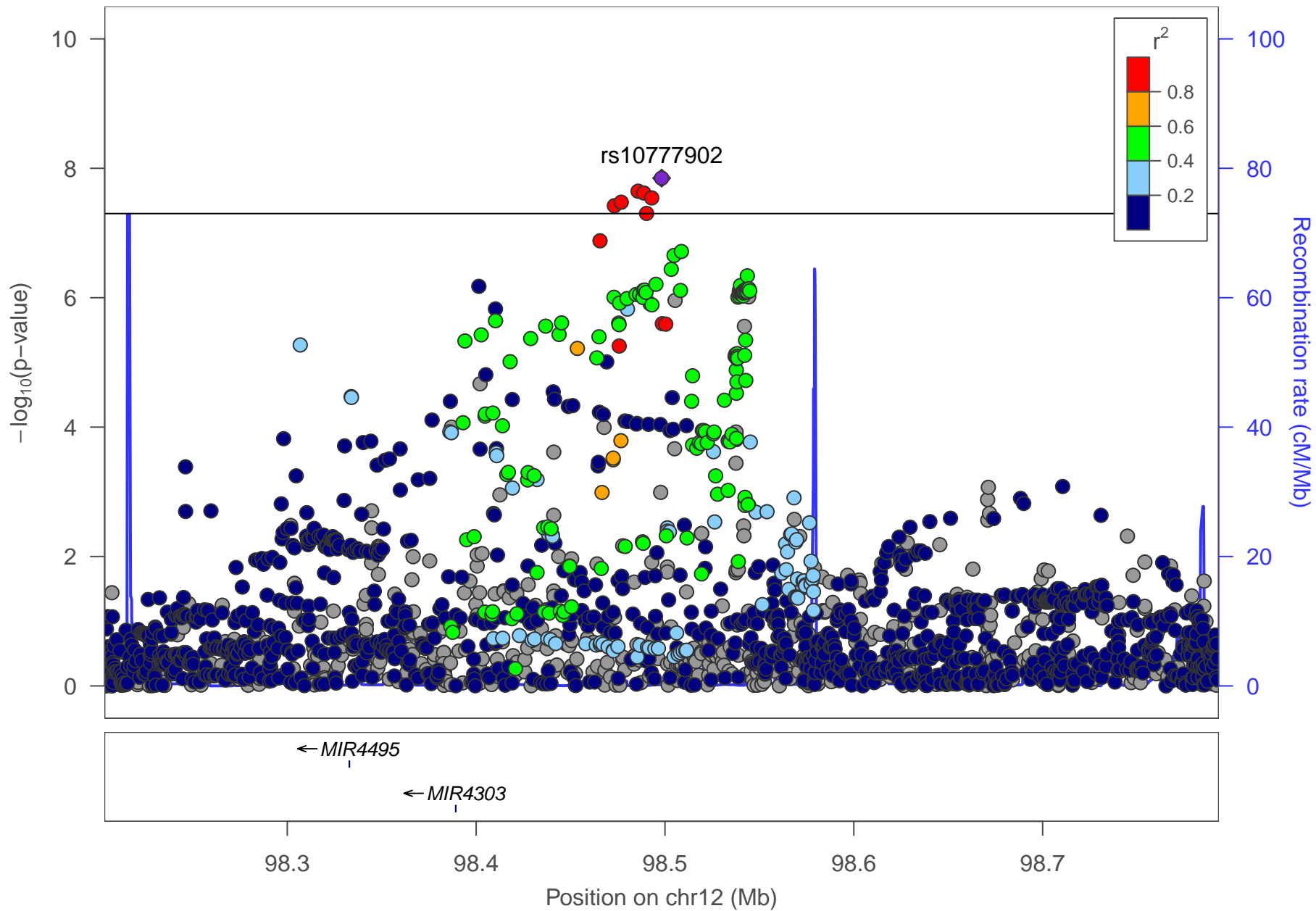

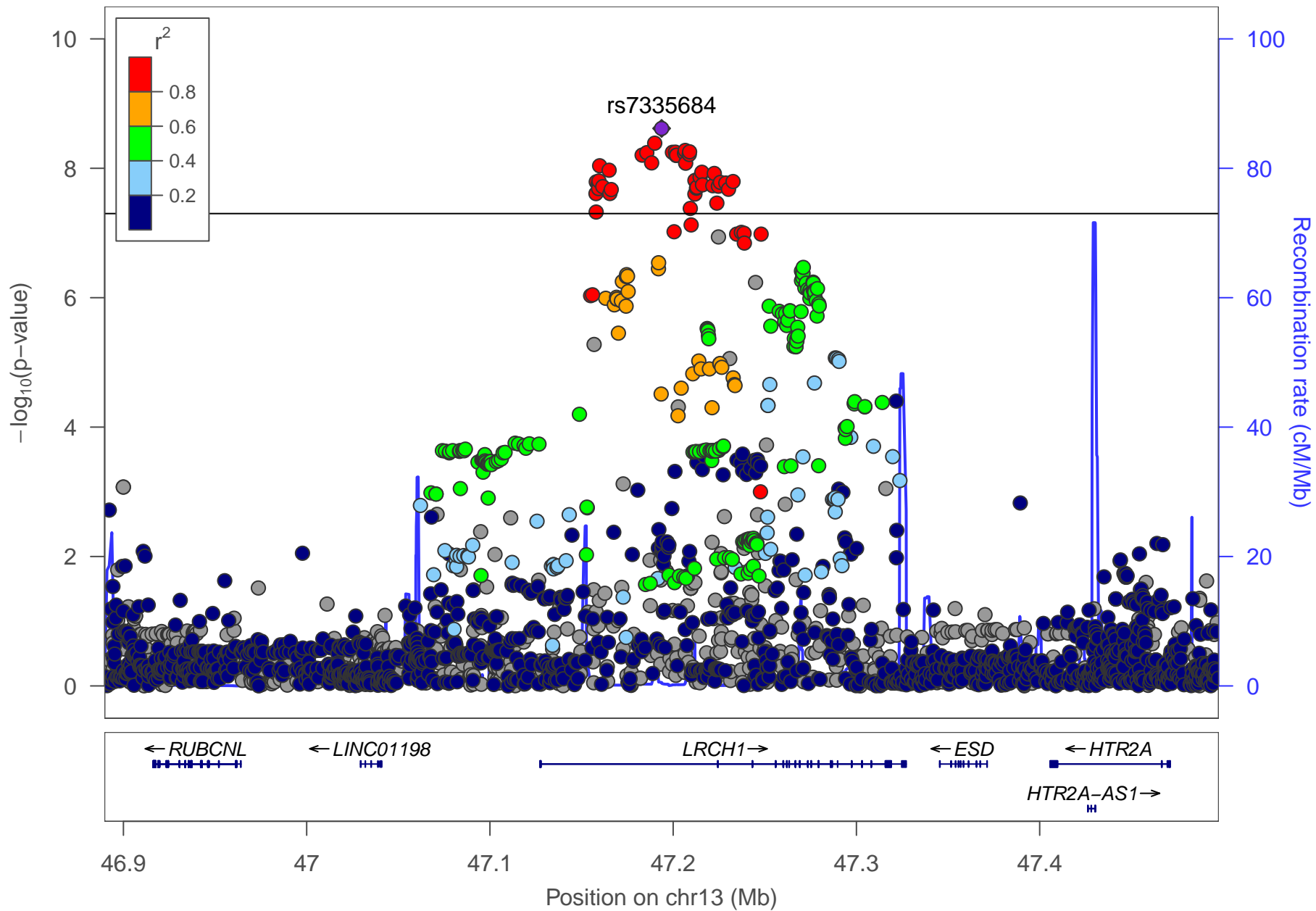

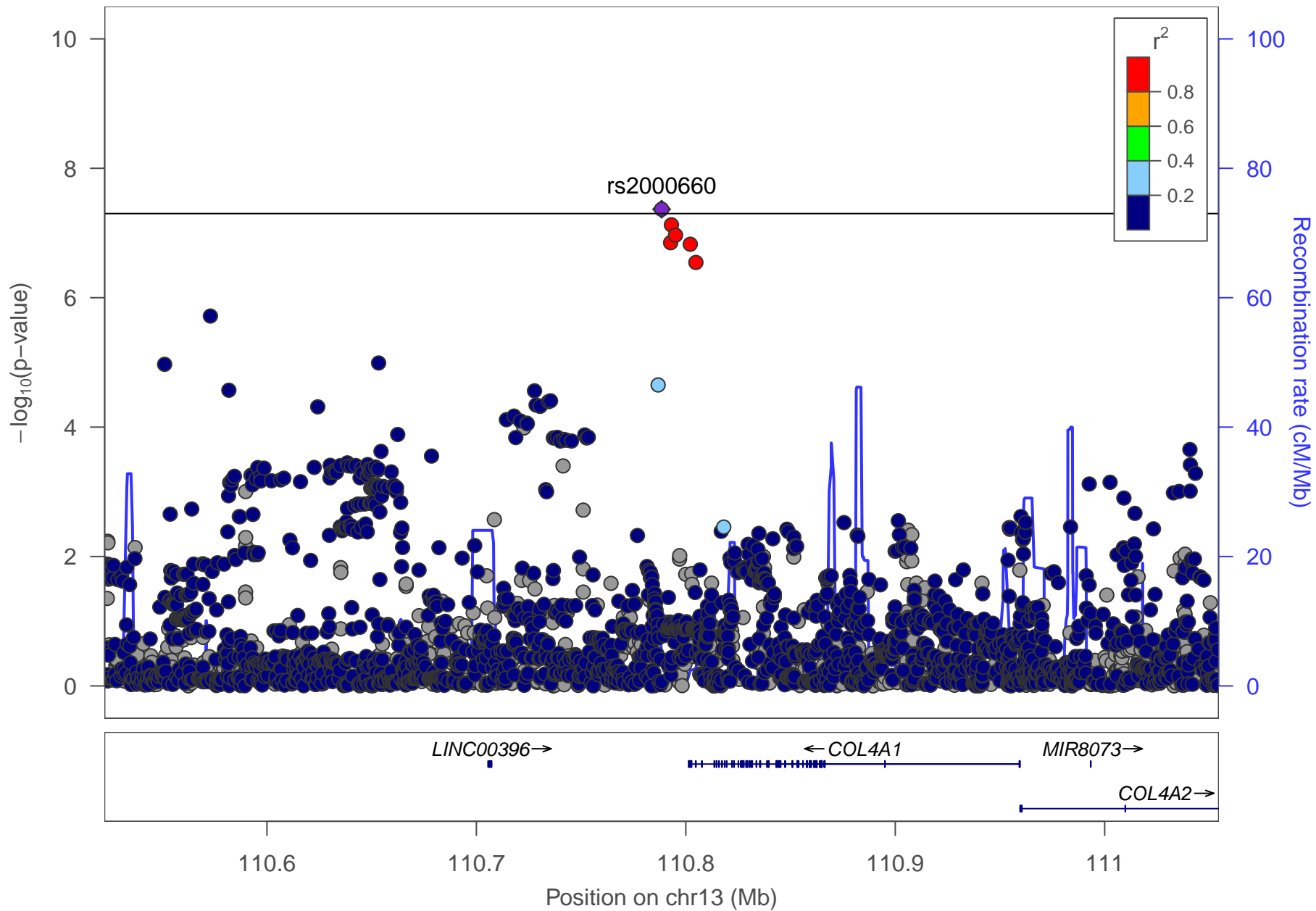

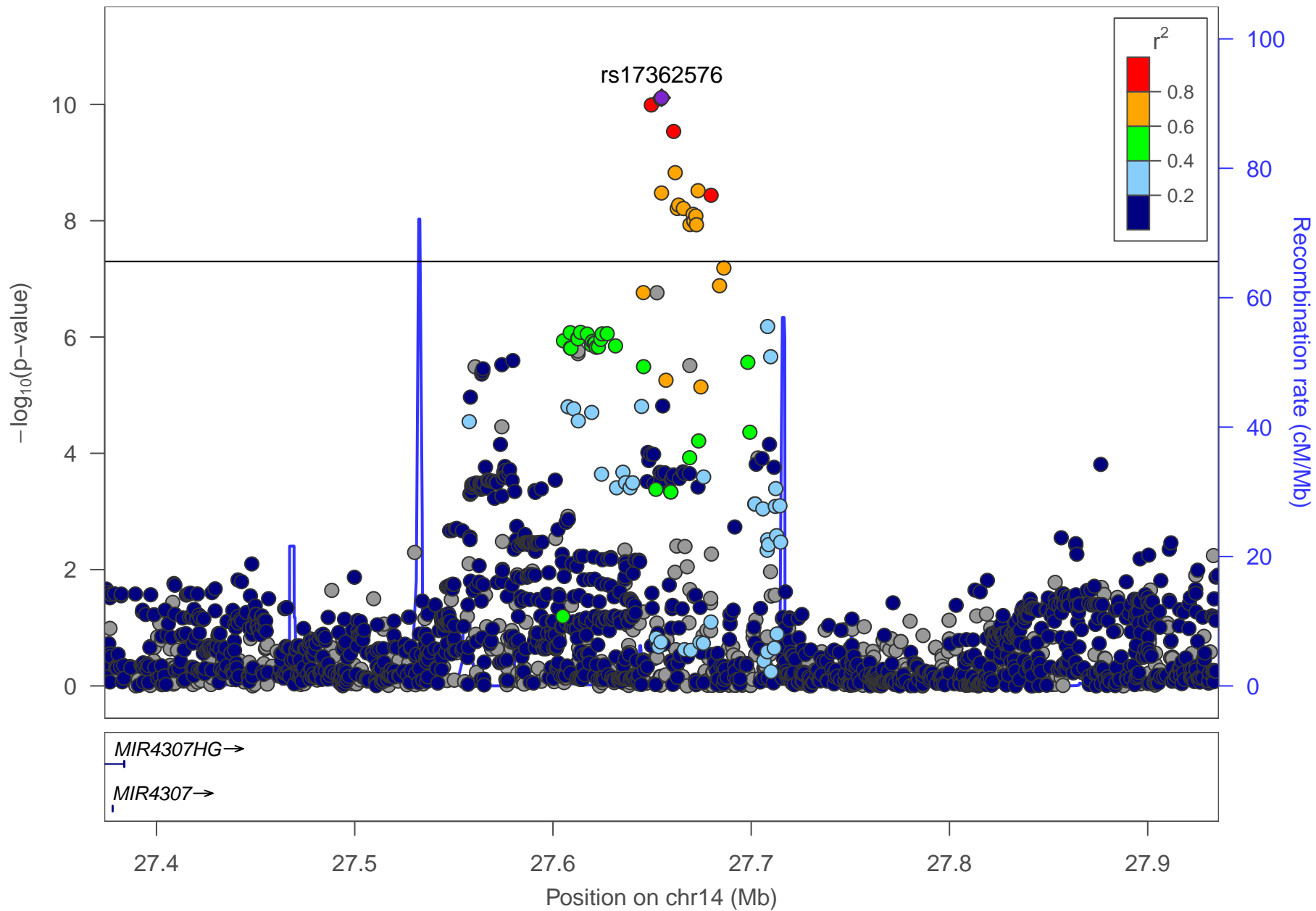

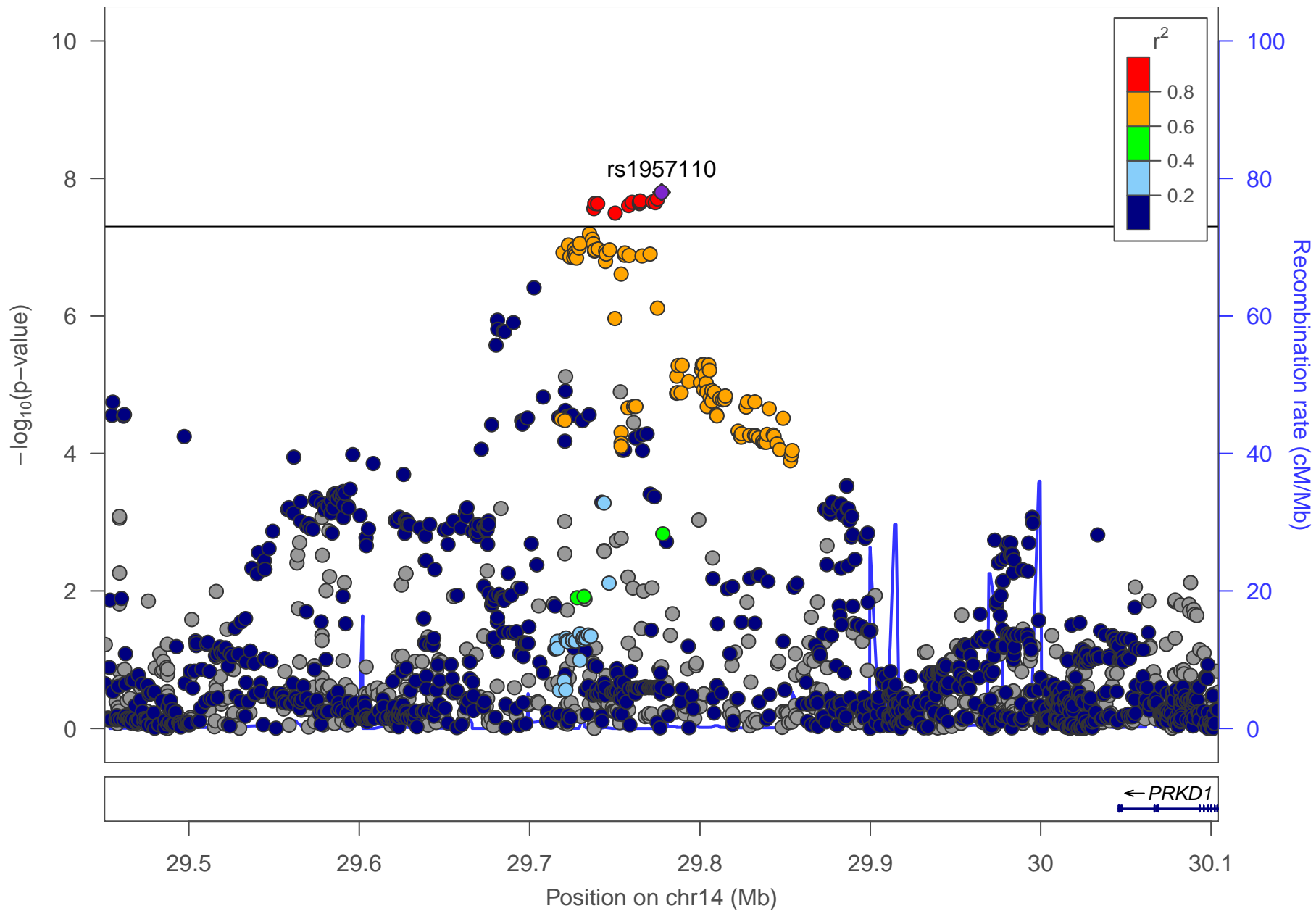

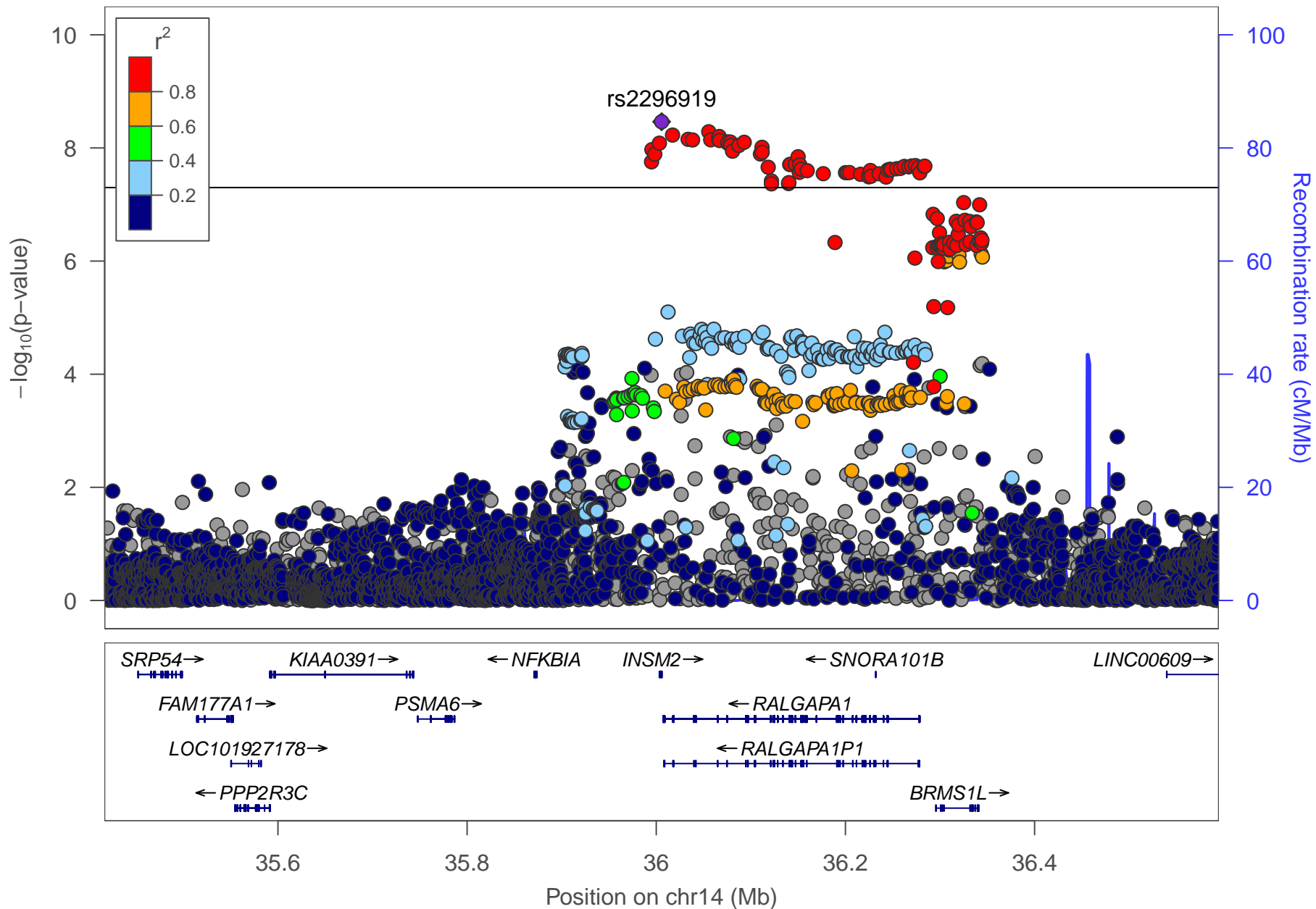

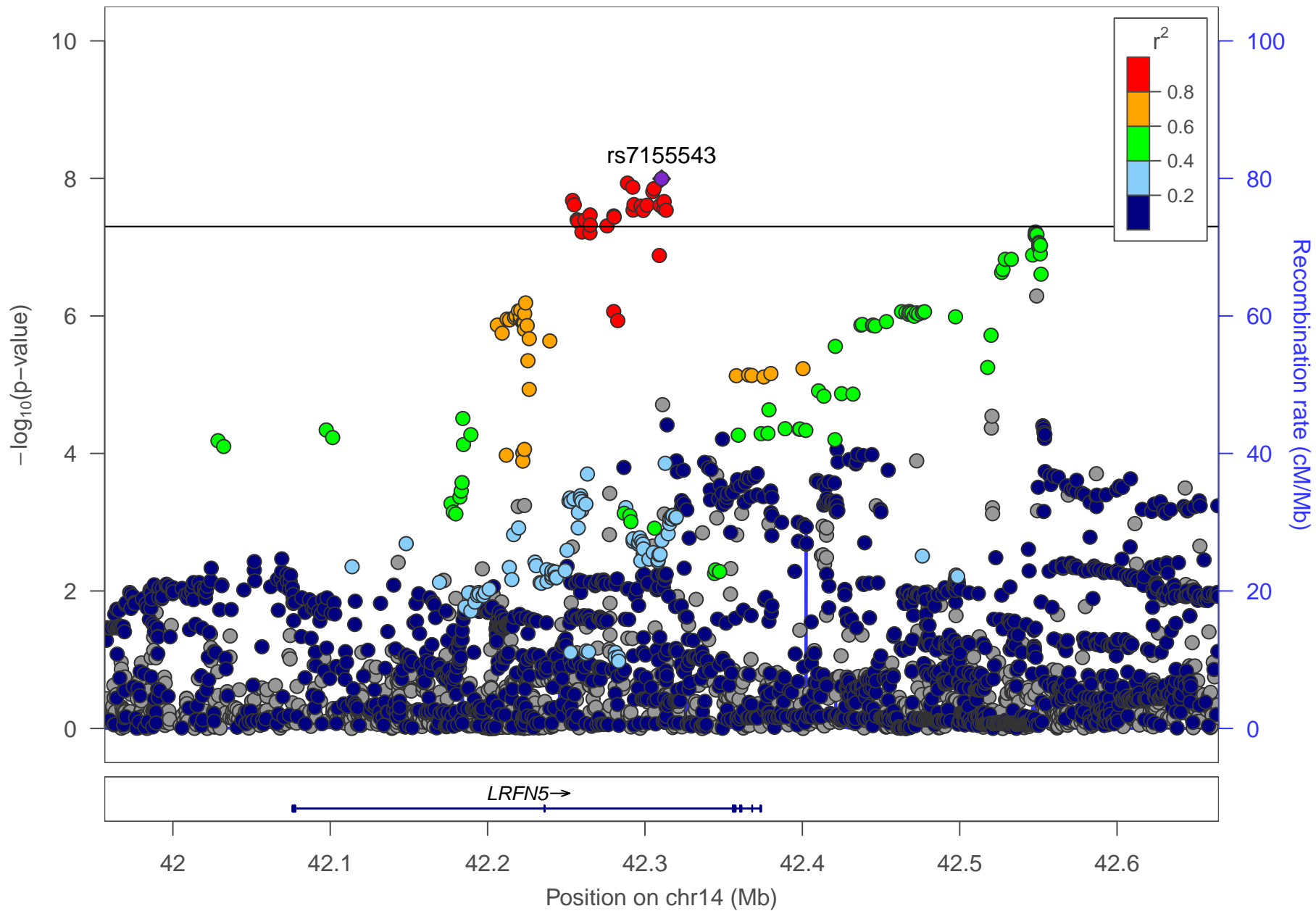

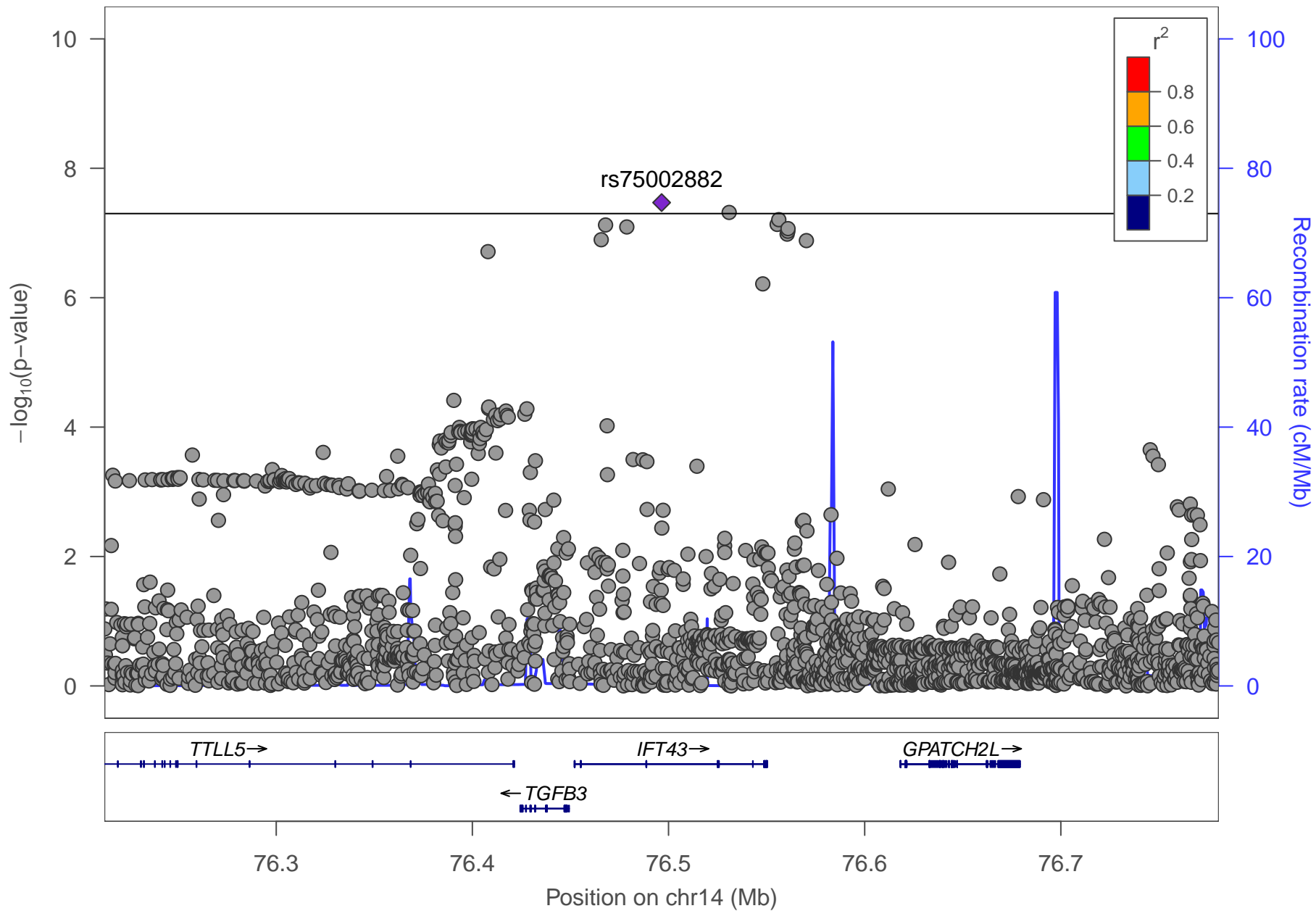

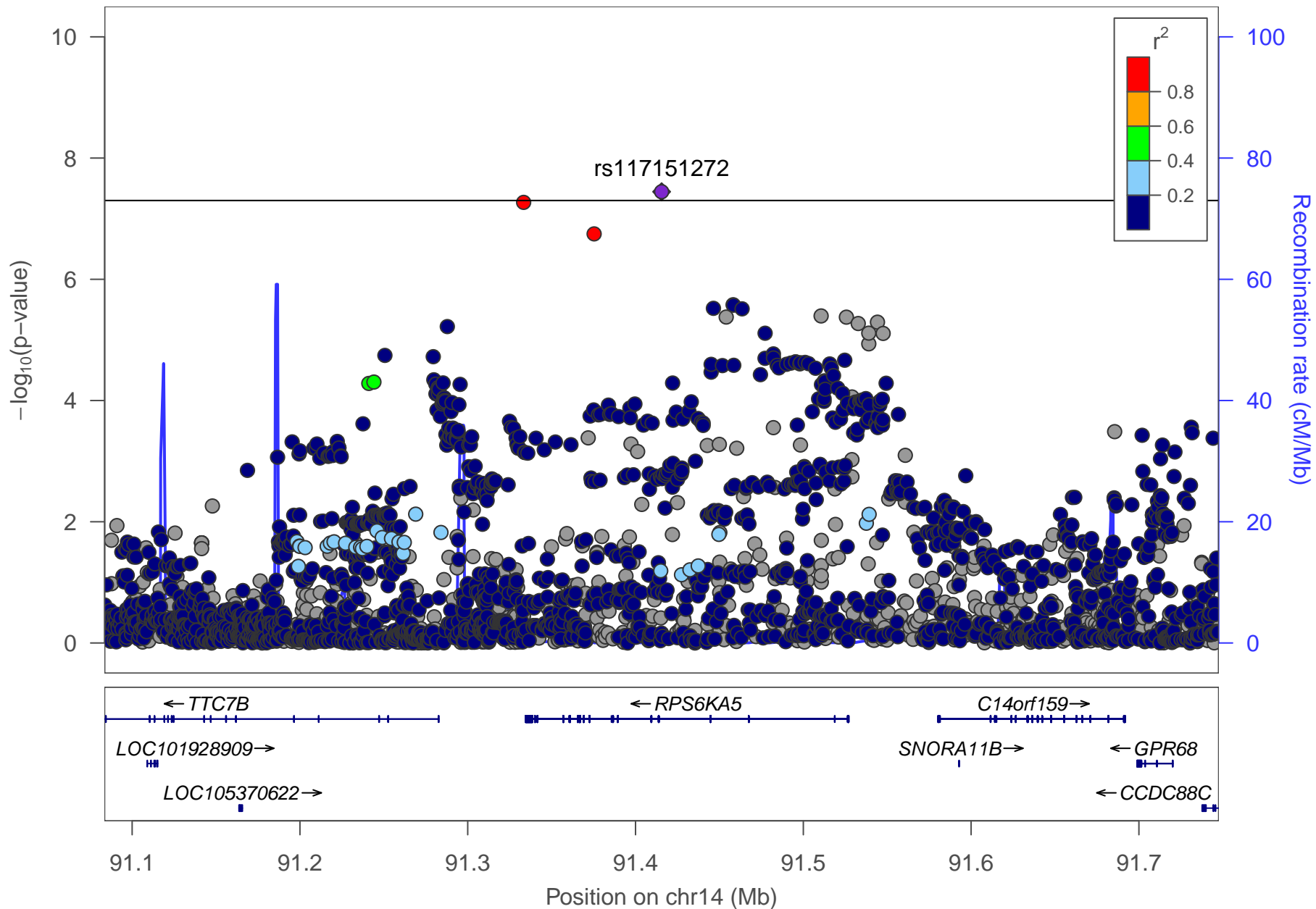

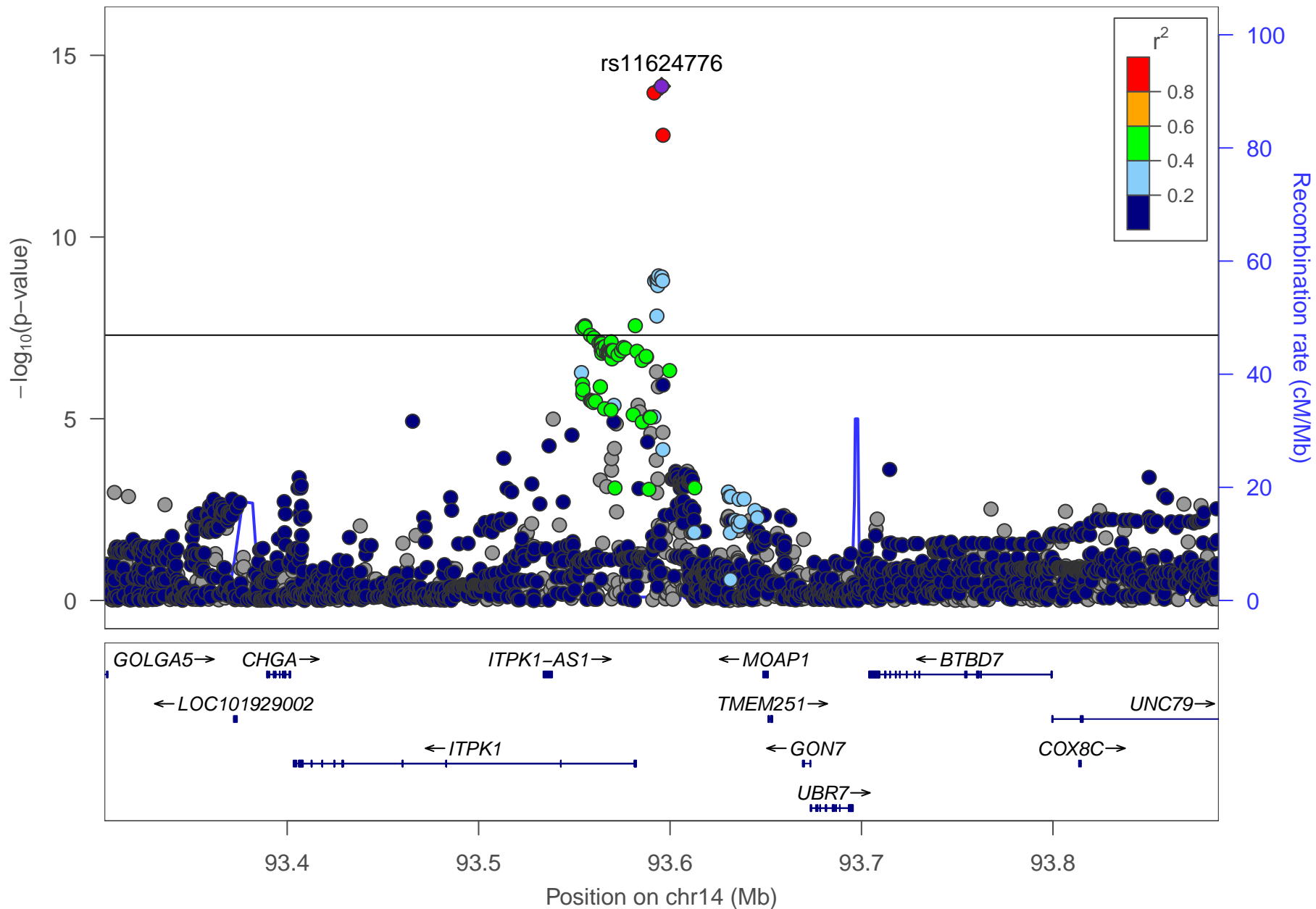

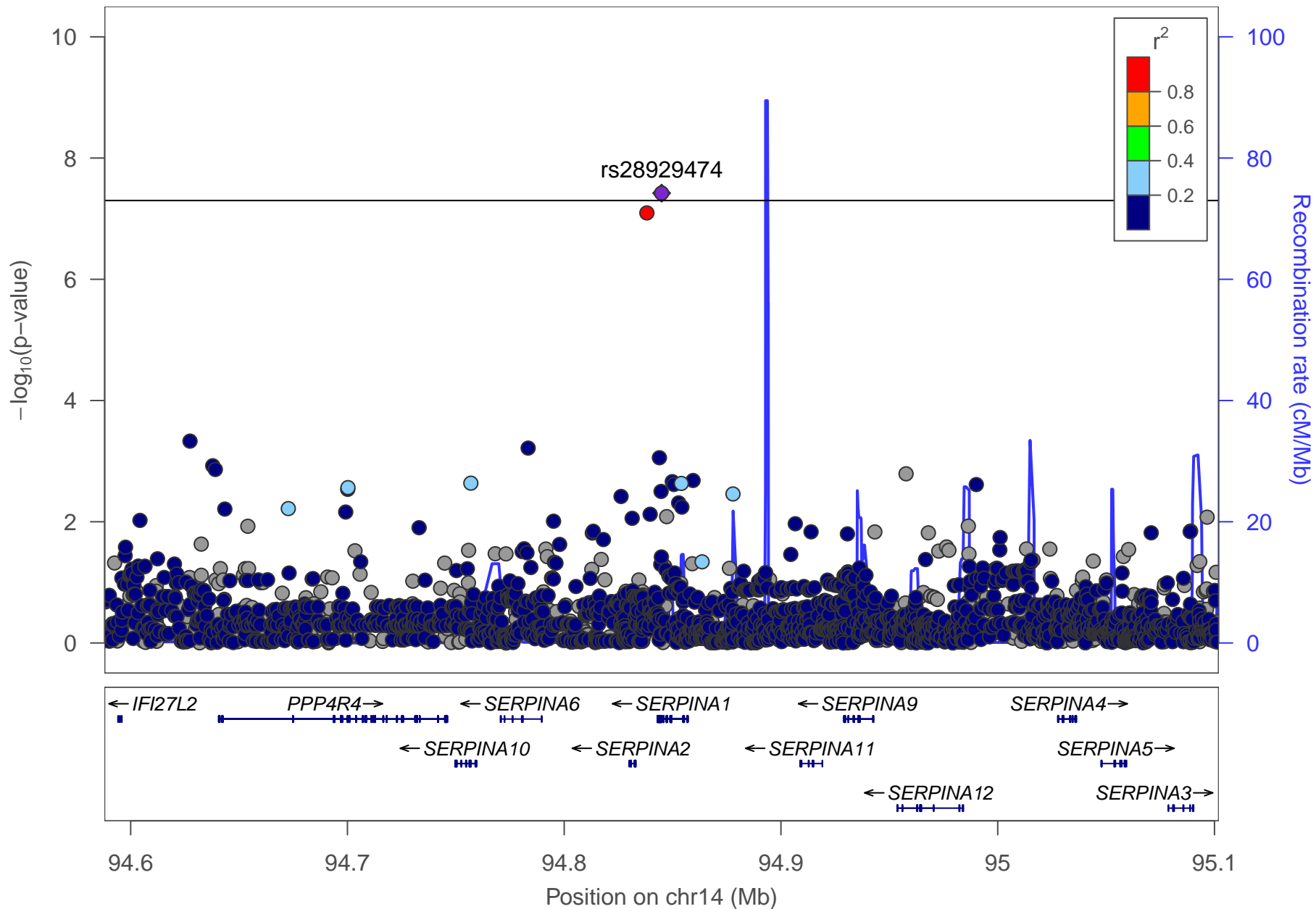

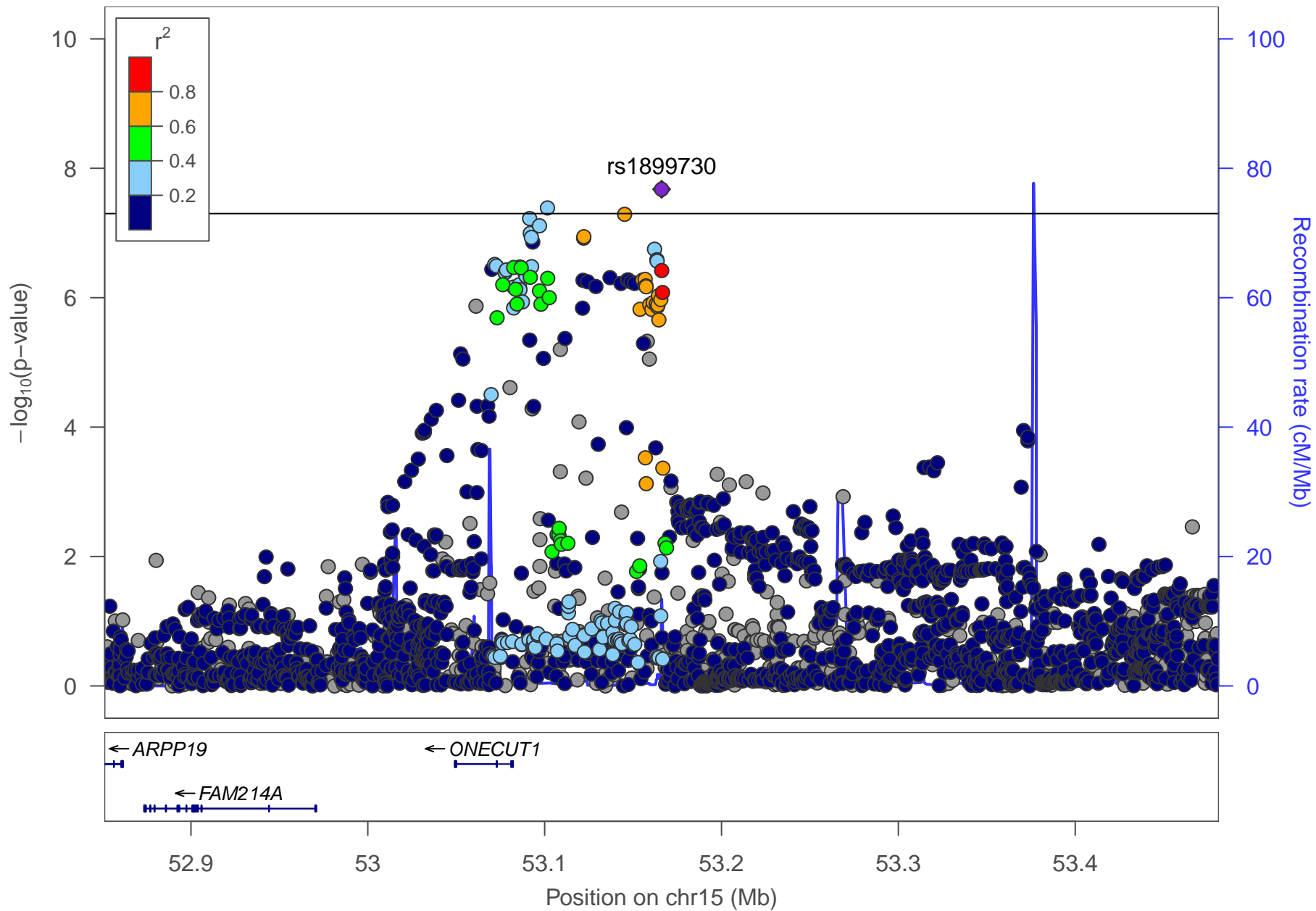

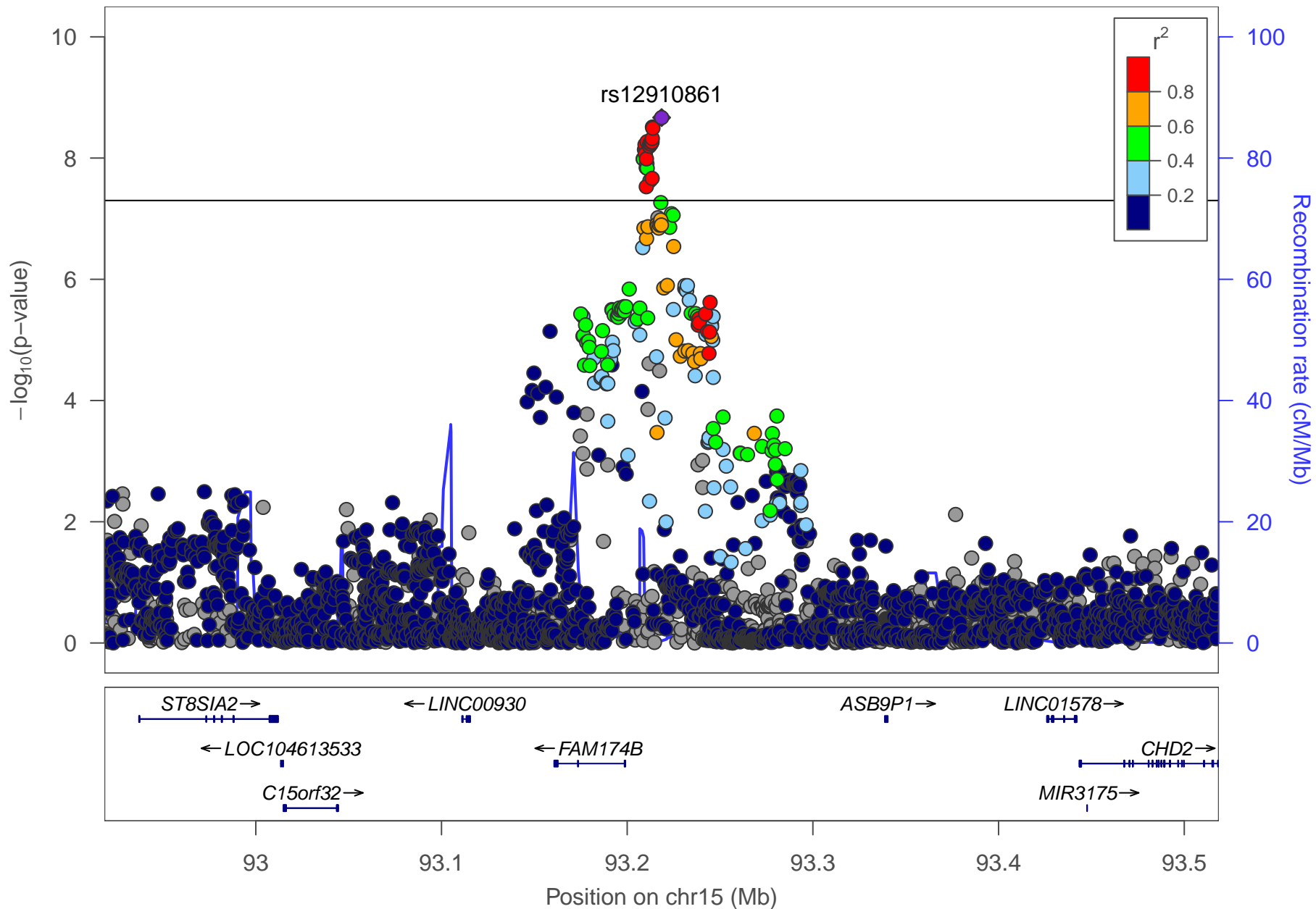

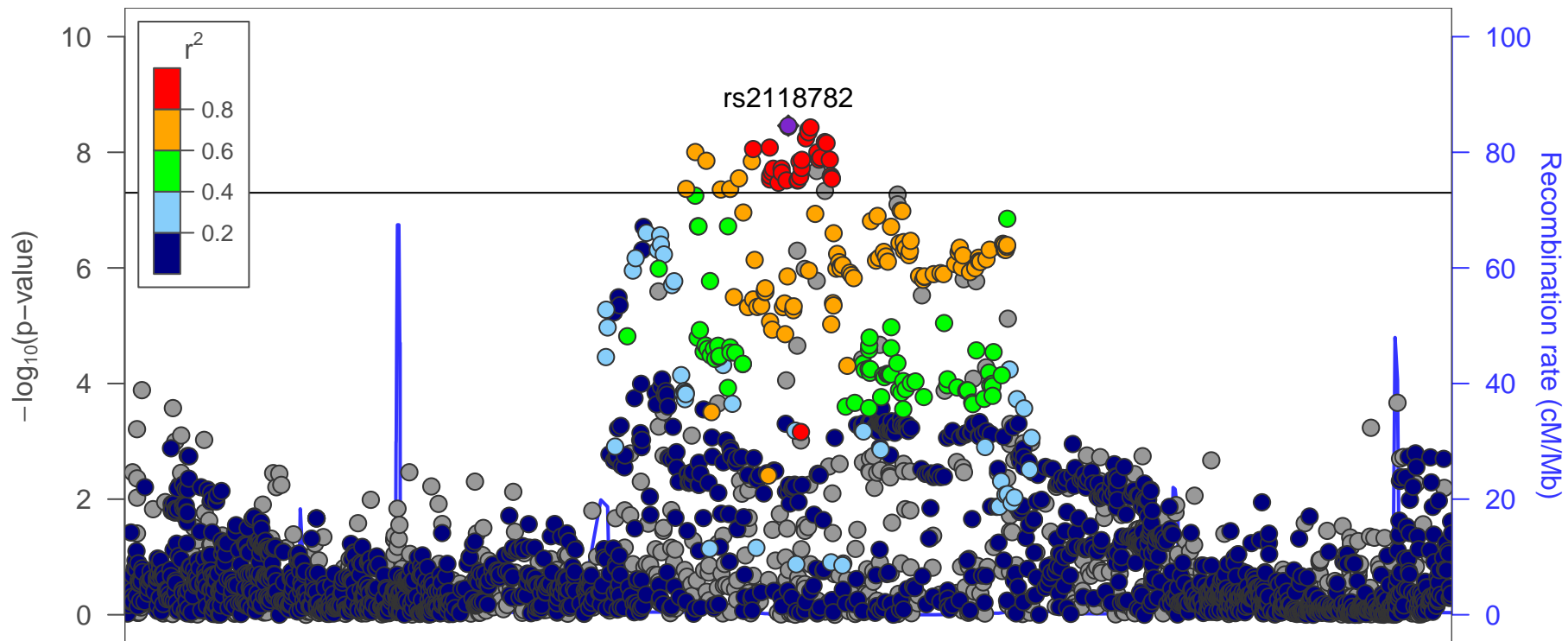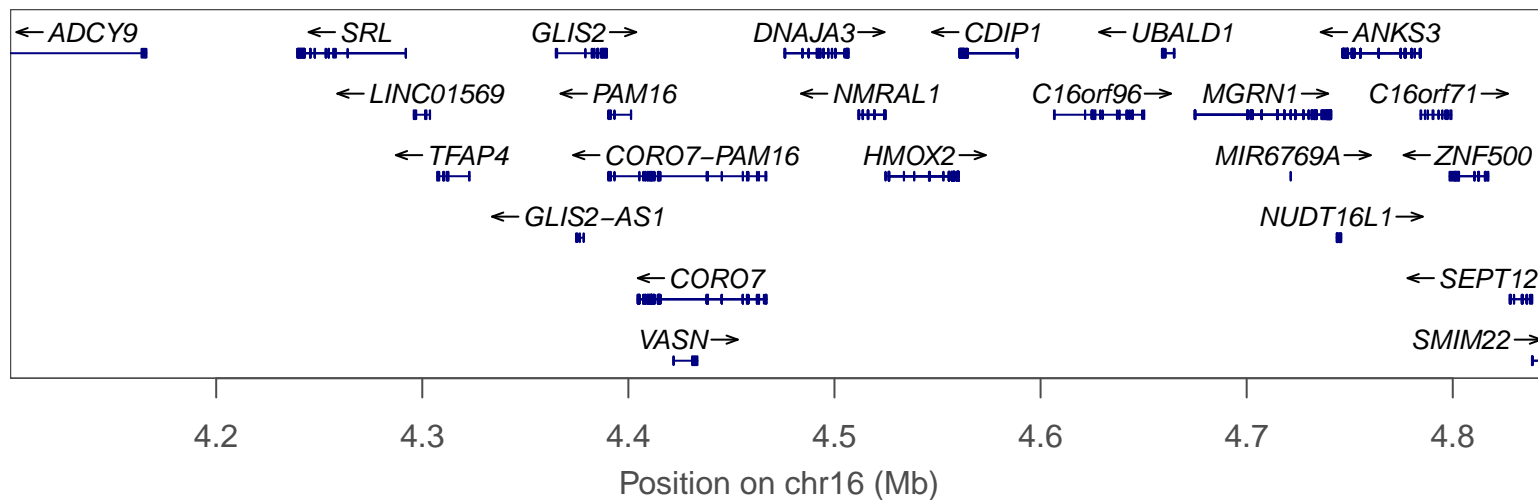

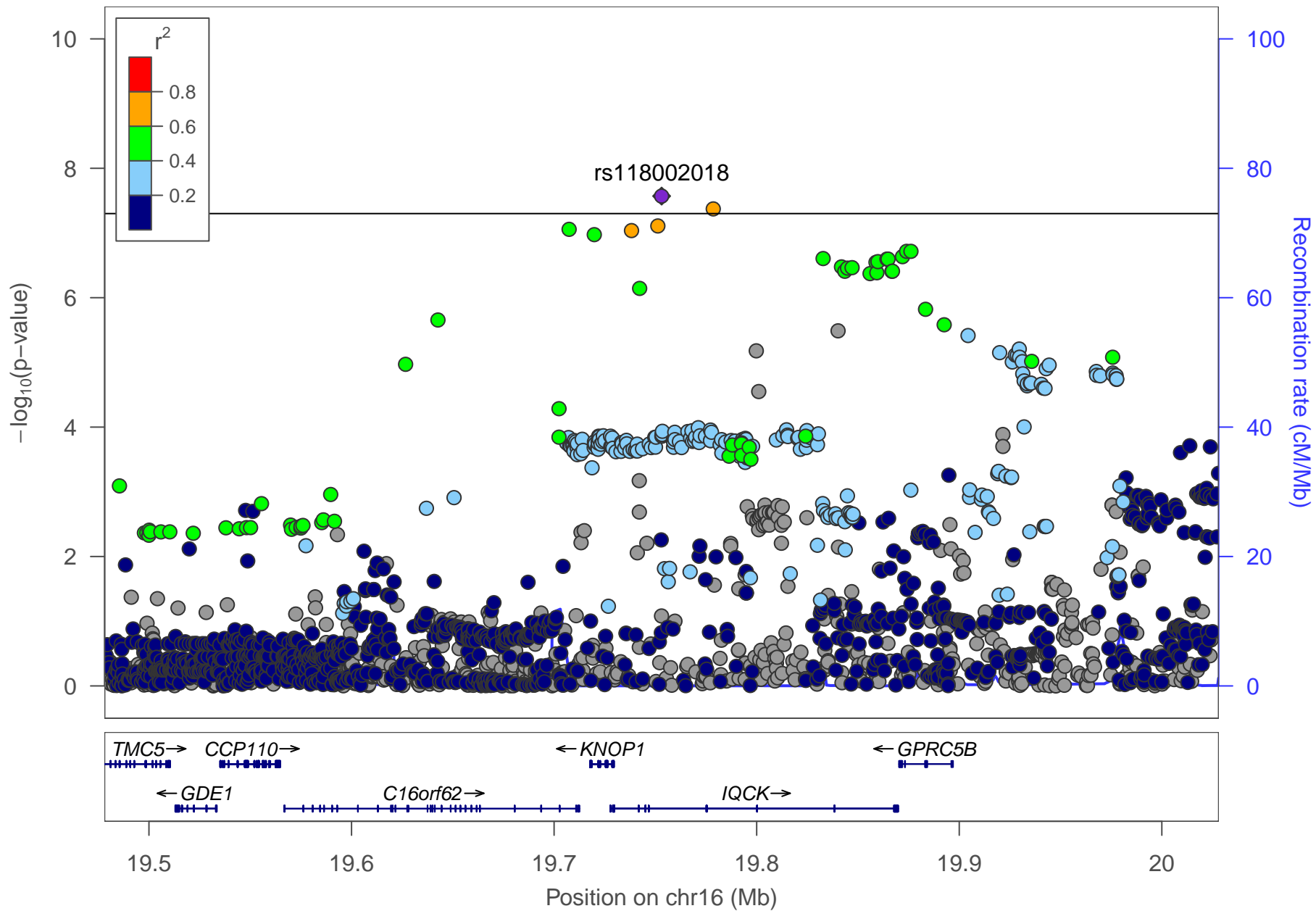

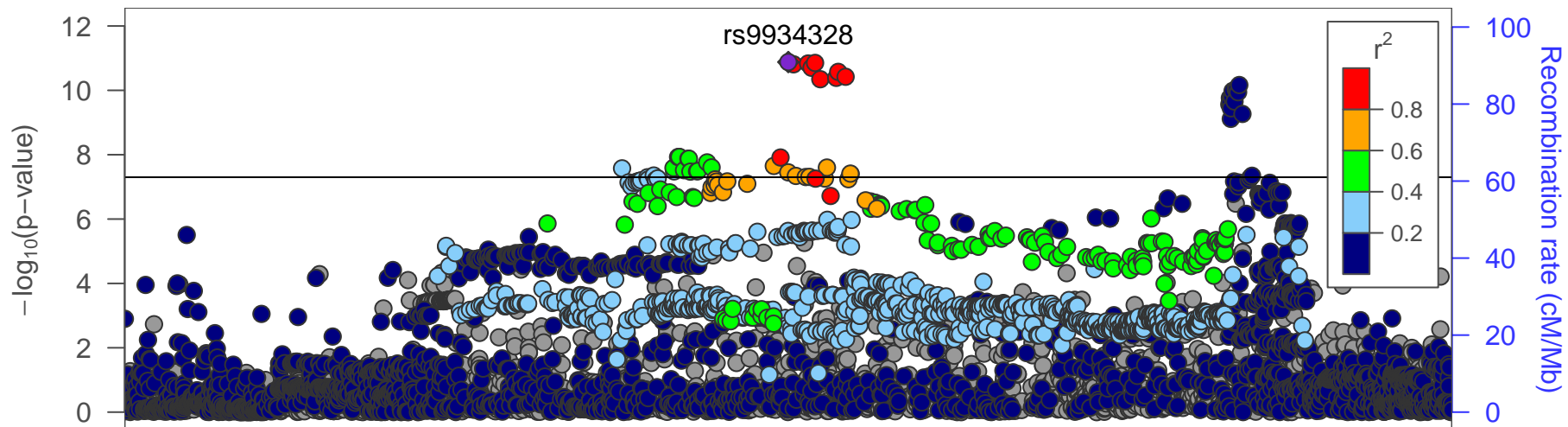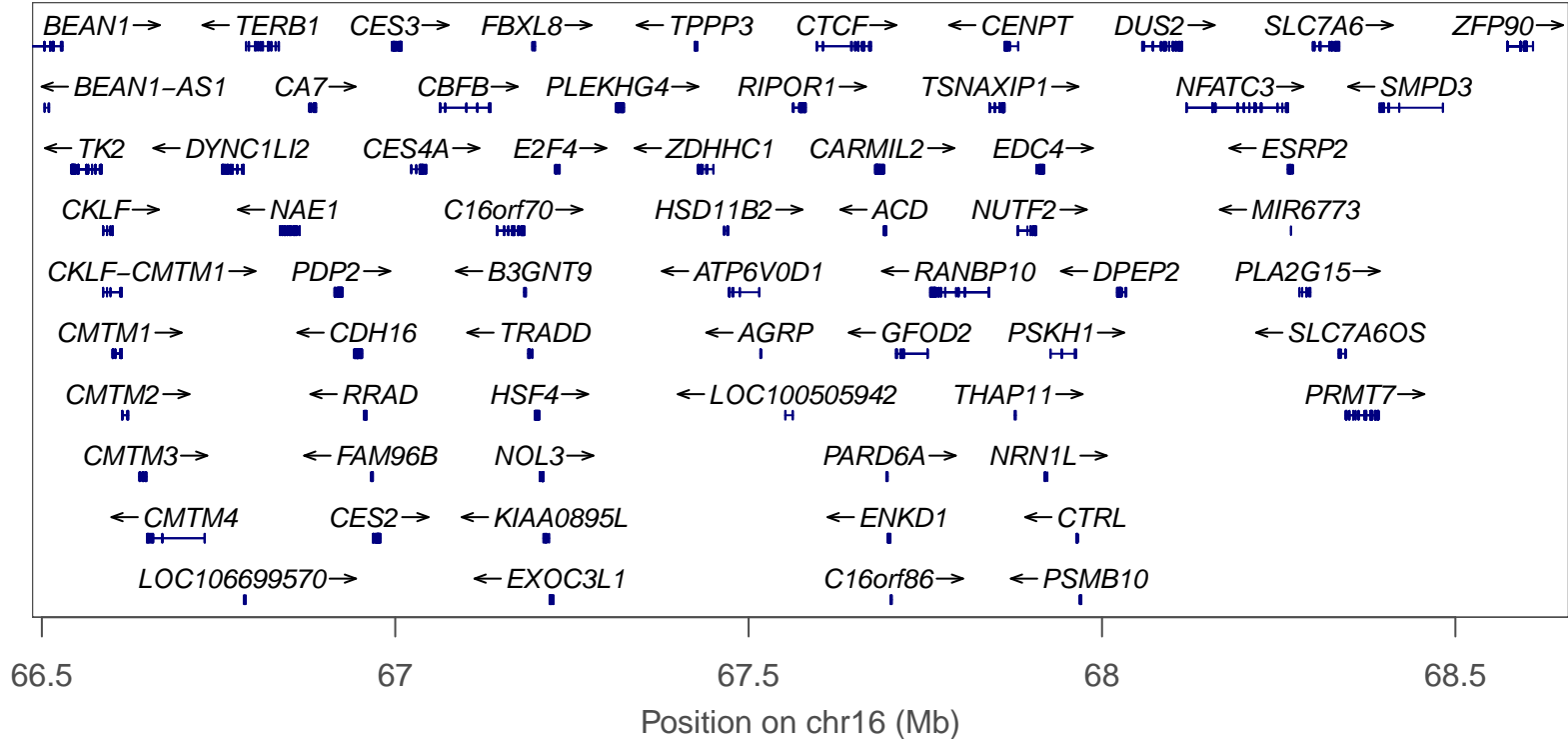

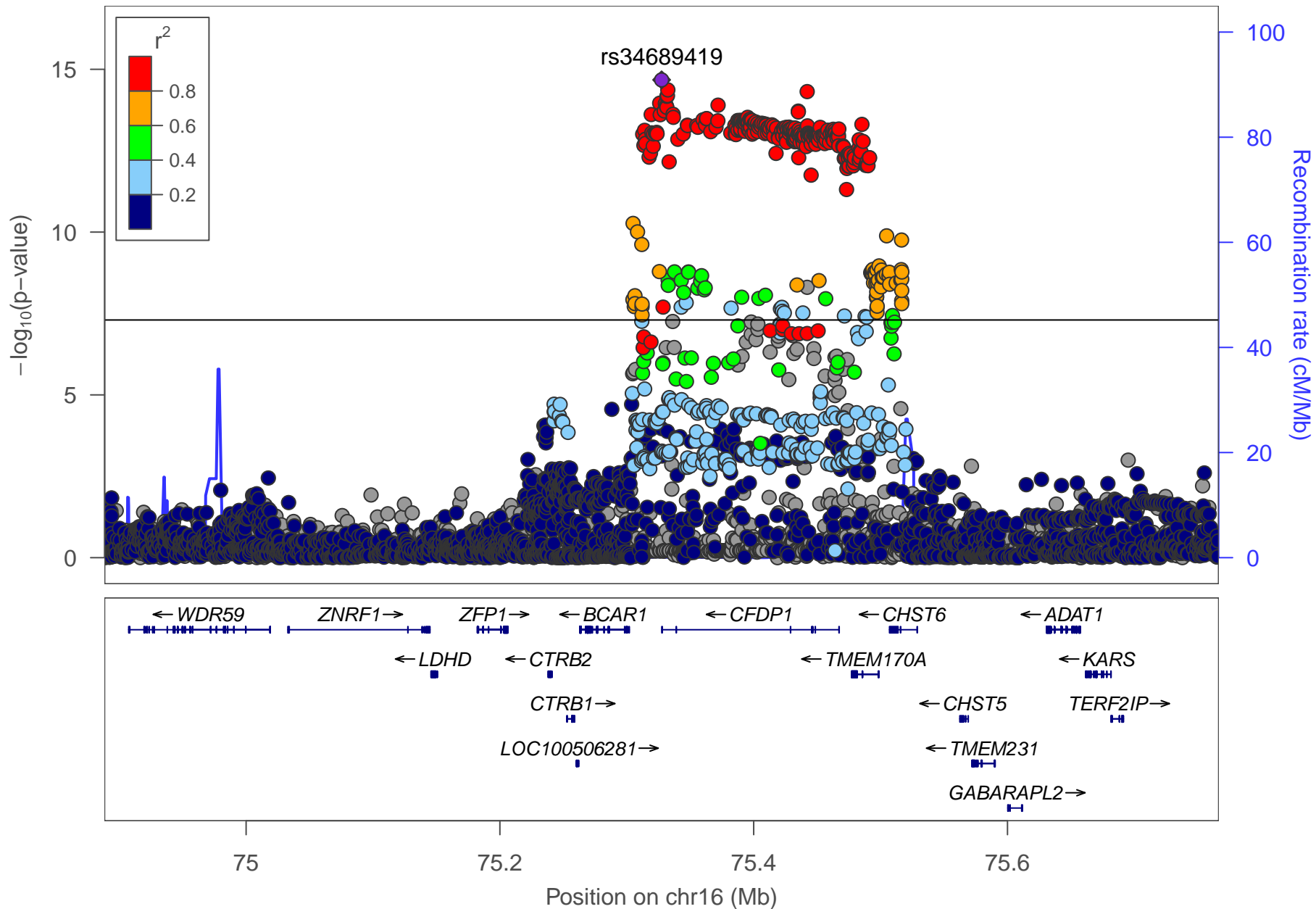

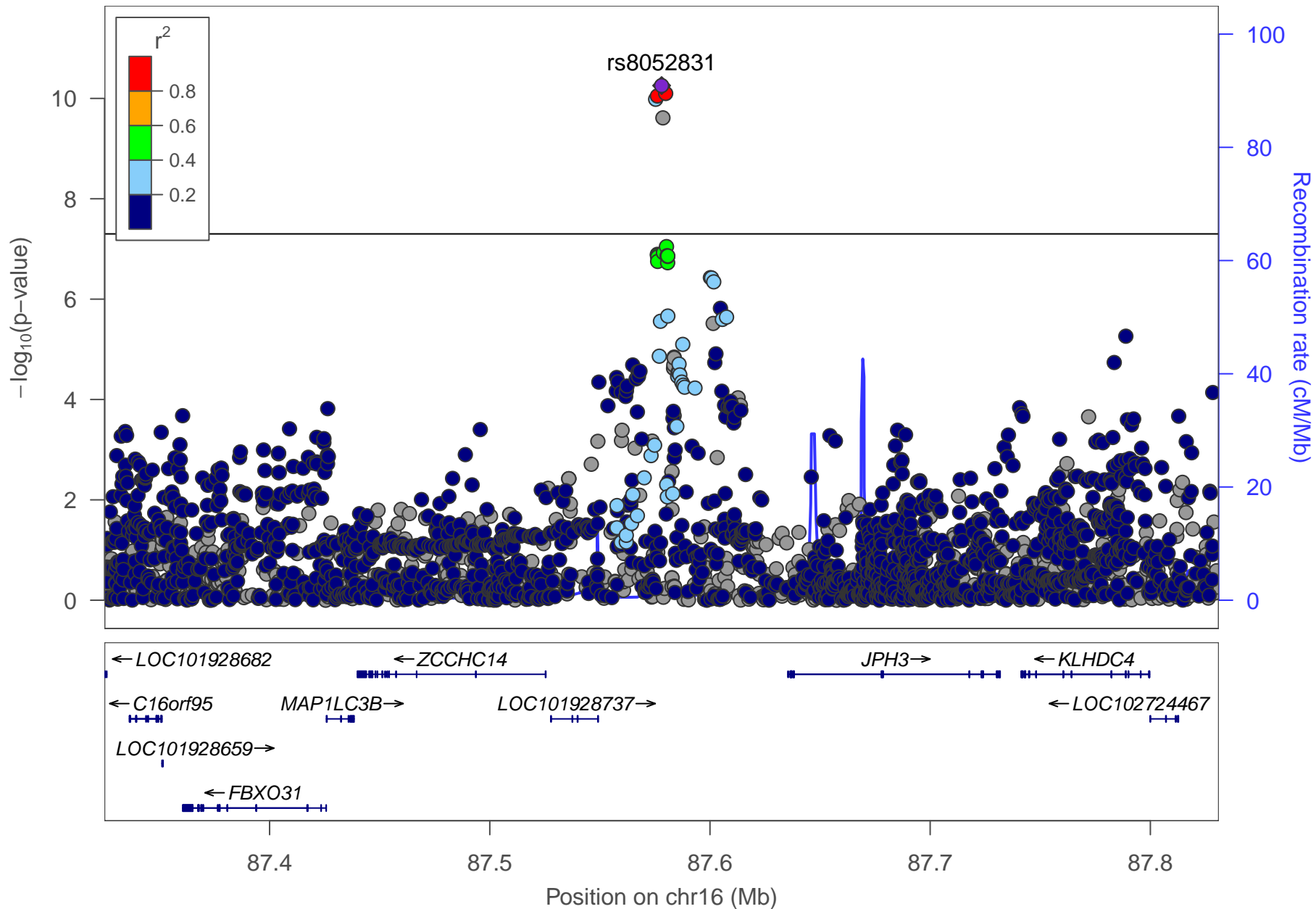

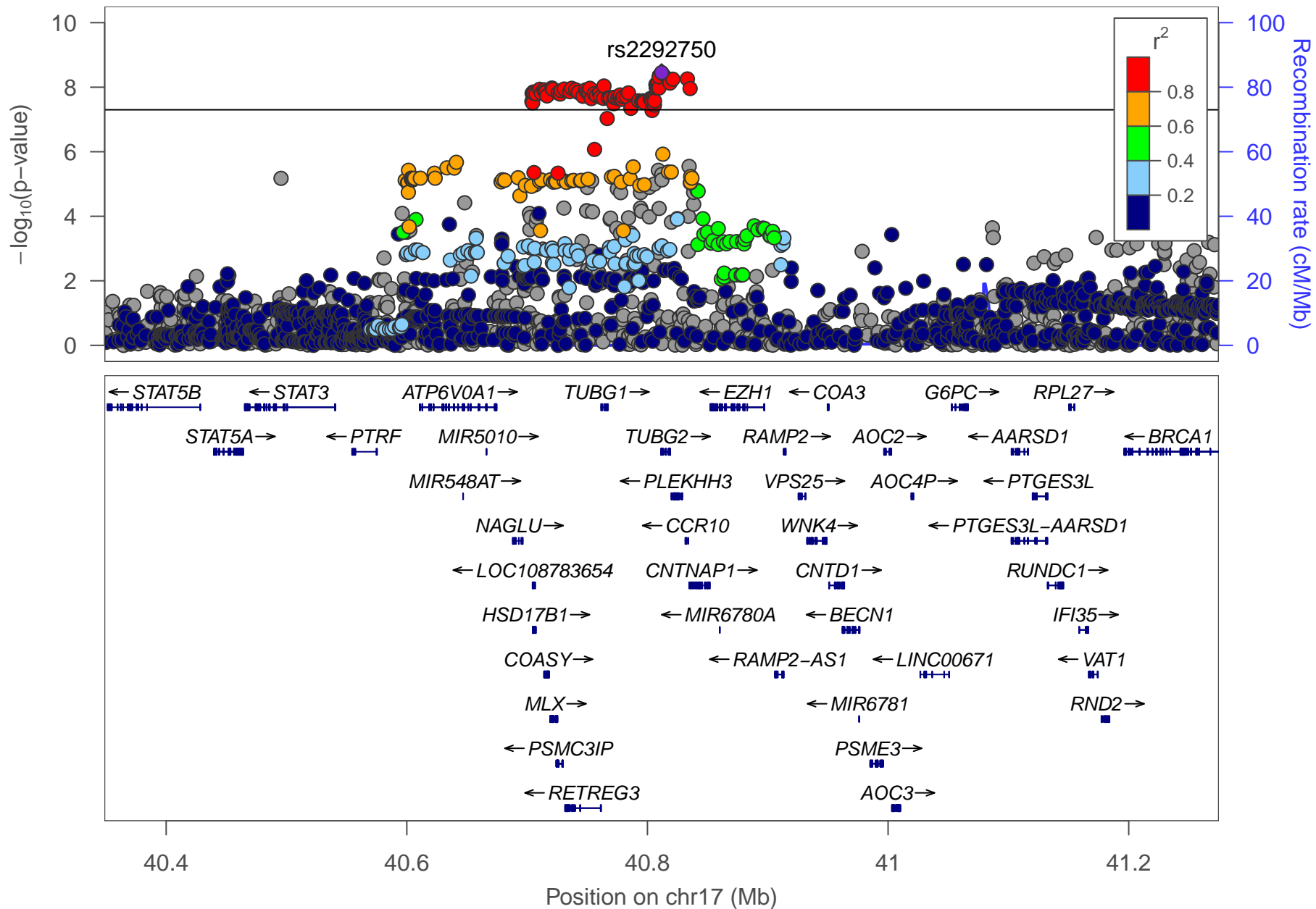

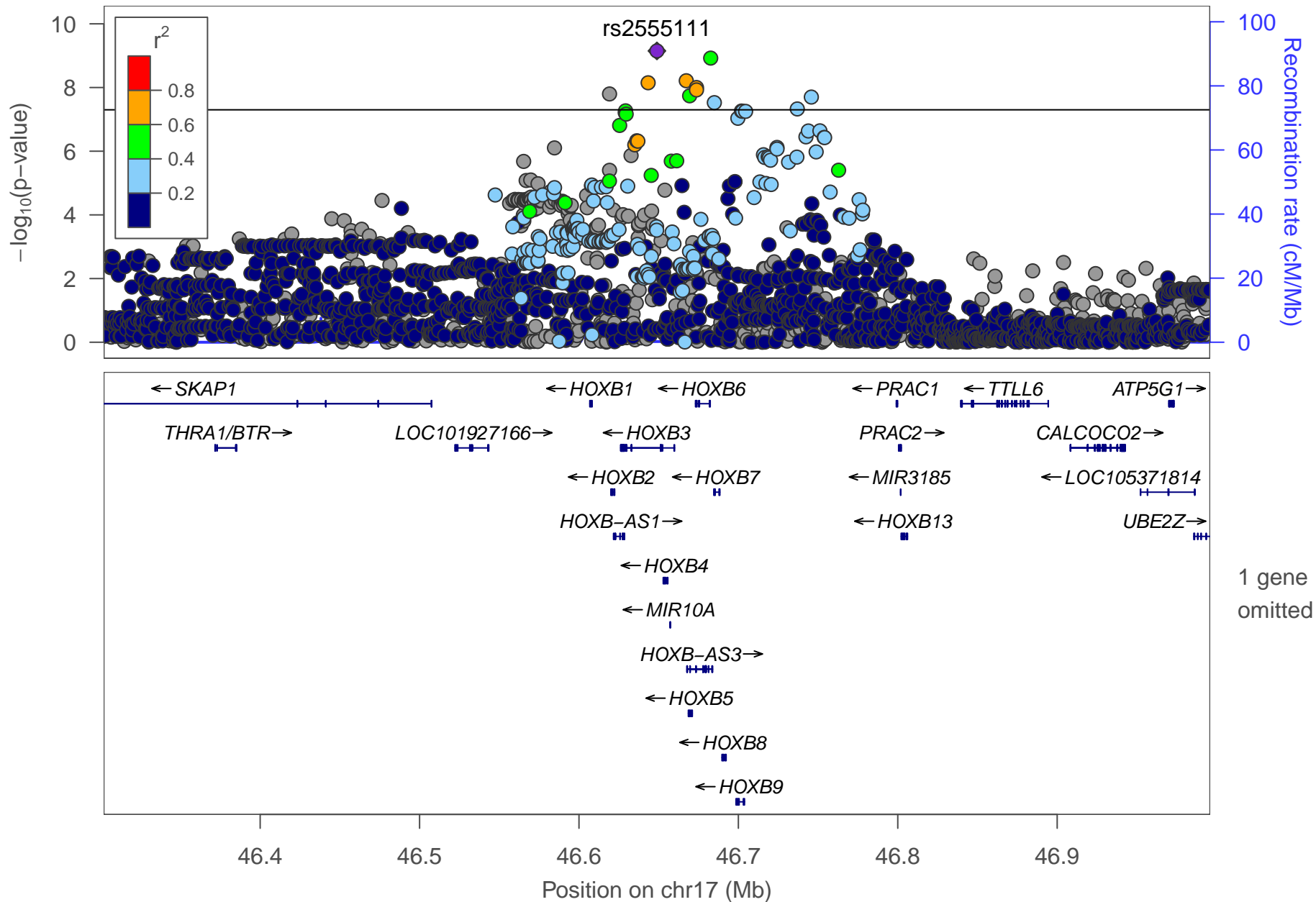

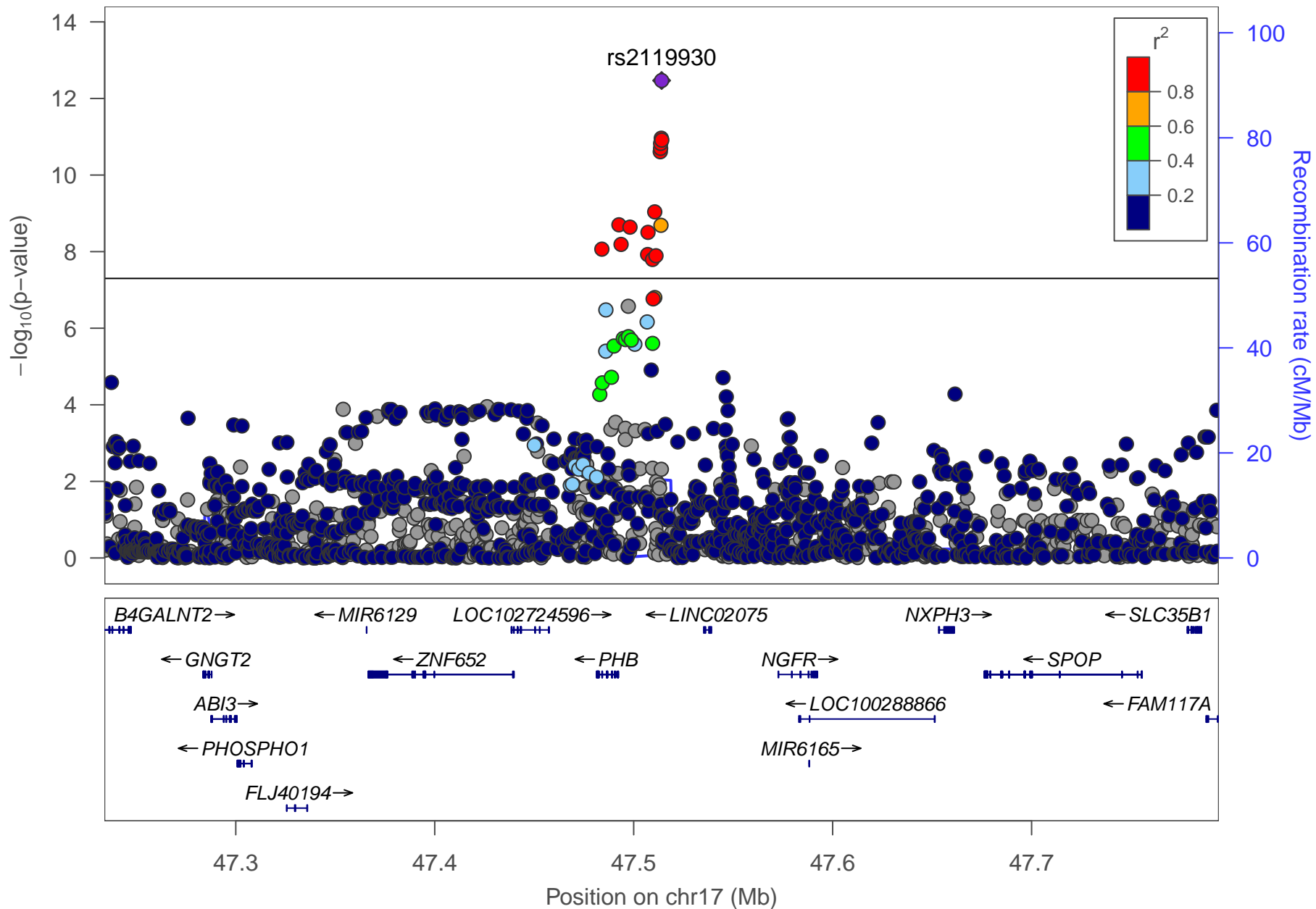

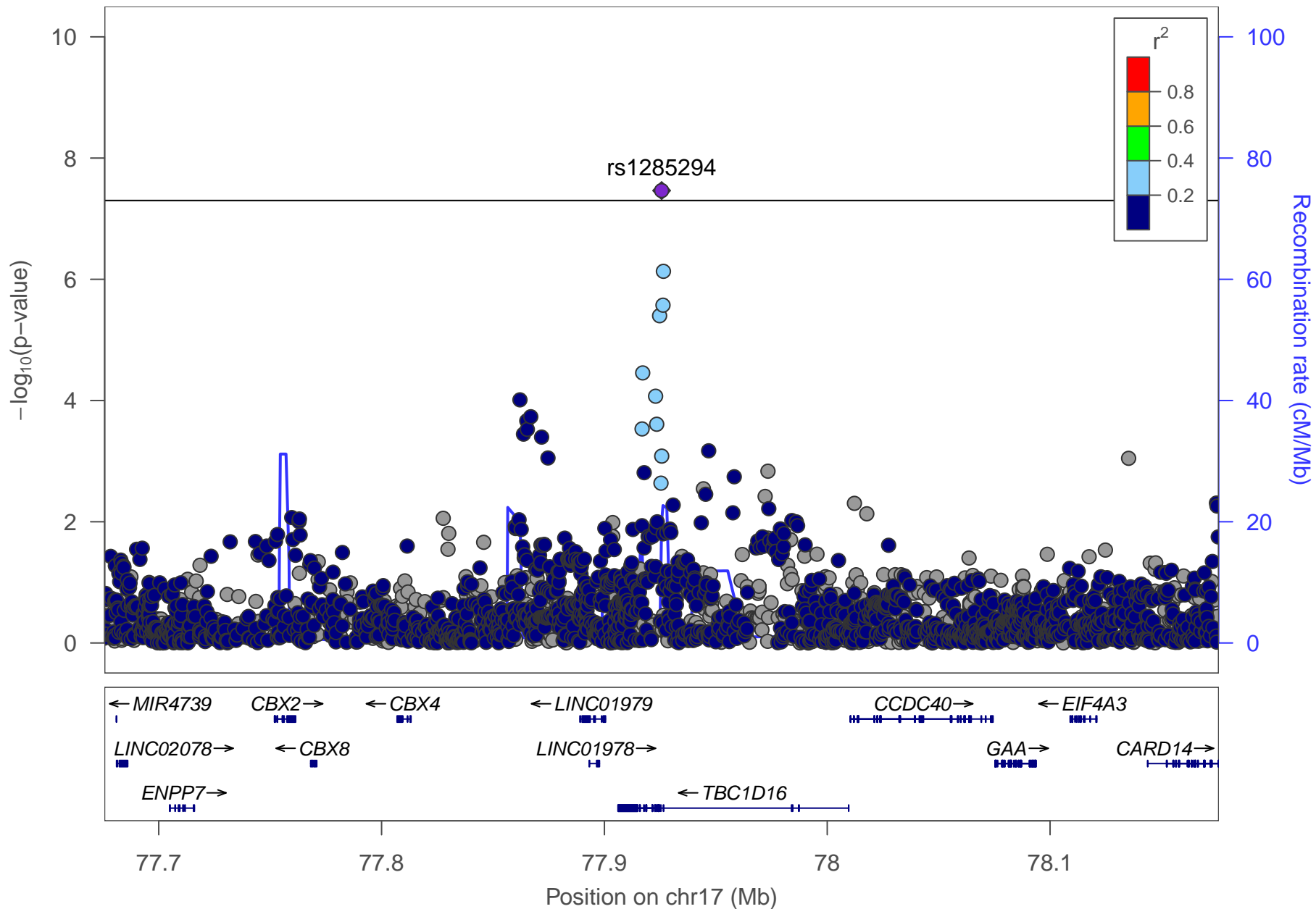

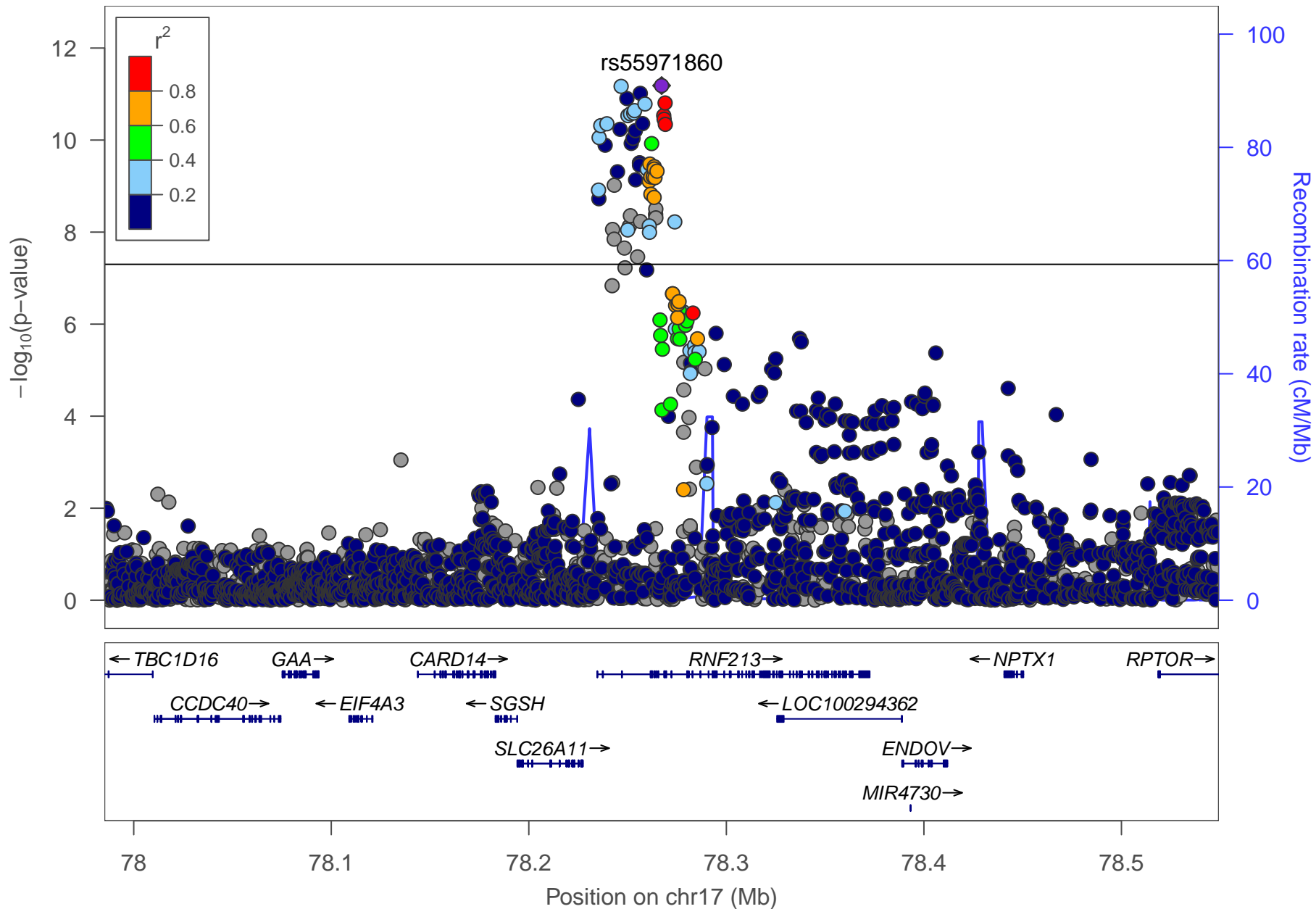

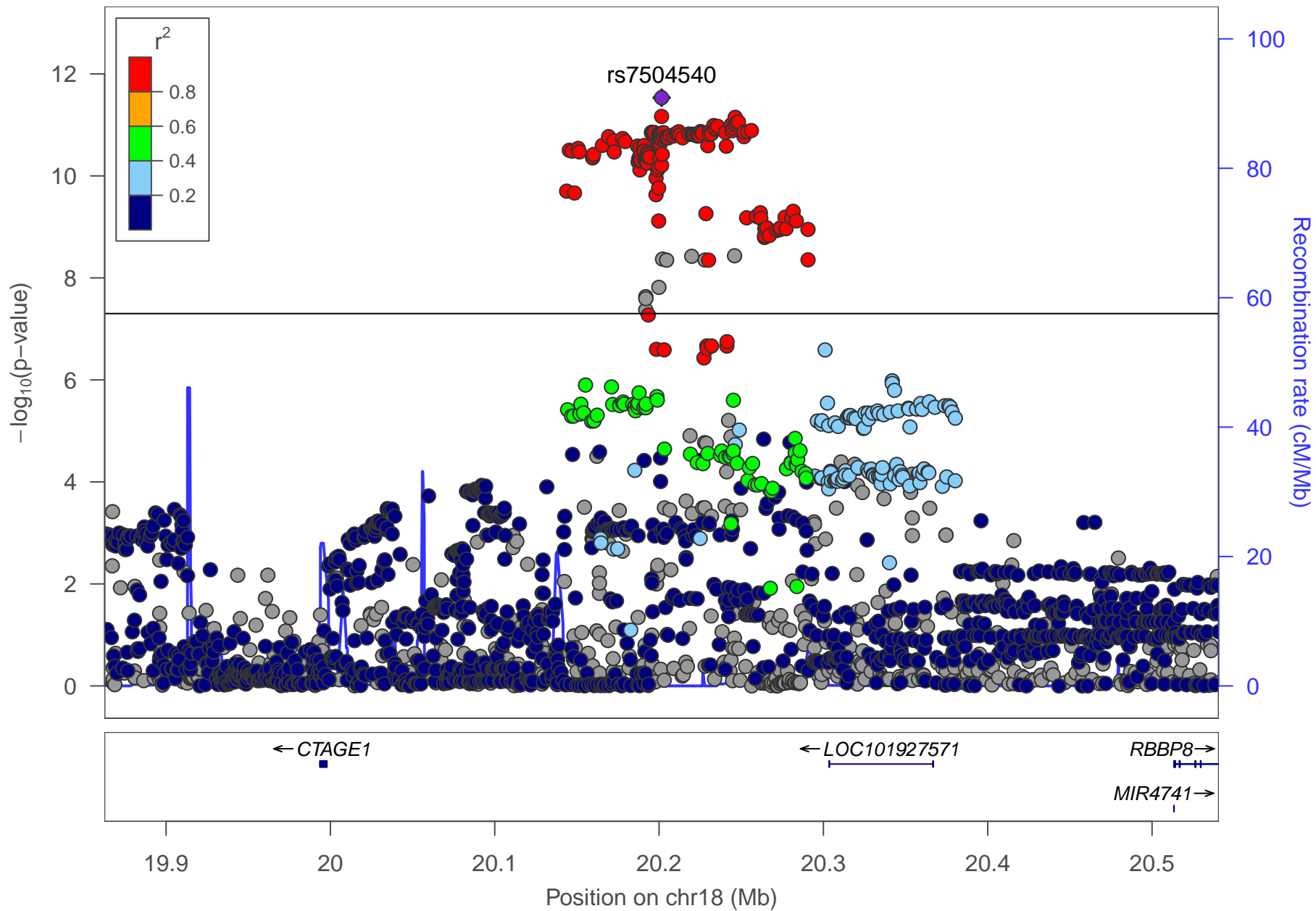

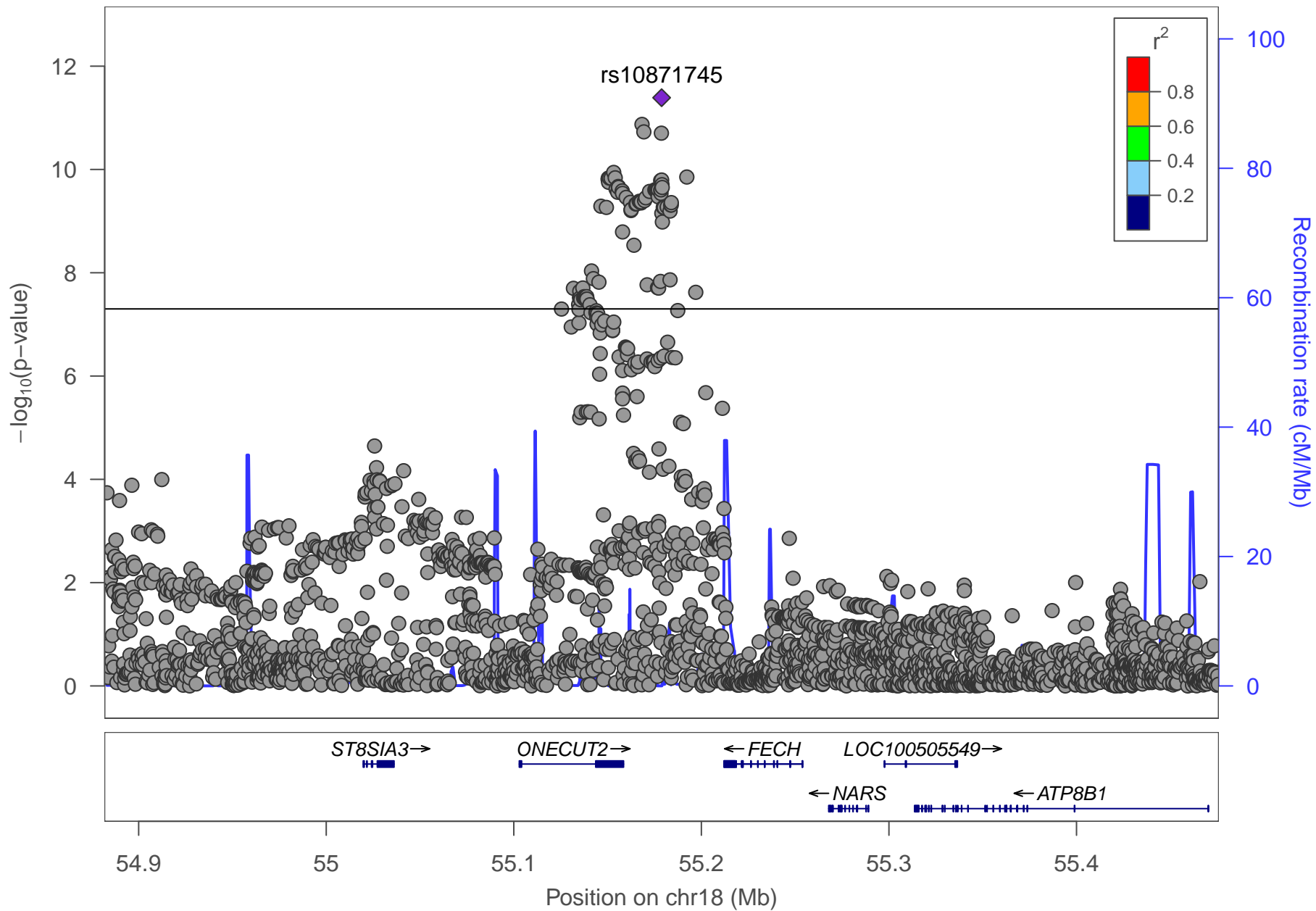

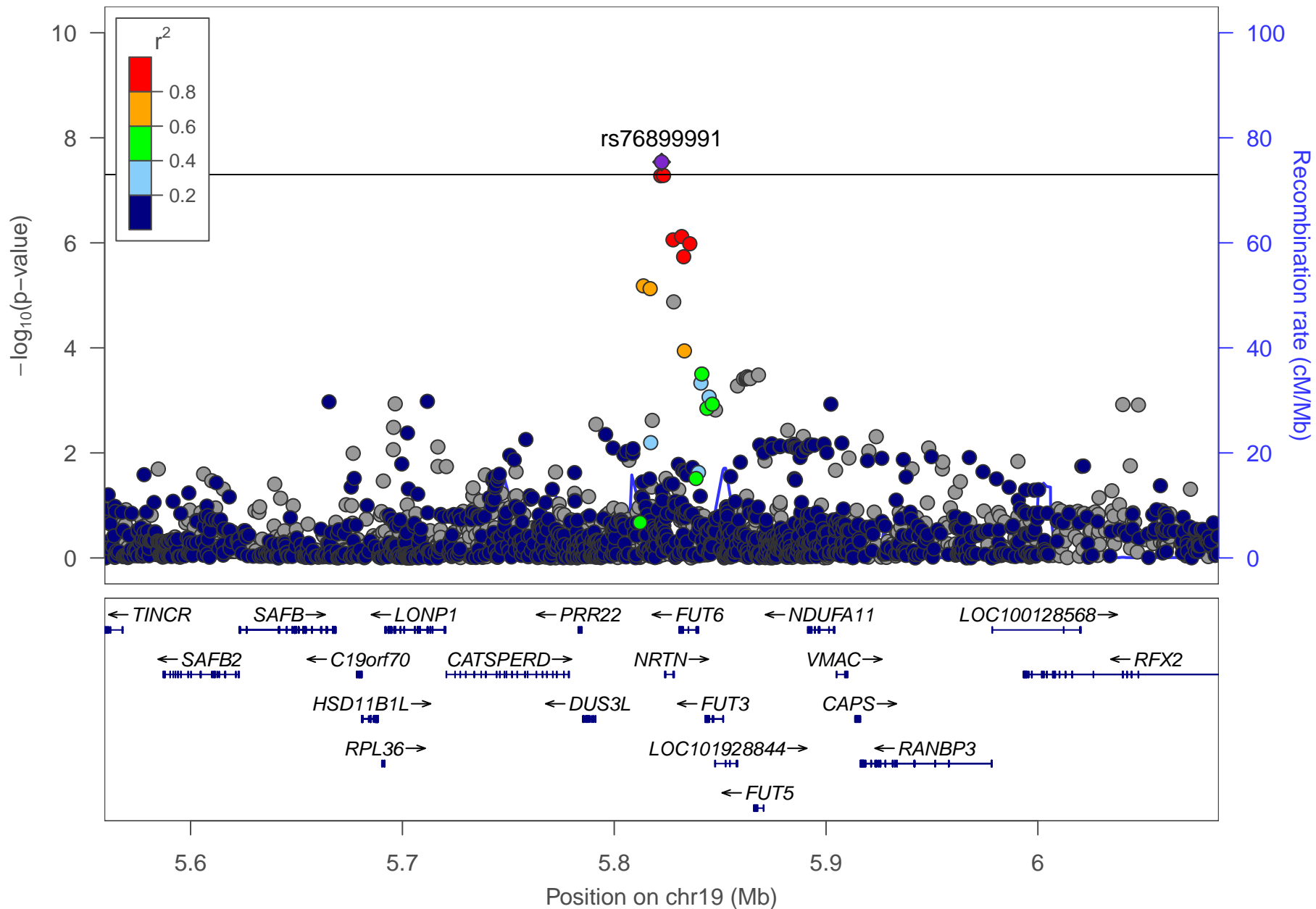

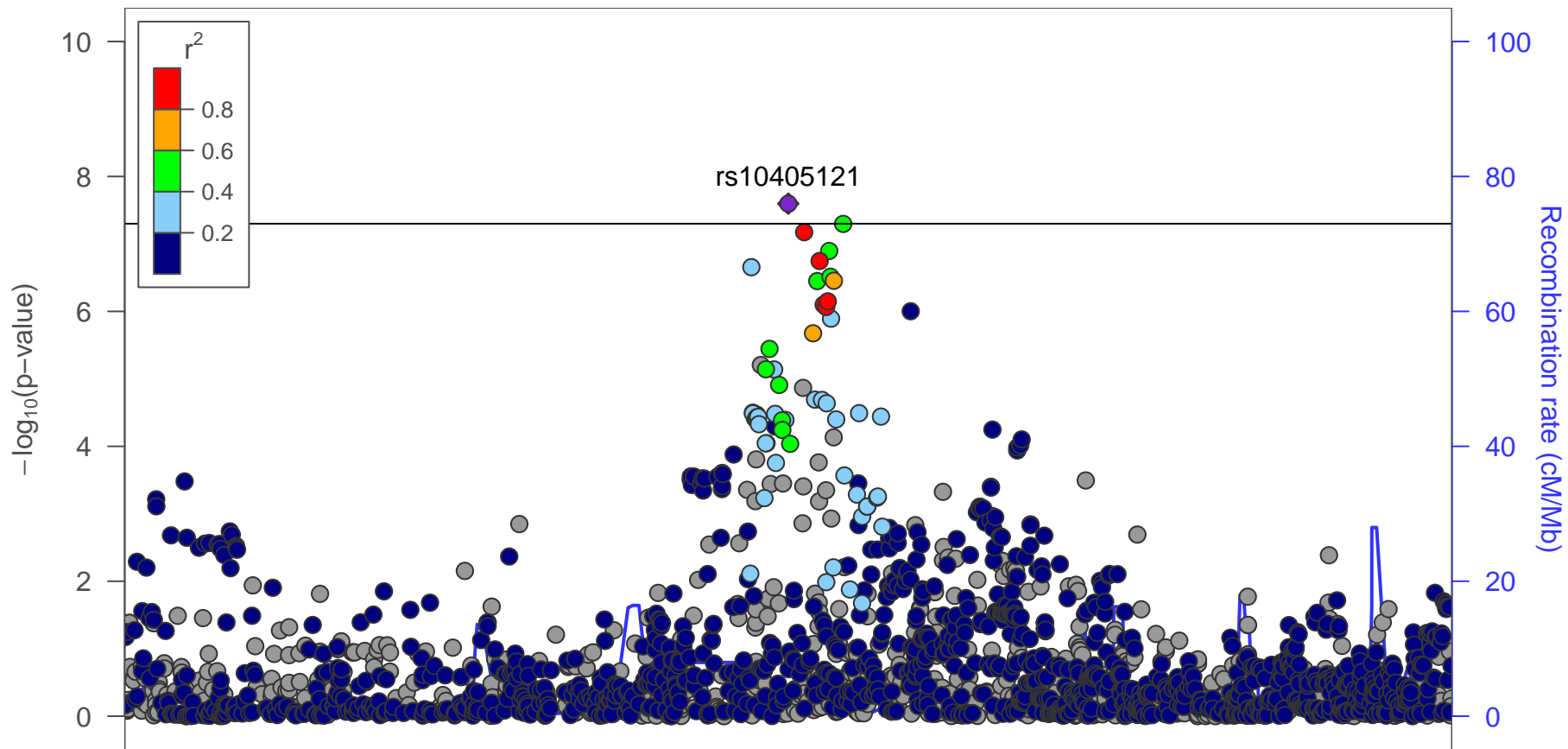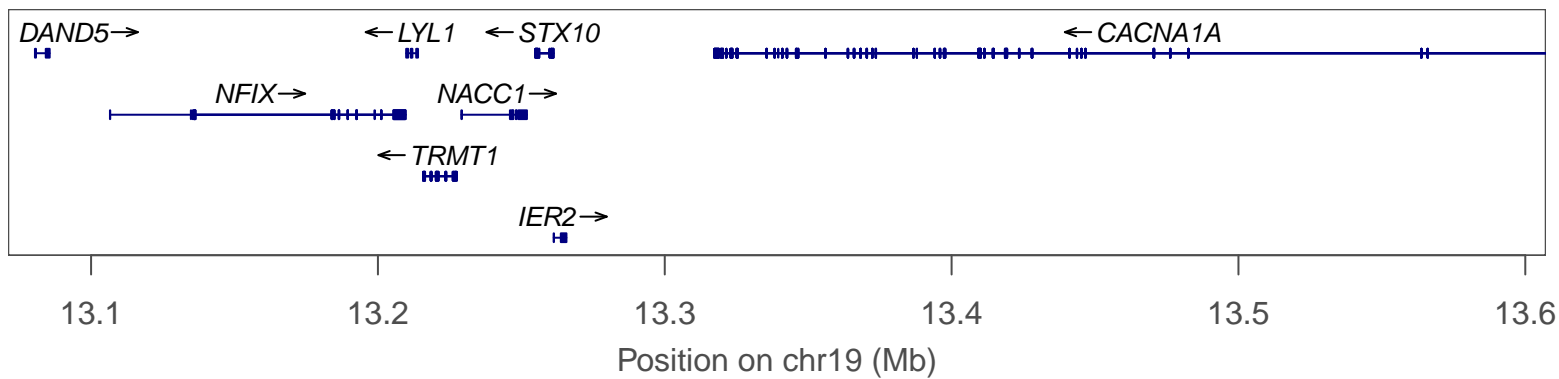

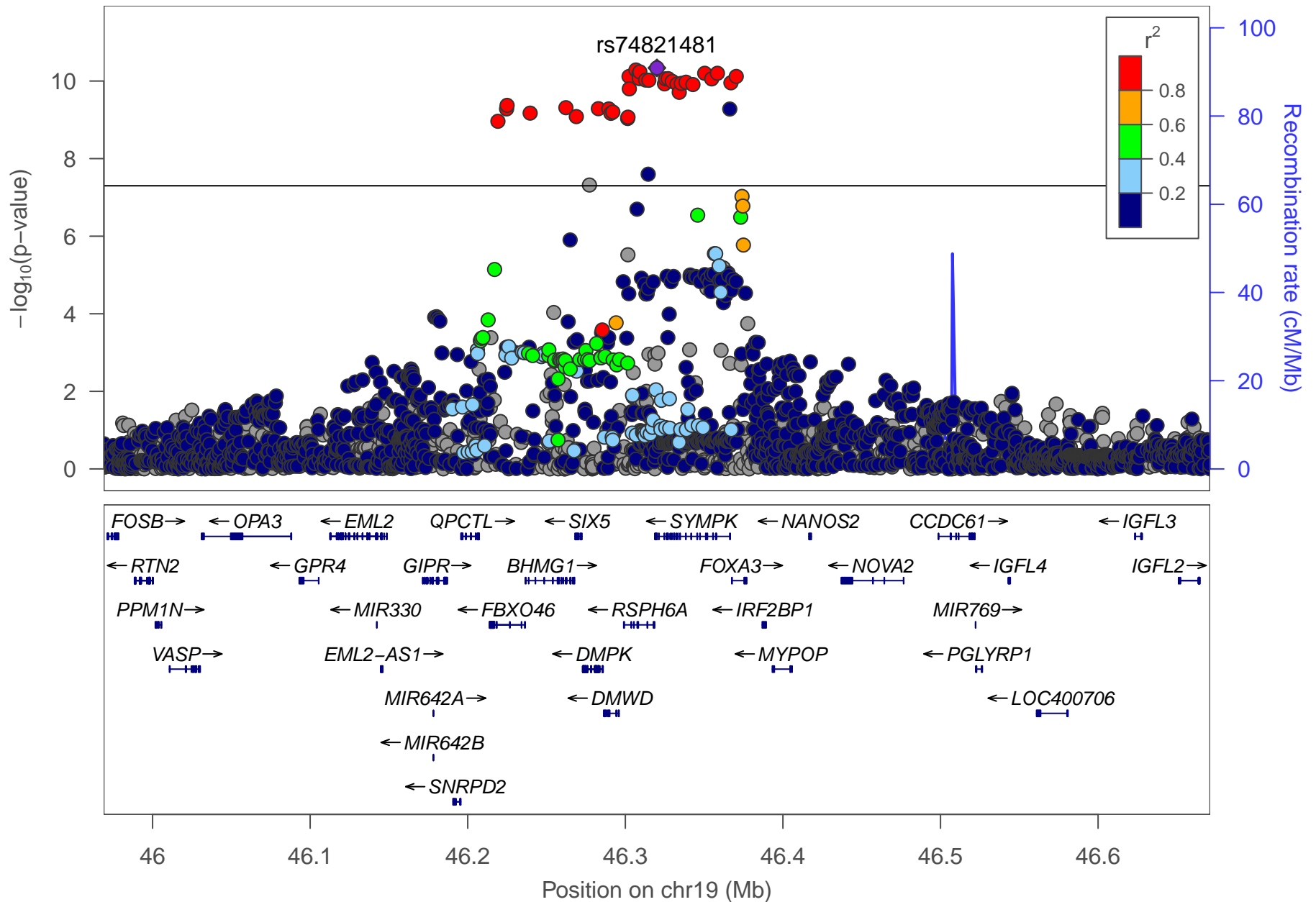

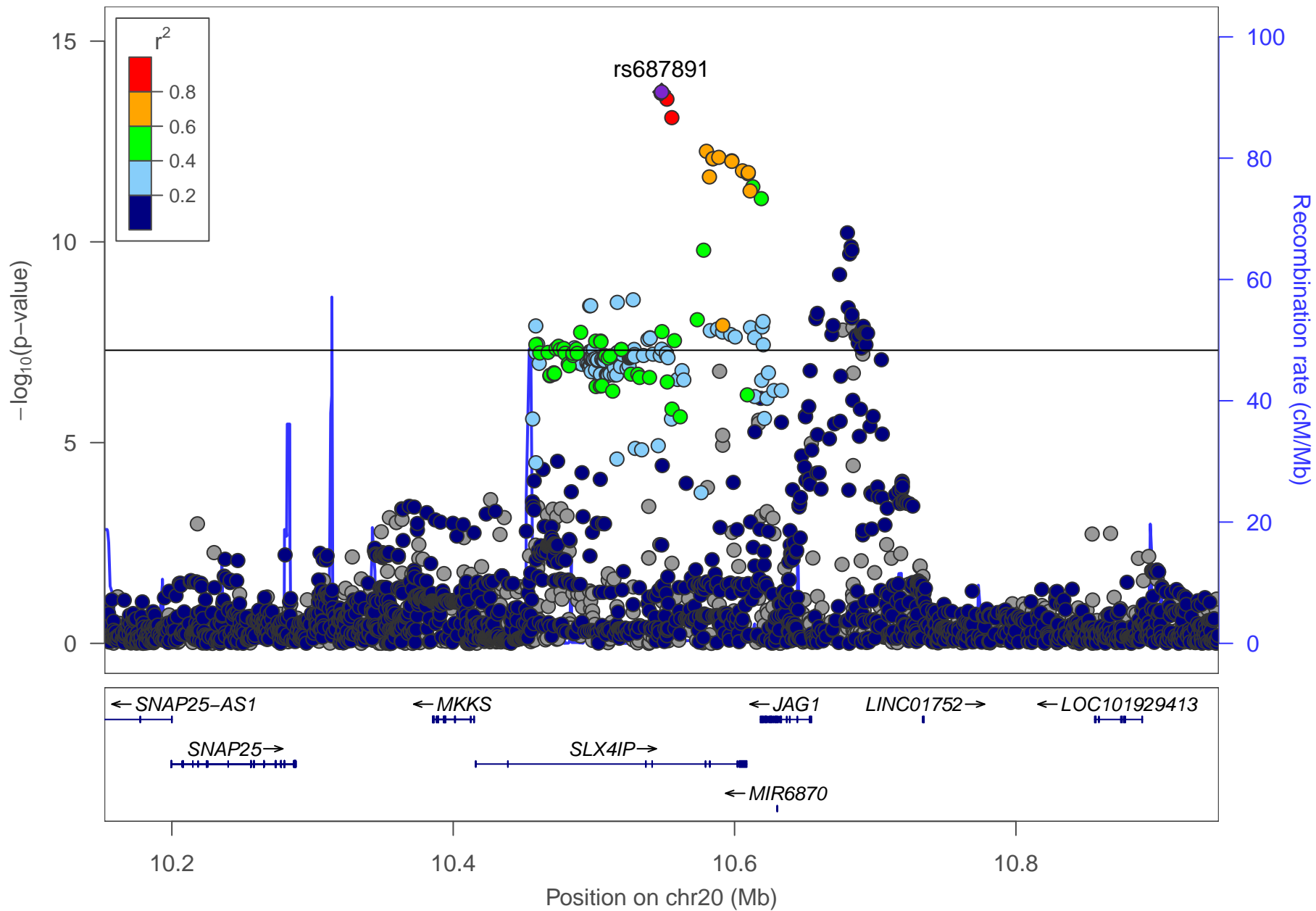

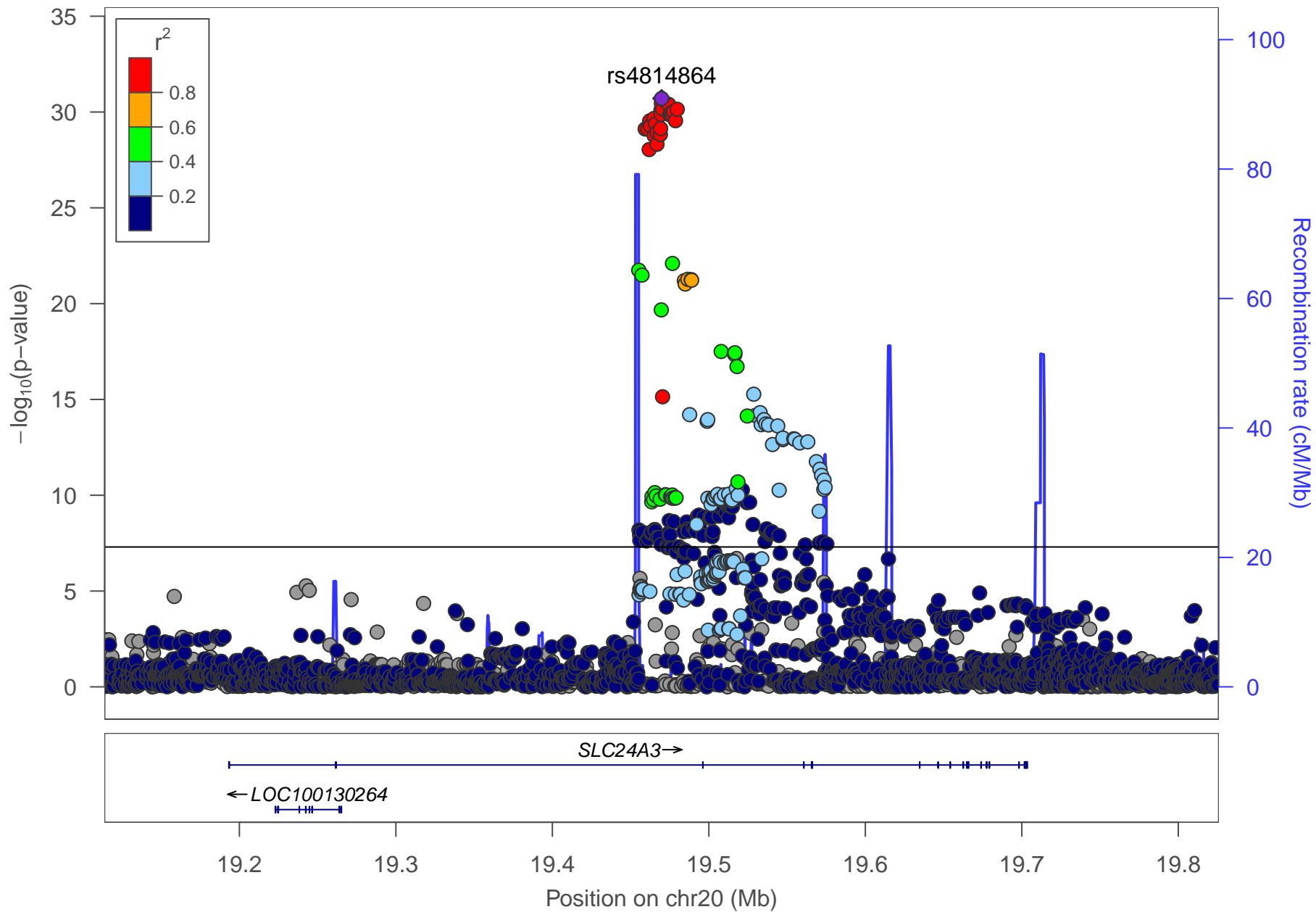

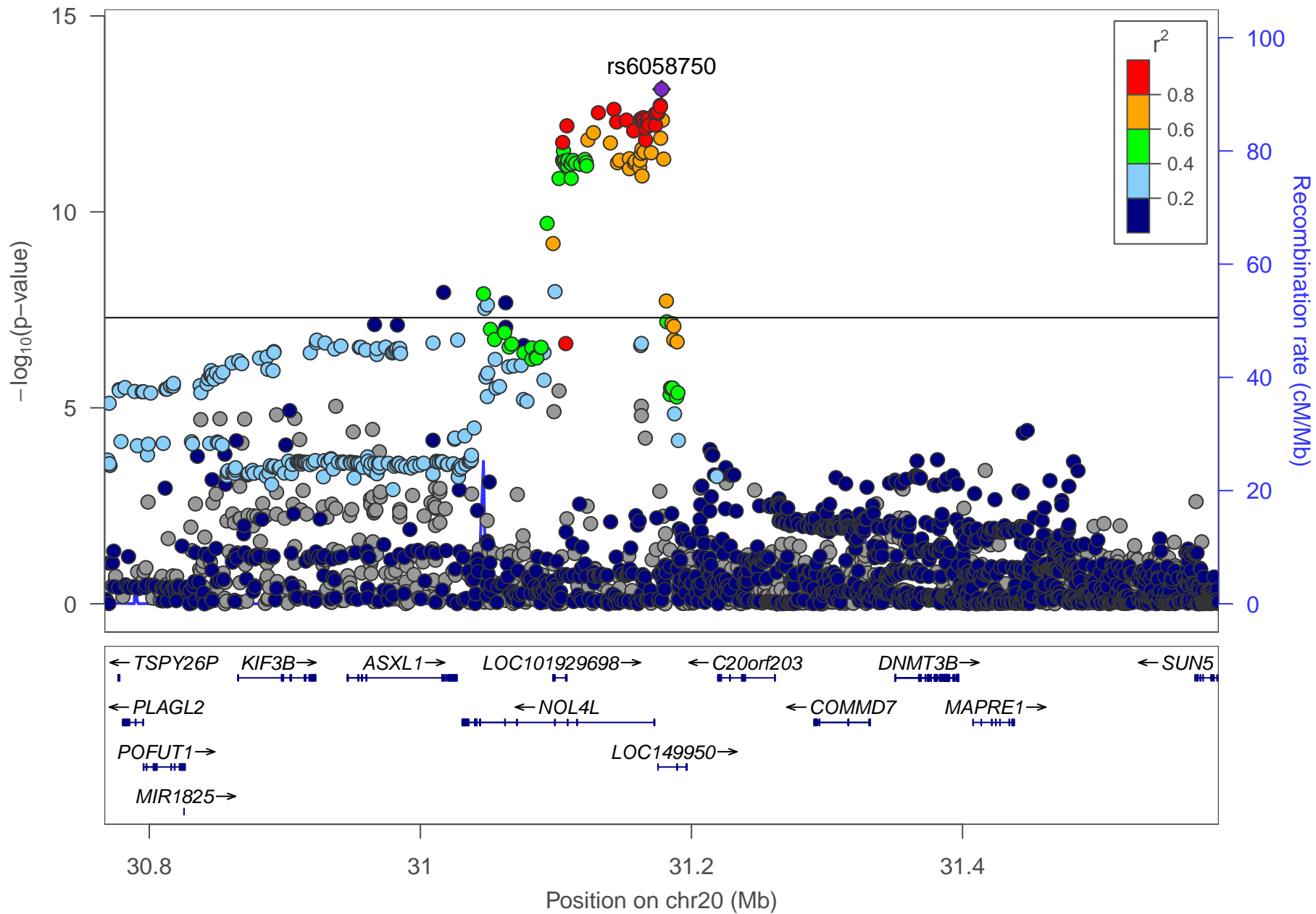

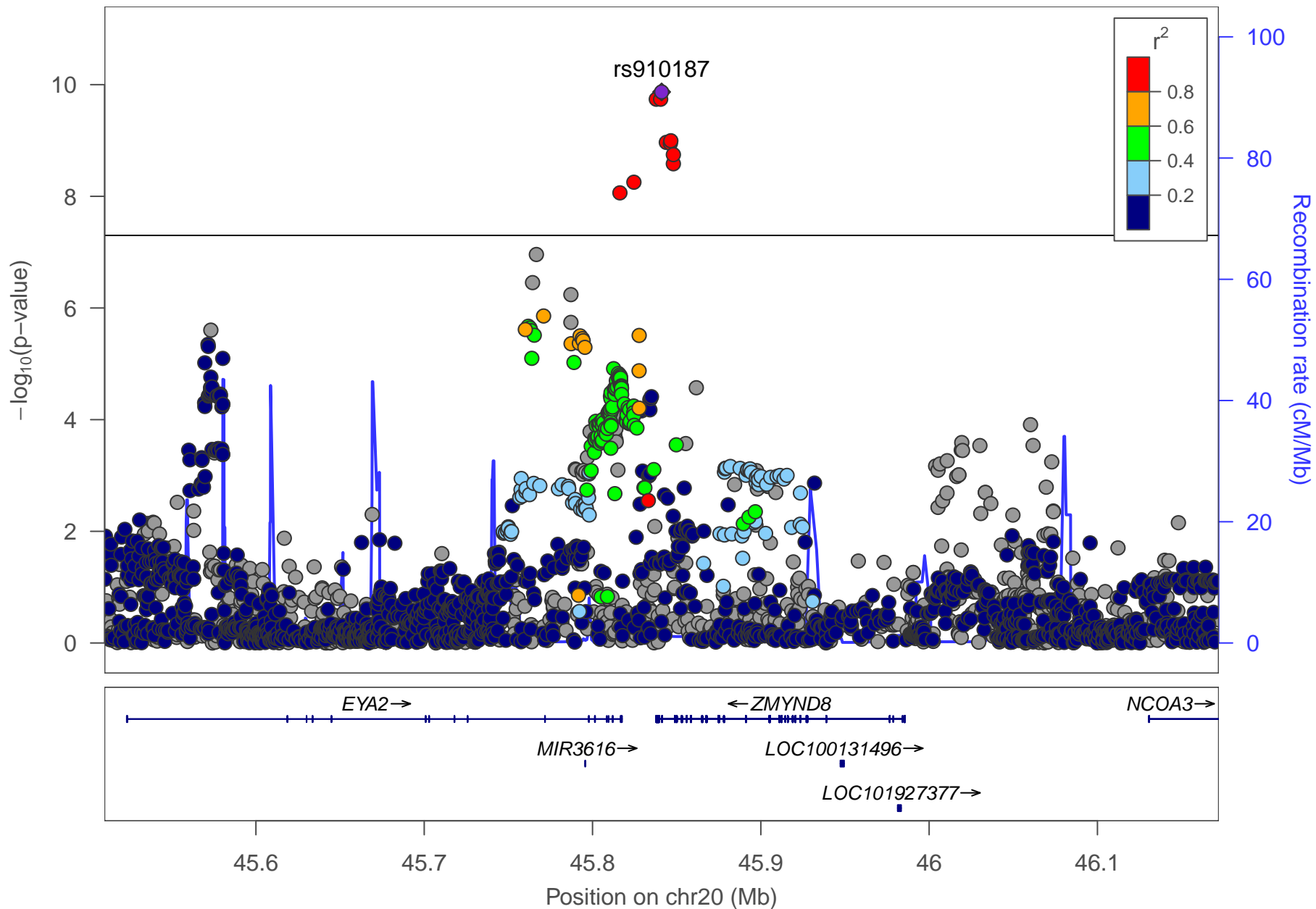

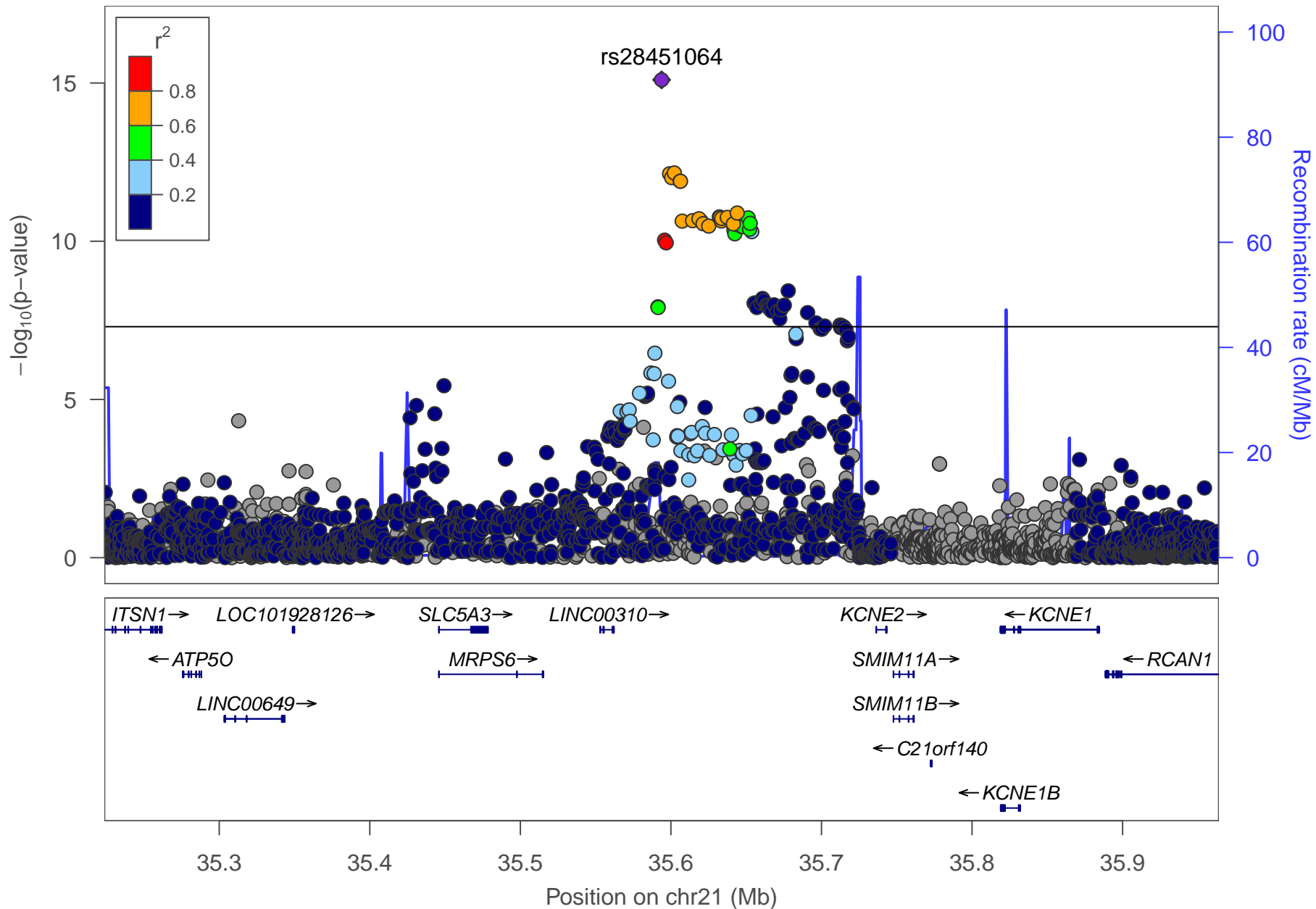

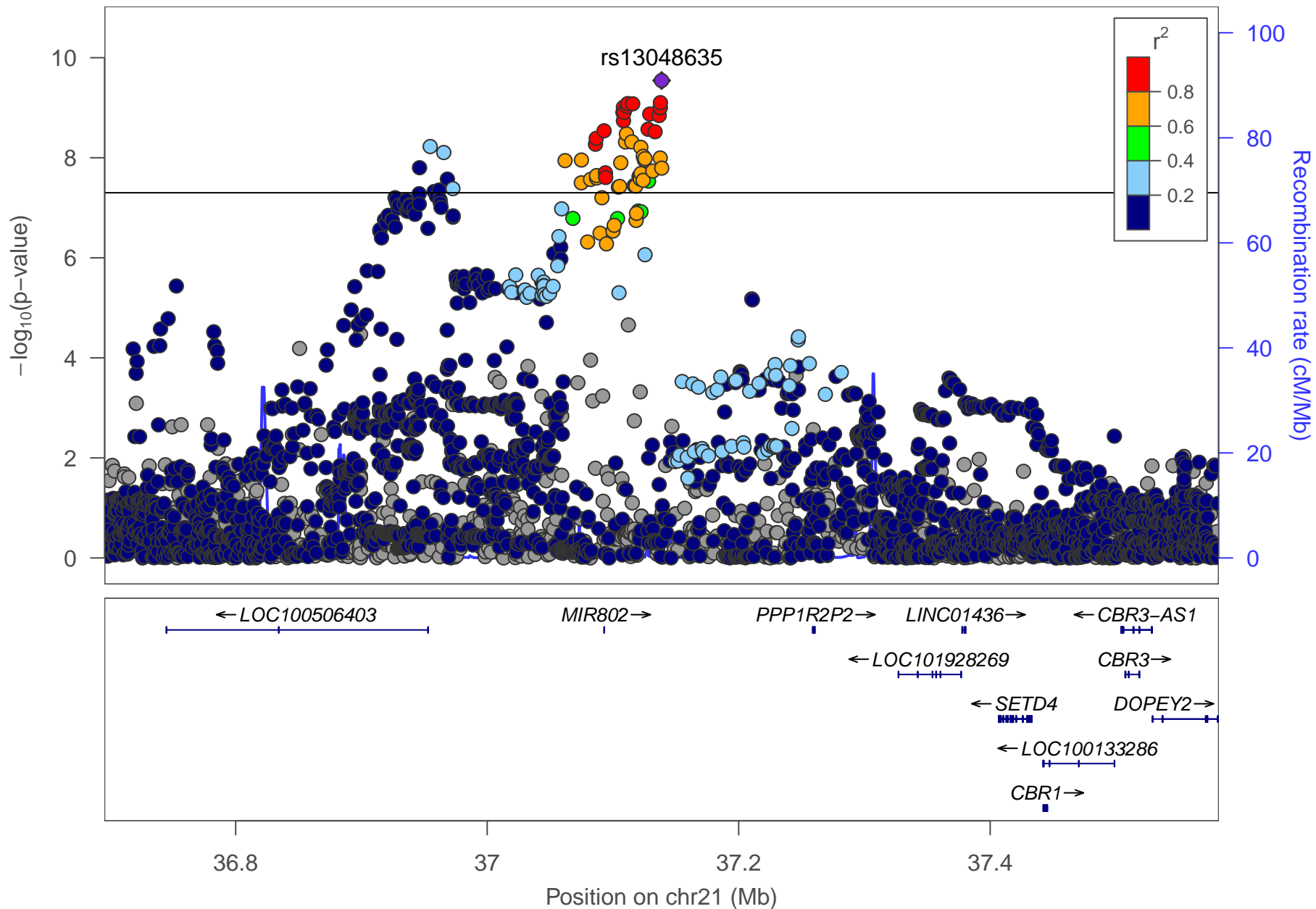

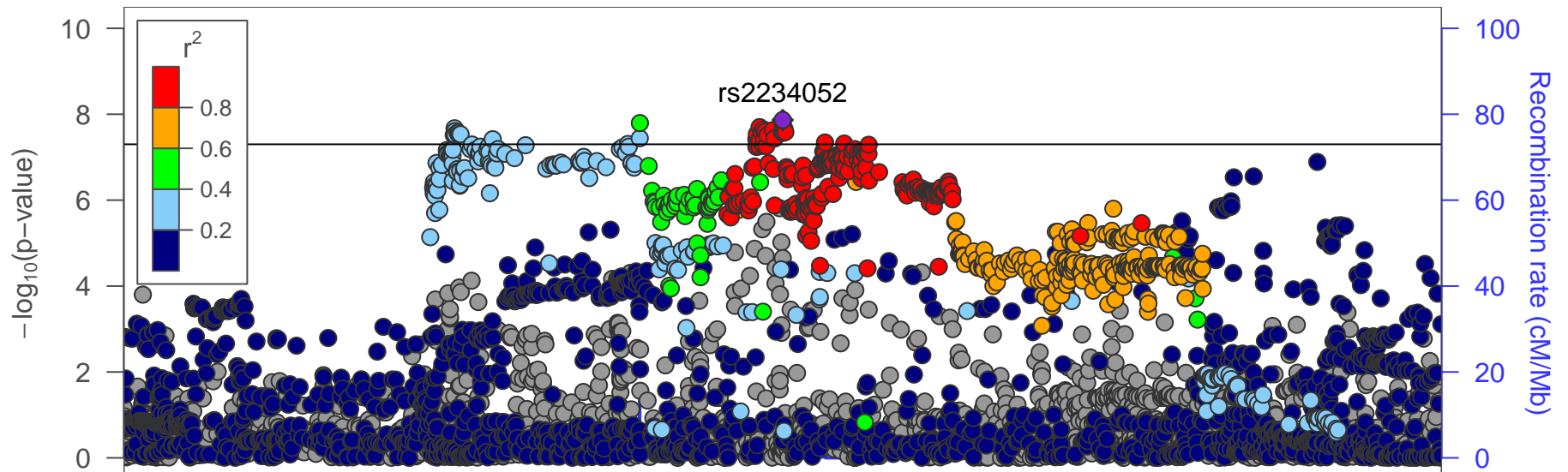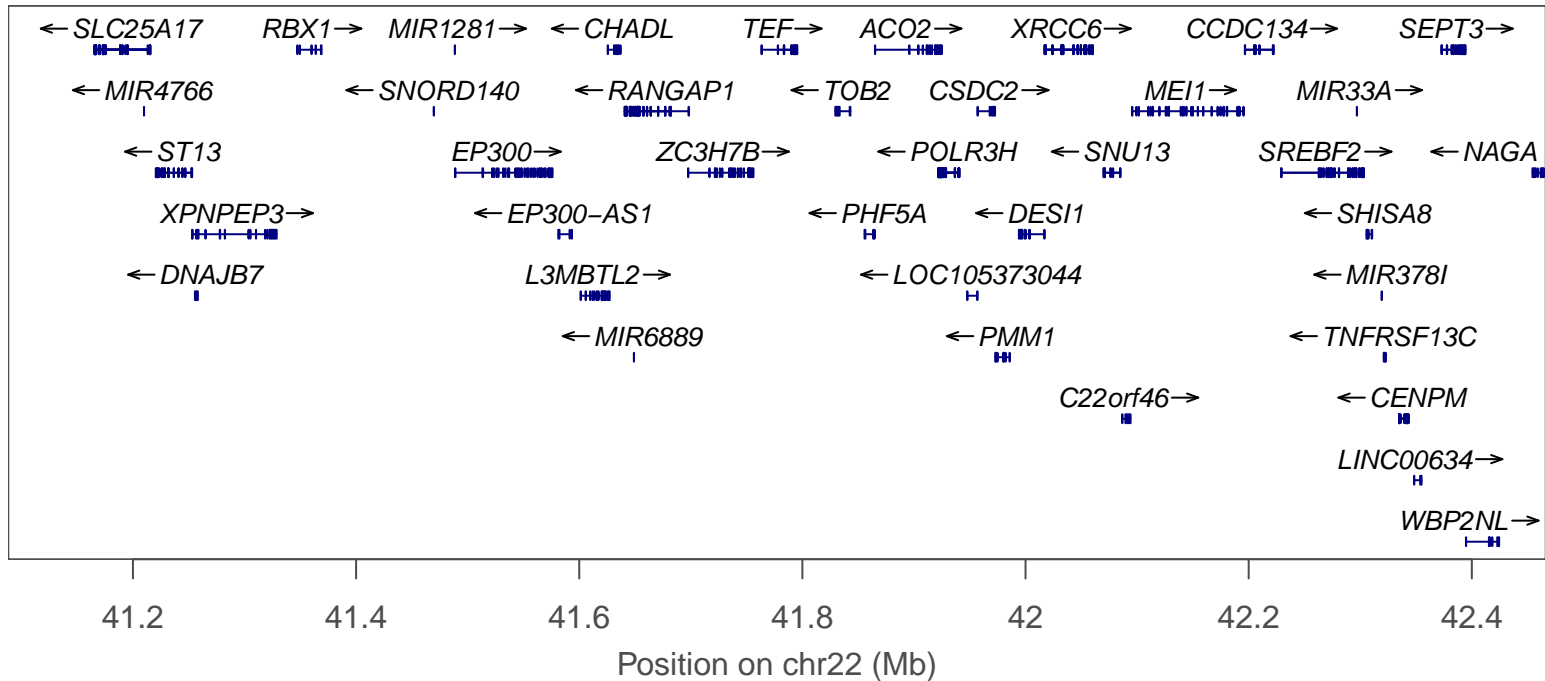

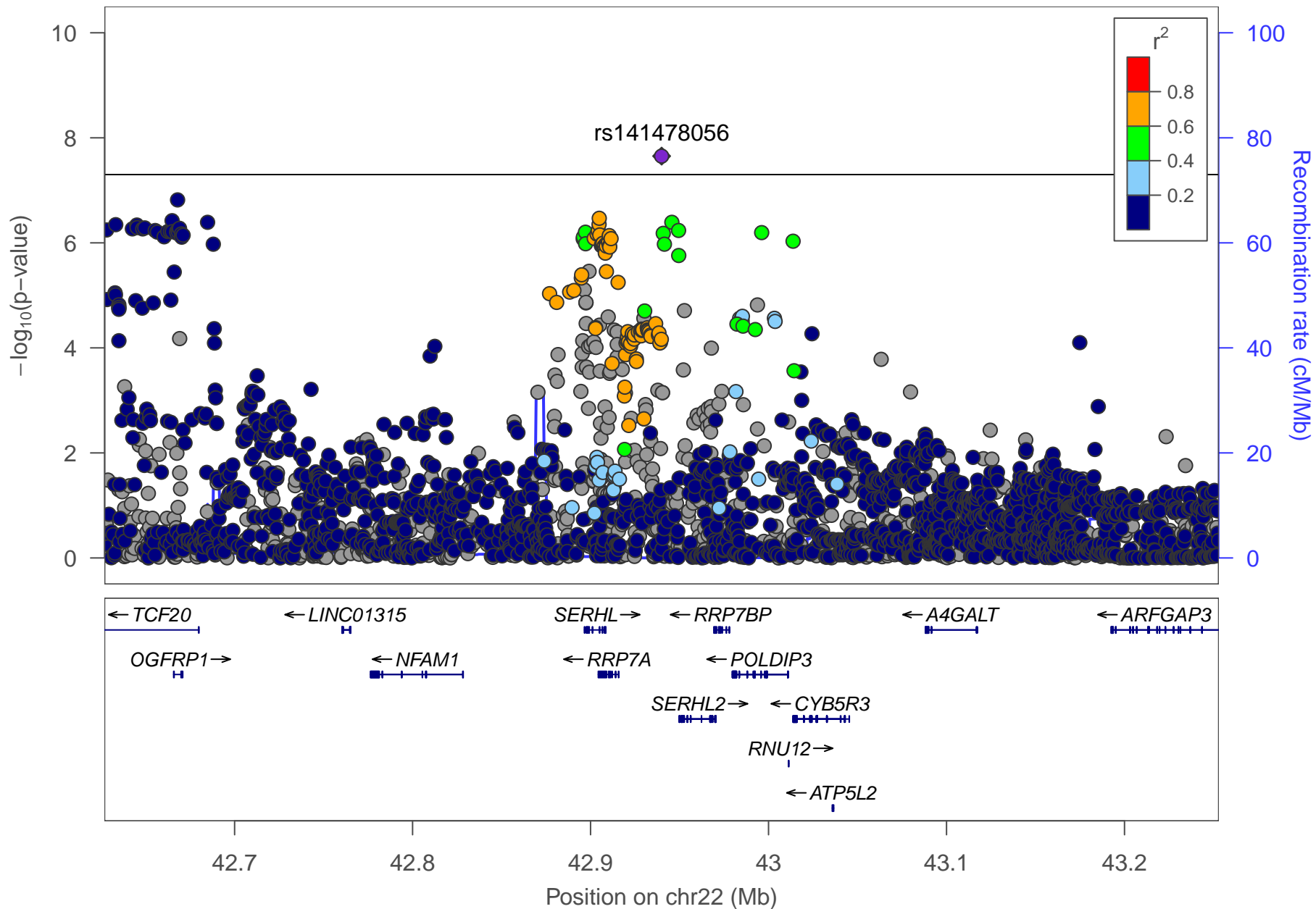

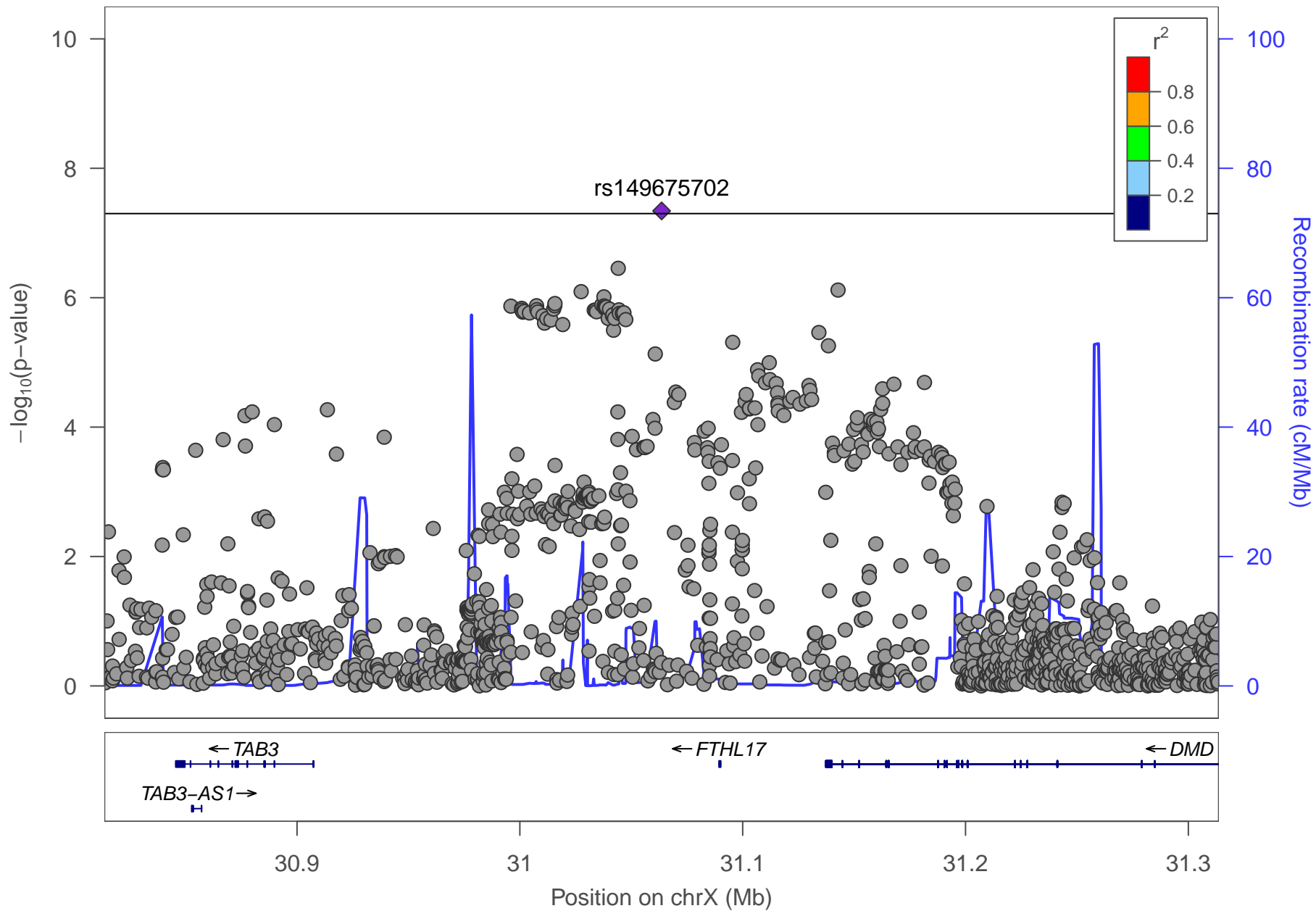

Supplement: Supplement 2 [file media-2.pdf]
