# Supplementary material for: Fine-mapping a genome-wide meta-analysis of 98,374 migraine cases identifies 181 sets of candidate causal variants
Source: medRxiv. 2024 May 20:2024.05.20.24307608. Preprint. [Version 1] doi: 10.1101/2024.05.20.24307608 (PMC11451805; doi:10.1101/2024.05.20.24307608)
Supplement: Supplement 4 [file media-4.pdf]

FinnGen Banner

Full Name	Affiliation	E-mail	Role 1	Role 2
Aarno Palotie	Institute for Molecular Medicine Finland (FIMM), HiLIFE, University of Helsinki, Helsinki,	aarno.palotie@helsinki.fi	Steering Committee	Steering Committee
Mark Daly	Institute for Molecular Medicine Finland (FIMM), HiLIFE, University of Helsinki, Helsinki,	mark.daly@helsinki.fi	Steering Committee	Steering Committee
Bridget Riley-Gills	Abbvie, Chicago, IL, United States	bridget.rileygillis@abbvie.com	Steering Committee	Pharmaceutical companies
Howard Jacob	Abbvie, Chicago, IL, United States	howard.jacob@abbvie.com	Steering Committee	Pharmaceutical companies
Dirk Paul	Astra Zeneca, Cambridge, United Kingdom	dirk.paul@astrazeneca.com	Steering Committee	Pharmaceutical companies
Slavé Petrovski	Astra Zeneca, Cambridge, United Kingdom	slav.petrovski@astrazeneca.com	Steering Committee	Pharmaceutical companies
Heiko Runz	Biogen, Cambridge, MA, United States	heiko.runz@biogen.com	Steering Committee	Pharmaceutical companies
Sally John	Biogen, Cambridge, MA, United States	sally.john@biogen.com	Steering Committee	Pharmaceutical companies
George Okafo	Boehringer Ingelheim, Ingelheim am Rhein, Germany	george.okafo@boehringer-ingelheim.com	Steering Committee	Pharmaceutical companies
Robert Plenge	Bristol Myers Squibb, New York, NY, United States	robert.plenge@bms.com	Steering Committee	Pharmaceutical companies
Joseph Maranville	Bristol Myers Squibb, New York, NY, United States	joseph.maranville@bms.com	Steering Committee	Pharmaceutical companies
Mark McCarthy	Genentech, San Francisco, CA, United States	mccarthy.mark@gene.com	Steering Committee	Pharmaceutical companies
Margaret G. Ehm	GlaxoSmithKline, Collegeville, PA, United States	meg.g.ehm@gsk.com	Steering Committee	Pharmaceutical companies
Kirsi Auro	GlaxoSmithKline, Espoo, Finland	kirsi.m.auro@gsk.com	Steering Committee	Pharmaceutical companies
Simonne Longerich	Merck, Kenilworth, NJ, United States	simonne.longerich@merck.com	Steering Committee	Pharmaceutical companies
Anders Mälarstig	Pfizer, New York, NY, United States	anders.malarstig@pfizer.com	Steering Committee	Pharmaceutical companies
Katherine Klinger	Translational Sciences, Sanofi R&D, Framingham, MA, USA	katherine.klinger@sanofi.com	Steering Committee	Pharmaceutical companies
Clement Chatelain	Translational Sciences, Sanofi R&D, Framingham, MA, USA	clement.chatelain@sanofi.com	Steering Committee	Pharmaceutical companies
Matthias Gossel	Translational Sciences, Sanofi R&D, Framingham, MA, USA	matthias.gossel@sanofi.com	Steering Committee	Pharmaceutical companies
Karol Estrada	Maze Therapeutics, San Francisco, CA, United States	kestrada@mazetx.com	Steering Committee	Pharmaceutical companies
Robert Graham	Maze Therapeutics, San Francisco, CA, United States	rgraham@mazetx.com	Steering Committee	Pharmaceutical companies
Dawn Waterworth	Janssen Research & Development, LLC, Spring House, PA, United States	dwaterwo@its.jnj.com	Steering Committee	Pharmaceutical companies
Chris O´Donnell	Novartis Institutes for BioMedical Research, Cambridge, MA, United States	chris.odonnell@novartis.com	Steering Committee	Pharmaceutical companies
Nicole Renaud	Novartis Institutes for BioMedical Research, Cambridge, MA, United States	nicole.renaud@novartis.com	Steering Committee	Pharmaceutical companies
Tomi P. Mäkelä	HiLIFE, University of Helsinki, Finland, Finland	tomi.makela@helsinki.fi	Steering Committee	University of Helsinki & Biobanks
Jaakko Kaprio	Institute for Molecular Medicine Finland (FIMM), HiLIFE, University of Helsinki, Helsinki,	jaakko.kaprio@helsinki.fi	Steering Committee	University of Helsinki & Biobanks
Petri Virolainen	Auria Biobank / University of Turku / Hospital District of Southwest Finland, Turku,	petri.virolainen@tyks.fi	Steering Committee	University of Helsinki & Biobanks
Antti Hakanen	Auria Biobank / University of Turku / Hospital District of Southwest Finland, Turku,	antti.hakanen@tyks.fi	Steering Committee	University of Helsinki & Biobanks
Terhi Kilpi	THL Biobank / Finnish Institute for Health and Welfare (THL), Helsinki, Finland	terhi.kilpi@thl.fi	Steering Committee	University of Helsinki & Biobanks
Markus Perola	THL Biobank / Finnish Institute for Health and Welfare (THL), Helsinki, Finland	markus.perola@thl.fi	Steering Committee	University of Helsinki & Biobanks
Jukka Partanen	Finnish Red Cross Blood Service / Finnish Hematology Registry and Clinical Biobank,	jukka.partanen@veripalvelu.fi	Steering Committee	University of Helsinki & Biobanks
Anne Pitkäranta	Helsinki Biobank / Helsinki University and Hospital District of Helsinki and Uusimaa,	anne.pitkaranta@hus.fi	Steering Committee	University of Helsinki & Biobanks
Taneli Raivio	Helsinki Biobank / Helsinki University and Hospital District of Helsinki and Uusimaa,	taneli.raivio@hus.fi	Steering Committee	University of Helsinki & Biobanks
Jani Tikkanen	Northern Finland Biobank Borealis / University of Oulu / Northern Ostrobothnia Hospital	jani.tikkanen@ppshp.fi	Steering Committee	University of Helsinki & Biobanks
Raisa Serpi	Northern Finland Biobank Borealis / University of Oulu / Northern Ostrobothnia Hospital	raisa.serpi@ppshp.fi	Steering Committee	University of Helsinki & Biobanks
Tarja Laitinen	Finnish Clinical Biobank Tampere / University of Tampere / Pirkanmaa Hospital District,	tarja.laitinen@pshp.fi	Steering Committee	University of Helsinki & Biobanks
Veli-Matti Kosma	Biobank of Eastern Finland / University of Eastern Finland / Northern Savo Hospital	veli-matti.kosma@uef.fi	Steering Committee	University of Helsinki & Biobanks
Jari Laukkanen	Central Finland Biobank / University of Jyväskylä / Central Finland Health Care District,	jari.laukkanen@ksshp.fi	Steering Committee	University of Helsinki & Biobanks
Marco Hautalahti	FINBB - Finnish biobank cooperative	marco.hautalahti@finbb.fi	Steering Committee	University of Helsinki & Biobanks
Outi Tuovila	Business Finland, Helsinki, Finland	outi.tuovila@businessfinland.fi	Steering Committee	Other Experts/ Non-Voting Members
Raimo Pakkanen	Business Finland, Helsinki, Finland	raimo.pakkanen@businessfinland.fi	Steering Committee	Other Experts/ Non-Voting Members
Jeffrey Waring	Abbvie, Chicago, IL, United States	jeff.waring@abbvie.com	Scientific Committee	Pharmaceutical companies
Bridget Riley-Gillis	Abbvie, Chicago, IL, United States	bridget.rileygillis@abbvie.com	Scientific Committee	Pharmaceutical companies
Fedik Rahimov	Abbvie, Chicago, IL, United States	fedik.rahimov@abbvie.com	Scientific Committee	Pharmaceutical companies
Ioanna Tachmazidou	Astra Zeneca, Cambridge, United Kingdom	ioanna.tachmazidou@astrazeneca.com	Scientific Committee	Pharmaceutical companies
Chia-Yen Chen	Biogen, Cambridge, MA, United States	chiayen.chen@biogen.com	Scientific Committee	Pharmaceutical companies
Heiko Runz	Biogen, Cambridge, MA, United States	heiko.runz@biogen.com	Scientific Committee	Pharmaceutical companies
Zhihao Ding	Boehringer Ingelheim, Ingelheim am Rhein, Germany	zhihao.ding@boehringer-ingelheim.com	Scientific Committee	Pharmaceutical companies
Marc Jung	Boehringer Ingelheim, Ingelheim am Rhein, Germany	marc_oliver.jung@boehringer-ingelheim.cc	Scientific Committee	Pharmaceutical companies
Hanati Tuoken	Boehringer Ingelheim, Ingelheim am Rhein, Germany	hanati.tuoken@boehringer-ingelheim.com	Scientific Committee	Pharmaceutical companies
Shameek Biswas	Bristol Myers Squibb, New York, NY, United States	Shameek.Biswas@bms.com	Scientific Committee	Pharmaceutical companies
Rion Pendergrass	Genentech, San Francisco, CA, United States	penders2@gene.com	Scientific Committee	Pharmaceutical companies
Margaret G. Ehm	GlaxoSmithKline, Collegeville, PA, United States	meg.g.ehm@gsk.com	Scientific Committee	Pharmaceutical companies
David Pulford	GlaxoSmithKline, Stevenage, United Kingdom	david.x.pulford@gsk.com	Scientific Committee	Pharmaceutical companies
Neha Raghavan	Merck, Kenilworth, NJ, United States	neha.raghavan@merck.com	Scientific Committee	Pharmaceutical companies
Adriana Huertas-Vazque	Merck, Kenilworth, NJ, United States	adriana.huertas.vazquez@merck.com	Scientific Committee	Pharmaceutical companies
Jae-Hoon Sul	Merck, Kenilworth, NJ, United States	jae.hoon.sul@merck.com	Scientific Committee	Pharmaceutical companies
Anders Mälarstig	Pfizer, New York, NY, United States	anders.malarstig@pfizer.com	Scientific Committee	Pharmaceutical companies
Xinli Hu	Pfizer, New York, NY, United States	xinli.hu@pfizer.com	Scientific Committee	Pharmaceutical companies
Åsa Hedman	Pfizer, New York, NY, United States	asa.hedman@pfizer.com	Scientific Committee	Pharmaceutical companies
Katherine Klinger	Translational Sciences, Sanofi R&D, Framingham, MA, USA	katherine.klinger@sanofi.com	Scientific Committee	Pharmaceutical companies
Robert Graham	Maze Therapeutics, San Francisco, CA, United States	rgraham@mazetx.com	Scientific Committee	Pharmaceutical companies
Manuel Rivas	Maze Therapeutics, San Francisco, CA, United States	mrivas@mazetx.com	Scientific Committee	Pharmaceutical companies
Dawn Waterworth	Janssen Research & Development, LLC, Spring House, PA, United States	dwaterwo@its.jnj.com	Scientific Committee	Pharmaceutical companies
Nicole Renaud	Novartis Institutes for BioMedical Research, Cambridge, MA, United States	nicole.renaud@novartis.com	Scientific Committee	Pharmaceutical companies
Ma´en Obeidat	Novartis Institutes for BioMedical Research, Cambridge, MA, United States	maen.obeidat@novartis.com	Scientific Committee	Pharmaceutical companies
Jonathan Chung	Novartis Institutes for BioMedical Research, Cambridge, MA, United States	jonathan.chung@novartis.com	Scientific Committee	Pharmaceutical companies
Jonas Zierer	Novartis Institutes for BioMedical Research, Cambridge, MA, United States	jonas.zierer@novartis.com	Scientific Committee	Pharmaceutical companies
Mari Niemi	Novartis Institutes for BioMedical Research, Cambridge, MA, United States	mari.niemi@novartis.com	Scientific Committee	Pharmaceutical companies
Samuli Ripatti	Institute for Molecular Medicine Finland (FIMM), HiLIFE, University of Helsinki, Helsinl	samuli.ripatti@helsinki.fi	Scientific Committee	University of Helsinki & Biobanks
Johanna Schleutker	Auria Biobank / Univ. of Turku / Hospital District of Southwest Finland, Turku, Finland	johanna.schleutker@utu.fi	Scientific Committee	University of Helsinki & Biobanks
Markus Perola	THL Biobank / Finnish Institute for Health and Welfare (THL), Helsinki, Finland	markus.perola@thl.fi	Scientific Committee	University of Helsinki & Biobanks
Mikko Arvas	Finnish Red Cross Blood Service / Finnish Hematology Registry and Clinical Biobank,	mikko.arvas@veripalvelu.fi	Scientific Committee	University of Helsinki & Biobanks
Olli Carpén	Helsinki Biobank / Helsinki University and Hospital District of Helsinki and Uusimaa,	olli.carpen@helsinki.fi	Scientific Committee	University of Helsinki & Biobanks
Reetta Hinttala	Northern Finland Biobank Borealis / University of Oulu / Northern Ostrobothnia Hospital	reetta.hinttala@oulu.fi	Scientific Committee	University of Helsinki & Biobanks
Johannes Kettunen	Northern Finland Biobank Borealis / University of Oulu / Northern Ostrobothnia Hospital	johannes.kettunen@oulu.fi	Scientific Committee	University of Helsinki & Biobanks
Arto Mannermaa	Biobank of Eastern Finland / University of Eastern Finland / Northern Savo Hospital	arto.mannermaa@uef.fi	Scientific Committee	University of Helsinki & Biobanks
Katriina Aalto-Setälä	Faculty of Medicine and Health Technology, Tampere University, Tampere, Finland	katriina.aalto-setala@tuni.fi	Scientific Committee	University of Helsinki & Biobanks
Mika Kähönen	Finnish Clinical Biobank Tampere / University of Tampere / Pirkanmaa Hospital District,	mika.kahonen@uta.fi	Scientific Committee	University of Helsinki & Biobanks
Jari Laukkanen	Central Finland Biobank / University of Jyväskylä / Central Finland Health Care District,	jari.laukkanen@ksshp.fi	Scientific Committee	University of Helsinki & Biobanks
Johanna Mäkelä	FINBB - Finnish biobank cooperative	johanna.makela@finbb.fi	Scientific Committee	University of Helsinki & Biobanks
Reetta Kälviäinen	Northern Savo Hospital District, Kuopio, Finland	reetta.kalviainen@kuh.fi	Clinical Groups	Neurology Group
Valtteri Julkunen	Northern Savo Hospital District, Kuopio, Finland	valtteri.julkunen@kuh.fi	Clinical Groups	Neurology Group
Hilkka Soininen	Northern Savo Hospital District, Kuopio, Finland	hilkka.soininen@uef.fi	Clinical Groups	Neurology Group
Anne Remes	Northern Ostrobothnia Hospital District, Oulu, Finland	anne.remes@oulu.fi	Clinical Groups	Neurology Group
Mikko Hiltunen	University of Eastern Finland, Kuopio, Finland	mikko.hiltunen@uef.fi	Clinical Groups	Neurology Group
Jukka Peltola	Pirkanmaa Hospital District, Tampere, Finland	jukka.peltola@pshp.fi	Clinical Groups	Neurology Group
Minna Raivio	Hospital District of Helsinki and Uusimaa, Helsinki, Finland	minna.raivio@geri.fi	Clinical Groups	Neurology Group
Pentti Tienari	Hospital District of Helsinki and Uusimaa, Helsinki, Finland	pentti.tienari@hus.fi	Clinical Groups	Neurology Group
Juha Rinne	Hospital District of Southwest Finland, Turku, Finland	juha.rinne@tyks.fi	Clinical Groups	Neurology Group
Roosa Kallionpää	Hospital District of Southwest Finland, Turku, Finland	roosa.kallionpaa@tyks.fi	Clinical Groups	Neurology Group
Juulia Partanen	Institute for Molecular Medicine Finland, HiLIFE, University of Helsinki, Finland	juulia.partanen@helsinki.fi	Clinical Groups	Neurology Group
Adam Ziemann	Abbvie, Chicago, IL, United States	adam.ziemann@abbvie.com	Clinical Groups	Neurology Group
Nizar Smaoui	Abbvie, Chicago, IL, United States	nizar.smaoui@abbvie.com	Clinical Groups	Neurology Group
Anne Lehtonen	Abbvie, Chicago, IL, United States	anne.lehtonen@abbvie.com	Clinical Groups	Neurology Group
Susan Eaton	Biogen, Cambridge, MA, United States	susan.eaton@biogen.com	Clinical Groups	Neurology Group
Heiko Runz	Biogen, Cambridge, MA, United States	heiko.runz@biogen.com	Clinical Groups	Neurology Group
Sanni Lahdenperä	Biogen, Cambridge, MA, United States	sanni.lahdenpera@biogen.com	Clinical Groups	Neurology Group
Shameek Biswas	Bristol Myers Squibb, New York, NY, United States	shameek.biswas@bms.com	Clinical Groups	Neurology Group
Natalie Bowers	Genentech, San Francisco, CA, United States	bowersn1@gene.com	Clinical Groups	Neurology Group
Edmond Teng	Genentech, San Francisco, CA, United States	teng.edmond@gene.com	Clinical Groups	Neurology Group
Rion Pendergrass	Genentech, San Francisco, CA, United States	penders2@gene.com	Clinical Groups	Neurology Group

Fanli Xu	GlaxoSmithKline, Brentford, United Kingdom	chun-fang.2.xu@gsk.com	Clinical Groups	Neurology Group
David Pulford	GlaxoSmithKline, Stevenage, United Kingdom	david.x.pulford@gsk.com	Clinical Groups	Neurology Group
Kirsi Auro	GlaxoSmithKline, Espoo, Finland	kirsi.m.auro@gsk.com	Clinical Groups	Neurology Group
Laura Addis	GlaxoSmithKline, Brentford, United Kingdom	laura.x.addis@gsk.com	Clinical Groups	Neurology Group
John Eicher	GlaxoSmithKline, Brentford, United Kingdom	john.d.eicher@gsk.com	Clinical Groups	Neurology Group
Qingqin S Li	Janssen Research & Development, LLC, Titusville, NJ 08560, United States	QLi2@its.jnj.com	Clinical Groups	Neurology Group
Karen He	Janssen Research & Development, LLC, Spring House, PA, United States	khe2@its.jnj.com	Clinical Groups	Neurology Group
Ekaterina Khramtsova	Janssen Research & Development, LLC, Spring House, PA, United States	ekhramts@its.jnj.com	Clinical Groups	Neurology Group
Neha Raghavan	Merck, Kenilworth, NJ, United States	neha.raghavan@merck.com	Clinical Groups	Neurology Group
Martti Färkkilä	Hospital District of Helsinki and Uusimaa, Helsinki, Finland	martti.farkkila@hus.fi	Clinical Groups	Gastroenterology Group
Jukka Koskela	Hospital District of Helsinki and Uusimaa, Helsinki, Finland	jukka.koskela@helsinki.fi	Clinical Groups	Gastroenterology Group
Sampsa Pikkarainen	Hospital District of Helsinki and Uusimaa, Helsinki, Finland	sampsa.pikkarainen@hus.fi	Clinical Groups	Gastroenterology Group
Airi Jussila	Pirkanmaa Hospital District, Tampere, Finland	airi.jussila@pshp.fi	Clinical Groups	Gastroenterology Group
Katri Kaukinen	Pirkanmaa Hospital District, Tampere, Finland	katri.kaukinen@tuni.fi	Clinical Groups	Gastroenterology Group
Timo Blomster	Northern Ostrobothnia Hospital District, Oulu, Finland	timo.blomster@ppshp.fi	Clinical Groups	Gastroenterology Group
Mikko Kiviniemi	Northern Savo Hospital District, Kuopio, Finland	mikko.kiviniemi@kuh.fi	Clinical Groups	Gastroenterology Group
Markku Voutilainen	Hospital District of Southwest Finland, Turku, Finland	markku.voutilainen@tyks.fi	Clinical Groups	Gastroenterology Group
Mark Daly	Institute for Molecular Medicine, Finland (FIMM), HiLIFE, University of Helsinki, Helsinki, Finland; Broad Institute of MIT and Harvard; Massachusetts General Hospital	mark.daly@helsinki.fi	Clinical Groups	Gastroenterology Group
Jeffrey Waring	Abbvie, Chicago, IL, United States	jeff.waring@abbvie.com	Clinical Groups	Gastroenterology Group
Nizar Smaoui	Abbvie, Chicago, IL, United States	nizar.smaoui@abbvie.com	Clinical Groups	Gastroenterology Group
Fedik Rahimov	Abbvie, Chicago, IL, United States	fedik.rahimov@abbvie.com	Clinical Groups	Gastroenterology Group
Anne Lehtonen	Abbvie, Chicago, IL, United States	anne.lehtonen@abbvie.com	Clinical Groups	Gastroenterology Group
Tim Lu	Genentech, San Francisco, CA, United States	lut8@gene.com	Clinical Groups	Gastroenterology Group
Natalie Bowers	Genentech, San Francisco, CA, United States	bowersn1@gene.com	Clinical Groups	Gastroenterology Group
Rion Pendergrass	Genentech, San Francisco, CA, United States	penders2@gene.com	Clinical Groups	Gastroenterology Group
Linda McCarthy	GlaxoSmithKline, Brentford, United Kingdom	linda.c.mccarthy@gsk.com	Clinical Groups	Gastroenterology Group
Amy Hart	Janssen Research & Development, LLC, Spring House, PA, United States	ahart13@its.jnj.com	Clinical Groups	Gastroenterology Group
Meijian Guan	Janssen Research & Development, LLC, Spring House, PA, United States	mguan4@its.jnj.com	Clinical Groups	Gastroenterology Group
Jason Miller	Merck, Kenilworth, NJ, United States	jason.miller4@merck.com	Clinical Groups	Gastroenterology Group
Kirsi Kalpala	Pfizer, New York, NY, United States	kirsi.kalpala@pfizer.com	Clinical Groups	Gastroenterology Group
Melissa Miller	Pfizer, New York, NY, United States	melissa.r.miller@pfizer.com	Clinical Groups	Gastroenterology Group
Xinli Hu	Pfizer, New York, NY, United States	xinli.hu@pfizer.com	Clinical Groups	Gastroenterology Group
Kari Eklund	Hospital District of Helsinki and Uusimaa, Helsinki, Finland	kari.eklund@hus.fi	Clinical Groups	Rheumatology Group
Antti Palomäki	Hospital District of Southwest Finland, Turku, Finland	ajpalo@utu.fi	Clinical Groups	Rheumatology Group
Pia Isomäki	Pirkanmaa Hospital District, Tampere, Finland	pia.isomaki@pshp.fi	Clinical Groups	Rheumatology Group
Laura Pirilä	Hospital District of Southwest Finland, Turku, Finland	laura.pirila@fimnet.fi,laura.pirila@tyks.fi	Clinical Groups	Rheumatology Group
Oili Kaipiainen-Seppänen	Northern Savo Hospital District, Kuopio, Finland	oili.kaipiainen-seppanen@kuh.fi	Clinical Groups	Rheumatology Group
Johanna Huhtakangas	Northern Ostrobothnia Hospital District, Oulu, Finland	johanna.huhtakangas@kuh.fi	Clinical Groups	Rheumatology Group
Nina Mars	Institute for Molecular Medicine Finland (FIMM), HiLIFE, University of Helsinki, Helsink	nina.mars@helsinki.fi	Clinical Groups	Rheumatology Group
Jeffrey Waring	Abbvie, Chicago, IL, United States	jeff.waring@abbvie.com	Clinical Groups	Rheumatology Group
Fedik Rahimov	Abbvie, Chicago, IL, United States	fedik.rahimov@abbvie.com	Clinical Groups	Rheumatology Group
Apinya Lertratanakul	Abbvie, Chicago, IL, United States	apinya.lertratanakul@abbvie.com	Clinical Groups	Rheumatology Group
Nizar Smaoui	Abbvie, Chicago, IL, United States	nizar.smaoui@abbvie.com	Clinical Groups	Rheumatology Group
Anne Lehtonen	Abbvie, Chicago, IL, United States	anne.lehtonen@abbvie.com	Clinical Groups	Rheumatology Group
Coralie Viollet	AstraZeneca, Cambridge, United Kingdom	coralie.viollet@astrazeneca.com	Clinical Groups	Rheumatology Group
Marla Hochfeld	Bristol Myers Squibb, New York, NY, United States	mhochfeld@celgene.com	Clinical Groups	Rheumatology Group
Natalie Bowers	Genentech, San Francisco, CA, United States	bowersn1@gene.com	Clinical Groups	Rheumatology Group
Rion Pendergrass	Genentech, San Francisco, CA, United States	penders2@gene.com	Clinical Groups	Rheumatology Group
Jorge Esparza Gordillo	GlaxoSmithKline, Brentford, United Kingdom	jorge.x.esparza-gordillo@gsk.com	Clinical Groups	Rheumatology Group
Kirsi Auro	GlaxoSmithKline, Espoo, Finland	kirsi.m.auro@gsk.com	Clinical Groups	Rheumatology Group
Dawn Waterworth	Janssen Research & Development, LLC, Spring House, PA, United States	dwaterwo@its.jnj.com	Clinical Groups	Rheumatology Group
Fabiana Farias	Merck, Kenilworth, NJ, United States	fabiana.farias@merck.com	Clinical Groups	Rheumatology Group
Kirsi Kalpala	Pfizer, New York, NY, United States	kirsi.kalpala@pfizer.com	Clinical Groups	Rheumatology Group
Nan Bing	Pfizer, New York, NY, United States	nan.bing@pfizer.com	Clinical Groups	Rheumatology Group
Xinli Hu	Pfizer, New York, NY, United States	xinli.hu@pfizer.com	Clinical Groups	Rheumatology Group
Tarja Laitinen	Pirkanmaa Hospital District, Tampere, Finland	tarja.laitinen@pshp.fi	Clinical Groups	Pulmonology Group
Margit Pelkonen	Northern Savo Hospital District, Kuopio, Finland	margit.pelkonen@kuh.fi	Clinical Groups	Pulmonology Group
Paula Kauppi	Hospital District of Helsinki and Uusimaa, Helsinki, Finland	paula.kauppi@hus.fi	Clinical Groups	Pulmonology Group
Hannu Kankaanranta	University of Gothenburg, Gothenburg, Sweden/ Seinäjoki Central Hospital, Seinäjoki, Finland/ Tampere University, Tampere, Finland	hannu.kankaanranta@tuni.fi	Clinical Groups	Pulmonology Group
Terttu Harju	Northern Ostrobothnia Hospital District, Oulu, Finland	terttu.harju@oulu.fi	Clinical Groups	Pulmonology Group
Riitta Lahesmaa	Hospital District of Southwest Finland, Turku, Finland	rilahes@utu.fi	Clinical Groups	Pulmonology Group
Nizar Smaoui	Abbvie, Chicago, IL, United States	nizar.smaoui@abbvie.com	Clinical Groups	Pulmonology Group
Coralie Viollet	AstraZeneca, Cambridge, United Kingdom	coralie.viollet@astrazeneca.com	Clinical Groups	Pulmonology Group
Susan Eaton	Biogen, Cambridge, MA, United States	susan.eaton@biogen.com	Clinical Groups	Pulmonology Group
Hubert Chen	Genentech, San Francisco, CA, United States	chenh37@gene.com	Clinical Groups	Pulmonology Group
Rion Pendergrass	Genentech, San Francisco, CA, United States	penders2@gene.com	Clinical Groups	Pulmonology Group
Natalie Bowers	Genentech, San Francisco, CA, United States	bowersn1@gene.com	Clinical Groups	Pulmonology Group
Joanna Betts	GlaxoSmithKline, Brentford, United Kingdom	joanna.c.betts@gsk.com	Clinical Groups	Pulmonology Group
Kirsi Auro	GlaxoSmithKline, Espoo, Finland	kirsi.m.auro@gsk.com	Clinical Groups	Pulmonology Group
Rajashree Mishra	GlaxoSmithKline, Brentford, United Kingdom	rajashree.x.mishra@gsk.com	Clinical Groups	Pulmonology Group
Majd Mouded	Novartis, Basel, Switzerland	majd.mouded@novartis.com	Clinical Groups	Pulmonology Group
Debby Ngo	Novartis, Basel, Switzerland	debby.ngo@novartis.com	Clinical Groups	Pulmonology Group
Teemu Niiranen	Finnish Institute for Health and Welfare (THL), Helsinki, Finland	teemu.niiranen@thl.fi	Clinical Groups	Cardiometabolic Diseases Group
Felix Vaura	Finnish Institute for Health and Welfare (THL), Helsinki, Finland	fechva@utu.fi	Clinical Groups	Cardiometabolic Diseases Group
Veikko Salomaa	Finnish Institute for Health and Welfare (THL), Helsinki, Finland	veikko.salomaa@thl.fi	Clinical Groups	Cardiometabolic Diseases Group
Kaj Metsärinne	Hospital District of Southwest Finland, Turku, Finland	kaj.metsarinne@tyks.fi	Clinical Groups	Cardiometabolic Diseases Group
Jenni Aittokallio	Hospital District of Southwest Finland, Turku, Finland	jemato@utu.fi	Clinical Groups	Cardiometabolic Diseases Group
Mika Kähönen	Pirkanmaa Hospital District, Tampere, Finland	mika.kahonen@uta.fi	Clinical Groups	Cardiometabolic Diseases Group
Jussi Hernesniemi	Pirkanmaa Hospital District, Tampere, Finland	jussi.hernesniemi@tuni.fi	Clinical Groups	Cardiometabolic Diseases Group
Daniel Gordin	Hospital District of Helsinki and Uusimaa, Helsinki, Finland	daniel.gordin@hus.fi	Clinical Groups	Cardiometabolic Diseases Group
Juha Sinisalo	Hospital District of Helsinki and Uusimaa, Helsinki, Finland	juha.sinisalo@hus.fi	Clinical Groups	Cardiometabolic Diseases Group
Marja-Riitta Taskinen	Hospital District of Helsinki and Uusimaa, Helsinki, Finland	marja-riitta.taskinen@helsinki.fi	Clinical Groups	Cardiometabolic Diseases Group
Tiinamaija Tuomi	Hospital District of Helsinki and Uusimaa, Helsinki, Finland	tiinamaija.tuomi@hus.fi	Clinical Groups	Cardiometabolic Diseases Group
Timo Hiltunen	Hospital District of Helsinki and Uusimaa, Helsinki, Finland	timo.hiltunen@hus.fi	Clinical Groups	Cardiometabolic Diseases Group
Jari Laukkanen	Central Finland Health Care District, Jyväskylä, Finland	jari.laukkanen@ksshp.fi	Clinical Groups	Cardiometabolic Diseases Group
Amanda Elliott	Institute for Molecular Medicine Finland (FIMM), HiLIFE, University of Helsinki, Helsinki, Finland; Broad Institute, Cambridge, MA, USA and Massachusetts General Hospital, Boston, MA, USA	aelliott@broadinstitute.org	Clinical Groups	Cardiometabolic Diseases Group
Mary Pat Reeve	Institute for Molecular Medicine Finland (FIMM), HiLIFE, University of Helsinki, Helsink	mary.reeve@helsinki.fi	Clinical Groups	Cardiometabolic Diseases Group
Sanni Ruotsalainen	Institute for Molecular Medicine Finland (FIMM), HiLIFE, University of Helsinki, Helsink	sanni.ruotsalainen@helsinki.fi	Clinical Groups	Cardiometabolic Diseases Group
Dirk Paul	Astra Zeneca, Cambridge, United Kingdom	dirk.paul@astrazeneca.com	Clinical Groups	Cardiometabolic Diseases Group
Natalie Bowers	Genentech, San Francisco, CA, United States	bowersn1@gene.com	Clinical Groups	Cardiometabolic Diseases Group
Rion Pendergrass	Genentech, San Francisco, CA, United States	penders2@gene.com	Clinical Groups	Cardiometabolic Diseases Group
Audrey Chu	GlaxoSmithKline, Brentford, United Kingdom	audrey.y.chu@gsk.com	Clinical Groups	Cardiometabolic Diseases Group
Kirsi Auro	GlaxoSmithKline, Espoo, Finland	kirsi.m.auro@gsk.com	Clinical Groups	Cardiometabolic Diseases Group
Dermot Reilly	Janssen Research & Development, LLC, Boston, MA, United States	dreill11@its.jnj.com	Clinical Groups	Cardiometabolic Diseases Group
Mike Mendelson	Novartis, Boston, MA, United States	mike.mendelson@novartis.com	Clinical Groups	Cardiometabolic Diseases Group
Jaakko Parkkinen	Pfizer, New York, NY, United States	jaakko.parkkinen@pfizer.com	Clinical Groups	Cardiometabolic Diseases Group
Melissa Miller	Pfizer, New York, NY, United States	melissa.r.miller@pfizer.com	Clinical Groups	Cardiometabolic Diseases Group
Tuomo Meretoja	Hospital District of Helsinki and Uusimaa, Helsinki, Finland	tuomo.meretoja@hus.fi	Clinical Groups	Oncology Group
Heikki Joensuu	Hospital District of Helsinki and Uusimaa, Helsinki, Finland	heikki.joensuu@hus.fi	Clinical Groups	Oncology Group
Olli Carpén	Hospital District of Helsinki and Uusimaa, Helsinki, Finland	olli.carpen@helsinki.fi	Clinical Groups	Oncology Group
Johanna Mattson	Hospital District of Helsinki and Uusimaa, Helsinki, Finland	johanna.mattson@hus.fi	Clinical Groups	Oncology Group
Eveliina Salminen	Hospital District of Helsinki and Uusimaa, Helsinki, Finland	eveliina.e.salminen@hus.fi	Clinical Groups	Oncology Group
Annika Auranen	Pirkanmaa Hospital District , Tampere, Finland	anaura@utu.fi	Clinical Groups	Oncology Group
Peeter Karihtala	Northern Ostrobothnia Hospital District, Oulu, Finland	peeter.karihtala@oulu.fi	Clinical Groups	Oncology Group
Päivi Auvinen	Northern Savo Hospital District, Kuopio, Finland	paivi.auvinen@kuh.fi	Clinical Groups	Oncology Group
Klaus Elenius	Hospital District of Southwest Finland, Turku, Finland	klaus.elenius@utu.fi	Clinical Groups	Oncology Group
Johanna Schleutker	Hospital District of Southwest Finland, Turku, Finland	johanna.schleutker@utu.fi	Clinical Groups	Oncology Group
Esa Pitkänen	Institute for Molecular Medicine Finland (FIMM), HiLIFE, University of Helsinki, Helsink	esa.pitkanen@helsinki.fi	Clinical Groups	Oncology Group
Nina Mars	Institute for Molecular Medicine Finland (FIMM), HiLIFE, University of Helsinki, Helsink	nina.mars@helsinki.fi	Clinical Groups	Oncology Group
Mark Daly	Institute for Molecular Medicine Finland (FIMM), HiLIFE, University of Helsinki, Helsinki, Finland; Broad Institute of MIT and Harvard; Massachusetts General Hospital	mark.daly@helsinki.fi	Clinical Groups	Oncology Group
Relja Popovic	Abbvie, Chicago, IL, United States	relja.popovic@abbvie.com	Clinical Groups	Oncology Group
Jeffrey Waring	Abbvie, Chicago, IL, United States	jeff.waring@abbvie.com	Clinical Groups	Oncology Group
Bridget Riley-Gillis	Abbvie, Chicago, IL, United States	bridget.rileygillis@abbvie.com	Clinical Groups	Oncology Group
Anne Lehtonen	Abbvie, Chicago, IL, United States	anne.lehtonen@abbvie.com	Clinical Groups	Oncology Group
Margarete Fabre	AstraZeneca, Cambridge, United Kingdom	margarete.fabre@astrazeneca.com	Clinical Groups	Oncology Group
Jennifer Schutzman	Genentech, San Francisco, CA, United States	schutzman.jennifer@gene.com	Clinical Groups	Oncology Group
Natalie Bowers	Genentech, San Francisco, CA, United States	bowersn1@gene.com	Clinical Groups	Oncology Group

Rion Pendergrass	Genentech, San Francisco, CA, United States	penders2@gene.com	Clinical Groups	Oncology Group
Diptee Kulkarni	GlaxoSmithKline, Brentford, United Kingdom	diptee.a.kulkarni@gsk.com	Clinical Groups	Oncology Group
Kirsi Auro	GlaxoSmithKline, Espoo, Finland	kirsi.m.auro@gsk.com	Clinical Groups	Oncology Group
Alessandro Porello	Janssen Research & Development, LLC, Spring House, PA, United States	APorrell@ITS.JNJ.com	Clinical Groups	Oncology Group
Andrey Loboda	Merck, Kenilworth, NJ, United States	andrey_loboda@merck.com	Clinical Groups	Oncology Group
Heli Lehtonen	Pfizer, New York, NY, United States	heli.lehtonen@pfizer.com	Clinical Groups	Oncology Group
Stefan McDonough	Pfizer, New York, NY, United States	stefan.McDonough@pfizer.com	Clinical Groups	Oncology Group
Sauli Vuoti	Janssen-Cilag Oy, Espoo, Finland	svuoti@its.jnj.com	Clinical Groups	Oncology Group
Kai Kaarniranta	Northern Savo Hospital District, Kuopio, Finland; Department of Molecular Genetics, University of Lodz, Lodz, Poland	kai.kaarniranta@uef.fi	Clinical Groups	Opthalmology Group
Joni A Turunen	Helsinki University Hospital and University of Helsinki, Helsinki, Finland; Eye Genetics Group, Folkhälsan Research Center, Helsinki, Finland	joni.turunen@helsinki.fi	Clinical Groups	Opthalmology Group
Terhi Ollila	Hospital District of Helsinki and Uusimaa, Helsinki, Finland	terhi.ollila@hus.fi	Clinical Groups	Opthalmology Group
Hannu Uusitalo	Pirkanmaa Hospital District, Tampere, Finland	hannu.uusitalo@tuni.fi	Clinical Groups	Opthalmology Group
Juha Karjalainen	Institute for Molecular Medicine Finland (FIMM), HiLIFE, University of Helsinki, Helsink	juha.karjalainen@helsinki.fi	Clinical Groups	Opthalmology Group
Esa Pitkänen	Institute for Molecular Medicine Finland (FIMM), HiLIFE, University of Helsinki, Helsink	esa.pitkanen@helsinki.fi	Clinical Groups	Opthalmology Group
Mengzhen Liu	Abbvie, Chicago, IL, United States	mengzhen.liu@abbvie.com	Clinical Groups	Opthalmology Group
Heiko Runz	Biogen, Cambridge, MA, United States	heiko.runz@biogen.com	Clinical Groups	Opthalmology Group
Stephanie Loomis	Biogen, Cambridge, MA, United States	stephanie.loomis@biogen.com	Clinical Groups	Opthalmology Group
Erich Strauss	Genentech, San Francisco, CA, United States	strauss.erich@gene.com	Clinical Groups	Opthalmology Group
Natalie Bowers	Genentech, San Francisco, CA, United States	bowersn1@gene.com	Clinical Groups	Opthalmology Group
Hao Chen	Genentech, San Francisco, CA, United States	haoc@gene.com	Clinical Groups	Opthalmology Group
Rion Pendergrass	Genentech, San Francisco, CA, United States	penders2@gene.com	Clinical Groups	Opthalmology Group
Kaisa Tasanen	Northern Ostrobothnia Hospital District, Oulu, Finland	kaisa.tasanen-maatta@oulu.fi	Clinical Groups	Dermatology Group
Laura Huilaja	Northern Ostrobothnia Hospital District, Oulu, Finland	laura.huilaja@oulu.fi	Clinical Groups	Dermatology Group
Katariina Hannula-Jouppi	Hospital District of Helsinki and Uusimaa, Helsinki, Finland	katariina.hannula-jouppi@hus.fi	Clinical Groups	Dermatology Group
Teea Salmi	Pirkanmaa Hospital District, Tampere, Finland	teea.salmi@pshp.fi	Clinical Groups	Dermatology Group
Sirkku Peltonen	Hospital District of Southwest Finland, Turku, Finland	sipelto@utu.fi	Clinical Groups	Dermatology Group
Leena Koulu	Hospital District of Southwest Finland, Turku, Finland	leena.koulu@tyks.fi	Clinical Groups	Dermatology Group
Nizar Smaoui	Abbvie, Chicago, IL, United States	nizar.smaoui@abbvie.com	Clinical Groups	Dermatology Group
Fedik Rahimov	Abbvie, Chicago, IL, United States	fedik.rahimov@abbvie.com	Clinical Groups	Dermatology Group
Anne Lehtonen	Abbvie, Chicago, IL, United States	anne.lehtonen@abbvie.com	Clinical Groups	Dermatology Group
David Choy	Genentech, San Francisco, CA, United States	choy.david@gene.com	Clinical Groups	Dermatology Group
Rion Pendergrass	Genentech, San Francisco, CA, United States	penders2@gene.com	Clinical Groups	Dermatology Group
Dawn Waterworth	Janssen Research & Development, LLC, Spring House, PA, United States	dwaterwo@its.jnj.com	Clinical Groups	Dermatology Group
Kirsi Kalpala	Pfizer, New York, NY, United States	kirsi.kalpala@pfizer.com	Clinical Groups	Dermatology Group
Ying Wu	Pfizer, New York, NY, United States	ying.wu3@pfizer.com	Clinical Groups	Dermatology Group
Pirkko Pussinen	Hospital District of Helsinki and Uusimaa, Helsinki, Finland	pirkko.pussinen@helsinki.fi	Clinical Groups	Odontology Group
Aino Salminen	Hospital District of Helsinki and Uusimaa, Helsinki, Finland	aino.m.salminen@helsinki.fi	Clinical Groups	Odontology Group
Tuula Salo	Hospital District of Helsinki and Uusimaa, Helsinki, Finland	tuula.salo@helsinki.fi	Clinical Groups	Odontology Group
David Rice	Hospital District of Helsinki and Uusimaa, Helsinki, Finland	david.rice@helsinki.fi	Clinical Groups	Odontology Group
Pekka Nieminen	Hospital District of Helsinki and Uusimaa, Helsinki, Finland	pekka.nieminen@helsinki.fi	Clinical Groups	Odontology Group
Ulla Palotie	Hospital District of Helsinki and Uusimaa, Helsinki, Finland	ulla.palotie@helsinki.fi	Clinical Groups	Odontology Group
Maria Siponen	Northern Savo Hospital District, Kuopio, Finland	maria.siponen@uef.fi	Clinical Groups	Odontology Group
Liisa Suominen	Northern Savo Hospital District, Kuopio, Finland	liisa.suominen@uef.fi	Clinical Groups	Odontology Group
Päivi Mäntylä	Northern Savo Hospital District, Kuopio, Finland	paivi.mantyla@uef.fi	Clinical Groups	Odontology Group
Ulvi Gursoy	Hospital District of Southwest Finland, Turku, Finland	ulvi.gursoy@utu.fi	Clinical Groups	Odontology Group
Vuokko Anttonen	Northern Ostrobothnia Hospital District, Oulu, Finland	vuokko.anttonen@oulu.fi	Clinical Groups	Odontology Group
Kirsi Sipilä	Research Unit of Oral Health Sciences Faculty of Medicine, University of Oulu, Oulu, Finland; Medical Research Center, Oulu, Oulu University Hospital and University of Oulu, Oulu, Finland	kirsi.sipila@oulu.fi	Clinical Groups	Odontology Group
Rion Pendergrass	Genentech, San Francisco, CA, United States	pendergrass.sarah@gene.com	Clinical Groups	Odontology Group
Hannele Laivuori	Institute for Molecular Medicine Finland (FIMM), HiLIFE, University of Helsinki, Helsink	hannele.laivuori@helsinki.fi	Clinical Groups	Women's Health and Reproduction Group
Venla Kurra	Pirkanmaa Hospital District, Tampere, Finland	venla.kurra@tuni.fi	Clinical Groups	Women's Health and Reproduction Group
Laura Kotaniemi-Talonen	Pirkanmaa Hospital District, Tampere, Finland	laura.kotaniemi-talonen@tuni.fi	Clinical Groups	Women's Health and Reproduction Group
Oskari Heikinheimo	Hospital District of Helsinki and Uusimaa, Helsinki, Finland	oskari.heikinheimo@helsinki.fi	Clinical Groups	Women's Health and Reproduction Group
Ilkka Kalliala	Hospital District of Helsinki and Uusimaa, Helsinki, Finland	ilkka.kalliala@hus.fi	Clinical Groups	Women's Health and Reproduction Group
Lauri Aaltonen	Hospital District of Helsinki and Uusimaa, Helsinki, Finland	lauri.aaltonen@helsinki.fi	Clinical Groups	Women's Health and Reproduction Group
Varpu Jokimaa	Hospital District of Southwest Finland, Turku, Finland	varpu.jokimaa@utu.fi	Clinical Groups	Women's Health and Reproduction Group
Johannes Kettunen	Northern Ostrobothnia Hospital District, Oulu, Finland	Johannes.Kettunen@oulu.fi	Clinical Groups	Women's Health and Reproduction Group
Marja Vääräsmäki	Northern Ostrobothnia Hospital District, Oulu, Finland	marja.vaarasmaki@oulu.fi	Clinical Groups	Women's Health and Reproduction Group
Outi Uimari	Northern Ostrobothnia Hospital District, Oulu, Finland	outi.uimari@oulu.fi	Clinical Groups	Women's Health and Reproduction Group
Laure Morin-Papunen	Northern Ostrobothnia Hospital District, Oulu, Finland	lmp@cc.oulu.fi	Clinical Groups	Women's Health and Reproduction Group
Maarit Niinimäki	Northern Ostrobothnia Hospital District, Oulu, Finland	maarit.niinimaki@oulu.fi	Clinical Groups	Women's Health and Reproduction Group
Terhi Piltonen	Northern Ostrobothnia Hospital District, Oulu, Finland	terhi.piltonen@oulu.fi	Clinical Groups	Women's Health and Reproduction Group
Katja Kivinen	Institute for Molecular Medicine Finland (FIMM), HiLIFE, University of Helsinki, Helsink	katja.kivinen@helsinki.fi	Clinical Groups	Women's Health and Reproduction Group
Elisabeth Widen	Institute for Molecular Medicine Finland (FIMM), HiLIFE, University of Helsinki, Helsink	elisabeth.widen@helsinki.fi	Clinical Groups	Women's Health and Reproduction Group
Taru Tukiainen	Institute for Molecular Medicine Finland (FIMM), HiLIFE, University of Helsinki, Helsink	taru.tukiainen@helsinki.fi	Clinical Groups	Women's Health and Reproduction Group
Mary Pat Reeve	Institute for Molecular Medicine Finland (FIMM), HiLIFE, University of Helsinki, Helsink	mary.reeve@helsinki.fi	Clinical Groups	Women's Health and Reproduction Group
Mark Daly	Institute for Molecular Medicine Finland (FIMM), HiLIFE, University of Helsinki, Helsinki, Finland; Broad Institute of MIT and Harvard; Massachusetts General Hospital	mark.daly@helsinki.fi	Clinical Groups	Women's Health and Reproduction Group
Niko Välimäki	University of Helsinki, Helsinki, Finland	niko.valimaki@helsinki.fi	Clinical Groups	Women's Health and Reproduction Group
Eija Laakkonen	University of Jyväskylä, Jyväskylä, Finland	eija.k.laakkonen@jyu.fi	Clinical Groups	Women's Health and Reproduction Group
Jaakko Tyrmi	University of Oulu, Oulu, Finland / University of Tampere, Tampere, Finland	jaakko.tyrmi@oulu.fi	Clinical Groups	Women's Health and Reproduction Group
Heidi Silven	University of Oulu, Oulu, Finland	heidi.silven@student.oulu.fi	Clinical Groups	Women's Health and Reproduction Group
Eeva Sliz	University of Oulu, Oulu, Finland	eeva.sliz@oulu.fi	Clinical Groups	Women's Health and Reproduction Group
Riikka Arffman	University of Oulu, Oulu, Finland	riikka.arffman@oulu.fi	Clinical Groups	Women's Health and Reproduction Group
Susanna Savukoski	University of Oulu, Oulu, Finland	susanna.savukoski@oulu.fi	Clinical Groups	Women's Health and Reproduction Group
Triin Laisk	Estonian biobank, Tartu, Estonia	triin.laisk@ut.ee	Clinical Groups	Women's Health and Reproduction Group
Natalia Pujol	Estonian biobank, Tartu, Estonia	natalia.pujolgualdo@oulu.fi	Clinical Groups	Women's Health and Reproduction Group
Mengzhen Liu	Abbvie, Chicago, IL, United States	mengzhen.liu@abbvie.com	Clinical Groups	Women's Health and Reproduction Group
Bridget Riley-Gillis	Abbvie, Chicago, IL, United States	bridget.rileygillis@abbvie.com	Clinical Groups	Women's Health and Reproduction Group
Rion Pendergrass	Genentech, San Francisco, CA, United States	penders2@gene.com	Clinical Groups	Women's Health and Reproduction Group
Janet Kumar	GlaxoSmithKline, Collegeville, PA, United States	janet.x.kumar@gsk.com	Clinical Groups	Women's Health and Reproduction Group
Kirsi Auro	GlaxoSmithKline, Espoo, Finland	kirsi.m.auro@gsk.com	Clinical Groups	Women's Health and Reproduction Group
Iiris Hovatta	University of Helsinki, Finland	iiris.hovatta@helsinki.fi	Clinical Groups	Depression group
Chia-Yen Chen	Biogen, Cambridge, MA, United States	chiayen.chen@biogen.com	Clinical Groups	Depression group
Erkki Isometsä	Hospital District of Helsinki and Uusimaa, Helsinki, Finland	erkki.isometsa@hus.fi	Clinical Groups	Depression group
Hanna Ollila	Institute for Molecular Medicine Finland (FIMM), HiLIFE, University of Helsinki, Helsink	hanna.m.ollila@helsinki.fi	Clinical Groups	Depression group
Jaana Suvisaari	Finnish Institute for Health and Welfare (THL), Helsinki, Finland	jaana.suvisaari@thl.fi	Clinical Groups	Depression group
Antti Mäkitie	Department of Otorhinolaryngology - Head and Neck Surgery, University of Helsinki and Helsinki University Hospital, Helsinki, Finland	antti.makitie@helsinki.fi	Clinical Groups	ENT (ear, nose and throath) Group
Argyro Bizaki-Vallaskang	Pirkanmaa Hospital District, Tampere, Finland	argyro.bizaki-vallaskangas@tuni.fi	Clinical Groups	ENT (ear, nose and throath) Group
Sanna Toppila-Salmi	University of Eastern Finland and Kuopio University Hospital, Department of Otorhinolaryngology, Kuopio, Finland and Department of Allergy, Helsinki University Hospital and University of Helsinki, Finland	sanna.salmi@helsinki.fi	Clinical Groups	ENT (ear, nose and throath) Group
Tytti Willberg	Hospital District of Southwest Finland, Turku, Finland	tytti.willberg@tyks.fi	Clinical Groups	ENT (ear, nose and throath) Group
Elmo Saarentaus	Institute for Molecular Medicine Finland (FIMM), HiLIFE, University of Helsinki, Helsink	elmo.saarentaus@helsinki.fi	Clinical Groups	ENT (ear, nose and throath) Group
Antti Aarnisalo	Hospital District of Helsinki and Uusimaa, Helsinki, Finland	antti.aarnisalo@hus.fi	Clinical Groups	ENT (ear, nose and throath) Group
Eveliina Salminen	Hospital District of Helsinki and Uusimaa, Helsinki, Finland	eveliina.e.salminen@hus.fi	Clinical Groups	ENT (ear, nose and throath) Group
Elisa Rahikkala	Northern Ostrobothnia Hospital District, Oulu, Finland	elisa.rahikkala@ppshp.fi	Clinical Groups	ENT (ear, nose and throath) Group
Johannes Kettunen	Northern Ostrobothnia Hospital District, Oulu, Finland	johannes.kettunen@oulu.fi	Clinical Groups	ENT (ear, nose and throath) Group
Kristiina Aittomäki	Department of Medical Genetics, Helsinki University Central Hospital, Helsinki, Finland	kristiina.aittomaki@helsinki.fi	Clinical Groups	POI (premature ovarian failure) Group
Fredrik Åberg	Transplantation and Liver Surgery Clinic, Helsinki University Hospital, Helsinki University, Helsinki, Finland	fredrik.aberg@helsinki.fi	Clinical Groups	LiverScore Group
Mitja Kurki	Institute for Molecular Medicine Finland (FIMM), HiLIFE, University of Helsinki, Helsinki, Finland; Broad Institute, Cambridge, MA, United States	mkurki@broadinstitute.org	FinnGen Analysis working g	FinnGen Analysis working group
Samuli Ripatti	Institute for Molecular Medicine Finland (FIMM), HiLIFE, University of Helsinki, Helsink	samuli.ripatti@helsinki.fi	FinnGen Analysis working g	FinnGen Analysis working group
Mark Daly	Institute for Molecular Medicine, Finland (FIMM), HiLIFE, University of Helsinki, Helsinki, Finland; Broad Institute of MIT and Harvard; Massachusetts General Hospital	mark.daly@helsinki.fi	FinnGen Analysis working g	FinnGen Analysis working group
Juha Karjalainen	Institute for Molecular Medicine Finland (FIMM), HiLIFE, University of Helsinki, Helsink	juha.karjalainen@helsinki.fi	FinnGen Analysis working g	FinnGen Analysis working group
Aki Havulinna	Institute for Molecular Medicine Finland (FIMM), HiLIFE, University of Helsinki, Helsinki,	aki.havulinna@helsinki.fi	FinnGen Analysis working g	FinnGen Analysis working group
Juha Mehtonen	Institute for Molecular Medicine Finland (FIMM), HiLIFE, University of Helsinki, Helsink	juha.mehtonen@helsinki.fi	FinnGen Analysis working g	FinnGen Analysis working group
Priit Palta	Institute for Molecular Medicine Finland (FIMM), HiLIFE, University of Helsinki, Helsink	priit.palta@helsinki.fi	FinnGen Analysis working g	FinnGen Analysis working group
Shabbeer Hassan	Institute for Molecular Medicine Finland (FIMM), HiLIFE, University of Helsinki, Helsink	shabbeer.hassan@helsinki.fi	FinnGen Analysis working g	FinnGen Analysis working group
Pietro Della Briotta Parol	Institute for Molecular Medicine Finland (FIMM), HiLIFE, University of Helsinki, Helsink	pietro.dellabriottaparolo@helsinki.fi	FinnGen Analysis working g	FinnGen Analysis working group
Wei Zhou	Broad Institute, Cambridge, MA, United States	wzhou@broadinstitute.org	FinnGen Analysis working g	FinnGen Analysis working group
Mutaamba Maasha	Broad Institute, Cambridge, MA, United States	mmaasha@broadinstitute.org	FinnGen Analysis working g	FinnGen Analysis working group
Shabbeer Hassan	Institute for Molecular Medicine Finland (FIMM), HiLIFE, University of Helsinki, Helsink	shabbeer.hassan@helsinki.fi	FinnGen Analysis working g	FinnGen Analysis working group
Susanna Lemmelä	Institute for Molecular Medicine Finland (FIMM), HiLIFE, University of Helsinki, Helsink	susanna.lemmela@helsinki.fi	FinnGen Analysis working g	FinnGen Analysis working group
Manuel Rivas	University of Stanford, Stanford, CA, United States	mrivas@stanford.edu	FinnGen Analysis working g	FinnGen Analysis working group

Aarno Palotie	Institute for Molecular Medicine Finland (FIMM), HiLIFE, University of Helsinki, Helsink	aarno.palotie@helsinki.fi	FinnGen Analysis working g	FinnGen Analysis working group
Aoxing Liu	Institute for Molecular Medicine Finland (FIMM), HiLIFE, University of Helsinki, Helsink	aoxing.liu@helsinki.fi	FinnGen Analysis working g	FinnGen Analysis working group
Arto Lehisto	Institute for Molecular Medicine Finland (FIMM), HiLIFE, University of Helsinki, Helsink	arto.lehisto@helsinki.fi	FinnGen Analysis working g	FinnGen Analysis working group
Andrea Ganna	Institute for Molecular Medicine Finland (FIMM), HiLIFE, University of Helsinki, Helsink	aganna@broadinstitute.org	FinnGen Analysis working g	FinnGen Analysis working group
Vincent Llorens	Institute for Molecular Medicine Finland (FIMM), HiLIFE, University of Helsinki, Helsink	vincent.llorens@helsinki.fi	FinnGen Analysis working g	FinnGen Analysis working group
Hannele Laivuori	Institute for Molecular Medicine Finland (FIMM), HiLIFE, University of Helsinki, Helsink	hannele.laivuori@helsinki.fi	FinnGen Analysis working g	FinnGen Analysis working group
Taru Tukiainen	Institute for Molecular Medicine Finland (FIMM), HiLIFE, University of Helsinki, Helsink	taru.tukiainen@helsinki.fi	FinnGen Analysis working g	FinnGen Analysis working group
Mary Pat Reeve	Institute for Molecular Medicine Finland (FIMM), HiLIFE, University of Helsinki, Helsink	mary.reeve@helsinki.fi	FinnGen Analysis working g	FinnGen Analysis working group
Henrike Heyne	Institute for Molecular Medicine Finland (FIMM), HiLIFE, University of Helsinki, Helsink	hheyne@broadinstitute.org	FinnGen Analysis working g	FinnGen Analysis working group
Nina Mars	Institute for Molecular Medicine Finland (FIMM), HiLIFE, University of Helsinki, Helsink	nina.mars@helsinki.fi	FinnGen Analysis working g	FinnGen Analysis working group
Joel Rämö	Institute for Molecular Medicine Finland (FIMM), HiLIFE, University of Helsinki, Helsink	joel.ramo@helsinki.fi	FinnGen Analysis working g	FinnGen Analysis working group
Elmo Saarentaus	Institute for Molecular Medicine Finland (FIMM), HiLIFE, University of Helsinki, Helsink	elmo.saarentaus@helsinki.fi	FinnGen Analysis working g	FinnGen Analysis working group
Hanna Ollila	Institute for Molecular Medicine Finland (FIMM), HiLIFE, University of Helsinki, Helsink	hanna.m.ollila@helsinki.fi	FinnGen Analysis working g	FinnGen Analysis working group
Rodos Rodosthenous	Institute for Molecular Medicine Finland (FIMM), HiLIFE, University of Helsinki, Helsink	rodos.rodosthenous@helsinki.fi	FinnGen Analysis working g	FinnGen Analysis working group
Satu Strausz	Institute for Molecular Medicine Finland (FIMM), HiLIFE, University of Helsinki, Helsink	satu.strausz@helsinki.fi	FinnGen Analysis working g	FinnGen Analysis working group
Tuula Palotie	University of Helsinki and Hospital District of Helsinki and Uusimaa, Helsinki, Finland	tuula.palotie@helsinki.fi	FinnGen Analysis working g	FinnGen Analysis working group
Kimmo Palin	University of Helsinki, Helsinki, Finland	kimmo.palin@helsinki.fi	FinnGen Analysis working g	FinnGen Analysis working group
Javier Garcia-Tabuenca	University of Tampere, Tampere, Finland	javier.graciatabuenca@tuni.fi	FinnGen Analysis working g	FinnGen Analysis working group
Harri Siirtola	University of Tampere, Tampere, Finland	harri.siirtola@tuni.fi	FinnGen Analysis working g	FinnGen Analysis working group
Tuomo Kiiskinen	Institute for Molecular Medicine Finland (FIMM), HiLIFE, University of Helsinki, Helsink	tuomo.kiiskinen@helsinki.fi	FinnGen Analysis working g	FinnGen Analysis working group
Jiwoo Lee	Institute for Molecular Medicine Finland (FIMM), HiLIFE, University of Helsinki, Helsinki, Finland; Broad Institute, Cambridge, MA, United States	jiwoo.lee@helsinki.fi	FinnGen Analysis working g	FinnGen Analysis working group
Kristin Tsuo	Institute for Molecular Medicine Finland (FIMM), HiLIFE, University of Helsinki, Helsinki, Finland; Broad Institute, Cambridge, MA, United States	kristintsuo@fas.harvard.edu	FinnGen Analysis working g	FinnGen Analysis working group
Amanda Elliott	Institute for Molecular Medicine Finland (FIMM), HiLIFE, University of Helsinki, Helsinki, Finland; Broad Institute, Cambridge, MA, USA and Massachusetts General Hospital, Boston, MA, USA	aelliott@broadinstitute.org	FinnGen Analysis working g	FinnGen Analysis working group
Kati Kristiansson	THL Biobank / Finnish Institute for Health and Welfare (THL), Helsinki, Finland	kati.kristiansson@thl.fi	FinnGen Analysis working g	FinnGen Analysis working group
Mikko Arvas	Finnish Red Cross Blood Service / Finnish Hematology Registry and Clinical Biobank, Helsinki, Finland	mikko.arvas@veripalvelu.fi	FinnGen Analysis working g	FinnGen Analysis working group
Kati Hyvärinen	Finnish Red Cross Blood Service, Helsinki, Finland	kati.hyvarinen@veripalvelu.fi	FinnGen Analysis working g	FinnGen Analysis working group
Jarmo Ritari	Finnish Red Cross Blood Service, Helsinki, Finland	jarmo.ritari@veripalvelu.fi	FinnGen Analysis working g	FinnGen Analysis working group
Olli Carpén	Helsinki Biobank / Helsinki University and Hospital District of Helsinki and Uusimaa, Helsinki	olli.carpen@helsinki.fi	FinnGen Analysis working g	FinnGen Analysis working group
Johannes Kettunen	Northern Finland Biobank Borealis / University of Oulu / Northern Ostrobothnia Hospital District, Oulu, Finland	johannes.kettunen@oulu.fi	FinnGen Analysis working g	FinnGen Analysis working group
Katri Pylkäs	University of Oulu, Oulu, Finland	katri.pylkas@oulu.fi	FinnGen Analysis working g	FinnGen Analysis working group
Eeva Sliz	University of Oulu, Oulu, Finland	eeva.sliz@oulu.fi	FinnGen Analysis working g	FinnGen Analysis working group
Minna Karjalainen	University of Oulu, Oulu, Finland	minna.k.karjalainen@oulu.fi	FinnGen Analysis working g	FinnGen Analysis working group
Tuomo Mantere	Northern Finland Biobank Borealis / University of Oulu / Northern Ostrobothnia Hospital District, Oulu, Finland	tuomo.mantere@oulu.fi	FinnGen Analysis working g	FinnGen Analysis working group
Eeva Kangasniemi	Finnish Clinical Biobank Tampere / University of Tampere / Pirkanmaa Hospital District, Tampere, Finland	eeva.kangasniemi@pshp.fi	FinnGen Analysis working g	FinnGen Analysis working group
Sami Heikkinen	University of Eastern Finland, Kuopio, Finland	sami.heikkinen@uef.fi	FinnGen Analysis working g	FinnGen Analysis working group
Arto Mannermaa	Biobank of Eastern Finland / University of Eastern Finland / Northern Savo Hospital District, Kuopio, Finland	arto.mannermaa@uef.fi	FinnGen Analysis working g	FinnGen Analysis working group
Eija Laakkonen	University of Jyväskylä, Jyväskylä, Finland	eija.k.laakkonen@jyu.fi	FinnGen Analysis working g	FinnGen Analysis working group
Nina Pitkänen	Auria Biobank / University of Turku / Hospital District of Southwest Finland, Turku, Finland	Niina.Pitkanen@tyks.fi	FinnGen Analysis working g	FinnGen Analysis working group
Samuel Lessard	Translational Sciences, Sanofi R&D, Framingham, MA, USA	samuel.lessard@sanofi.com	FinnGen Analysis working g	FinnGen Analysis working group
Clément Chatelain	Translational Sciences, Sanofi R&D, Framingham, MA, USA	clement.chatelain@sanofi.com	FinnGen Analysis working g	FinnGen Analysis working group
Lila Kallio	Auria Biobank / University of Turku / Hospital District of Southwest Finland, Turku, Finland	Lila.Kallio@tyks.fi	Biobank directors	Biobank directors
Tiina Wahlfors	THL Biobank / Finnish Institute for Health and Welfare (THL), Helsinki, Finland	tiina.wahlfors@thl.fi	Biobank directors	Biobank directors
Jukka Partanen	Finnish Red Cross Blood Service / Finnish Hematology Registry and Clinical Biobank, Helsinki, Finland	jukka.partanen@veripalvelu.fi	Biobank directors	Biobank directors
Eero Punkka	Helsinki Biobank / Helsinki University and Hospital District of Helsinki and Uusimaa, Helsinki	eero.punkka@hus.fi	Biobank directors	Biobank directors
Raisa Serpi	Northern Finland Biobank Borealis / University of Oulu / Northern Ostrobothnia Hospital District, Oulu, Finland	raisa.serpi@ppshp.fi	Biobank directors	Biobank directors
Sanna Siltanen	Finnish Clinical Biobank Tampere / University of Tampere / Pirkanmaa Hospital District, Tampere, Finland	sanna.siltanen@pshp.fi	Biobank directors	Biobank directors
Veli-Matti Kosma	Biobank of Eastern Finland / University of Eastern Finland / Northern Savo Hospital District, Kuopio, Finland	veli-matti.kosma@uef.fi	Biobank directors	Biobank directors
Teijo Kuopio	Central Finland Biobank / University of Jyväskylä / Central Finland Health Care District, Jyväskylä, Finland	teijo.kuopio@ksshp.fi	Biobank directors	Biobank directors
Anu Jalanko	Institute for Molecular Medicine Finland (FIMM), HiLIFE, University of Helsinki, Helsink	anu.jalanko@helsinki.fi	FinnGen Teams	Administration
Huei-Yi Shen	Institute for Molecular Medicine Finland (FIMM), HiLIFE, University of Helsinki, Helsink	huei-yi.shen@helsinki.fi	FinnGen Teams	Administration
Risto Kajanne	Institute for Molecular Medicine Finland (FIMM), HiLIFE, University of Helsinki, Helsink	risto.kajanne@helsinki.fi	FinnGen Teams	Administration
Mervi Aavikko	Institute for Molecular Medicine Finland (FIMM), HiLIFE, University of Helsinki, Helsink	mervi.aavikko@helsinki.fi	FinnGen Teams	Administration
Helen Cooper	Institute for Molecular Medicine Finland (FIMM), HiLIFE, University of Helsinki, Helsink	helen.cooper@helsinki.fi	FinnGen Teams	Administration
Denise Öller	Institute for Molecular Medicine Finland (FIMM), HiLIFE, University of Helsinki, Helsink	denise.oller@helsinki.fi	FinnGen Teams	Administration
Rasko Leinonen	Institute for Molecular Medicine Finland (FIMM), HiLIFE, University of Helsinki, Helsink	rasko@ebi.ac.uk	FinnGen Teams	Administration
Henna Palin	Finnish Clinical Biobank Tampere / University of Tampere / Pirkanmaa Hospital District, Tampere, Finland	henna.palin@pshp.fi	FinnGen Teams	Administration
Malla-Maria Linna	Helsinki Biobank / Helsinki University and Hospital District of Helsinki and Uusimaa, Helsinki	malla-maria.linna@hus.fi	FinnGen Teams	Administration
Mitja Kurki	Institute for Molecular Medicine Finland (FIMM), HiLIFE, University of Helsinki, Helsinki, Finland; Broad Institute, Cambridge, MA, United States	mkurki@broadinstitute.org	FinnGen Teams	Analysis
Juha Karjalainen	Institute for Molecular Medicine Finland (FIMM), HiLIFE, University of Helsinki, Helsink	juha.karjalainen@helsinki.fi	FinnGen Teams	Analysis
Pietro Della Briotta Parol	Institute for Molecular Medicine Finland (FIMM), HiLIFE, University of Helsinki, Helsink	pietro.dellabriottaparolo@helsinki.fi	FinnGen Teams	Analysis
Arto Lehisto	Institute for Molecular Medicine Finland (FIMM), HiLIFE, University of Helsinki, Helsink	arto.lehisto@helsinki.fi	FinnGen Teams	Analysis
Juha Mehtonen	Institute for Molecular Medicine Finland (FIMM), HiLIFE, University of Helsinki, Helsink	juha.mehtonen@helsinki.fi	FinnGen Teams	Analysis
Wei Zhou	Broad Institute, Cambridge, MA, United States	wzhou@broadinstitute.org	FinnGen Teams	Analysis
Masahiro Kanai	Broad Institute, Cambridge, MA, United States	mkanai@broadinstitute.org	FinnGen Teams	Analysis
Mutaamba Maasha	Broad Institute, Cambridge, MA, United States	mmaasha@broadinstitute.org	FinnGen Teams	Analysis
Zhili Zheng	Broad Institute, Cambridge, MA, United States	zhengzhi@broadinstitute.org	FinnGen Teams	Analysis
Hannele Laivuori	Institute for Molecular Medicine Finland (FIMM), HiLIFE, University of Helsinki, Helsink	hannele.laivuori@helsinki.fi	FinnGen Teams	Clinical Endpoint Development
Aki Havulinna	Institute for Molecular Medicine Finland (FIMM), HiLIFE, University of Helsinki, Helsinki,	aki.havulinna@helsinki.fi	FinnGen Teams	Clinical Endpoint Development
Susanna Lemmelä	Institute for Molecular Medicine Finland (FIMM), HiLIFE, University of Helsinki, Helsink	susanna.lemmela@helsinki.fi	FinnGen Teams	Clinical Endpoint Development
Tuomo Kiiskinen	Institute for Molecular Medicine Finland (FIMM), HiLIFE, University of Helsinki, Helsink	tuomo.kiiskinen@helsinki.fi	FinnGen Teams	Clinical Endpoint Development
L. Elisa Lahtela	Institute for Molecular Medicine Finland (FIMM), HiLIFE, University of Helsinki, Helsink	laura.lahtela@helsinki.fi	FinnGen Teams	Clinical Endpoint Development
Mari Kaunisto	Institute for Molecular Medicine Finland (FIMM), HiLIFE, University of Helsinki, Helsink	mari.kaunisto@helsinki.fi	FinnGen Teams	Communication
Elina Kilpeläinen	Institute for Molecular Medicine Finland (FIMM), HiLIFE, University of Helsinki, Helsink	elina.kilpelainen@helsinki.fi	FinnGen Teams	E-Science
Timo P. Sipilä	Institute for Molecular Medicine Finland (FIMM), HiLIFE, University of Helsinki, Helsink	timo.p.sipila@helsinki.fi	FinnGen Teams	E-Science
Oluwaseun Alexander Da	Institute for Molecular Medicine Finland (FIMM), HiLIFE, University of Helsinki, Helsink	alexander.dada@helsinki.fi	FinnGen Teams	E-Science
Awaisa Ghazal	Institute for Molecular Medicine Finland (FIMM), HiLIFE, University of Helsinki, Helsink	awaisa.ghazal@helsinki.fi	FinnGen Teams	E-Science
Anastasia Kytölä	Institute for Molecular Medicine Finland (FIMM), HiLIFE, University of Helsinki, Helsink	anastasia.shcherban@helsinki.fi	FinnGen Teams	E-Science
Rigbe Weldatsadik	Institute for Molecular Medicine Finland (FIMM), HiLIFE, University of Helsinki, Helsink	rigbe.weldatsadik@helsinki.fi	FinnGen Teams	E-Science
Sanni Ruotsalainen	Institute for Molecular Medicine Finland (FIMM), HiLIFE, University of Helsinki, Helsink	sanni.ruotsalainen@helsinki.fi	FinnGen Teams	E-Science
Kati Donner	Institute for Molecular Medicine Finland (FIMM), HiLIFE, University of Helsinki, Helsink	kati.donner@helsinki.fi	FinnGen Teams	Genotyping
Timo P. Sipilä	Institute for Molecular Medicine Finland (FIMM), HiLIFE, University of Helsinki, Helsink	timo.p.sipila@helsinki.fi	FinnGen Teams	Genotyping
Anu Loukola	Helsinki Biobank / Helsinki University and Hospital District of Helsinki and Uusimaa, Helsinki	anu.loukola@hus.fi	FinnGen Teams	Sample Collection Coordination
Päivi Laiho	THL Biobank / Finnish Institute for Health and Welfare (THL), Helsinki, Finland	paivi.laiho@thl.fi	FinnGen Teams	Sample Logistics
Tuuli Sistonen	THL Biobank / Finnish Institute for Health and Welfare (THL), Helsinki, Finland	tuuli.sistonen@thl.fi	FinnGen Teams	Sample Logistics
Essi Kaiharju	THL Biobank / Finnish Institute for Health and Welfare (THL), Helsinki, Finland	essi.kaiharju@thl.fi	FinnGen Teams	Sample Logistics
Markku Laukkanen	THL Biobank / Finnish Institute for Health and Welfare (THL), Helsinki, Finland	markku.laukkanen@thl.fi	FinnGen Teams	Sample Logistics
Elina Järvensivu	THL Biobank / Finnish Institute for Health and Welfare (THL), Helsinki, Finland	elina.jarvensivu@thl.fi	FinnGen Teams	Sample Logistics
Sini Lähteenmäki	THL Biobank / Finnish Institute for Health and Welfare (THL), Helsinki, Finland	sini.lahteenmaki@thl.fi	FinnGen Teams	Sample Logistics
Lotta Männikkö	THL Biobank / Finnish Institute for Health and Welfare (THL), Helsinki, Finland	lotta.mannikko@thl.fi	FinnGen Teams	Sample Logistics
Regis Wong	THL Biobank / Finnish Institute for Health and Welfare (THL), Helsinki, Finland	regis.wong@thl.fi	FinnGen Teams	Sample Logistics
Auli Toivola	THL Biobank / Finnish Institute for Health and Welfare (THL), Helsinki, Finland	auli.toivola@thl.fi	FinnGen Teams	Sample Logistics
Minna Brunfeldt	THL Biobank / Finnish Institute for Health and Welfare (THL), Helsinki, Finland	minna.brunfeldt@thl.fi	FinnGen Teams	Registry Data Operations
Hannele Mattsson	THL Biobank / Finnish Institute for Health and Welfare (THL), Helsinki, Finland	hannele.mattsson@thl.fi	FinnGen Teams	Registry Data Operations
Kati Kristiansson	THL Biobank / Finnish Institute for Health and Welfare (THL), Helsinki, Finland	kati.kristiansson@thl.fi	FinnGen Teams	Registry Data Operations
Susanna Lemmelä	Institute for Molecular Medicine Finland (FIMM), HiLIFE, University of Helsinki, Helsink	susanna.lemmela@helsinki.fi	FinnGen Teams	Registry Data Operations
Sami Koskelainen	THL Biobank / Finnish Institute for Health and Welfare (THL), Helsinki, Finland	sami.koskelainen@thl.fi	FinnGen Teams	Registry Data Operations
Tero Hiekkalinna	THL Biobank / Finnish Institute for Health and Welfare (THL), Helsinki, Finland	tero.hiekkalinna@helsinki.fi	FinnGen Teams	Registry Data Operations
Teemu Paajanen	THL Biobank / Finnish Institute for Health and Welfare (THL), Helsinki, Finland	teemu.paajanen@thl.fi	FinnGen Teams	Registry Data Operations
Priit Palta	Institute for Molecular Medicine Finland (FIMM), HiLIFE, University of Helsinki, Helsink	priit.palta@helsinki.fi	FinnGen Teams	Sequencing Informatics
Shuang Luo	Institute for Molecular Medicine Finland (FIMM), HiLIFE, University of Helsinki, Helsink	shuang.luo@helsinki.fi	FinnGen Teams	Sequencing Informatics
Tarja Laitinen	Pirkanmaa Hospital District, Tampere, Finland	tarja.laitinen@pshp.fi	FinnGen Teams	Trajectory

Mary Pat Reeve	Institute for Molecular Medicine Finland (FIMM), HiLIFE, University of Helsinki, Helsinl	mary.reeve@helsinki.fi	FinnGen Teams	**Trajectory**
Shanmukha Sampath Pa	Institute for Molecular Medicine Finland (FIMM), HiLIFE, University of Helsinki, Helsinl	sam.padmanabhuni@helsinki.fi	FinnGen Teams	**Trajectory**
Marianna Niemi	University of Tampere, Tampere, Finland	marianna.niemi@tuni.fi	FinnGen Teams	**Trajectory**
Harri Siirtola	University of Tampere, Tampere, Finland	harri.siirtola@tuni.fi	FinnGen Teams	**Trajectory**
Javier Gracia-Tabuenca	University of Tampere, Tampere, Finland	javier.graciatabuenca@tuni.fi	FinnGen Teams	**Trajectory**
Mika Helminen	University of Tampere, Tampere, Finland	mika.helminen@tuni.fi	FinnGen Teams	**Trajectory**
Tiina Luukkaala	University of Tampere, Tampere, Finland	tiina.luukkaala@tuni.fi	FinnGen Teams	**Trajectory**
Iida Vähätalo	University of Tampere, Tampere, Finland	iida.vahatalo@epshp.fi	FinnGen Teams	**Trajectory**
Jyrki Tammerluoto	Institute for Molecular Medicine Finland (FIMM), HiLIFE, University of Helsinki, Helsinl	jyrki.tammerluoto@helsinki.fi	FinnGen Teams	**Data protection officer**
Marco Hautalahti	Finnish Biobank Cooperative - FINBB	marco.hautalahti@finbb.fi	FinnGen Teams	**FINBB - Finnish biobank cooperative**
Johanna Mäkelä	Finnish Biobank Cooperative - FINBB	johanna.makela@finbb.fi	FinnGen Teams	**FINBB - Finnish biobank cooperative**
Sarah Smith	Finnish Biobank Cooperative - FINBB	sarah.smith@finbb.fi	FinnGen Teams	**FINBB - Finnish biobank cooperative**
Tom Southerington	Finnish Biobank Cooperative - FINBB	tom.southerington@finbb.fi	FinnGen Teams	**FINBB - Finnish biobank cooperative**
Petri Lehto	Finnish Biobank Cooperative - FINBB	petri.lehto@finbb.fi	FinnGen Teams	**FINBB - Finnish biobank cooperative**
